# Transcriptome profiling of *Cucumis metuliferus* infected by *Meloidogyne incognita* provides new insights into putative defense regulatory network in Cucurbitaceae

**DOI:** 10.1038/s41598-017-03563-6

**Published:** 2017-06-14

**Authors:** Jian Ling, Zhenchuan Mao, Mingjuan Zhai, Feng Zeng, Yuhong Yang, Bingyan Xie

**Affiliations:** Institute of Vegetables and Flowers, Chinese Academy of Agricultural Sciences, 12 Zhongguancun South Street, Beijing, 100081 China

## Abstract

Root-knot nematodes (RKN) represent extensive challenges to Cucurbitaceae crops. However, *Cucumis metuliferus* (*Cm*) is known to be resistant to *Meloidogyne incognita* (*Mi*) infections. Thus, analysis of differentially expressed genes may lead to a comprehensive gene expression profiling of the incompatible *Cm*-*Mi* interaction. In this study, the time-course transcriptome of *Cm* against *Mi* infection was monitored using RNA-Seq. More than 170000 transcripts were examined in *Cm* roots, and 2430 genes were subsequently identified as differentially expressed in response to *Mi* infection. Based on function annotation and orthologs finding, the potential mechanism of transcriptional factor, cytoskeleton, pathogen-related genes and plant hormone were assessed at the transcription level. A comparison of gene expression levels between *Mi*-infected *Cm* and cucumber plants revealed that cytoskeleton-related genes are key regulators of *Cm* resistance to *Mi*. We herein discuss the dual nature of cytoskeleton-related genes in the susceptibility and resistance of plant hosts to *Mi*. Our observations provide novel insights into the responses of *Cm* to *Mi* at the transcriptome level. The data generated in this study may be useful for elucidating the mechanism underlying resistance to RKNs in cucurbitaceous crops.

## Introduction

Plant–parasitic root-knot nematodes (RKNs) are among the most dispersed plant pathogens responsible for considerable yield loses lost in various economic crops, including rice, soybean, cucumber and so on^1, 2^. Once infected, the second-stage juvenile nematodes can migrate into host plant root cells, and manipulate the normal root physiology by secreting several so-called effectors. This ultimately results in the generation of a specialized nematode feeding site (NFS) consisting of four to eight root cells that have been converted into giant cells^3^. The giant cells are employed by RKN to divert plant nutrients to supply the metabolic energy necessary for RKN’s development. Concomitant with the formation of giant cell, the surrounding root tissue forms root gall, in which RKNs develop continuously and eventually become adults^4^.

The transformation of root cells into NFS structures induced by RKN is related to the complex physiological processes change of host plants. RKN has the ability to manipulate these processes for facilitating their invading. Recent studies indicates that RKN can usurp host plant cell cycle by inducing the genes associated with cell cycle such as cyclin-dependent kinase (CDKs)^5^. In RKN infection sites, *XYLOGLUCAN ENDO-TRANSGLYCOSYLASE/HYDROLASE* (*XTHs*) gene family, responsible for cell wall reinforcement, was induced, suggesting RKN can manipulate plant cell expansion^6^. RKN can also induce partial depolymerisation of the microtubule, suggesting RKN can regulate cytoskeleton of host plant^7^. On the other hands, when host plants perceive RKN, they can respond with a defense reaction against the invading RKN. An important defense response involves the induction of hormone pathways that play an important role in plant defense response against pathogen invading. For example, jasmonic acid (JA) and ethylene (ET) pathways were induced at early time points in RKN-infected plants^8^. Auxin manipulation is known to be an important process during initiation and early development of NFS of sedentary plant-parasitic nematodes^9^. Salicylic acid (SA)-related genes are strongly induced in infected roots^7, 10^. It has been noted that crosstalk between various hormones also exists in the host plant response against pathogens^9^. Beside of hormones, transcription factors are involved in the host plant responses to RKN invading. Gene expression profiling of nematode-infected Arabidopsis roots and of feeding cell indicated that transcription factors are key contributors to regulate the genes expression of hosts infected by nematode^11, 12^. Jin *et al*., reported that two Arabidopsis bHLH transcription factor genes interact in planta and positively influence cyst nematode parasitism^13^. WRKY transcription factor encoding genes are known to be widely involved in plants responses to nematode infection. Knocking down the expression of WRKY23 resulted in lower infection of the cyst nematode *Heterodera schachtii* ^14^. WRKY2 was preferentially induced during incompatible interactions of pepper plants with RKN^15^. In addition, pathogenesis-related genes are also involved in RKN invading. For example, the expression of PR-1, PR-2 and PR-5, was induced in both roots and leaves of RKN infected plants^16^.

Several high-throughput methods have been employed to analyze host plant gene expression level changes induced by an RKN infection, including gene chip arrays, suppression subtractive hybridization libraries (SSH), and RNA sequencing (RNA-seq)^17–20^. Transcriptome analysis based on RNA-seq is now recognized as a powerful approach to detect large numbers of genes, and it has been used widely to study the interactions between RKNs and their hosts^20^. Transcriptional analyses of galls at 3 days after an infection with the *Meloidogyne graminicola* (*Mg*) revealed that some plant hormone biosynthesis and signaling pathways are activated^17^. Beneventi *et al*. described the crosstalk between hormones and reactive oxygen species based on transcriptome of RKN infected soybean roots^18^.

*Cucumis* species belong to the Cucurbitaceae family, which includes several economically important crops, such as cucumber, melon, and watermelon. Despite their economic importance, cultivated Cucurbitaceae plants have always been vulnerable to serious RKN infections because they lack the RKN resistance genes^21^. In contrast, several wild melon species are reportedly resistant to *Mi* ^22, 23^. Among the *Mi*-resistant Cucumis species, the African horned melon *Cucumis metuliferus* (*Cm*) is known to be highly resistant to *Mi* ^21, 23^. In this study, we carried out a transcriptome analysis of *Cm* infected by *Mi* to investigate the *Mi*-resistance mechanism. To our knowledge, this is the first study of the interactions between Cucurbitaceae plant and RKN using transcriptome data. It provides a new insight into the responses of Cucurbitaceae plant to RKN infection, which are expected to be highly useful for dissecting the nematode resistant mechanism of Cucurbitaceae plant.

## Results

### *Mi* infection and development in cucumber and *Cm*

**Figure 1:**
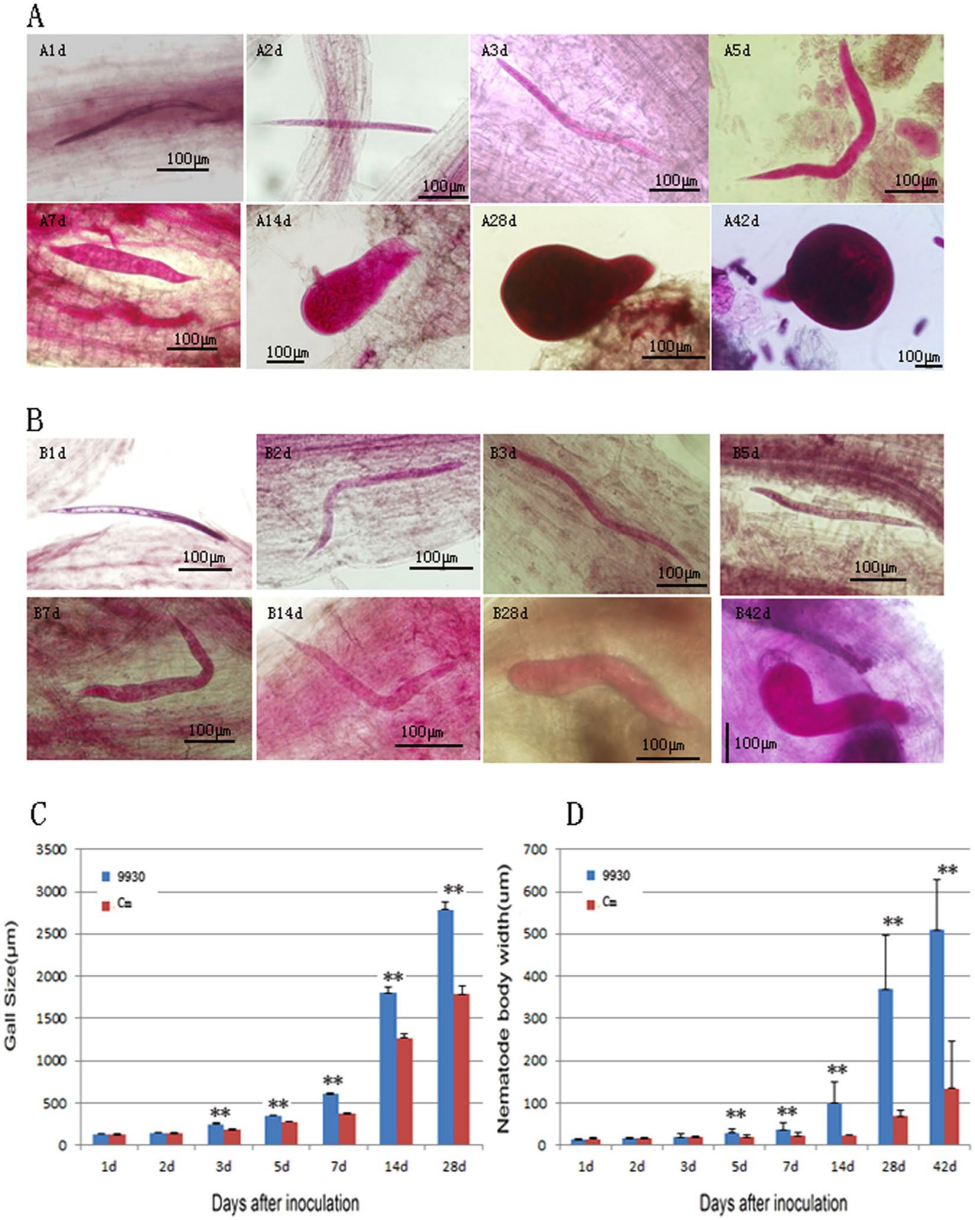
The development comparison of *Mi* between 9930 and *Cm*. (**A**) Represents 9930 and **B** represents *Cm*; The data followed (**A** or **B**) represents the days after inoculation; (**C**) comparison of gall size between 9930 and Cm; (**D**). comparison of nematode body width between 9930 and *Cm*. The **Indicate significant difference at 0.01 level of T-test.

In this study, we compared *Mi* development in the infected root tissues of *Cm* and cultivated cucumber (*Cucumis sativus* L.) inbred line 9930 at 1, 3, 5, 7, 14, 28 and 42 days after infection (DAI). We observed that *Mi* can invaded the roots of both 9930 and *Cm* plants (Fig. 1A,B and Supplementary Fig. S1). However, there were significant differences (p < 0.01) between 9930 and *Cm* roots from 3 to 28 DAI regarding gall size, which reflects the ability of *Mi* to invade roots (Fig. 1C). We also observed a significant difference in nematode body width between 9930 and Cm plants from 5 to 28 DAI (Fig. 1D). The average body width was 369.5 μm for nematodes in 9930 plants at 28 DAI, which was almost 6-fold greater than that for nematodes in *Cm* plants (i.e., 68.8 μm) at the same time point. The hysteresis associated with Mi development in the infected roots was more apparent in Cm plants than in 9930 plants (Fig. 1A and B). At 14 DAI, most of *Mi* that penetrate in 9930 roots developed into J3/J4 stages, while most of *Mi* in infected *Cm* roots were still at their J2 stage. Consequently, the nematode body width differed significantly between 9930 (99.2 μm) and Cm (23.4 μm) plants. At 28 DAI, all *M*i in 9930 roots were at J3/J4 stages while only a small number of *Mi* in *Cm* roots were at J3/J4 stages. At 42 DAI, we observed *Mi* spawning behavior in 9930 roots, which was in contrast to most of the *Mi* nematodes in *Cm* roots, which were still at the J2 stage. Our results indicate that *Cm* can inhibit *Mi* development better than 9930 plants.

### *De novo* RNA-seq assembly and annotation of non-redundant unigenes

We constructed 12 cDNA libraries for RNA-seq analyses of uninfected *Cm* roots (control) and Mi-infected *Cm* roots at three time points (3, 14, and 28 DAI). The analyses were conducted with two biological replicates for each time point. A summary of the RNA-seq transcriptome data in the 12 libraries is provided in Table 1. On average, each library yielded 22 million high quality reads and roughly 4.48 GB of nucleotides. Because the whole-genome sequence of *Cm* is not available, the valid reads from the 12 libraries were merged for de novo assembly using trinity software. The assembly statistics are given in Table 2. A total of 178,059 transcripts longer than 200 bp were obtained. The total length of all the transcripts was approximately 157 Mb. The longest transcript for each locus was taken as the unigene, resulting in 138,390 unigenes with an average length of 680 bp. Functional annotation of the transcripts was performed using BLASTX searches against various public protein databases. Of the 178,059 transcripts, 88,965 (49.96%) matched to known protein sequences in the NR database, 60,156 (33.78%) matched sequences in SWISSPROT database, 41919(23.54%) in the KEGG database, 68838(38.67%) in the KOG database, 45785(25.71%) in the PFAM database. The expression levels of transcripts were obtained using RSEM package of the TRINITY software. Analyses of the transcript levels revealed that the results for the biological replicates were highly correlated (average 96.47%) at each time point (see Supplementary Fig. S2).

**Table 1:** The statistics of RNA-seq sequencing data in this study.

	3dc.ex1	3dc.ex2	3dt.ex1	3dt.ex2	14dc.ex1	14dc.ex2
reads	21,233,705	22,000,102	24,889,149	23,877,916	26,533,935	21,728,330
Bases(Gb)	4.25	4.40	5.00	4.78	5.31	4.35
Map ratio (%)	91.42	90.60	90.02	92.12	89.96	90.52
	**14dt.ex1**	**14dt.ex2**	**28dc.ex1**	**28dc.ex2**	**28dt.ex1**	**28dt.ex2**
reads	20,368,323	23,906,752	23,767,821	20,251,127	20,539,857	19,498,421
Bases(Gb)	4.07	4.81	4.78	4.05	4.11	3.90
Map ratio (%)	91.86	91.84	92.02	91.88	89.86	92.51
	**reads**		**Bases(Gb)**		**Map ratio**	
average	22,382,953		4.48		91.30	

*Dc and dt represent the control and the treatment in the same time point, respectively; ex1 and ex2 represent two biological replicates.

**Table 2:** The transcripts and unigenes clustered from the de novo assembly.

	Number	N50	Average length	Max length	Min length	Total length
transcripts	178059	1677	883.48	21856	201	15,7311,565
unigenes	138390	1079	680.02	21856	201	94,105,200

### Identification and functional classification of differentially expressed unigenes

**Figure 2:**
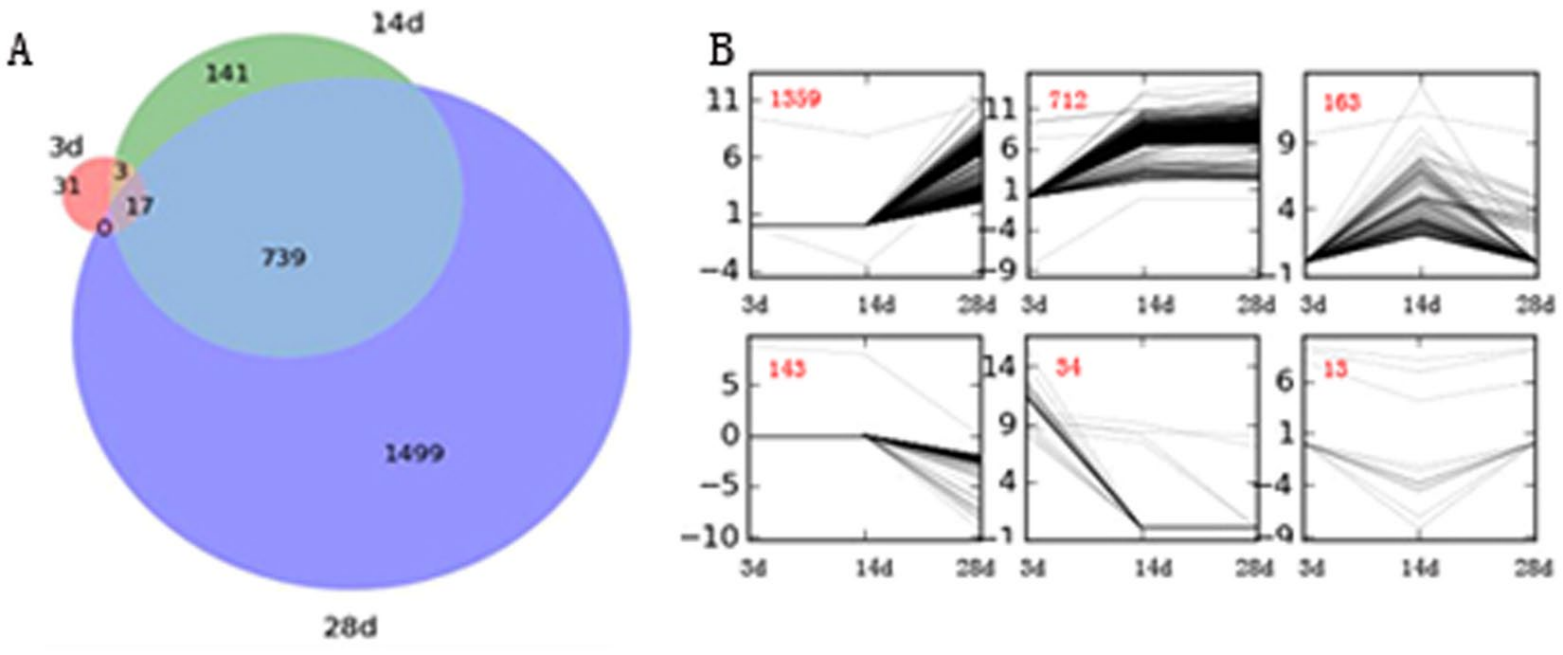
The DEUs and clustering analysis of the DEUs. (**A**) The Venn diagrams of DEUs at 3d, 14d and 28d treatment. (**B**) The clustering analysis of DEUs. The black lines show expression patterns. The red numbers on the left top represent the number of DEUs in each cluster. X-axis represents the treatment time (3d, 14d and 28d). Y-axis represents the log2 fold change compared with controls.

We identified differentially expressed unigenes (DEUs) by comparing the transcriptome data of the *Mi*-treated and control plants at the 3, 14, and 28 DAI time points. A total of 2430 DEUs were identified, most (2276/2430) of which were up-regulated (Supplementary Table S1). Of 2430 DEUs, 51 showed a differential expression pattern in 3d treatment, 900 in 14d treatment and 2255 in 28d treatment (Fig. 2A). The time series analysis suggested that the expression patterns of DEUs could be grouped into six clusters, with most (2051/2430) exhibiting higher expression levels in the Mi-treated plants than in the controls from 3 to 28 DAI (Fig. 2B).

**Figure 3:**
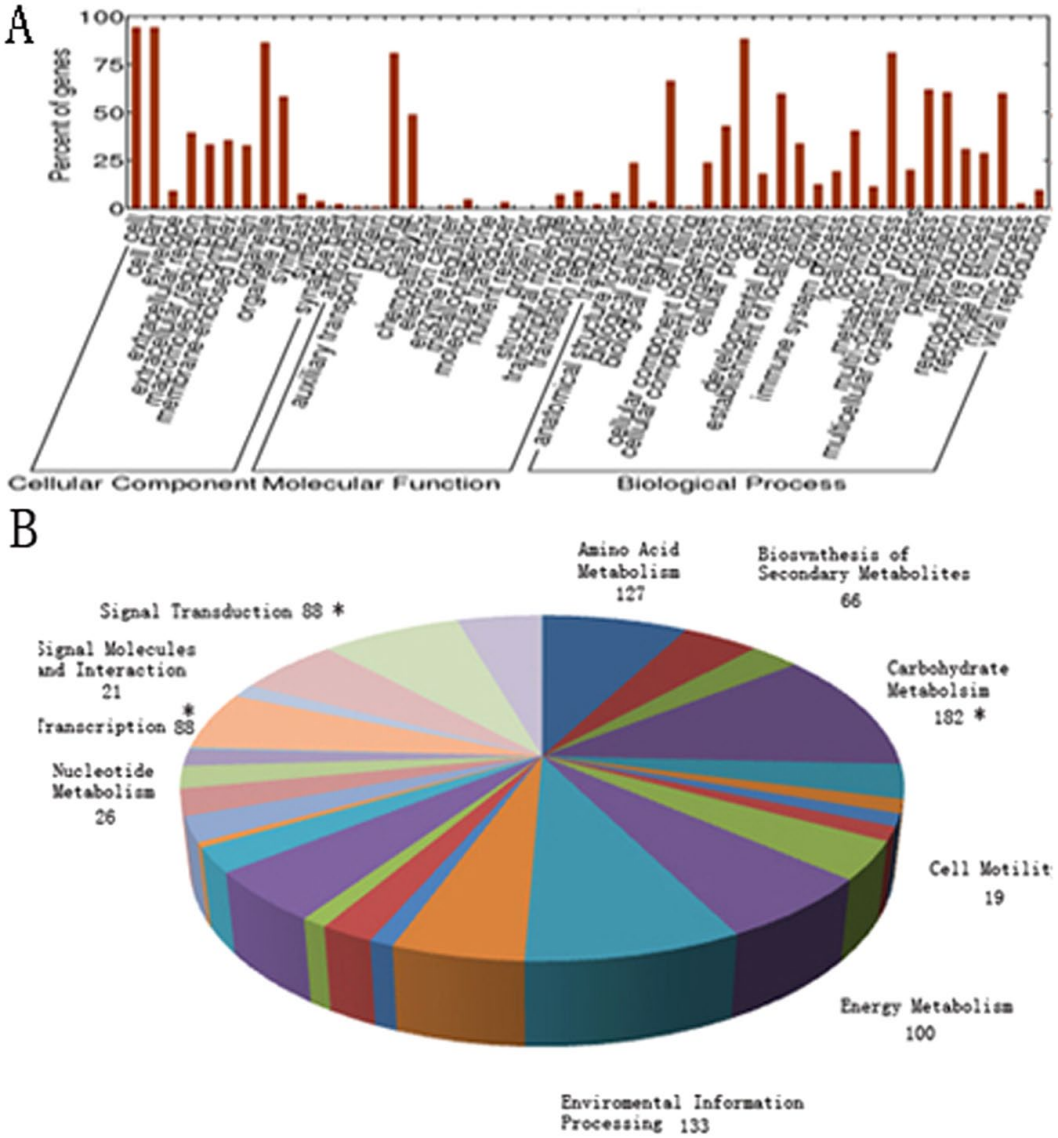
The GO, KEGG analysis of DEUs. (**A**) The GO classification of DEUs. The x-axis indicates the different subcategories, and the y-axis indicates the percent of genes in a subcategory. (**B**) The main KEGG pathways of DEUs. Different colors represent the pathway entries and pathway names, and the number represent the gene numbers in the pathway. The “*” represent the enriched subcategories.

**Figure 4:**
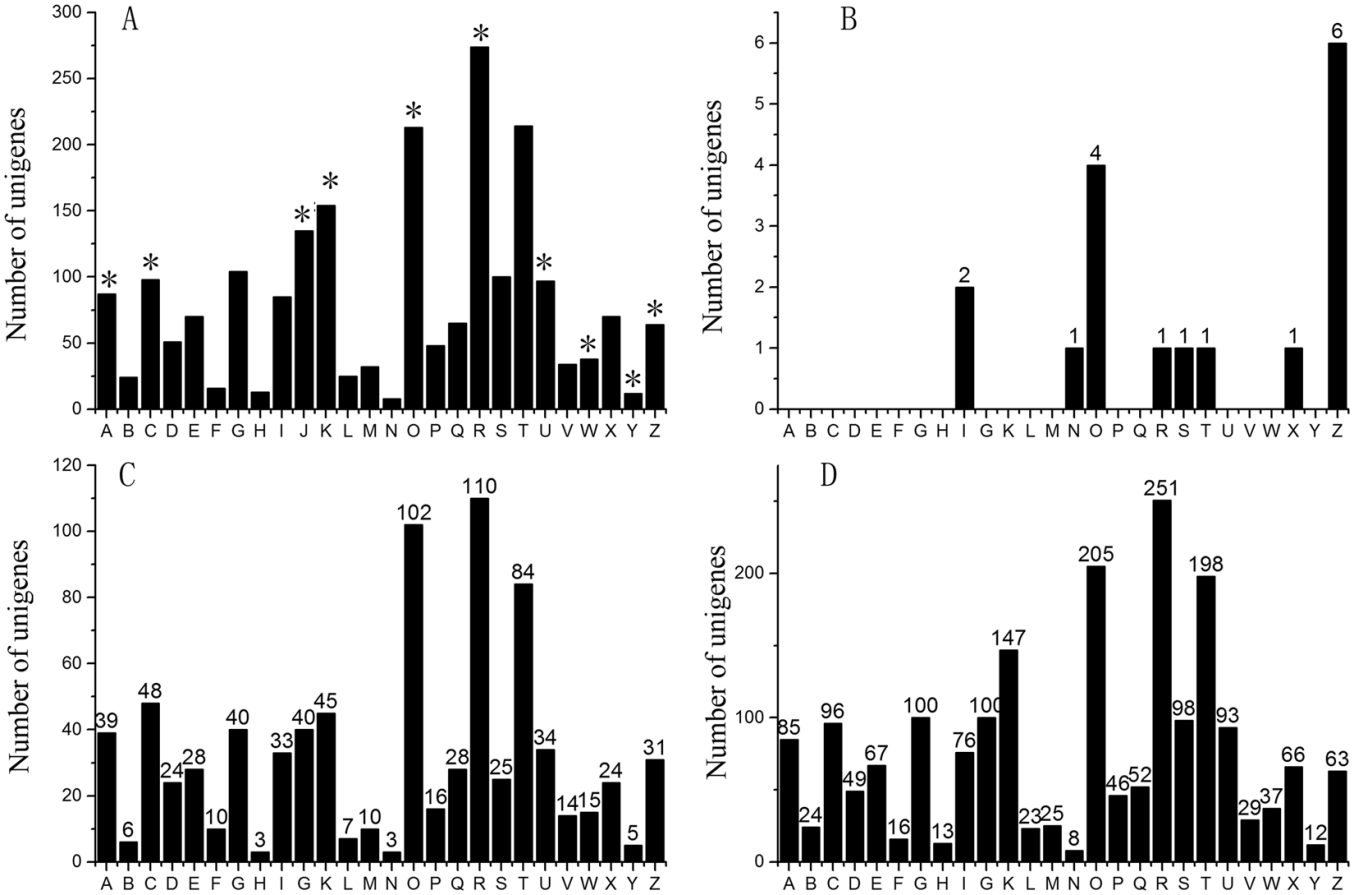
The KOG analysis of DEUs of 3d, 14d and 28d treatments. The x-axis indicates the different subcategories of KOG analysis, and the upper cases of x-axis are the abbreviations of their corresponding subcategories, which are showed in Supplementary Table S2. Y-axis indicates the gene number of each subcategories. The numbers on each column represent the gene number of the subcategories. (**A**) The KOG analysis of all DEUs. The “*” represent the enriched subcategories. (**B**) The KOG analysis for the DEUs of 3d treatment. (**C**) The KOG analysis for the DEUs of 14d treatment. (**D**) The KOG analysis for the DEUs of 28d treatment.

Functional annotation of the DEUs was performed by searching GO, KEGG and KOG database (Supplementary Table S2). The functional classifications were assigned using GO terms (Fig. 3A). The pathways were defined based on the KEGG database and the results showed that the DEUs were enriched in cell wall of carbohydrate metabolism pathway, plant hormone signal transduction and transcriptions (Fig. 3B). Additionally, the KOG analysis of the DEUs at different time points revealed that during the early stage of an Mi infection (3 DAI), the most abundant DEUs were related to the cytoskeleton (6/16) (Fig. 4B). This indicated that genes encoding components of the cytoskeleton may be involved in the early responses of *Cm* to *Mi*. During the middle (14 DAI) and late (28 DAI) stages of an *Mi* infection, the DEUs associated with signal transduction, post-translational modification, and transcription were the most abundant, implying that these processes may be important for *Cm* responses to *Mi* in these stages (Fig. 4C,D).

### Analysis of *Mi*-responsive hormone-relative genes

**Figure 5:**
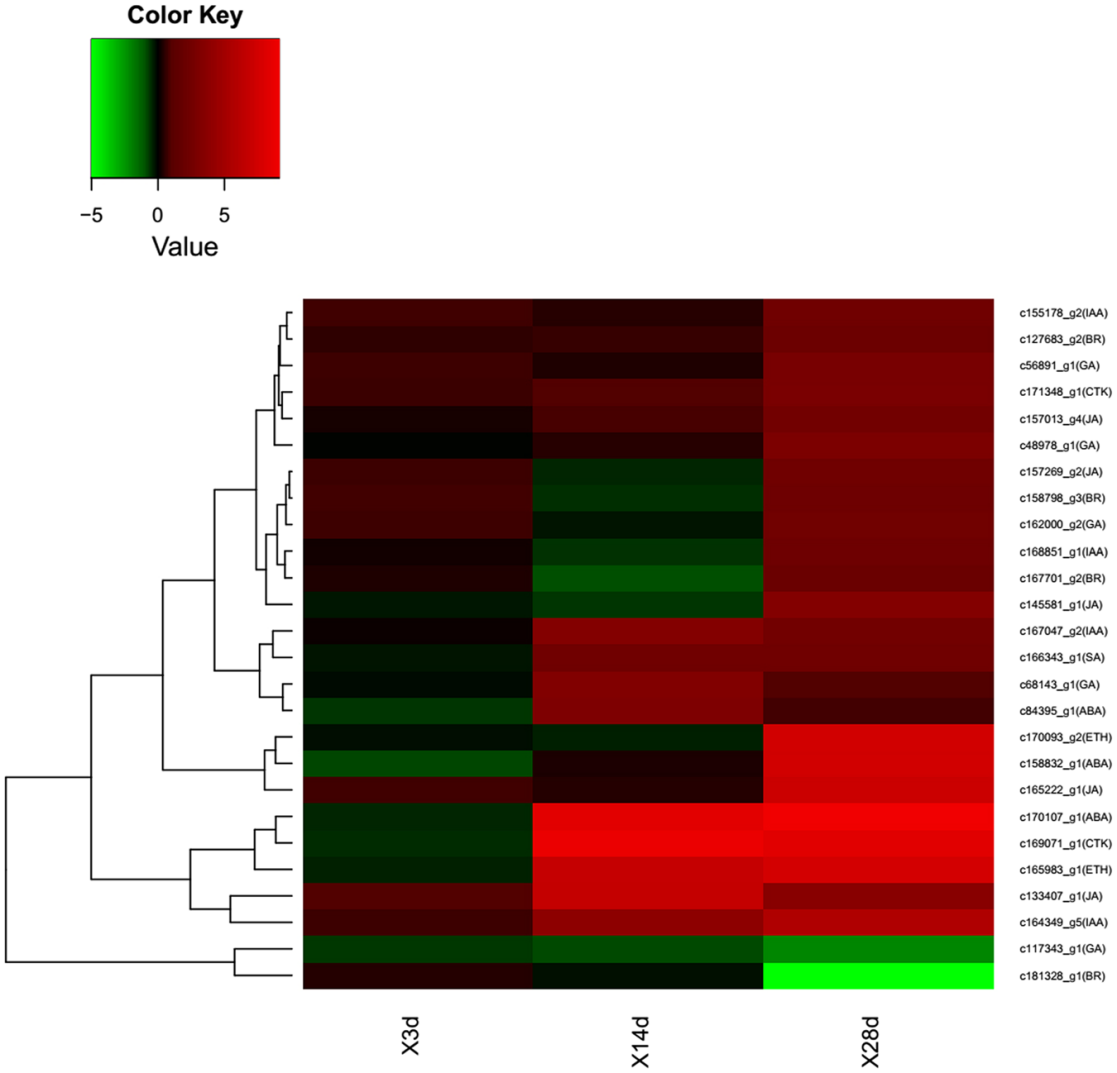
The expression levels of 26 hormone biosynthesis in 3d, 14d and 28d treatment. The RPKM values of hormone biosynthesis genes after infected by *Mi* were used for hierarchical cluster analysis with the R heatmap package. Details of annotated unigenes shown on the right are provided in Supplementary Fig. S3.

Plant hormones have been shown to be widely involved in the host plant response to RKN infection. In this study, we identified 26 DEUs associated with the biosynthesis of plant hormones, including ethylene (ET), abscisic acid (ABA), auxin, jasmonic acid (JA), salicylic acid (SA), gibberellic acid (GA), cytokinin, and brassinosteroid (BR). None of the 26 genes were differentially expressed between the control and Mi-infected plants at 3 DAI, while nine and 24 were differentially expressed at 14 and 28 DAI, respectively (Fig. 5 and Table 3). Most of the tested DEUs (24/26) were up-regulated response to the *Mi-*infection. The exceptions were *c181328_g1 (SAG29)* and *c117343_g1 (GA5)*, a BR and a GA biosynthesis gene respectively, which were down-regulated at 28d treatment. To validate the RNA-seq data, we analyzed the expression of five hormone biosynthesis genes by quantitative RT-PCR (qRT-PCR), including *DXPS2 (c170107)* for ABA biosynthesis, *NGA1 (c164349_g5)* for auxin, *SAG29 (c181328_g1)* for BA, *IPT4 (c169071_g1)* for CK and *GA5 (c117343_g1)* for GA. Our data indicated that the expression levels of three (DXPS2, NGA1, and IPT4) and two (SAG29 and GA5) genes were up- and down-regulated, respectively, in response to Mi. These observations were consistent with the results of the RNA-seq analysis (Supplementary Fig. S3).

**Table 3:** The differentially expressed hormone biosynthesis genes.

Geneid	Orthologs	e-value	Hormone	Pathway^#^	3dt^#^	14dt	28dt
c158832_g1	AT1G16540	1.00E-25	abscisic acid	HB	—	—	up
c84395_g1	AT1G52340	5.00E-82	abscisic acid	HB	—	up	—
c170107_g1	AT4G15560	5.00E-23	abscisic acid	HB	—	up	up
c155178_g2	AT5G11320	2.00E-25	auxin	HB	—	—	up
c167047_g2	AT4G31500	1.00E-107	auxin	HB	—	up	up
c168851_g1	AT4G39950	1.00E-153	auxin	HB	—	—	up
c164349_g5	AT2G46870	5.00E-68	auxin	HB	—	up	up
c158798_g3	AT3G50660	0	brassinosteroid	HB	—	—	up
c167701_g2	AT3G13730	0	brassinosteroid	HB	—	—	up
c127683_g2	AT1G20330	3.00E-22	brassinosteroid	HB	—	—	up
c181328_g1	AT5G13170	4.00E-86	brassinosteroid	HB	—	—	down
c169071_g1	AT4G24650	5.00E-25	cytokinin	HB	—	up	up
c171348_g1	AT3G19160	3.00E-74	cytokinin	HB	—	—	up
c165983_g1	AT2G43790	3.00E-29	ethylene	HB	—	up	up
c170093_g2	AT5G57740	8.00E-22	ethylene	HB	—	—	up
c56891_g1	AT4G21690	2.00E-43	gibberellin	HB	—	—	up
c68143_g1	AT1G78440	1.00E-108	gibberellin	HB	—	up	—
c117343_g1	AT4G25420	2.00E-153	gibberellin	HB	—	—	down
c162000_g2	AT4G08150	9.00E-38	gibberellin	HB	—	—	up
c48978_g1	AT1G65620	6.00E-21	gibberellin	HB	—	—	up
c157013_g4	AT5G42650	0	jasmonic acid	HB	—	—	up
c145581_g1	AT2G43710	4.00E-176	jasmonic acid	HB	—	—	up
c157269_g2	AT3G45140	0	jasmonic acid	HB	—	—	up
c165222_g1	AT4G29010	4.00E-71	jasmonic acid	HB	—	—	up
c133407_g1	AT1G19640	6.00E-78	jasmonic acid	HB	—	up	up
c166343_g1	AT1G74710	2.00E-21	salicylic acid	HB	—	up	up

^#^HB: hormone biosynthesis. ^#^3dt: 3d treatment. :-Not differentially expressed genes. Up: up-regulated. Down: down-regulated.

In addition to the DEUs associated with plant hormone biosynthesis, we also identified 139 DEUs associated with other plant hormone-related genes, including hormone response, hormone signal receptor, hormone signal transduction and hormone transportation. The expression patterns of these genes are presented in Supplementary Table S3. Similar to results for the hormone biosynthesis genes, the expression levels of most of the other hormone-related genes were up-regulated (128/139), with the highest expression levels at 28 DAI (98/139).

In addition to genes related to hormone pathways, we also detected many DEUs associated with pathways downstream of plant hormones (Supplementary Table S4). For example, we identified six ethylene-responsive factor (ERF) transcription factor (TF) genes, whose expression levels are affected by ET signals to regulate the expression of downstream genes. We also detected two ABA-responsive SNRK2-encoding genes that influence the expression of downstream genes via phosphorylations. Two auxin response factor (ARF) genes were identified, which influence the downstream auxin-regulated gene expression.

### Analysis of nematode-responsive transcription factors

**Figure 6:**
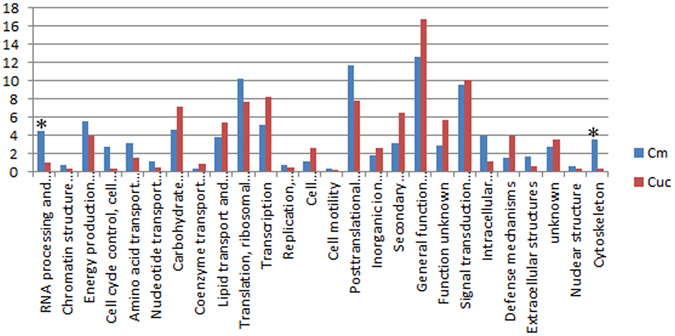
The category of differentially expressed transcriptional factors. (**A**) The number of differential type transcriptional factors. Y-axis represents the number of TF and x-axis represent the type of TFs. (**B**) The expression pattern of WRKY genes. Red indicate up-regulated and black indicate no significant difference. (**C**) The phylogenetic tree of 14 differentially expressed WRKY genes. The tree was constructed using the neighbor-joining method as implemented in PHYLIP 3.2. Numbers on internal nodes are the percentage bootstrap support values (1000 re-sampling).

Transcription factors play key roles in modulating plant adaptations to biotic stresses. In this study, we searched the Plant Transcription Factor Database for matches for the 2,430 DEUs, and identified 337 DEUs as candidate TF-encoding genes. Of these, 207 DEUs were categorized into 33 common plant TF families (Supplementary Table S5). Among the 207 DEUs, MYB TFs were the most abundant (19), followed by NAC (18), ERF (18), bHLH (16), and WRKY (14) TFs (Fig. 6A). Most of differentially expressed TFs (DETFs) were up-regulated by the *Mi* infection (186/207). The down-regulated DETFs included some important TFs such as MYB (3), SBP (2), GRAS (2), and C2H2 (2). We detected only two DETFs at 3 DAI, namely a *WRKY* gene (c170216_g2) and an *E2F* gene (*c165727_g1*). Of the 207 DETFs, 81 were detected at 14 DAI, with all but one of them being up-regulated. Additionally, 188 DETFs were detected at 28 DAI, with 168 being up-regulated and 20 being down-regulated. An analysis of the expression levels of the 207 DETFs revealed that 18 DETFs were most highly expressed at 3 DAI, while 41 and 148 were most highly expressed at 14 DAI and 28 DAI, respectively.

The *WRKY* genes form a large family of transcriptional regulators in higher plants, and affect many biological processes, such as responses to biotic and abiotic stresses, development, and metabolism^24^. In this study, the 14 *WRKY* genes identified among the DETFs belong to different subgroups, specifically groups 1, 2b, 2c, and 3 (Fig. 6C). All WRKY genes were up-regulated at one of the treatment time points (Fig. 6B). For example, the expression of *c170216_g2* (*WRKY23*) was up-regulated at 3 DAI, indicating *WRKY23* influences an early response to a *Mi* infection. Additionally, the expression levels of eight and 12 *WRKY* genes were up-regulated at 14 and 28 DAI, respectively. The group 3 *WRKY* genes are reportedly involved in plant host pathogen resistance. We identified five group 3 *WRKY* genes whose expression levels were up-regulated in response to *Mi*, suggesting they are important for the resistance of *Cm* to *Mi*. The qRT-PCR data (Supplementary Fig. S2) revealed that *WRKY33*, *WRKY40*, and *WRKY63* expression levels were up-regulated at 14 and 28 DAI.

### Comparisons of transcriptomes of *Cm* and cucumber response to *Mi* infections

Most Cucurbitaceae crops are sensitive to RKNs. The inbred cucumber line 9930, which is widely cultivated in northern China, has been considerably affected by RKNs. In this study, we compared the transcriptome data of *Mi*-infected 9930 plants with the data for infected *Cm* at 14 DAI. We identified 561 differentially expressed cucumber genes (cucDEGs), with 208 and 353 being down- and up-regulated, respectively (Supplementary Table S6). The cucDEGs were functionally annotated using the GO, KOG, and KEGG databases. Several cucDEGs were associated with plant hormones, TFs, and pathogens, similar to the differentially expressed Cm genes. For example, 87 cucDEGs were identified as plant hormone-related genes, including 14 hormone biosynthesis genes associated with the ET, ABA, SA, or JA biosynthesis pathways (Supplementary Table S6).

**Figure 7:**
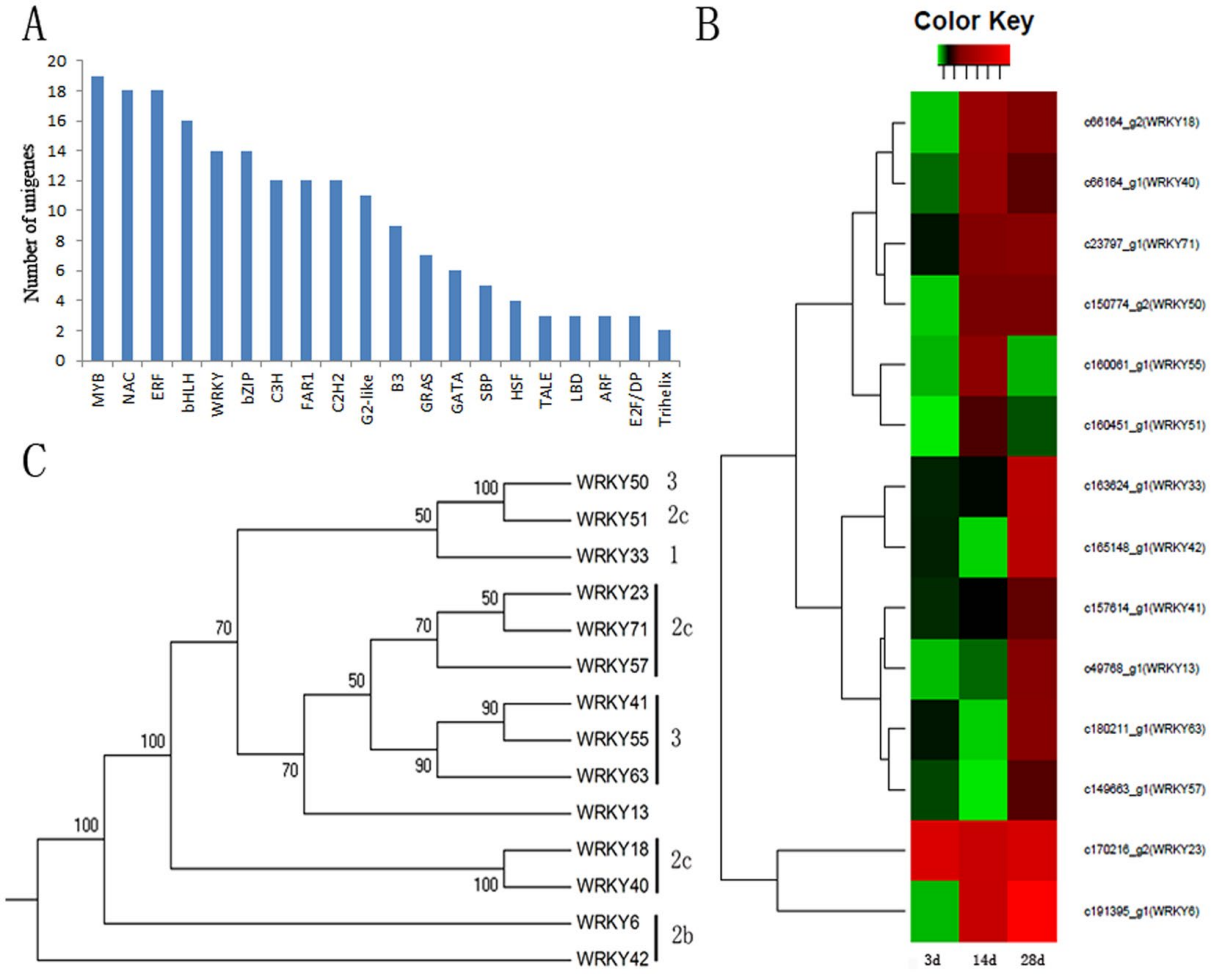
Comparison of DEGs between Cm and cucumber after 14 RKN treatment. The KOG analysis and comparison between cucumber and *Cm*. The x-axis indicates clusters arranged by KOG. The y-axis indicates the number of the KOG clusters. Blue columns represent *Cm* and red columns represent cucumber. *Indicate very significant difference between cucumber and *Cm* (p < 0.001).

We compared the genes that were differentially expressed between Cm and 9930 plants at 14 DAI based on a KOG analysis (Fig. 7). We observed significant differences in two gene clusters (i.e., cytoskeleton and RNA processing), suggesting that these genes may be associated with the resistance of Cm to *Mi*.

### Analysis of nematode-responsive cytoskeleton-related genes

**Figure 8:**
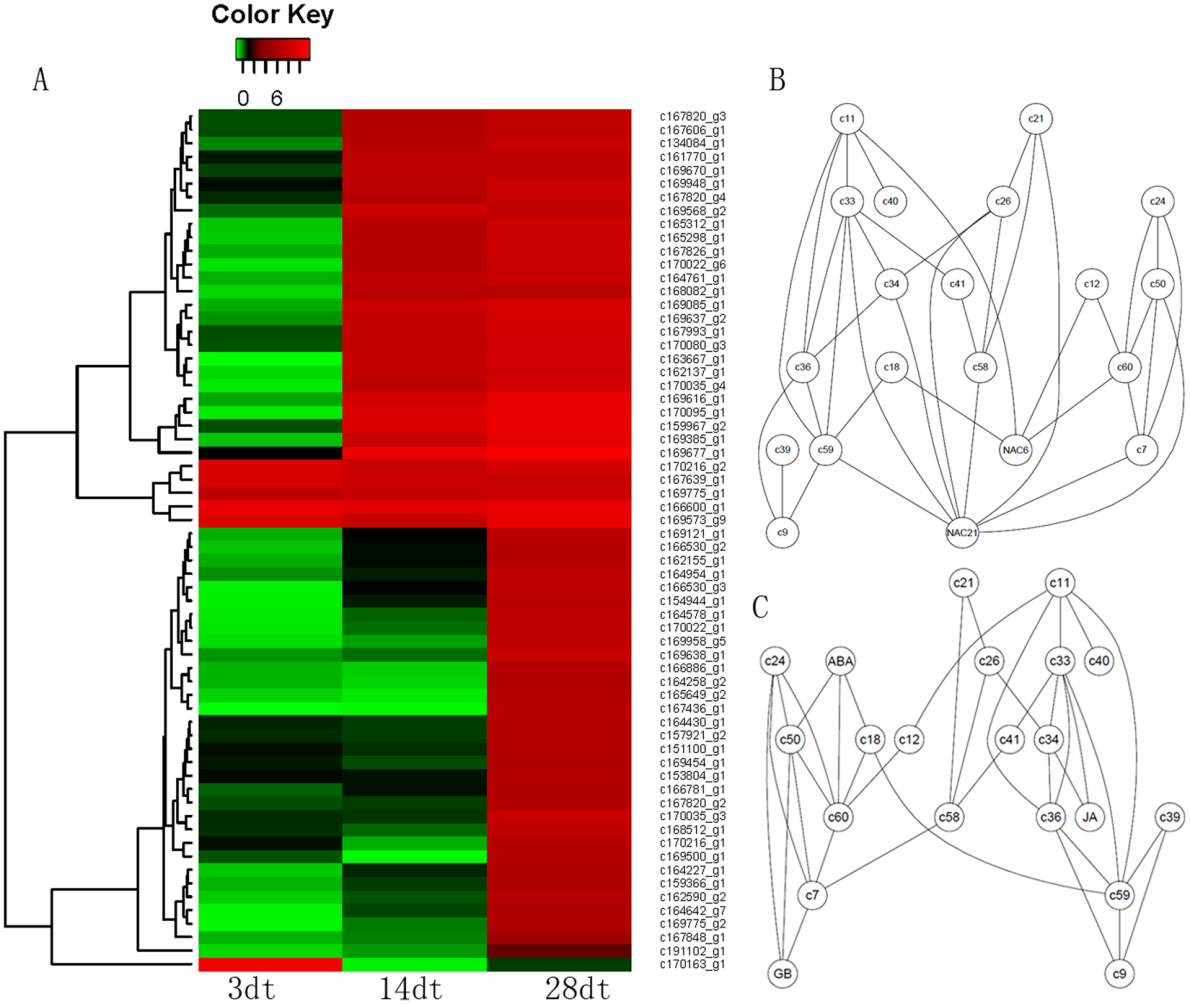
The expression pattern and co-expression network of cytoskeleton-related DEUs. (**A**) The log2 Ratio values of *Cm* cytoskeleton related DEUs were used for hierarchical cluster analysis with the R heatmap package. Red represents up-regulated unigenes, green represents down-regulated unigenes, and black indicates no significant difference in unigene expression. Details of annotated unigenes shown on the right are provided in Supplementary Table S7. (**B**) The co-expression net containing 18 cytoskeleton-related genes and two NAC transcriptional factors; the gene names, such as *c9* and *c39*, were corresponding to cytoskeleton related genes and were listed in Supplementary Table S7. (**C**) The co-expression net containing 17 cytoskeleton-related genes and three hormone biosynthesis genes.

Our comparison of the *Cm* and cucumber transcriptomes suggested that *Cm* cytoskeleton genes may be involved in the interactions between *Cm* and *Mi*, with important consequences for the resistance of *Cm* to *Mi*. A total of 64 cytoskeleton-related DEUs were detected, including six at 3 DAI, 31 at 14 DAI, and 63 at 28 DAI (Fig. 8A). Only 51 DEUs were detected at 3 DAI, and the six cytoskeleton-related genes were likely involved in early responses to invading Mi nematodes. All of the DEUs were up-regulated in response to Mi (Fig. 8A). Five genes (*c169775_g1, c167639_g1, c169573_g9, c170216_g2, and c166600_g1*) were constitutively differentially expressed at all three time points (Supplementary Table S7). Based on analyses of the correlations among the DEU expression levels, two co-expression networks were constructed for the cytoskeleton-related genes. One network contained two NAC domain-containing TFs (*c172779_g1 and c170025_g1*) and 18 cytoskeleton-related genes (Fig. 8B), including two myosins and four tubulins. Tubulins are a major component of eukaryotic microtubules, while myosins are responsible for microtubule-based motility, which is important for plant defence responses. Our results suggested that NAC TFs may regulate cell wall remodelling following Mi infections. The other network comprised 17 cytoskeleton-related genes and hormone biosynthesis genes for ABA (*c170107_g1*), GA (*c162000_g1*), and JA (*c165222*) (Fig. 8C). This implied that hormone signals help regulate the expression of cytoskeleton-related genes.

In addition to the cytoskeleton-related genes, two DEUs were identified as encoding the actin depolymerizing factor (ADF), which is important for actin formation. We observed similarities between c164561_g1 and Arabidopsis thaliana ADF8, as well as between *c166034_g2* and ADF5. The qRT-PCR results revealed that the ADF-encoding genes were differentially expressed at all time points. Additionally, their highest relative expression levels occurred at 28 DAI (Supplementary Fig. S3), suggesting they are important for responses to Mi infections.

### Analysis of *Cm* pathogen-related genes and nematode effectors

**Figure 9:**
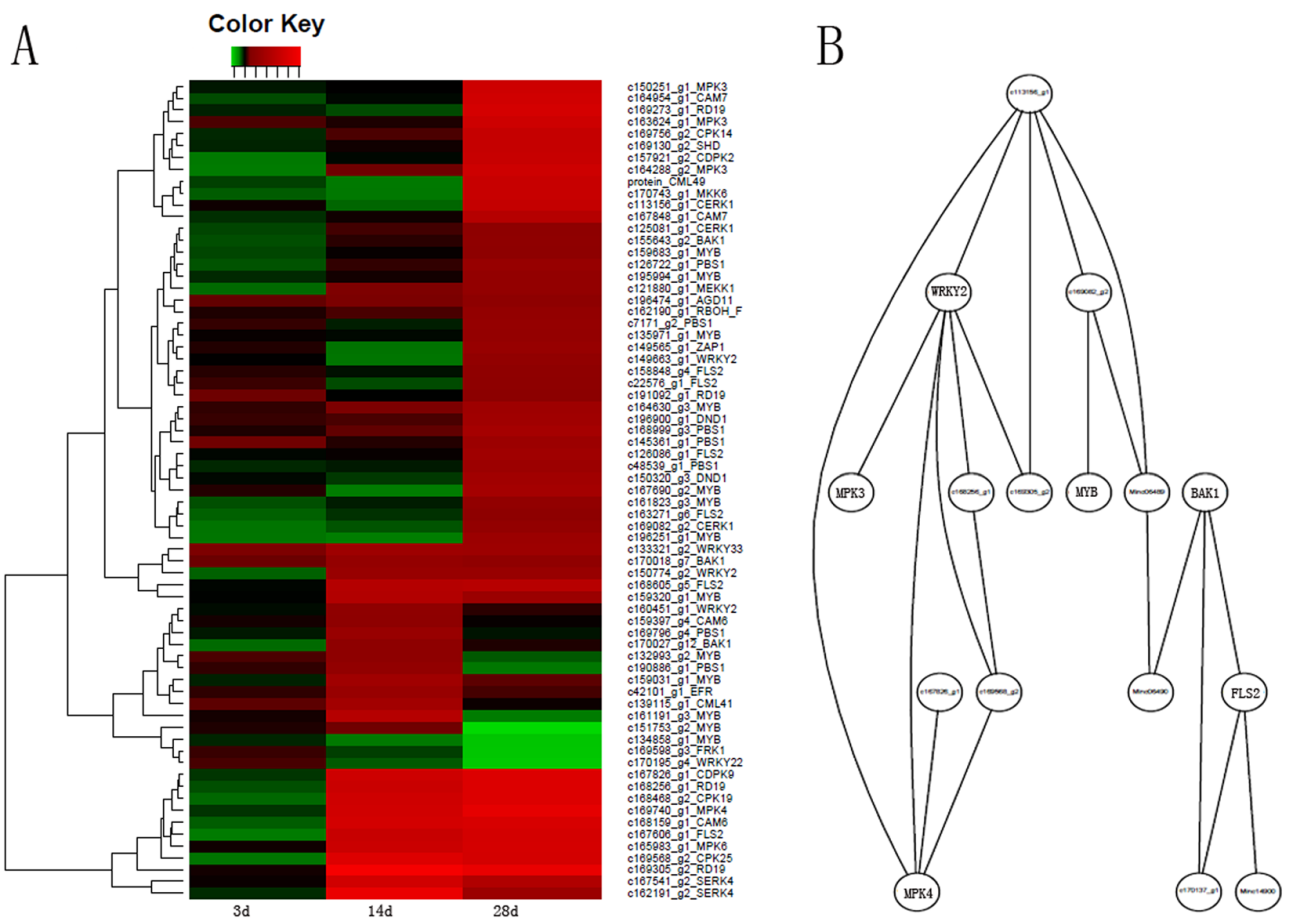
The expression pattern of Cm pathogen-related genes (**A**) and the co-expression of pathogen-related genes and RKN effectors. (**A**) The log2 Ratio values of *Cm* pathogen related DEUs were used for hierarchical cluster analysis with the R heatmap package. Red represents up-regulated unigenes, green represents down-regulated unigenes, and black indicates no significant difference in unigene expression. Details of annotated unigenes shown on the right are provided in Supplementary Table S8. (**B**) The co-expression net of *Cm* pathogen related DEUs and *Mi* effectors. The net contains *Cm* 13 pathogen related genes and three known RKN effectors (*Minc06489*, *Min06490* and *Minc14900*).

We identified a total of 71 DEUs associated with plant pathogen-related genes in all three time points (Supplementary Table S8). It was noted that none of the DEUs were detected at 3d treatment. 28 out of 71 DEUs were detected at 14 d treatment, with 10 DEUs specific to 14d treatment. 57 DEUs were detected at 28 d treatment, with 39 genes were specific to 28d treatment. Only four DEUs were down-regulated genes and all of they were down-regulated at 28d treatment, including *WRKY22*, *FRK1* and two MYB genes (Fig. 9A). Some plant pathogen-related DEUs were transcriptional factors, including four WRKY genes (*WRKY1*, *WRKY2*, *WRKY22* and *WRKY33*) and 13 MYB genes. Some were associated with pathogen-related signal transduction pathway, including MPK, MKK and Calcium ion associated protein genes (CAM). It was noted that some key genes involving PAMPs (pathogen-associated molecular patterns) were also identified, including *FLS2*, *BAK1*, *CDPK9* and SERK4, suggesting that the basic defense response were inspired in *Cm* response to *Mi* infection. The expression of *SERK4*, *FLS2* and *BAK1* were validated by qRT-PCR (Supplementary Fig. S3), which verified them as up-regulated genes after *Mi* treatment.

To identify putative *Mi* genes, we mapped all the *Cm* RNA-seq reads to the *Mi* genome. As a result, we identified a total of 6043 *Mi* genes whose expressions were detected only in the transcriptomes of the *Mi*-infected *Cm* roots, and we considered these genes as putative *Mi* genes (Supplementary Table S9). Of the 6043 genes, 247 were identified as encoding secreted proteins. We compared the sequences of these proteins with all known and annotated secreted proteins from plant parasitic nematodes. A total of 31 homologs were identified, 18 of which were reported previously as putative effectors from the esophageal gland cells of *Mi* ^25^ and, among the remaining 13, some were cell wall enzymes, including peroxidases, glutathione S-transferases, and xylanases, suggesting the modification of host cell wall by *Mi* effectors. The expression patterns of the 31 genes encoding putative effectors in the different libraries were analyzed. Two genes encoding putative effectors *Minc03287* and *Minc19171* were expressed only in the 3d treatment libraries, suggesting they may play roles in the early stage of nematode infection. The expressions of 28 genes were detected in 14d treatment. In 28d treatment, 28 gene expressions were detected, in which *Minc08210* was specifically expressed in 28d treatment. The highest expression levels were detected for the gene encoding *Minc08986*, a fatty acid and retinol-binding protein. The *Minc08986*-encoding gene was highly expressed in all the treatment libraries and its expression levels peak in the 28d treatment libraries, suggesting that *Minc08986* may play important roles in nematode infections.

The co-expression net of *Cm* pathogen related DEUs and *Mi* effectors were analyzed (Fig. 9B). The net contains 13 *Cm* pathogen related genes and three known *Mi* effectors (*Minc06489*, *Min06490* and *Minc14900*). The co-expression net included two transcriptional factors (*WRKY2* and MYB), PAMPs-associated genes (*BAK1*, *FLS2* and so on) and pathogen-related signal genes (*MPK3* and *MPK4*), suggesting a complicated gene interactions between *Mi* and the host.

## Discussion

### Plant hormone plays an important role in the *Mi* infection

Plant hormones are known to play important roles in the regulation of plant immune responses to various biostresses. Hormone signaling pathways form a complex interconnected regulation network through which plants can rapidly adapt to biostresses in the environment^26^. Because of their important roles, the number of known hormone-related genes in Arabidopsis has been reported as 617^27^, which accounts for about 2.3% of total number of annotated genes in the Arabidopsis genome. In this study, we found that 595 of the 617 Arabidopsis hormones-related genes (87%) had homologs among the 138,390 assembled unigenes from the *Cm* transcriptome data, indicating that most of the known hormone-related genes were detected in our transcriptome analysis. Some DEUs were identified as plant hormone-biosynthesis genes, involving in ET, ABA, auxin, JA, SA, CK and BR pathway. For example, two of the hormone-related DEUs were predicted to be involved in auxin biosynthesis, and they both encoded proteins that belonged to the cytochrome P450 family. *c167047_g2* was found to be a homolog of *AT4G31500*, which encodes an oxime-metabolizing enzyme in the biosynthetic pathway of glucosinolates^28^, and *c168851_g1* was predicted to encode a cytochrome P450 involved in tryptophan metabolism^28^. A DEU associated with CK biosynthesis, *c171348_g1*, was found to be a homolog of *AT3G19160*, which encodes *IPT8*, which catalyzes the formation of the CK iPMP from DMAPP and AMP^29^. These results indicated that many hormone biosynthesis-related genes were involved in the resistance response of *Cm* to *Mi*.

Some of the hormone-related DEUs appeared to form integrity pathways for plant hormone biosynthesis. For example, two DEUs associated with the ABA biosynthesis pathway, *c158832_g1* and *c84395_g1*, were identified. *c158832_g1* was found to be a homolog of *AT1G16540*, which encodes molybdenum cofactor sulfurase, which catalyzes the conversion of ABA-aldehyde to ABA in the last step of ABA biosynthesis^30^. C84395_g1 was found to be a homolog of *AT1G52340*, which encodes ABA2, a cytosolic short-chain dehydrogenase/reductase involved in the conversion of xanthoxin to ABA-aldehyde in the penultimate step of ABA biosynthesis^30^. Besides these ABA biosynthesis DEUs, 28 DEUs associated with ABA signal transduction and ABA receptors were identified. Down-stream DEUs in the ABA biosynthesis pathway were also identified. The regulation of ABA is by through snrk gene family, which can phosphorylate AREB genes and then regulate downstream gene expression^31^. In Arabidopsis, there are 16 snrk genes. In this study, we identified a total of 24 homologs of snrk genes in *Cm* transcriptome data, and two of they are DEUs. We also identified an AREB gene in DEUs. *C186097_g1* is a homolog of AREB1 of Arabidopsis, which can regulate down-stream genes who have ABA-response element in their promoter region^32^.

### The various functions of RKN effectors in the RKN infection

Plants have evolved a two-layered system to defend themselves against pathogen invasions, namely pathogen-associated molecular pattern (PAMP)-triggered immunity (PTI) and effector-triggered immunity (ETI). Because PTI serves as the basal defence system in plants, it is not surprising that pathogens secrete so-called effectors that target the genes involved in PTI. For example, in *A. thaliana*, AvrPtoA and AvrPtoB secreted by Pseudomonas syringae directly target the signalling partner *BAK1*, which is involved in *PTI* ^33^. Therefore, identifying the effectors secreted by RKNs is crucial for elucidating the molecular basis underlying RKN parasitism of plants. Huang *et al*. reported that 185 putative effectors are expressed in RKN oesophageal gland cells throughout the parasitic cycle, most of which have unknown functions^34^. In this study, we identified 247 RKN transcripts encoding small secreted proteins in the transcriptomes of RKN-infected *Cm* plants, including 31 transcripts encoding known nematode effectors. Some effector functions are known. For example, *Minc08210* encodes a substrate-adhesion molecule, while *Minc13373* is associated with a lysosomal acid phosphatase^34^. Additionally, *Minc08986* encodes a fatty acid- and retinol-binding protein that can modify plant tissues and defense responses^35^. *Minc11599* was annotated as a glutathione S-transferase thought to prevent the oxidative burst generated in response to nematode infections. Furthermore, we identified 71 pathogenesis-related genes (e.g., *FLS2*, *BAK1*, *CDPK9*, and *SERK4*), as well as some keys genes affecting PAMP and pathogen-related signal transduction pathways (e.g., MPK, MKK, and calcium ion pathways). Based on gene expression patterns, we constructed a co-expression network that includes three RKN effector genes (*Minc06489*, *Min06490*, and *Minc14900*), two PAMP-associated genes (*BAK1* and *FLS2*), and two pathogen-related signalling genes (*MPK3* and *MPK4*). The *BAK1* and *FLS2* genes play important roles in plant immune responses, thus making them pathogen effector targets. The *BAK1* gene encodes a receptor-like protein kinase, which functions as a co-receptor with *FLS2* to mediate PTI^36^. The AvrPtoB effector secreted by P. syringae can suppress plant host PTI by binding to *BAK1* ^37^, while AvrPto binds to FLS2 to block plant immune responses^38^. Therefore, we speculate that the RKN effectors interact with BAK1 and FLS2 during invasions.

### The dual nature of Cytoskeleton-related genes: the susceptible and resistant reactions of host to RKN

**Figure 10:**
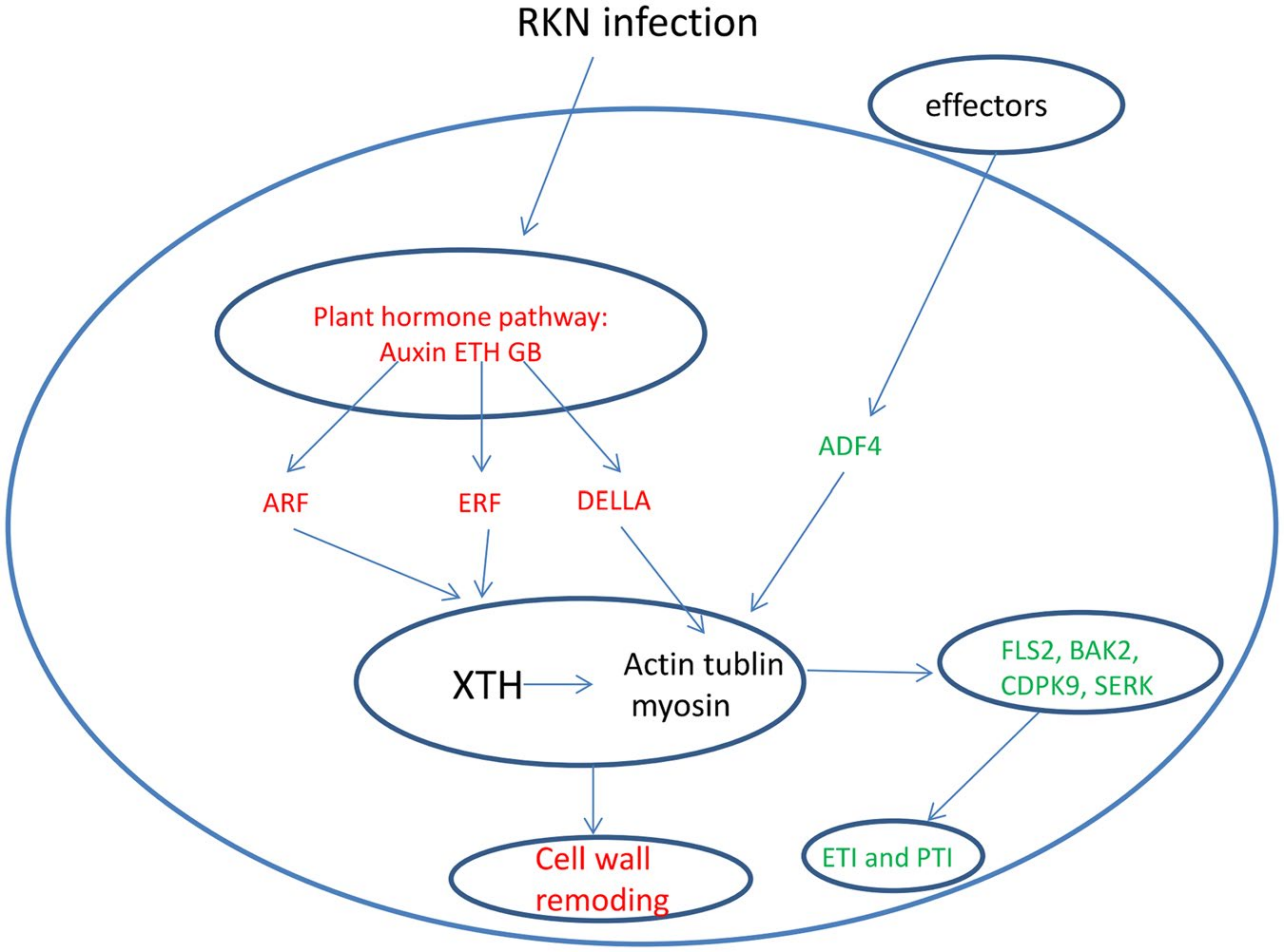
Overview of the roles of cytoskeleton related genes under RKN infection in Cm. A sketch for putative roles of cytoskeleton related genes in compatible and incompatible reaction of *Cm* to *M*i. Red represents compatible reaction and green represent incompatible reaction.

The formation of giant cells is the key step of RKN invasions^3^, with cytoskeleton remodelling mediated by dozens of genes representing an important process^39^. Some studies concluded that plant hormones are the key regulators of cytoskeleton-related genes. For example, GA can induce the degradation of DELLA proteins by the proteasome^40^. Five *A. thaliana* DELLA proteins interact with cytoskeleton-related prefoldin, which is an essential part of the chaperone machinery facilitating the assembly of active α/β-tubulin dimers^41^. Ethylene can regulate the ERF and consequently inactivate the XTH. The *XTH* gene family is associated with the formation of plant cells. Root-knot nematode invasions alter the expression of *XTH* genes as well as their target genes that regulate myosin, tubulin, and lignin contents^6^. The ARFs bind to the promoter of auxin-responsive genes to regulate expression. The ARF targets include cytoskeleton genes, such as *expansi*n, *XTH*, and *yieldin*, indicating that auxin can regulate the expression of cytoskeleton-related genes. We detected 26 DEUs associated with hormone biosynthesis and 139 DEUs related to plant hormones. Therefore, we hypothesize that the hormone levels of *Cm* plants are influenced by an RKN invasion, ultimately leading to the regulation of cytoskeleton-related genes and increased susceptibility of plant hosts to RKNs (Fig. 10).

In contrast, the cytoskeleton is also an important component of the PTI and ETI plant defence mechanisms against pathogenic organisms^41^. In A. thaliana, the ADF increases the density of actin filaments, which are required by the PTI components *BIK1* (Botrytis-induced kinase 1) and BAK1^42^. In this study, we identified two ADF-encoding DEUs that were significantly differentially expressed at all three time points. We also observed that BAK1 was differentially expressed between control and *Mi-*infected plants. Our data suggest that *BAK1* and *ADFs* may be important for the resistance of Cm to RKNs. Additionally, two MPK-encoding DEUs (*MPK3* and *MPK6*) were detected. In A. thaliana, ADF can induce the expression of *MPK3* and *MPK6*, which help establish PTI and ETI^43^. Therefore, our results suggest that ADF-related plant immune responses are responsible for the resistance of *Cm* to RKNs (Fig. 10).

## Materials and Methods

### Plant materials and *Mi* treatments

The resistance of horned melon (*Cucumis metuliferus*) and the inbred cucumber line 9930, widely planted in northern China, were used in this study. The plant seedlings were grown in greenhouse. J2 juveniles of *Mi* freshly hatched from egg masses were used as inoculate. After 14 days of sowing, single plants were transplanted into 15 × 15 *Cm* (diameter/high) plastic pots, which contain a pasteurized mix of sandy loam soil and fine washed river sand (2:1 by volume), the pots were placed in growth chambers programmed to maintain a temperature of 22 °C and 16 h/8 h (light/dark) cycles^44^. Ten days after transplant, each seedling was inoculated with 500 J2 of *Mi*. Control was mock-inoculated with water. Plant root galls were harvested 1, 2, 3, 5, 7, 14, 28 and 42 day after inoculation (DAI). The roots of 3, 14, and 28 DAI were used to RNA extraction for RNA-seq, with two independent biological replicates of each. All treated roots were further stained with acid fuchsin for observing the development of *Mi* and estimating gall size and nematode body width, with at least three biological replicates for each treatment time points. Statistical analysis was carried out using R programming language.

### RNA extraction and qRT-PCR

Total RNA was isolated from *Cm* root samples treated with either *Mi* or water (as a control) for 3d, 14d and 28d using the Trizol reagent (Invitrogen, USA) according to the manufacturer’s instructions. The specific primers of target genes were designed using Primer5 software (Supplementary Table 10). For real-time PCR, we performed real-time RT-PCR using BIO-RAD CFX96 (Bio-Rad, USA). Amplification consisted of 40 cycles each as 30 s at 94 °C, 30 s at 55 or 60 °C, and 30 s at 72 °C. The experiments were repeated at least three times with independent RNA samples. Relative gene expression was calculated according to Ling *et al*.^44^. The *beta-actin* gene of *Cm* (ID: c168675_g9) was used as an internal control and the primers were listed as blow:

5′ -ATCCACGAAACTACTTACAACTCC -3′

5′ -ATAGACCCTCCAATCCAGACAC -3′

### Transcriptome sequencing and de novo assembly

Poly(A) mRNA was isolated from 10 μg total RNA of each *Cm* root sample. Each library had an insert size of approximately 300 bp and 100 bp read length, which was sequenced on a HiSeq 2000 system (Illumina, Berry Genomics, BeiJing). After filtering of low-quality raw reads, we used Tophat software to map transcriptome data on *Mi* genome, and the reads which showed perfect match to *Mi* genome were view as *Mi* transcriptome data. After filtering, transcriptome de novo assembly was carried out using Trinity software according to the manufacturer’s instructions^45^. The assembled transcript whose length is larger than 200 bp was kept. The longest transcript in each locus was taken as the unigene.

### Functional analysis of unigenes

To assign possible function annotations, all unigene sequences were searched using Blastx (e-value <10−5) against the following protein databases: PFAM (http://pfam.janelia.org/), KOG (http://www.ncbi.nlm.nih.gov/COG/), SWISSPROT (UniProt), NR (NCBI). The gene ontology (GO) were carried out using the Blast2GO program, and were categorized using WEGO software^46^. Kyoto Encyclopedia of Genes and Genomes (KEGG) annotations were obtained in http://www.kegg.jp/.

### Identification and functional analysis of DEUs

The DEUs were obtained using edgR method of trinity software by comparing *Mi* infected libraries with the corresponding control library for each time point. The transcripts were identified as DEUs based on FDR statistical method and changed folds (FDR < 0.001 and log2 Ratio| > 1). The co-expression of DEUs were obtained by R package RNASeqNet^47^. To obtain the function annotation of *Cm* DEUs, the DEUs sequences were search using Blastx against plant transcription factor database (plantTFDB v3.0)^48^ to identify transcription factors, against Arabidopsis hormone database (AHD2.0 v2.0) to identify plant hormone related genes, against Arabidopsis information resource(TAIR: http://www.arabidopsis.org/) to identify pathogen-related genes^27^.

### Transcriptome analysis of *Mi* and the identification of effectors

The Tophat and cufflink pipeline were used to analyze the transcripts of *Mi* by mapping RNA-seq data on published *Mi* genome^49, 50^ according to the manufacturer’s instructions. The numbers of reads of all 12 libraries were normalized to per kilo-base of exon model per million mapped reads (RPKM). SignalP 3.0 (http://www.cbs.dtu.dk/services/SignalP/) was used to predict potential secreted proteins and the transmembrane proteins were removed using the TMHMM software (http://www.cbs.dtu.dk/services/TMHMM/). The putative mitochondrial proteins were removed using TargetP (http://www.cbs.dtu.dk/services/TargetP/). The published nematode-related effectors were downloaded from NCBI. The predicted secreted proteins were search to the nematode-related effectors using blastp.

## Electronic supplementary material

supplementary file
