# Supplementary material for: Transcriptome profiling of Cucumis metuliferus infected by Meloidogyne incognita provides new insights into putative defense regulatory network in Cucurbitaceae
Source: Sci Rep. 2017 Jun 14;7:3544. doi: 10.1038/s41598-017-03563-6 (PMC5471208; doi:10.1038/s41598-017-03563-6)
Supplement: Supplementary file 1 — supplementary file [file 41598_2017_3563_MOESM1_ESM.pdf]

Transcriptome profiling of *Cucumis metuliferus* infected by *Meloidogyne incognita* provides new insights into putative defense regulatory network in Cucurbitaceae

Jian Ling#, Zhenchuang Mao#, *Mi*ngjuan Zhai, Feng Zeng, Yuhong Yang and Bingyan Xie*

Address: Institute of Vegetables and Flowers, Chinese Academy of Agricultural Sciences, 12 Zhongguancun South Street, Beijing, 100081 China

E-mail:

Jian Ling: lingjian@caas.cn;

Zhenchuan Mao: maozhenchuan@caas.cn

*Mi*ngjuan Zhai: 755409969@qq.com

Feng Zeng: zengfeng_1989@163.com

Yuhong Yang: yangyuhong@caas.cn

Bingyan Xie: xiebingyan@caas.cn

# These authors contributed equally to this work

* Corresponding author

Tel: +0086 010 82109545; Fax: +0086 010 62124615

Supplementary Figure S1

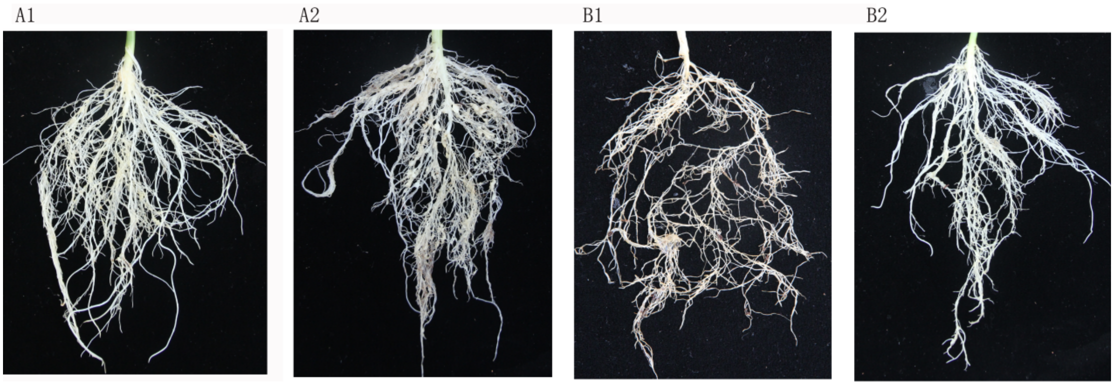

Supplementary Figure S1

Root phenotype of 9930 (A) and *Cm* (B) after 42 *Mi* treatment and their controls

A1: 9930 control; A2: 9930 infected with *Mi*

B1: *Cm* control; B2: *Cm* infected with *Mi*

Supplementary Figure S2

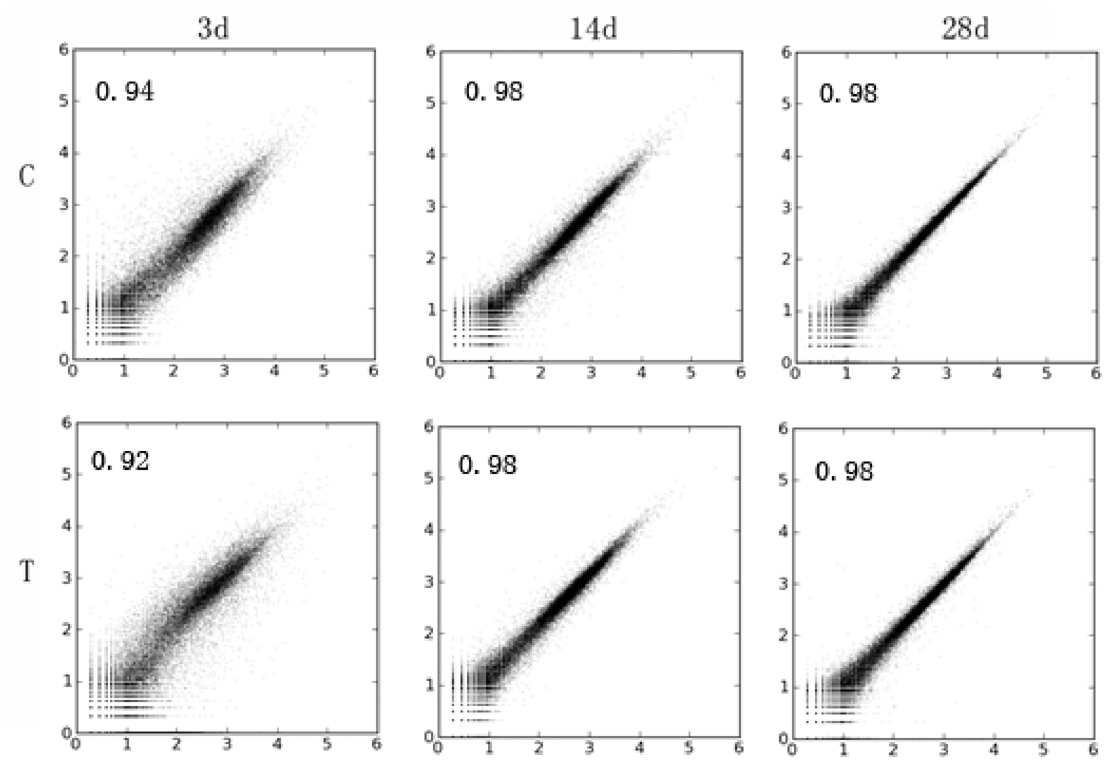

Supplementary Figure S2

The log value (log10) of expression levels of each biological repeats was used to analyze the correction. 3 d, 14 d and 28 d on the upper plot represent the time points. The capital letters on the left of plot represent controls (C) and treatments (T). The numbers on top left of each plot represent the correlation between the biological repeats

Supplementary Figure S2

The expression patterns of 24 selected Cm gene were analyzed using Real-time PCR analysis. The y-axis represents genes relative expression of after *Mi* infected compared to the controls. The X-axis represents the treatment time points. The gene name were listed under X-axis.

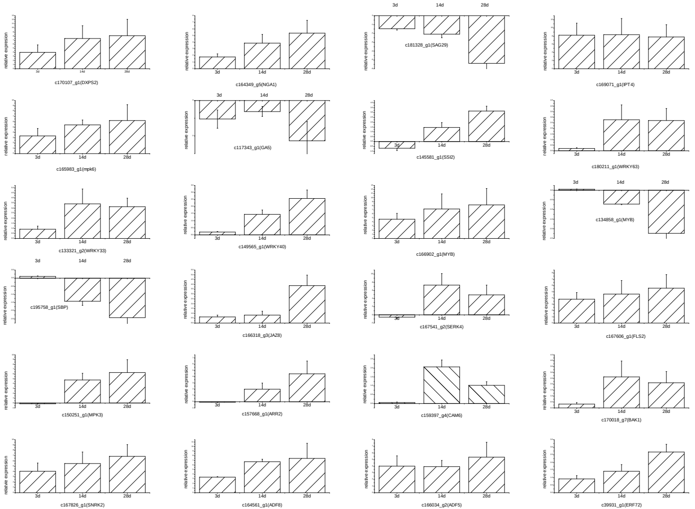
relative expression
3d
14d
28d
c170107_g1(DXPS2)
c164349_g5(NGA1)
c181328_g1(SAG29)
c169071_g1(IPT4)
c165983_g1(mpk6)
c117343_g1(GA5)
c145581_g1(SSI2)
c180211_g1(WRKY63)
c133321_g2(WRKY33)
c149565_g1(WRKY40)
c166902_g1(MYB)
c134858_g1(MYB)
c195758_g1(SBP)
c166318_g3(JAZ8)
c167541_g2(SERK4)
c167606_g1(FLS2)
c150251_g1(MPK3)
c157668_g1(ARR2)
c159397_g4(CAM6)
c170018_g7(BAK1)
c167826_g1(SNRK2)
c164561_g1(ADF8)
c166034_g2(ADF5)
c39931_g1(ERF72)

Supplementary Tables S1

Identification of differentially expressed unigenes of 3 DAI, 14 DAI and 28 DAI. logFC: log2 Fold chang. logCPM: log2-counts-per-million. FDR:false discovery rate.

3 DAI

ID	logFC	logCPM	pvalue	FDR
c114278_g1	11.57858618	3.324693262	0.000636248	0.00096966
c145624_g1	11.51533058	3.262087882	0.000688996	0.00096966
c149798_g1	12.7253368	4.463464992	0.000124158	0.00056855
c163368_g1	8.728513298	0.561751985	0.000967471	0.00091526
c164438_g1	11.55017699	3.296572589	0.00065669	0.00096966
c165643_g1	11.25631317	3.006047541	0.000942033	0.00093325
c165727_g1	9.136726656	0.945612524	0.000684228	0.00096966
c166493_g1	11.40332149	3.151301632	0.000785014	0.00096582
c166600_g1	10.22670354	1.99505292	2.85E-05	0.00038298
c167639_g1	9.18085431	0.987381364	0.000288222	0.00077439
c167773_g1	9.377434869	1.174590299	0.000153469	0.00058596
c167773_g2	9.362764836	1.16053259	0.000125043	0.00056855
c167861_g1	8.717616246	0.551526333	0.000731881	0.00098491
c168212_g3	7.89759393	5.820811007	3.79E-06	0.00022408
c168357_g1	8.204710685	2.293576915	0.00056904	0.00096966
c168538_g1	10.03857275	1.811960354	3.24E-05	0.00038298
c168633_g1	8.992710133	0.809327763	0.000338969	0.00079928
c168794_g1	8.797707653	0.626266662	0.000617294	0.00096966
c169037_g2	14.17775459	5.911695604	2.09E-05	0.00038298
c169038_g1	9.706884386	1.490909599	8.79E-05	0.00056855
c169053_g1	9.078233511	0.890134928	0.000365097	0.00079928
c169118_g2	8.091882442	2.182277682	8.69E-05	0.00056855
c169144_g1	11.68345518	3.428544521	0.000547652	0.00096966
c169335_g1	9.43967198	1.234074103	9.61E-05	0.00056855
c169375_g1	9.657236227	1.443074223	0.000107631	0.00056855
c169573_g9	9.4373089	1.23184628	0.000117704	0.00056855
c169645_g3	12.16025391	3.901538174	0.000280026	0.00077439
c169775_g1	7.241195705	1.360230326	0.000328059	0.00079928
c170009_g2	9.483648716	1.276279519	0.000158611	0.00058596
c170109_g1	9.212119923	1.017045962	0.000221355	0.00072689
c170138_g1	15.65272174	7.385116276	1.01E-05	0.00029922
c170141_g3	8.881586475	0.704667427	0.000354501	0.00079928
c170157_g2	8.63344657	0.473271272	0.000861817	0.00091417
c170163_g1	11.59351214	3.339469801	0.000614531	0.00096966
c170216_g2	9.344521611	1.143292962	0.000651251	0.00096966
c170247_g1	12.28732266	4.027786593	0.00024083	0.00074922
c175822_g1	11.20134806	2.951784965	0.00099827	0.00094912
c175876_g1	12.64701737	4.385513102	0.000137712	0.00058143
c17716_g3	-8.725016487	0.558809379	0.000533093	0.00096966
c180829_g1	11.58420149	3.330252215	0.000632423	0.00096966
c181648_g1	11.61012121	3.355914484	0.000604468	0.00096966
c185765_g1	12.48336824	4.222699193	0.000175539	0.00061035
c187572_g1	11.32723122	3.076097244	0.000857453	0.00097517
c191178_g1	12.93981023	4.677027886	9.09E-05	0.00056855
c191375_g1	11.56160789	3.307886717	0.000647767	0.00096966
c196457_g1	11.84808744	3.591719857	0.000438158	0.00092497
c197011_g1	11.75656044	3.500982474	0.000498092	0.00096966
c198390_g1	11.4054376	3.153393768	0.000784568	0.00095836
c26481_g1	12.18140151	3.922544219	0.000273251	0.00077439
c27310_g1	9.488463534	1.280956585	0.000335975	0.00079928
c40260_g1	11.41176739	3.159652034	0.000781439	0.00098194

logFC: log2 Fold chang

logCPM: log2-counts-per-million
FDR:false discovery rate

14 DAI

ID	logFC	logCPM	PValue	FDR
c101685_g1	7.637817	-0.45886	2.36E-09	3.29E-07
c102249_g1	2.641597	2.904122	1.33E-07	1.29E-05
c105238_g1	4.693664	-0.25632	3.96E-08	4.38E-06
c113101_g1	7.328536	-0.71163	1.35E-07	1.29E-05
c113541_g1	6.851856	-1.09992	5.13E-06	0.000328
c114366_g1	3.610961	3.622116	8.41E-11	1.50E-08
c114534_g1	4.784787	3.802075	4.41E-19	2.97E-16
c114534_g2	4.847991	-0.13409	2.11E-08	2.45E-06
c114773_g1	5.073178	0.07846	1.82E-09	2.60E-07
c114884_g1	9.144562	0.92648	1.66E-17	8.50E-15
c115018_g2	5.736471	-0.13201	1.22E-05	0.000695
c116224_g1	3.01631	3.700745	2.59E-12	6.29E-10
c117571_g1	3.085608	-0.30425	1.02E-05	0.000591
c11806_g1	2.697122	1.482888	5.83E-06	0.000366
c118140_g1	2.378899	6.876896	2.53E-09	3.49E-07
c118260_g1	3.667746	-0.64738	1.45E-05	0.000803
c118318_g1	-3.7405	-0.08713	2.27E-06	0.000158
c118646_g1	2.856568	1.946611	3.52E-07	3.06E-05
c12180_g1	4.921305	7.044205	8.03E-16	3.36E-13
c122451_g2	4.37777	1.953198	1.36E-16	6.11E-14
c123324_g1	7.299224	-0.75012	6.24E-08	6.58E-06
c123443_g1	7.419713	-0.64079	2.14E-08	2.47E-06
c124782_g1	7.965089	-0.17649	4.20E-10	6.68E-08
c125150_g1	8.910345	0.703122	5.96E-16	2.51E-13
c125415_g2	2.115138	5.401188	4.51E-08	4.92E-06
c12562_g1	3.334118	0.103306	1.40E-06	0.000103
c125630_g1	4.819804	0.689168	1.73E-12	4.28E-10
c125700_g1	3.757119	3.545012	2.94E-17	1.47E-14
c126171_g1	9.631577	1.396691	3.26E-21	3.34E-18
c126175_g1	4.283501	-0.6147	1.92E-06	0.000137
c126600_g2	7.563116	-0.51101	1.92E-08	2.25E-06
c128727_g1	2.829959	2.591902	1.51E-11	3.13E-09
c129826_g1	-8.24547	0.16186	4.68E-08	5.07E-06
c130219_g1	2.120144	2.463417	1.85E-06	0.000133
c130794_g1	9.026983	0.816965	2.67E-17	1.35E-14
c130909_g1	6.667318	-1.25513	5.75E-06	0.000363
c131095_g1	2.724998	0.868713	1.24E-05	0.000708
c131236_g1	4.042151	2.227041	1.13E-16	5.34E-14
c132215_g1	8.114124	-0.03225	9.39E-12	2.03E-09
c132727_g1	7.317689	-0.72906	4.71E-08	5.08E-06
c132993_g2	2.057775	3.062709	1.15E-05	0.000661
c133258_g1	9.891716	1.650572	9.95E-24	1.30E-20
c133321_g2	3.373361	4.508149	3.17E-11	6.10E-09
c133407_g1	6.76038	-1.17697	5.86E-06	0.000366
c133911_g1	3.097999	0.340212	1.77E-06	0.000128
c134084_g1	6.895919	-1.07889	1.53E-06	0.000112
c134638_g1	13.20877	4.941861	5.00E-07	4.15E-05
c136792_g1	3.840061	0.112035	1.18E-07	1.15E-05
c137151_g2	2.656225	2.29392	5.77E-10	9.02E-08
c138860_g1	7.478459	-0.5972	1.13E-08	1.36E-06
c139115_g1	3.674934	3.084961	4.53E-17	2.21E-14
c139668_g1	3.16113	2.543849	9.94E-08	9.89E-06
c141318_g1	3.518322	2.754911	1.48E-14	4.98E-12

c142939_g2	3.243958	2.12767	3.26E-10	5.27E-08
c144077_g2	3.306074	-0.32971	1.11E-05	0.000642
c144990_g1	2.419645	1.271914	1.21E-06	9.09E-05
c145163_g1	3.029149	4.856642	3.15E-15	1.21E-12
c145441_g1	6.872529	-1.10775	4.99E-06	0.000321
c145604_g2	-4.55549	-0.96224	1.38E-05	0.000773
c145742_g1	2.048278	3.089218	1.90E-07	1.76E-05
c146621_g1	7.802093	-0.3125	3.54E-10	5.67E-08
c146670_g3	8.357956	0.204017	4.24E-11	7.97E-09
c146694_g1	6.722413	-1.21182	4.01E-06	0.000264
c146790_g1	-4.64462	-0.88148	9.38E-06	0.00055
c147108_g1	7.143841	-0.86944	2.73E-07	2.45E-05
c147690_g1	6.065582	0.179141	1.53E-11	3.17E-09
c147979_g1	6.626531	-1.29243	1.38E-05	0.000773
c147987_g2	3.180658	0.987405	2.62E-09	3.60E-07
c148189_g1	7.738968	-0.37374	1.35E-09	1.98E-07
c148532_g1	3.233163	1.115725	1.10E-07	1.07E-05
c148599_g1	2.983618	1.886478	4.53E-07	3.82E-05
c148610_g1	5.101464	0.107105	3.69E-10	5.89E-08
c148610_g2	4.299678	-0.60548	1.38E-06	0.000102
c150348_g3	6.602433	-1.30352	8.28E-06	0.000494
c150516_g1	4.752573	-0.95424	1.27E-05	0.000719
c150564_g1	2.705228	3.507338	2.07E-09	2.92E-07
c150613_g1	7.096826	-0.9066	6.10E-07	4.93E-05
c150647_g1	7.525097	-0.55175	7.02E-09	8.75E-07
c150774_g2	2.864878	2.460483	5.49E-09	6.95E-07
c151038_g1	2.638349	2.985753	1.47E-07	1.40E-05
c151042_g1	6.805579	-1.15548	5.49E-06	0.000349
c151144_g1	8.587502	0.407492	5.24E-14	1.64E-11
c151209_g1	7.06224	-0.93793	4.24E-07	3.60E-05
c151344_g1	8.422026	2.43558	1.07E-27	2.71E-24
c151432_g2	2.433296	3.175241	7.41E-10	1.14E-07
c152753_g1	2.606963	1.894428	6.28E-07	5.04E-05
c153334_g1	2.222966	0.895201	1.01E-05	0.000586
c153473_g2	3.61101	1.791054	1.39E-09	2.02E-07
c153505_g1	4.300221	-0.10045	6.42E-08	6.71E-06
c153528_g1	7.563117	-0.51101	1.92E-08	2.25E-06
c153528_g2	6.915464	-1.05436	2.05E-06	0.000143
c153659_g1	9.544949	1.314673	3.71E-21	3.72E-18
c154125_g1	7.94278	-0.19742	5.56E-10	8.72E-08
c154142_g1	8.280244	0.118854	1.01E-12	2.67E-10
c154560_g1	6.87542	-1.08942	1.46E-06	0.000107
c154754_g1	6.471658	-1.40131	1.77E-05	0.000959
c155258_g2	2.0205	2.27877	3.03E-06	0.000204
c155293_g2	2.228056	2.970332	9.42E-08	9.41E-06
c155775_g1	6.609932	-1.29983	8.65E-06	0.000512
c156113_g1	8.210245	0.05262	3.65E-12	8.58E-10
c156192_g1	8.222825	0.070389	4.09E-12	9.49E-10
c156270_g1	4.680172	2.001563	4.29E-16	1.84E-13
c156270_g2	3.522857	1.587893	8.48E-11	1.51E-08
c156295_g1	9.566872	1.336141	2.74E-21	2.91E-18
c156390_g1	8.045812	-0.088	6.05E-11	1.11E-08
c156486_g1	5.313487	-0.47753	6.35E-08	6.68E-06
c156489_g1	8.101346	-0.0459	1.07E-11	2.29E-09

c156553_g1	8.400198	0.235446	1.22E-12	3.17E-10
c156643_g2	7.58969	-0.49618	3.75E-09	4.96E-07
c156692_g1	7.257684	-0.77263	1.94E-07	1.80E-05
c156792_g1	7.287591	-0.75656	6.50E-08	6.75E-06
c156993_g1	7.603202	-0.47946	6.07E-09	7.63E-07
c157118_g1	3.905615	0.168483	4.70E-08	5.08E-06
c157128_g1	7.012678	-0.95811	8.16E-06	0.000491
c157257_g1	8.549612	0.36709	3.60E-14	1.16E-11
c157307_g1	8.522866	0.34424	4.86E-14	1.53E-11
c157328_g2	7.175929	-0.83969	4.32E-07	3.66E-05
c157588_g1	6.659986	-1.25879	5.87E-06	0.000366
c157621_g1	8.579758	2.589775	3.05E-27	7.36E-24
c157668_g1	2.017419	2.356084	3.49E-06	0.000232
c157880_g1	6.722413	-1.21182	4.01E-06	0.000264
c158000_g1	6.868205	-1.09292	1.81E-06	0.00013
c158225_g1	8.157448	0.018178	1.18E-10	2.02E-08
c158707_g1	6.65238	-1.26244	6.78E-06	0.000419
c158742_g1	3.086827	4.575595	4.65E-12	1.06E-09
c158988_g1	8.94437	0.736136	2.26E-16	1.01E-13
c159031_g1	3.452357	1.323201	4.20E-11	7.91E-09
c159125_g1	6.764136	-1.19371	1.07E-05	0.000615
c159160_g1	9.226322	1.00972	1.50E-18	9.52E-16
c159240_g3	2.113656	4.849033	2.70E-09	3.70E-07
c159299_g1	8.221204	0.059988	6.98E-12	1.54E-09
c159320_g1	4.852744	1.97249	8.38E-18	4.64E-15
c159397_g4	2.069602	5.238427	9.04E-09	1.11E-06
c159560_g1	2.232396	3.227336	8.76E-06	0.000517
c159600_g1	5.162668	3.370028	3.86E-24	6.04E-21
c159612_g1	2.942828	7.81096	2.04E-13	5.99E-11
c159624_g1	9.404269	1.179482	4.68E-20	4.08E-17
c159706_g1	2.945653	2.517689	6.09E-11	1.12E-08
c159967_g2	9.324316	1.100945	2.52E-19	1.84E-16
c159999_g1	4.437198	-0.48442	8.71E-07	6.80E-05
c159999_g2	3.149964	1.361648	8.14E-07	6.37E-05
c160061_g1	3.839548	-0.18578	2.67E-07	2.41E-05
c160075_g1	9.579607	1.35081	1.02E-20	9.56E-18
c160089_g1	8.356051	0.173397	6.97E-11	1.26E-08
c160139_g1	7.052438	-0.95645	5.90E-07	4.79E-05
c160139_g2	8.305309	0.149474	5.72E-12	1.28E-09
c160140_g1	3.430293	1.453798	3.35E-11	6.43E-09
c160140_g2	3.361831	2.004681	1.16E-12	3.05E-10
c160284_g4	3.058209	5.20959	1.63E-08	1.93E-06
c160313_g1	7.045423	-0.95986	6.17E-07	4.96E-05
c160333_g1	6.968418	-1.0134	8.89E-07	6.86E-05
c160412_g1	6.699081	-1.22266	1.02E-05	0.00059
c160451_g1	2.177943	5.62819	4.26E-08	4.66E-06
c160508_g1	6.644076	-1.26608	1.28E-05	0.000722
c160520_g1	6.775353	-1.16982	2.87E-06	0.000194
c160557_g1	6.819052	-1.13258	2.47E-06	0.00017
c160631_g1	6.652378	-1.26244	6.79E-06	0.000419
c160686_g2	2.049535	6.404085	2.33E-07	2.12E-05
c160846_g1	2.288646	5.209304	2.06E-07	1.89E-05
c160887_g1	10.1275	5.48275	8.19E-06	0.000493
c160902_g1	2.491803	1.30463	2.15E-07	1.97E-05

c161026_g1	7.473153	-0.60027	9.68E-09	1.19E-06
c161058_g1	7.129633	-0.87605	7.50E-07	5.90E-05
c161149_g1	7.402838	-0.66021	1.95E-08	2.27E-06
c161191_g1	3.625383	-0.67025	1.67E-05	0.000912
c161191_g3	5.177657	-0.5893	3.23E-07	2.86E-05
c161267_g1	7.589689	-0.49618	3.75E-09	4.96E-07
c161334_g1	7.293333	-0.75334	7.08E-08	7.28E-06
c161372_g1	7.341554	1.368828	1.20E-14	4.10E-12
c161373_g1	6.907633	-1.05782	2.83E-06	0.000193
c161384_g1	8.953078	0.739982	4.62E-15	1.72E-12
c161510_g2	4.852645	2.197287	1.14E-17	6.01E-15
c161530_g1	6.594848	-1.30721	9.04E-06	0.000531
c161620_g1	9.404374	1.174315	2.18E-18	1.32E-15
c161770_g1	7.243548	-0.77905	4.53E-07	3.82E-05
c161902_g1	6.922723	-1.05089	1.14E-06	8.58E-05
c161965_g1	7.651528	-0.44263	2.04E-09	2.89E-07
c161970_g1	7.842844	-0.27332	2.89E-10	4.69E-08
c161999_g1	2.975	6.96693	1.31E-16	5.94E-14
c162137_g1	7.63674	-0.45138	3.69E-09	4.90E-07
c162191_g1	6.848162	1.728047	9.68E-19	6.27E-16
c162191_g2	9.305124	1.082504	3.47E-19	2.43E-16
c162558_g1	7.693134	-0.40933	1.11E-09	1.64E-07
c162616_g1	6.875424	-1.08942	1.46E-06	0.000107
c162660_g1	7.796677	1.824112	8.24E-24	1.12E-20
c162665_g1	7.375126	-0.67585	4.24E-08	4.65E-06
c162739_g3	6.622183	2.029783	1.18E-20	1.08E-17
c162742_g4	4.25473	5.803125	9.64E-18	5.23E-15
c162787_g1	6.94466	-1.02369	3.67E-06	0.000243
c162806_g1	6.558555	-1.34227	1.70E-05	0.000925
c162831_g1	2.801931	3.863496	1.26E-13	3.77E-11
c163074_g1	7.864301	-0.25955	2.25E-10	3.70E-08
c163088_g2	3.39758	0.767882	6.46E-08	6.73E-06
c163088_g3	4.15577	1.009937	4.02E-11	7.63E-09
c163115_g2	6.609929	-1.29983	8.63E-06	0.000512
c163120_g1	7.038783	-0.96327	5.20E-07	4.30E-05
c163126_g1	7.715439	-0.38809	8.14E-10	1.24E-07
c163131_g1	8.001684	-0.13393	3.52E-11	6.72E-09
c163241_g1	2.874425	0.74995	1.57E-06	0.000114
c163311_g1	7.938011	-0.20013	4.47E-10	7.10E-08
c163330_g1	7.97389	-0.16387	9.59E-11	1.68E-08
c163371_g4	2.266778	1.847069	1.13E-05	0.000651
c163393_g1	6.609932	-1.29983	8.65E-06	0.000512
c163448_g3	2.321837	3.952541	4.50E-09	5.86E-07
c163592_g9	2.76926	6.37287	4.61E-11	8.57E-09
c163615_g1	6.889699	-1.06475	7.06E-06	0.000432
c163655_g1	6.961147	-1.01683	1.62E-06	0.000118
c163667_g1	7.754506	-0.36513	4.19E-09	5.48E-07
c163669_g1	7.905773	-0.22688	3.28E-10	5.28E-08
c163717_g1	6.71515	-1.21544	4.32E-06	0.000282
c163953_g5	2.041378	6.777676	1.83E-06	0.000131
c163993_g1	7.081261	-0.92782	3.42E-07	2.98E-05
c163994_g4	3.898757	2.726371	1.51E-12	3.85E-10
c164024_g1	7.038782	-0.96327	5.20E-07	4.30E-05
c164119_g1	8.701605	0.506684	8.51E-15	3.02E-12

c164171_g1	7.148807	-0.87988	4.19E-07	3.58E-05
c164270_g1	4.595548	-1.0489	1.44E-05	0.000801
c164297_g1	8.106671	-0.03731	1.06E-11	2.27E-09
c164349_g5	3.850157	0.550638	4.14E-08	4.56E-06
c164381_g1	9.152142	0.934285	1.09E-17	5.81E-15
c164435_g1	7.110158	-0.89993	2.72E-07	2.45E-05
c164509_g1	8.573203	0.390686	2.28E-14	7.47E-12
c164516_g1	6.471659	-1.40131	1.77E-05	0.000959
c164525_g1	7.712926	-0.38143	3.39E-09	4.52E-07
c164527_g1	6.964984	-1.03004	1.60E-06	0.000117
c164561_g1	6.775353	-1.16982	2.86E-06	0.000194
c164627_g1	6.760382	-1.17697	5.86E-06	0.000366
c164637_g1	6.903182	-1.07538	1.90E-06	0.000136
c164684_g1	7.328534	-0.71163	1.35E-07	1.29E-05
c164761_g1	7.281899	-0.75978	6.46E-08	6.73E-06
c164872_g1	9.3554	1.130452	1.76E-19	1.36E-16
c164874_g1	6.742457	-1.18411	1.25E-05	0.000711
c164903_g1	6.981828	-1.00652	6.84E-07	5.45E-05
c165004_g1	7.110159	-0.89993	2.72E-07	2.45E-05
c165036_g2	6.581296	2.381808	8.26E-26	1.51E-22
c165084_g1	4.815569	3.243417	2.99E-21	3.12E-18
c165109_g1	8.024698	-0.10741	7.03E-11	1.27E-08
c165192_g1	7.473153	-0.60027	9.70E-09	1.19E-06
c165193_g1	8.489039	0.309613	8.90E-14	2.73E-11
c165227_g2	6.644074	-1.26608	1.28E-05	0.000722
c165238_g1	9.127051	0.914144	5.83E-18	3.36E-15
c165246_g1	7.012603	-0.97688	7.00E-07	5.54E-05
c165248_g1	8.400197	0.235446	1.22E-12	3.17E-10
c165257_g1	7.068729	-0.93456	3.58E-07	3.09E-05
c165273_g1	8.419371	0.245086	2.54E-13	7.28E-11
c165298_g1	6.715149	-1.21544	4.32E-06	0.000282
c165312_g1	6.889698	-1.06475	7.06E-06	0.000432
c165350_g1	6.915467	-1.05436	2.04E-06	0.000143
c165361_g1	8.135041	-0.00796	2.09E-11	4.18E-09
c165365_g1	7.062239	-0.93793	4.24E-07	3.60E-05
c165398_g1	6.586674	-1.3109	1.65E-05	0.000903
c165424_g1	6.602433	-1.30352	8.27E-06	0.000494
c165474_g1	7.189303	-0.83315	1.73E-07	1.63E-05
c165475_g1	9.230285	1.012929	1.12E-18	7.19E-16
c165488_g1	8.878817	0.678762	3.58E-16	1.56E-13
c165491_g1	7.250831	-0.77584	2.88E-07	2.57E-05
c165505_g1	8.412955	0.248455	1.70E-12	4.27E-10
c165515_g1	7.734082	-0.37661	1.32E-09	1.94E-07
c165544_g1	7.334587	-0.7195	4.10E-08	4.53E-06
c165577_g1	9.770143	1.533028	6.25E-23	7.22E-20
c165585_g1	7.182819	-0.83642	2.27E-07	2.06E-05
c165633_g1	7.540238	-0.54269	5.27E-09	6.73E-07
c165639_g1	8.578813	0.383315	2.63E-12	6.35E-10
c165647_g1	6.691709	-1.24415	1.13E-05	0.000651
c165659_g1	6.903184	-1.07538	1.91E-06	0.000136
c165672_g1	13.16953	4.898528	1.30E-60	3.46E-56
c165705_g2	7.025974	-0.97008	4.89E-07	4.07E-05
c165726_g1	6.889695	-1.06475	7.07E-06	0.000432
c165727_g1	10.75165	2.494446	1.51E-30	6.69E-27

c165743_g1	7.425995	-0.64768	5.19E-08	5.52E-06
c165747_g1	7.306157	-0.73543	6.41E-08	6.71E-06
c165748_g1	6.833243	-1.1255	1.96E-06	0.000138
c165764_g1	7.156516	-0.86283	1.88E-07	1.75E-05
c165875_g1	3.272183	2.023514	3.06E-11	5.91E-09
c165875_g2	4.042004	1.292043	2.99E-11	5.81E-09
c165875_g5	3.238873	1.840834	6.48E-12	1.43E-09
c165886_g1	6.644072	-1.26608	1.28E-05	0.000722
c165890_g1	8.934856	0.728773	1.23E-16	5.65E-14
c165913_g1	6.609933	-1.29983	8.67E-06	0.000512
c165922_g1	9.48998	1.26445	5.42E-20	4.65E-17
c165946_g1	6.782413	-1.16624	2.85E-06	0.000194
c165948_g1	7.025973	-0.97008	4.90E-07	4.07E-05
c165983_g1	6.81905	-1.13258	2.47E-06	0.00017
c165992_g1	6.826291	-1.12904	2.08E-06	0.000145
c165999_g1	6.689699	-1.22626	1.51E-05	0.000836
c166006_g1	6.847435	-1.1184	2.47E-06	0.00017
c166100_g1	6.942871	-1.04047	1.00E-06	7.65E-05
c166109_g1	7.040127	-0.94803	1.64E-06	0.000118
c166224_g1	7.103678	-0.90327	3.35E-07	2.93E-05
c166254_g1	7.124497	-0.85929	1.29E-05	0.000725
c166275_g1	7.681736	-0.4353	3.32E-08	3.74E-06
c166280_g1	7.306157	-0.73543	6.41E-08	6.71E-06
c166329_g1	6.471659	-1.40131	1.77E-05	0.000959
c166343_g1	2.319579	5.227459	3.14E-09	4.22E-07
c166362_g1	6.842443	-1.10341	8.29E-06	0.000494
c166380_g1	7.136968	-0.87275	5.09E-07	4.22E-05
c166404_g1	6.868207	-1.09292	1.81E-06	0.00013
c166405_g1	7.005275	-0.98028	1.33E-06	9.86E-05
c166420_g1	6.775352	-1.16982	2.87E-06	0.000194
c166423_g1	7.680513	-0.42512	3.01E-09	4.05E-07
c166457_g1	8.53191	0.354111	6.67E-14	2.07E-11
c166468_g1	7.089476	-0.90994	8.85E-07	6.86E-05
c166473_g1	7.25688	-0.78469	1.06E-07	1.05E-05
c166486_g1	5.453402	-0.34508	1.29E-08	1.55E-06
c166501_g1	7.799832	-0.30664	1.06E-09	1.57E-07
c166512_g1	6.903182	-1.07538	1.90E-06	0.000136
c166520_g1	7.175928	-0.83969	4.32E-07	3.66E-05
c166525_g1	7.206551	-0.81064	5.49E-07	4.51E-05
c166543_g1	6.944659	-1.02369	3.67E-06	0.000243
c166544_g1	6.949799	-1.037	1.21E-06	9.04E-05
c166594_g1	8.85795	0.655984	4.84E-16	2.06E-13
c166599_g1	6.768118	-1.17339	3.23E-06	0.000216
c166600_g1	9.877979	1.637917	7.19E-24	1.03E-20
c166610_g1	6.625447	0.704154	1.45E-14	4.92E-12
c166625_g1	7.397418	-0.66334	2.45E-08	2.78E-06
c166636_g1	6.981828	-1.00652	6.84E-07	5.45E-05
c166647_g1	6.860398	-1.09642	3.47E-06	0.000231
c166652_g1	7.941049	-0.18522	1.04E-10	1.80E-08
c166679_g1	7.06224	-0.93793	4.24E-07	3.60E-05
c166688_g1	9.349171	1.126544	1.24E-19	1.01E-16
c166692_g1	6.968417	-1.0134	8.90E-07	6.86E-05
c166693_g1	9.117154	0.904561	6.58E-18	3.72E-15
c166697_g1	6.471659	-1.40131	1.77E-05	0.000959

c166717_g1	8.049943	-0.08543	4.34E-11	8.11E-09
c166724_g1	6.586674	-1.3109	1.65E-05	0.000903
c166725_g1	6.903181	-1.07538	1.90E-06	0.000136
c166736_g1	4.743289	0.929664	3.47E-12	8.18E-10
c166736_g2	4.972684	1.156634	6.80E-14	2.10E-11
c166745_g1	6.602433	-1.30352	8.28E-06	0.000494
c166752_g1	7.790524	-0.36012	8.51E-07	6.65E-05
c166752_g2	9.180589	0.947822	5.67E-13	1.56E-10
c166770_g1	6.811256	-1.13611	4.60E-06	0.000298
c166775_g2	6.953246	-1.02026	2.35E-06	0.000163
c166777_g1	6.842445	-1.10341	8.28E-06	0.000494
c166808_g1	7.820853	-0.30999	4.99E-09	6.42E-07
c166821_g1	6.889014	-1.0824	1.35E-06	0.0001
c166833_g1	6.61783	-1.29613	1.03E-05	0.000596
c166893_g1	6.691707	-1.24415	1.13E-05	0.000651
c166894_g1	7.604352	-0.48727	2.90E-09	3.95E-07
c166902_g1	7.720024	-0.38522	8.32E-10	1.27E-07
c166908_g1	7.862595	-0.2542	2.79E-10	4.55E-08
c166918_g1	7.470467	-0.61286	5.06E-08	5.40E-06
c166968_g1	6.609932	-1.29983	8.66E-06	0.000512
c166994_g1	7.328884	-0.72269	4.81E-08	5.16E-06
c166995_g1	6.534681	-1.35347	1.25E-05	0.000708
c166999_g2	6.760378	-1.17697	5.87E-06	0.000366
c167027_g3	6.824444	-1.1483	9.51E-06	0.000557
c167047_g2	3.349028	6.593548	3.04E-22	3.30E-19
c167054_g1	7.08764	-0.92445	3.91E-07	3.35E-05
c167087_g1	10.66608	2.402891	5.33E-19	3.54E-16
c167087_g2	7.113706	-0.93253	1.23E-05	0.000701
c167098_g2	6.768119	-1.17339	3.23E-06	0.000216
c167115_g1	7.911585	-0.2164	1.12E-10	1.94E-08
c167120_g1	6.775353	-1.16982	2.86E-06	0.000194
c167139_g1	7.352952	-0.70993	9.53E-08	9.51E-06
c167141_g1	7.18282	-0.83642	2.26E-07	2.06E-05
c167161_g1	8.115239	-0.02631	2.26E-11	4.47E-09
c167173_g1	9.199708	0.985274	4.83E-18	2.89E-15
c167177_g1	7.494279	-0.57882	9.04E-09	1.11E-06
c167185_g1	6.979581	-0.99047	5.26E-06	0.000335
c167200_g6	2.118693	3.531109	5.71E-06	0.000362
c167251_g1	6.659986	-1.25879	5.87E-06	0.000366
c167274_g1	6.667318	-1.25513	5.75E-06	0.000363
c167289_g1	8.045813	-0.088	6.05E-11	1.11E-08
c167291_g1	7.35256	-0.69901	3.56E-08	3.98E-06
c167313_g1	7.256876	-0.78469	1.06E-07	1.05E-05
c167334_g1	8.528795	0.348593	4.37E-14	1.39E-11
c167358_g1	7.7464	-0.36152	6.09E-10	9.49E-08
c167378_g1	8.040779	-0.09706	2.20E-11	4.37E-09
c167379_g1	6.782413	-1.16624	2.85E-06	0.000194
c167399_g1	8.793765	0.599737	2.22E-15	8.93E-13
c167404_g1	9.536717	1.305724	6.62E-21	6.29E-18
c167429_g1	6.97958	-0.99047	5.26E-06	0.000335
c167430_g1	4.469497	0.905743	1.37E-12	3.51E-10
c167476_g1	8.779952	0.579077	6.38E-15	2.32E-12
c167486_g1	7.871261	-0.24874	1.51E-10	2.54E-08
c167487_g1	7.075015	-0.93119	3.33E-07	2.92E-05

c167510_g1	6.925391	-1.03054	1.05E-05	0.000607
c167514_g3	7.669497	-0.4238	2.24E-09	3.14E-07
c167541_g1	7.732723	-0.37005	9.91E-10	1.49E-07
c167541_g2	7.280575	2.154109	6.89E-26	1.31E-22
c167556_g1	8.735049	0.545336	6.99E-15	2.53E-12
c167565_g1	6.609932	-1.29983	8.66E-06	0.000512
c167582_g1	6.961148	-1.01683	1.62E-06	0.000118
c167583_g1	6.715149	-1.21544	4.32E-06	0.000282
c167586_g1	7.651528	-0.44263	2.04E-09	2.89E-07
c167592_g2	7.233403	-0.79768	1.07E-07	1.06E-05
c167598_g1	7.397418	-0.66334	2.44E-08	2.78E-06
c167606_g1	6.644079	-1.26608	1.28E-05	0.000722
c167621_g1	7.540238	-0.54269	5.26E-09	6.73E-07
c167632_g1	8.181471	0.03295	9.12E-12	1.98E-09
c167639_g1	8.369447	0.210876	9.04E-12	1.97E-09
c167646_g1	6.910995	-1.07187	2.60E-06	0.000179
c167649_g6	2.494073	8.067203	2.78E-15	1.09E-12
c167696_g29	2.468633	1.922422	4.90E-06	0.000317
c167759_g1	6.953244	-1.02026	2.35E-06	0.000163
c167760_g1	6.471659	-1.40131	1.77E-05	0.000959
c167773_g1	10.82669	2.566548	3.51E-27	8.11E-24
c167773_g2	10.84824	2.588643	5.98E-29	1.67E-25
c167780_g1	7.594586	-0.49321	3.51E-09	4.67E-07
c167787_g1	6.840198	-1.12195	2.08E-06	0.000145
c167788_g1	8.5098	0.338935	8.31E-13	2.25E-10
c167794_g1	9.716659	1.481495	1.59E-22	1.76E-19
c167795_g2	7.025974	-0.97008	4.89E-07	4.07E-05
c167820_g3	6.67481	-1.25148	6.45E-06	0.000401
c167820_g4	6.775352	-1.16982	2.87E-06	0.000194
c167826_g1	6.99735	-0.98368	1.98E-06	0.000139
c167861_g1	7.446324	-0.60316	2.04E-07	1.88E-05
c167869_g1	6.689697	-1.22626	1.51E-05	0.000836
c167885_g1	6.928457	-1.06483	4.39E-06	0.000285
c167925_g1	6.707479	-1.21905	7.56E-06	0.000459
c167961_g2	7.239679	-0.80692	3.23E-07	2.86E-05
c167975_g1	8.952194	0.748414	1.19E-16	5.57E-14
c167977_g1	7.430458	-0.63459	1.78E-08	2.09E-06
c167984_g1	8.397121	0.22914	3.66E-13	1.03E-10
c167986_g1	6.652379	-1.26244	6.78E-06	0.000419
c167993_g1	7.784249	-0.32369	6.79E-10	1.05E-07
c167999_g1	6.936208	-1.04395	9.37E-07	7.20E-05
c168006_g2	7.710841	-0.39096	1.11E-09	1.65E-07
c168019_g2	8.467309	0.290399	1.14E-13	3.44E-11
c168032_g1	7.386787	-0.68005	5.90E-08	6.24E-06
c168041_g1	6.942871	-1.04047	1.00E-06	7.65E-05
c168048_g1	7.503358	-0.56382	2.57E-08	2.92E-06
c168069_g1	9.799859	1.562405	3.58E-23	4.22E-20
c168074_g1	8.048488	-0.09188	1.95E-11	3.95E-09
c168081_g1	9.118581	0.907406	1.32E-17	6.80E-15
c168082_g1	6.833243	-1.1255	1.96E-06	0.000138
c168083_g1	7.012604	-0.97688	7.00E-07	5.54E-05
c168092_g1	7.116387	-0.89659	2.44E-07	2.21E-05
c168105_g1	6.699072	-1.22266	1.02E-05	0.00059
c168125_g1	7.732722	-0.37005	9.92E-10	1.49E-07

c168144_g1	8.898771	0.701048	1.57E-15	6.39E-13
c168152_g1	7.239337	-0.79443	9.40E-08	9.41E-06
c168156_g1	9.112087	0.902344	2.97E-17	1.48E-14
c168159_g1	7.473154	-0.60027	9.71E-09	1.19E-06
c168162_g2	2.355358	1.532108	4.40E-07	3.72E-05
c168178_g1	2.740499	2.825682	3.95E-09	5.20E-07
c168178_g2	6.929568	-1.04742	9.83E-07	7.50E-05
c168182_g6	2.122718	3.803234	7.65E-07	6.02E-05
c168183_g1	7.352559	-0.69901	3.56E-08	3.98E-06
c168212_g3	4.084389	3.077116	7.88E-18	4.41E-15
c168214_g1	6.935411	-1.02711	6.01E-06	0.000374
c168239_g1	6.872528	-1.10775	4.99E-06	0.000321
c168252_g1	6.953244	-1.02026	2.35E-06	0.000163
c168256_g1	6.707482	-1.21905	7.55E-06	0.000459
c168266_g1	6.832174	-1.10691	1.42E-05	0.000791
c168273_g1	7.062242	-0.93793	4.23E-07	3.60E-05
c168306_g1	6.889698	-1.06475	7.06E-06	0.000432
c168307_g1	7.509666	-0.56968	7.44E-09	9.26E-07
c168307_g2	8.486152	0.303778	3.25E-13	9.19E-11
c168318_g1	6.968419	-1.0134	8.88E-07	6.86E-05
c168326_g4	6.093072	4.882426	3.11E-44	2.76E-40
c168327_g1	7.075016	-0.93119	3.33E-07	2.92E-05
c168341_g1	6.988792	-0.98707	3.14E-06	0.000211
c168370_g1	6.699075	-1.22266	1.02E-05	0.00059
c168375_g1	7.477055	-0.60976	8.04E-08	8.17E-06
c168377_g1	7.794855	-0.30941	1.58E-09	2.29E-07
c168391_g1	8.403539	0.233699	2.72E-13	7.78E-11
c168409_g1	7.386555	-0.66959	2.73E-08	3.09E-06
c168410_g1	6.217648	6.124975	7.78E-47	8.26E-43
c168410_g2	2.713423	1.627535	5.28E-09	6.73E-07
c168415_g1	8.229994	0.07524	2.68E-12	6.45E-10
c168421_g6	4.549579	1.00149	1.83E-12	4.51E-10
c168428_g1	7.225921	-0.81348	1.60E-07	1.52E-05
c168455_g1	6.982468	-1.02307	3.93E-06	0.000259
c168468_g2	7.089478	-0.90994	8.84E-07	6.86E-05
c168472_g1	6.936208	-1.04395	9.40E-07	7.20E-05
c168477_g1	6.802697	-1.13965	6.65E-06	0.000412
c168479_g1	8.339579	0.172902	5.04E-13	1.40E-10
c168481_g1	7.044359	-0.92658	1.60E-05	0.000879
c168502_g1	7.328535	-0.71163	1.35E-07	1.29E-05
c168509_g1	7.045423	-0.95986	6.17E-07	4.96E-05
c168520_g1	8.062364	-0.07141	8.00E-11	1.44E-08
c168528_g1	2.199209	1.719746	7.23E-07	5.72E-05
c168537_g1	8.308951	0.147246	9.37E-13	2.50E-10
c168538_g1	9.043036	0.835169	3.82E-17	1.88E-14
c168550_g1	7.679148	-0.41801	1.52E-09	2.21E-07
c168559_g1	6.915464	-1.05436	2.05E-06	0.000143
c168565_g26	2.487564	1.203595	1.05E-06	7.98E-05
c168567_g1	7.25688	-0.78469	1.06E-07	1.05E-05
c168575_g1	7.397418	-0.66334	2.45E-08	2.78E-06
c168579_g1	7.213398	-0.82004	1.44E-07	1.37E-05
c168591_g2	5.644914	-0.19623	1.53E-09	2.22E-07
c168600_g3	6.742457	-1.18411	1.25E-05	0.000711
c168604_g1	8.316111	0.156509	1.95E-12	4.79E-10

c168605_g1	4.31406	1.712769	9.90E-15	3.44E-12
c168605_g5	5.08366	1.219915	2.31E-15	9.21E-13
c168617_g1	6.802697	-1.13965	6.65E-06	0.000412
c168622_g1	7.743997	-0.37087	1.89E-09	2.69E-07
c168631_g1	7.47244	-0.59099	2.33E-08	2.68E-06
c168633_g1	6.875257	0.93865	1.23E-16	5.65E-14
c168645_g1	9.442578	1.217113	3.04E-20	2.73E-17
c168663_g1	8.16474	0.011999	5.30E-12	1.19E-09
c168669_g1	8.954971	0.748604	8.60E-17	4.08E-14
c168672_g1	7.305327	-0.7469	7.98E-08	8.13E-06
c168690_g1	7.143841	-0.86944	2.73E-07	2.45E-05
c168701_g1	7.045423	-0.95986	6.17E-07	4.96E-05
c168718_g1	7.56166	-0.5306	1.41E-08	1.68E-06
c168735_g1	8.833116	0.636896	1.10E-15	4.59E-13
c168740_g1	7.641802	-0.44846	2.84E-09	3.88E-07
c168742_g4	6.826292	-1.12904	2.08E-06	0.000145
c168746_g1	8.671678	0.481511	5.39E-15	1.97E-12
c168748_g1	6.994987	-0.99964	7.89E-07	6.19E-05
c168750_g1	8.230055	0.080323	1.22E-11	2.59E-09
c168755_g1	10.62399	2.368552	1.68E-29	5.26E-26
c168772_g1	6.644077	-1.26608	1.28E-05	0.000722
c168775_g4	6.699079	-1.22266	1.02E-05	0.00059
c168776_g1	9.878211	1.640077	2.16E-23	2.61E-20
c168786_g1	7.28803	1.331324	7.49E-20	6.22E-17
c168790_g1	6.915465	-1.05436	2.04E-06	0.000143
c168794_g1	8.045813	-0.088	6.05E-11	1.11E-08
c168798_g1	7.207414	-0.82332	1.31E-07	1.27E-05
c168799_g1	7.772287	-0.33821	4.61E-10	7.29E-08
c168808_g1	6.707484	-1.21905	7.55E-06	0.000459
c168814_g1	7.317689	-0.72906	4.71E-08	5.08E-06
c168815_g1	6.851854	-1.09992	5.14E-06	0.000328
c168818_g28	2.012479	5.257019	3.82E-08	4.25E-06
c168822_g1	7.250967	-0.78794	9.37E-08	9.40E-06
c168827_g1	9.275327	1.059901	1.08E-17	5.79E-15
c168838_g1	7.65376	-0.43247	8.36E-09	1.04E-06
c168845_g1	6.542313	-1.34974	1.20E-05	0.000689
c168863_g3	7.048069	-0.94467	1.06E-06	8.04E-05
c168868_g1	7.264133	-0.76942	1.06E-07	1.05E-05
c168875_g2	6.594848	-1.30721	9.04E-06	0.000531
c168879_g1	9.134787	0.921986	5.93E-18	3.39E-15
c168884_g3	3.802118	7.339794	2.31E-19	1.71E-16
c168887_g1	8.649491	0.465031	2.06E-14	6.83E-12
c168899_g1	7.305328	-0.7469	7.98E-08	8.13E-06
c168912_g1	6.875424	-1.08942	1.46E-06	0.000107
c168933_g1	7.457489	-0.60949	1.35E-08	1.62E-06
c168933_g2	8.774794	0.586101	2.94E-14	9.58E-12
c168942_g2	6.889014	-1.0824	1.35E-06	0.0001
c168943_g1	7.664454	-0.42669	3.00E-09	4.05E-07
c168946_g1	8.997117	0.787669	6.03E-17	2.89E-14
c168951_g1	8.745771	0.553295	2.40E-15	9.52E-13
c168957_g4	7.025973	-0.97008	4.90E-07	4.07E-05
c168970_g1	6.751882	-1.18054	8.20E-06	0.000493
c168987_g1	6.811255	-1.13611	4.60E-06	0.000298
c168991_g1	12.68499	4.415196	2.52E-53	3.35E-49

c168998_g1	8.422999	0.251307	1.70E-13	5.02E-11
c169013_g1	7.568838	-0.50804	1.00E-08	1.22E-06
c169017_g1	7.287591	-0.75656	6.50E-08	6.75E-06
c169038_g1	9.377409	1.159321	1.27E-17	6.64E-15
c169041_g18	-7.07785	-0.88153	1.42E-06	0.000104
c169047_g1	7.618502	-0.47063	2.95E-09	4.00E-07
c169053_g1	9.467626	1.243785	2.25E-19	1.69E-16
c169053_g2	6.833243	-1.1255	1.95E-06	0.000138
c169056_g2	8.504675	2.519975	6.69E-30	2.37E-26
c169071_g1	8.892035	0.706362	4.27E-12	9.87E-10
c169072_g1	7.025973	-0.97008	4.90E-07	4.07E-05
c169074_g1	7.341085	-0.70532	5.18E-08	5.52E-06
c169075_g1	10.25823	2.011605	2.87E-26	5.86E-23
c169076_g1	7.019447	-0.97348	5.58E-07	4.56E-05
c169077_g1	6.851855	-1.09992	5.13E-06	0.000328
c169078_g1	6.70748	-1.21905	7.56E-06	0.000459
c169081_g1	7.0554	-0.9413	5.50E-07	4.51E-05
c169084_g1	8.213854	0.055076	4.33E-12	9.92E-10
c169085_g1	8.360136	0.19981	3.11E-12	7.40E-10
c169090_g1	9.231185	1.018763	5.72E-17	2.76E-14
c169100_g1	7.56025	-0.52221	4.88E-09	6.31E-07
c169108_g1	7.934656	-0.19531	8.22E-11	1.47E-08
c169108_g3	7.270268	-0.76621	8.26E-08	8.38E-06
c169109_g1	8.657956	0.471217	8.57E-15	3.02E-12
c169112_g1	7.220769	-0.80416	1.81E-07	1.69E-05
c169117_g1	7.96141	-0.17188	6.56E-11	1.20E-08
c169126_g1	8.290508	0.147012	5.00E-10	7.88E-08
c169127_g1	7.293334	-0.75334	7.09E-08	7.28E-06
c169130_g1	7.519939	-0.55477	8.16E-09	1.01E-06
c169141_g1	8.735639	0.540507	2.87E-15	1.11E-12
c169142_g1	6.851854	-1.09992	5.14E-06	0.000328
c169153_g2	6.699077	-1.22266	1.02E-05	0.00059
c169162_g1	8.102982	-0.03386	6.92E-11	1.26E-08
c169163_g1	9.992476	1.752272	1.15E-23	1.45E-20
c169163_g2	8.711907	0.519909	3.41E-15	1.30E-12
c169174_g2	3.539642	0.852099	2.01E-09	2.86E-07
c169183_g1	7.162575	-0.85953	1.78E-07	1.66E-05
c169201_g1	8.258777	0.099593	1.64E-12	4.13E-10
c169208_g1	7.514633	-0.55779	1.02E-08	1.24E-06
c169218_g1	7.306904	-0.72108	5.78E-07	4.71E-05
c169231_g2	6.471658	-1.40131	1.77E-05	0.000959
c169232_g1	9.854991	3.856489	1.14E-43	8.67E-40
c169235_g1	6.586675	-1.3109	1.65E-05	0.000903
c169240_g1	7.53043	-0.52583	3.32E-07	2.92E-05
c169263_g2	7.334587	-0.7195	4.10E-08	4.53E-06
c169268_g1	8.057927	-0.08029	2.06E-11	4.15E-09
c169281_g1	6.833243	-1.1255	1.96E-06	0.000138
c169282_g1	8.027337	-0.12498	8.08E-10	1.24E-07
c169284_g1	7.53016	-0.54873	6.47E-09	8.11E-07
c169289_g1	7.535186	-0.54571	4.86E-09	6.30E-07
c169305_g2	10.24467	1.996565	5.79E-27	1.28E-23
c169306_g1	7.135097	-0.88657	3.19E-07	2.84E-05
c169306_g2	8.161705	-0.0019	1.44E-10	2.43E-08
c169314_g1	7.478215	-0.58795	1.65E-08	1.96E-06

c169315_g1	10.06156	1.816419	5.39E-25	9.56E-22
c169317_g1	7.909609	-0.21145	1.99E-10	3.29E-08
c169323_g1	6.760379	-1.17697	5.86E-06	0.000366
c169325_g1	7.089476	-0.90994	8.85E-07	6.86E-05
c169328_g1	7.460016	-0.59708	7.14E-08	7.32E-06
c169336_g1	7.245155	-0.79119	9.12E-08	9.20E-06
c169337_g1	7.90516	-0.21414	2.72E-10	4.45E-08
c169341_g1	7.938781	-0.19262	9.20E-11	1.62E-08
c169344_g1	7.414455	-0.63098	2.38E-07	2.15E-05
c169346_g1	7.363572	-0.69269	3.41E-08	3.82E-06
c169352_g1	6.682851	-1.24782	8.13E-06	0.00049
c169364_g1	6.542313	-1.34974	1.20E-05	0.000689
c169374_g1	7.838678	-0.26787	2.36E-09	3.29E-07
c169375_g1	8.078502	-0.05504	1.17E-10	2.00E-08
c169376_g1	7.055396	-0.9413	5.51E-07	4.51E-05
c169381_g1	7.162575	-0.85953	1.78E-07	1.66E-05
c169384_g1	10.12859	1.88344	6.65E-26	1.31E-22
c169385_g1	8.07323	-0.07001	1.50E-11	3.12E-09
c169386_g1	6.659985	-1.25879	5.87E-06	0.000366
c169386_g2	7.466384	-0.59404	4.52E-08	4.92E-06
c169387_g1	7.419714	-0.64079	2.14E-08	2.47E-06
c169390_g1	7.18282	-0.83642	2.26E-07	2.06E-05
c169394_g1	6.875424	-1.08942	1.46E-06	0.000107
c169398_g1	8.273445	0.114074	1.38E-12	3.52E-10
c169404_g1	7.328883	-0.72269	4.81E-08	5.16E-06
c169405_g1	8.403597	0.237714	8.61E-13	2.32E-10
c169408_g1	11.24897	2.985114	1.37E-30	6.61E-27
c169421_g1	8.280244	0.118854	1.01E-12	2.67E-10
c169422_g2	6.652377	-1.26244	6.79E-06	0.000419
c169427_g1	8.1531	0.015715	1.97E-10	3.27E-08
c169430_g2	7.959323	-0.16733	1.13E-10	1.94E-08
c169433_g1	10.34031	2.092364	1.10E-26	2.34E-23
c169439_g1	8.135042	-0.00796	2.09E-11	4.18E-09
c169443_g1	6.936208	-1.04395	9.38E-07	7.20E-05
c169449_g2	7.068729	-0.93456	3.58E-07	3.09E-05
c169460_g5	8.760192	0.576338	3.86E-13	1.07E-10
c169471_g1	7.250966	-0.78794	9.36E-08	9.40E-06
c169477_g1	6.819051	-1.13258	2.47E-06	0.00017
c169485_g1	6.855154	-1.11486	3.20E-06	0.000215
c169487_g1	8.62466	0.437826	1.15E-14	3.98E-12
c169493_g1	7.419713	-0.64079	2.14E-08	2.47E-06
c169495_g1	6.889015	-1.0824	1.35E-06	0.0001
c169495_g2	8.473802	0.298523	9.05E-14	2.76E-11
c169499_g1	7.116387	-0.89659	2.44E-07	2.21E-05
c169501_g9	3.152843	2.784596	3.58E-11	6.81E-09
c169504_g1	6.471659	-1.40131	1.77E-05	0.000959
c169516_g1	8.301409	0.137879	8.15E-13	2.22E-10
c169519_g1	7.306901	-0.72108	5.79E-07	4.71E-05
c169537_g1	6.707483	-1.21905	7.55E-06	0.000459
c169539_g1	6.534684	-1.35347	1.24E-05	0.000708
c169563_g1	8.036869	-0.09965	2.56E-11	5.01E-09
c169565_g1	7.603203	-0.47946	6.07E-09	7.63E-07
c169568_g2	8.315037	0.16101	1.62E-11	3.34E-09
c169570_g1	6.975254	-1.00996	7.41E-07	5.84E-05

c169571_g1	8.400345	0.23142	3.08E-13	8.76E-11
c169573_g18	8.889759	0.69181	1.17E-15	4.82E-13
c169573_g5	6.961146	-1.01683	1.62E-06	0.000118
c169573_g9	7.813564	1.842517	1.00E-23	1.30E-20
c169576_g1	6.819052	-1.13258	2.47E-06	0.00017
c169579_g1	7.664453	-0.42669	3.01E-09	4.05E-07
c169583_g1	5.826174	-0.02323	1.88E-10	3.13E-08
c169588_g1	8.737766	0.547327	5.35E-15	1.97E-12
c169591_g1	6.805579	-1.15548	5.49E-06	0.000349
c169595_g1	7.055397	-0.9413	5.50E-07	4.51E-05
c169599_g1	8.084718	-0.06231	1.73E-11	3.53E-09
c169616_g1	8.984292	0.781745	4.28E-16	1.84E-13
c169624_g1	7.430458	-0.63459	1.78E-08	2.09E-06
c169625_g1	7.001933	-0.9962	9.73E-07	7.44E-05
c169637_g2	7.875494	-0.246	1.38E-10	2.34E-08
c169652_g1	9.397152	1.17357	7.08E-20	5.98E-17
c169661_g1	6.775353	-1.16982	2.86E-06	0.000194
c169668_g1	6.602433	-1.30352	8.27E-06	0.000494
c169670_g1	7.375125	-0.67585	4.24E-08	4.65E-06
c169672_g1	9.511892	1.288728	1.84E-18	1.14E-15
c169675_g1	7.397418	-0.66334	2.44E-08	2.78E-06
c169677_g1	10.59227	2.33939	1.12E-29	3.71E-26
c169678_g1	7.103677	-0.90327	3.35E-07	2.93E-05
c169682_g1	6.652378	-1.26244	6.78E-06	0.000419
c169691_g1	7.799833	-0.30664	1.06E-09	1.57E-07
c169715_g2	7.328533	-0.71163	1.35E-07	1.29E-05
c169716_g1	6.707481	-1.21905	7.56E-06	0.000459
c169730_g3	8.132892	-0.0196	1.38E-11	2.88E-09
c169731_g7	2.009448	3.60078	6.61E-07	5.30E-05
c169737_g1	6.667318	-1.25513	5.75E-06	0.000363
c169740_g1	7.321713	-0.71478	2.09E-07	1.92E-05
c169744_g2	6.832173	-1.10691	1.42E-05	0.000791
c169746_g1	10.36824	5.205598	1.83E-65	9.70E-61
c169749_g1	6.988337	-1.00308	6.95E-07	5.52E-05
c169751_g1	6.644073	-1.26608	1.28E-05	0.000722
c169754_g2	7.040124	-0.94803	1.64E-06	0.000118
c169755_g1	6.659987	-1.25879	5.86E-06	0.000366
c169759_g1	7.333193	-0.69458	9.22E-07	7.10E-05
c169775_g1	8.057926	-0.08029	2.06E-11	4.15E-09
c169780_g1	7.194958	-0.84297	3.76E-07	3.23E-05
c169782_g1	6.737196	-1.20459	5.00E-06	0.000321
c169783_g2	7.276159	-0.76299	7.00E-08	7.22E-06
c169792_g1	7.005274	-0.98028	1.33E-06	9.86E-05
c169796_g4	2.917551	2.344494	1.73E-11	3.53E-09
c169800_g1	6.922724	-1.05089	1.14E-06	8.58E-05
c169805_g1	7.586043	-0.48828	2.73E-08	3.09E-06
c169806_g1	7.276159	-0.76299	7.00E-08	7.22E-06
c169809_g1	7.853486	-0.25967	5.15E-10	8.10E-08
c169817_g2	10.73533	2.480185	2.88E-31	1.53E-27
c169818_g1	6.83217	-1.10691	1.42E-05	0.000791
c169821_g1	7.025974	-0.97008	4.89E-07	4.07E-05
c169824_g3	9.522681	1.292867	6.12E-21	5.91E-18
c169826_g1	8.584822	0.402313	2.10E-14	6.92E-12
c169828_g1	6.889696	-1.06475	7.06E-06	0.000432

c169834_g1	5.38504	-0.41901	2.44E-08	2.78E-06
c169835_g1	6.936208	-1.04395	9.39E-07	7.20E-05
c169837_g1	6.860395	-1.09642	3.47E-06	0.000231
c169838_g1	8.86006	0.659839	3.41E-16	1.50E-13
c169840_g1	8.10667	-0.03731	1.06E-11	2.27E-09
c169848_g1	7.665742	-0.43387	1.61E-09	2.31E-07
c169849_g1	9.329946	1.105559	3.60E-19	2.48E-16
c169851_g1	7.923506	-0.1959	3.54E-10	5.67E-08
c169870_g2	8.107164	-0.03134	4.60E-11	8.57E-09
c169871_g2	7.466386	-0.59404	4.52E-08	4.92E-06
c169874_g1	9.896405	1.657208	8.11E-24	1.12E-20
c169879_g1	6.586673	-1.3109	1.65E-05	0.000903
c169887_g4	7.93652	-0.18058	1.00E-09	1.50E-07
c169889_g1	7.213887	-0.8074	3.53E-07	3.06E-05
c169889_g2	7.674384	-0.4209	1.78E-09	2.55E-07
c169891_g1	8.31154	0.144994	1.13E-12	2.98E-10
c169892_g1	6.609933	-1.29983	8.66E-06	0.000512
c169892_g2	8.263025	0.1069	2.34E-12	5.70E-10
c169898_g2	6.479439	-1.39754	1.83E-05	0.000985
c169900_g1	10.59008	2.336604	3.69E-30	1.40E-26
c169909_g1	8.308978	0.15182	3.86E-12	9.00E-10
c169913_g1	7.189305	-0.83315	1.73E-07	1.63E-05
c169916_g1	7.355785	-0.68522	1.83E-07	1.70E-05
c169918_g1	8.432814	0.261969	2.26E-13	6.56E-11
c169920_g1	7.932511	-0.19056	1.72E-10	2.89E-08
c169921_g1	8.055132	-0.06772	2.42E-09	3.35E-07
c169923_g1	7.618502	-0.47063	2.95E-09	4.00E-07
c169923_g2	7.048068	-0.94467	1.06E-06	8.04E-05
c169927_g1	6.534682	-1.35347	1.25E-05	0.000708
c169927_g2	7.584715	-0.49915	4.31E-09	5.63E-07
c169928_g1	7.835685	-0.28442	2.98E-10	4.83E-08
c169931_g1	7.055398	-0.9413	5.50E-07	4.51E-05
c169934_g2	7.121784	-0.87935	1.18E-06	8.84E-05
c169935_g1	7.227249	-0.80092	1.33E-07	1.29E-05
c169937_g1	7.947192	-0.18725	1.06E-10	1.85E-08
c169939_g1	9.141644	0.925907	5.11E-18	3.02E-15
c169939_g2	9.896679	1.656194	5.27E-24	7.78E-21
c169947_g1	8.587695	0.404437	1.89E-14	6.31E-12
c169948_g1	7.16076	-0.84623	1.05E-06	7.94E-05
c169954_g9	3.340977	0.439864	2.03E-08	2.36E-06
c169961_g1	7.068729	-0.93456	3.58E-07	3.09E-05
c169962_g1	6.534682	-1.35347	1.25E-05	0.000708
c169970_g1	7.407059	-0.63405	4.18E-07	3.58E-05
c169971_g2	8.027219	-0.11145	2.65E-11	5.17E-09
c169972_g2	7.20655	-0.81064	5.49E-07	4.51E-05
c169978_g1	7.936831	-0.18789	1.30E-10	2.21E-08
c169979_g2	6.842447	-1.10341	8.28E-06	0.000494
c169986_g9	2.768004	4.621875	1.26E-14	4.29E-12
c169993_g1	7.718133	-0.37859	2.28E-09	3.19E-07
c170004_g1	12.71691	4.447446	3.83E-55	6.78E-51
c170009_g2	9.571819	1.345687	2.60E-19	1.85E-16
c170009_g3	7.579609	-0.50211	5.34E-09	6.79E-07
c170015_g1	8.204134	0.05301	4.82E-12	1.09E-09
c170017_g1	7.550107	-0.52819	6.54E-09	8.18E-07

c170018_g7	2.597475	2.56326	1.28E-07	1.24E-05
c170022_g6	6.550093	-1.34601	1.34E-05	0.000749
c170024_g1	9.440647	1.218064	4.33E-19	2.95E-16
c170025_g1	7.189306	-0.83315	1.73E-07	1.63E-05
c170027_g12	2.03484	5.555421	1.08E-07	1.06E-05
c170028_g1	9.249663	1.033062	2.19E-18	1.32E-15
c170030_g3	7.298177	-0.76518	5.51E-07	4.51E-05
c170035_g4	7.189304	-0.83315	1.73E-07	1.63E-05
c170040_g2	8.393199	0.230908	2.78E-12	6.66E-10
c170047_g2	8.146547	0.005191	4.10E-11	7.75E-09
c170051_g1	7.890956	-0.22222	1.18E-09	1.74E-07
c170052_g1	7.312013	-0.73225	5.26E-08	5.58E-06
c170059_g1	7.457488	-0.60949	1.35E-08	1.62E-06
c170059_g2	6.745382	-1.20096	6.61E-06	0.000411
c170069_g3	6.47944	-1.39754	1.83E-05	0.000985
c170070_g1	6.609933	-1.29983	8.66E-06	0.000512
c170070_g2	6.479439	-1.39754	1.83E-05	0.000985
c170077_g1	7.94105	-0.18522	1.03E-10	1.80E-08
c170078_g1	9.136879	0.923662	5.21E-18	3.04E-15
c170079_g1	8.693245	0.50316	4.55E-15	1.70E-12
c170080_g3	7.679149	-0.41801	1.52E-09	2.21E-07
c170081_g1	7.096825	-0.9066	6.10E-07	4.93E-05
c170081_g3	8.173271	0.022539	5.11E-12	1.16E-09
c170090_g1	8.576067	0.395938	4.42E-14	1.40E-11
c170092_g2	8.056179	-0.0867	2.18E-11	4.35E-09
c170095_g1	9.389507	1.162386	2.01E-19	1.52E-16
c170104_g1	9.260152	1.040154	5.88E-19	3.86E-16
c170107_g1	8.41303	0.240533	2.08E-13	6.08E-11
c170107_g2	7.737336	-0.36721	8.55E-10	1.30E-07
c170109_g1	8.813174	0.619483	3.37E-15	1.29E-12
c170111_g1	9.3554	1.130452	1.76E-19	1.36E-16
c170119_g1	7.859987	-0.26231	1.97E-10	3.27E-08
c170126_g1	8.137795	-0.01123	7.68E-12	1.69E-09
c170127_g1	8.71457	0.52193	2.86E-15	1.11E-12
c170132_g1	7.959322	-0.16733	1.13E-10	1.94E-08
c170137_g1	10.52143	2.270593	8.02E-28	2.13E-24
c170146_g1	5.174808	-0.60134	1.63E-07	1.55E-05
c170155_g6	4.714186	1.174035	8.42E-10	1.28E-07
c170157_g1	7.963524	-0.16468	8.84E-11	1.57E-08
c170157_g2	8.259447	0.104508	3.80E-12	8.89E-10
c170165_g1	6.699073	-1.22266	1.02E-05	0.00059
c170181_g1	8.240466	0.082512	2.14E-12	5.23E-10
c170182_g1	10.11539	1.872899	4.87E-24	7.40E-21
c170189_g4	7.081262	-0.92782	3.42E-07	2.98E-05
c170193_g3	6.855156	-1.11486	3.20E-06	0.000215
c170202_g1	5.518618	-0.29118	4.65E-09	6.05E-07
c170216_g2	8.086308	-0.0561	1.29E-11	2.71E-09
c170242_g2	7.363572	-0.69269	3.41E-08	3.82E-06
c170256_g1	3.127604	7.692712	3.38E-12	8.02E-10
c170280_g1	4.696392	1.114791	4.03E-14	1.29E-11
c170365_g1	2.830789	5.780531	1.61E-12	4.07E-10
c170568_g1	-2.27153	3.061125	3.91E-06	0.000259
c170964_g1	2.124365	4.188093	5.85E-09	7.39E-07
c171754_g1	6.348291	1.749756	7.87E-15	2.81E-12

c172779_g1	9.126203	0.910173	9.64E-18	5.23E-15
c175321_g1	3.115212	7.764612	1.30E-22	1.47E-19
c175484_g1	3.015113	4.976241	4.31E-12	9.92E-10
c176080_g1	5.180774	1.961708	2.60E-19	1.85E-16
c176232_g1	6.103943	3.046626	3.36E-24	5.42E-21
c176834_g1	2.690719	3.141972	1.40E-05	0.000783
c1785_g1	7.514632	-0.55779	1.02E-08	1.24E-06
c178600_g1	3.570635	-0.37189	4.56E-06	0.000297
c178719_g1	6.975254	-1.00996	7.41E-07	5.84E-05
c181140_g1	7.584864	1.629941	7.56E-10	1.16E-07
c181204_g1	5.504154	3.343396	3.82E-20	3.38E-17
c181387_g1	7.245155	-0.79119	9.13E-08	9.20E-06
c182311_g1	-3.44006	1.989758	3.59E-06	0.000238
c182811_g1	6.722413	-1.21182	4.01E-06	0.000264
c185603_g1	4.224938	3.210763	5.43E-09	6.89E-07
c185786_g1	2.324269	4.529303	4.13E-09	5.42E-07
c186391_g1	2.761766	2.00616	1.20E-09	1.76E-07
c186746_g1	3.971282	-0.39315	7.24E-06	0.000442
c186760_g1	-2.77697	0.809589	1.96E-06	0.000138
c187029_g1	2.105395	3.631215	1.63E-08	1.94E-06
c187141_g1	8.88635	2.907215	1.80E-32	1.06E-28
c187273_g1	2.669066	3.07329	2.36E-11	4.65E-09
c190719_g2	2.115288	7.291592	2.39E-09	3.32E-07
c190821_g1	2.013277	6.264671	7.24E-10	1.12E-07
c190886_g1	2.346388	5.22552	1.72E-12	4.28E-10
c190910_g1	4.685126	4.84308	2.71E-30	1.11E-26
c191047_g1	2.196256	3.135713	3.78E-08	4.22E-06
c191395_g1	7.927344	1.951148	1.23E-24	2.04E-21
c191491_g1	4.502103	4.506747	1.56E-15	6.39E-13
c191940_g1	5.798099	1.60601	1.34E-19	1.07E-16
c192079_g1	7.938781	-0.19262	9.20E-11	1.62E-08
c192609_g1	7.276079	-0.77494	1.84E-07	1.72E-05
c192811_g1	2.921957	3.511347	3.67E-13	1.03E-10
c193104_g1	2.814964	1.639112	1.58E-08	1.89E-06
c193965_g1	7.868679	-0.2568	2.71E-10	4.45E-08
c195954_g1	2.573175	5.1619	1.93E-08	2.25E-06
c196047_g1	5.925853	3.803883	1.64E-18	1.03E-15
c196163_g1	2.798504	5.692381	2.90E-16	1.28E-13
c196502_g1	3.843333	6.315257	7.31E-25	1.25E-21
c196504_g1	3.137471	5.086127	6.65E-13	1.82E-10
c197065_g1	8.483217	0.308776	1.22E-13	3.67E-11
c197082_g1	3.053744	1.160858	7.66E-08	7.84E-06
c23797_g1	3.114553	2.813712	5.43E-12	1.22E-09
c25409_g1	4.78281	0.308309	1.61E-10	2.71E-08
c25648_g1	4.127412	3.59619	1.29E-11	2.71E-09
c26450_g1	6.192133	2.930047	3.19E-29	9.41E-26
c27266_g1	2.953552	1.378244	4.91E-09	6.34E-07
c27266_g2	2.44985	2.167989	4.76E-08	5.12E-06
c27310_g1	11.08458	2.824905	6.28E-35	4.17E-31
c30023_g1	2.303574	4.235637	6.89E-07	5.48E-05
c31398_g1	4.564007	0.452336	1.68E-11	3.44E-09
c34030_g1	6.47944	-1.39754	1.83E-05	0.000985
c34762_g1	3.444146	3.988796	6.33E-12	1.41E-09
c39320_g1	10.55503	2.295138	4.63E-21	4.55E-18

c39757_g1	2.808585	7.093242	8.27E-12	1.81E-09
c40173_g1	2.161026	7.677194	1.24E-11	2.62E-09
c42101_g1	2.860893	4.025579	3.57E-14	1.16E-11
c42235_g1	2.719172	2.832658	1.14E-08	1.37E-06
c42648_g1	-3.9076	2.314872	1.66E-07	1.58E-05
c42787_g1	2.990497	3.515235	2.36E-13	6.82E-11
c42811_g1	10.21022	1.955421	1.31E-19	1.05E-16
c43818_g1	2.163634	3.77747	6.81E-07	5.45E-05
c44528_g1	7.089474	-0.90994	8.86E-07	6.86E-05
c45337_g1	8.464237	0.288167	1.07E-13	3.26E-11
c45740_g1	-3.84521	1.092275	3.01E-06	0.000203
c46769_g1	7.23263	-0.8102	2.22E-07	2.03E-05
c48500_g1	6.586675	-1.3109	1.65E-05	0.000903
c48518_g2	4.271748	2.691625	1.32E-12	3.41E-10
c48865_g1	3.308337	5.325284	1.02E-09	1.53E-07
c49021_g1	-4.28744	1.766414	3.31E-09	4.43E-07
c49705_g1	2.314878	1.721961	9.67E-06	0.000566
c63698_g1	2.170049	4.267161	9.30E-09	1.14E-06
c64947_g1	2.686745	5.645614	8.80E-07	6.86E-05
c65107_g1	4.836	-0.8844	4.71E-06	0.000305
c66040_g1	8.646985	0.457387	9.15E-15	3.20E-12
c66164_g1	4.480924	2.630093	1.36E-13	4.03E-11
c66164_g2	4.741658	2.799134	4.44E-15	1.67E-12
c66412_g1	2.496494	4.891457	6.42E-10	9.97E-08
c66521_g1	3.356284	1.409522	1.74E-10	2.91E-08
c66745_g1	7.219535	-0.81676	1.25E-07	1.21E-05
c68143_g1	3.24409	3.16855	7.77E-15	2.79E-12
c68169_g1	3.907817	3.532099	7.66E-11	1.38E-08
c68182_g1	3.371234	5.812243	1.52E-23	1.88E-20
c68182_g2	2.349386	4.886706	2.69E-11	5.23E-09
c71527_g1	3.336325	0.845065	3.25E-08	3.66E-06
c73522_g1	3.227532	0.481275	1.10E-07	1.07E-05
c77082_g1	2.314601	2.494576	1.56E-08	1.86E-06
c84395_g1	3.145597	1.840853	3.79E-07	3.25E-05
c84912_g2	3.910512	0.200098	8.91E-08	9.02E-06
c89444_g1	7.665742	-0.43387	1.61E-09	2.31E-07
c91879_g1	2.954919	3.640357	9.29E-13	2.49E-10

28 DAI

ID	logFC	logCPM	PValue	FDR
c10105_g1	-2.25673	0.17103	1.08E-05	0.000180515
c10276_g1	2.791756	2.033318	2.95E-16	2.74E-14
c102953_g1	6.380978	-1.37772	1.92E-05	0.000303523
c105238_g1	3.234945	-0.92355	2.96E-05	0.00044803
c105379_g1	6.39313	-1.37539	6.64E-05	0.000919401
c105606_g1	2.963706	2.979859	7.55E-09	2.39E-07
c106589_g1	-2.44767	0.631211	4.20E-07	9.53E-06
c10902_g1	6.782056	-1.08213	2.44E-06	4.73E-05
c109400_g1	-3.2502	1.089802	7.75E-13	4.67E-11
c111101_g1	6.463165	-1.32266	2.99E-05	0.00045029
c113156_g1	6.305477	-1.43244	3.53E-05	0.000522258
c113207_g1	-2.57217	-0.16146	4.44E-05	0.000642298
c113299_g1	6.225961	-1.4893	6.83E-05	0.000939196
c113463_g1	6.782056	-1.08213	2.44E-06	4.73E-05
c113541_g1	7.790106	-0.23434	4.53E-12	2.41E-10
c113614_g1	6.586361	-1.22492	4.42E-06	8.16E-05
c113835_g1	2.214724	2.957029	4.77E-15	3.83E-13
c113949_g1	-2.39156	1.515529	4.46E-10	1.75E-08
c114004_g2	-2.64364	1.560621	6.26E-12	3.26E-10
c114351_g2	-3.4205	1.637299	2.40E-13	1.54E-11
c114421_g2	3.296059	2.572697	7.55E-19	9.49E-17
c114534_g1	2.997983	4.27341	8.74E-12	4.45E-10
c114534_g2	2.843078	-0.08459	1.99E-06	3.94E-05
c114696_g1	6.530367	-1.27178	1.48E-05	0.000240602
c114884_g1	9.609461	1.472247	1.07E-27	3.63E-25
c114899_g1	-2.8299	5.81013	1.32E-38	1.26E-35
c115091_g1	2.279917	7.436529	1.50E-26	4.52E-24
c115091_g2	2.191863	0.423531	3.55E-06	6.70E-05
c115636_g1	2.462137	4.136235	2.13E-17	2.32E-15
c115788_g1	-2.26664	3.386167	2.26E-10	9.23E-09
c116224_g1	2.536206	4.064774	8.89E-20	1.24E-17
c116639_g1	6.463167	-1.32266	2.99E-05	0.00045029
c116710_g1	3.964293	3.355973	4.80E-22	8.87E-20
c117343_g1	-2.12942	4.078796	2.00E-11	9.61E-10
c117571_g1	2.809013	1.233584	1.91E-11	9.23E-10
c117714_g2	2.446677	1.419767	5.45E-11	2.44E-09
c118031_g1	-2.39103	5.258791	1.10E-20	1.72E-18
c118034_g1	6.450213	-1.32576	1.07E-05	0.000180232
c118055_g1	3.017036	3.296703	4.70E-23	1.00E-20
c118147_g2	2.746937	4.015077	9.01E-26	2.53E-23
c118162_g2	2.039263	1.137955	2.36E-07	5.61E-06
c118260_g1	3.654502	0.237907	2.13E-09	7.47E-08
c118265_g1	5.512689	4.711171	1.41E-61	6.61E-58
c118347_g2	6.45894	-1.32344	1.84E-05	0.000292425
c118439_g1	3.251806	1.923568	1.19E-19	1.62E-17
c118485_g1	2.478841	2.340749	2.93E-14	2.12E-12
c118617_g1	6.523751	-1.27332	7.53E-06	0.000131459
c118780_g2	2.345135	5.1894	2.82E-17	3.01E-15
c118814_g1	2.869525	4.495662	2.45E-19	3.22E-17
c12024_g1	-2.14283	4.857656	3.29E-10	1.31E-08
c120606_g2	2.052563	0.392432	2.61E-05	0.000401112
c120606_g3	2.498772	5.713077	1.20E-23	2.68E-21
c120737_g1	2.360333	4.801957	7.12E-25	1.83E-22

c121181_g1	6.920804	-0.96114	8.96E-07	1.90E-05
c121360_g1	6.891367	-0.99604	8.86E-07	1.88E-05
c12180_g1	2.182808	6.130351	7.18E-08	1.89E-06
c121826_g1	2.886175	6.881108	1.62E-44	2.90E-41
c121880_g1	2.421455	6.739064	3.84E-06	7.20E-05
c121922_g1	6.530368	-1.27178	1.48E-05	0.000240602
c122022_g3	7.293801	-0.66337	4.42E-09	1.47E-07
c122156_g1	6.651148	-1.17659	2.83E-06	5.43E-05
c122210_g1	-2.14279	1.107678	4.85E-07	1.08E-05
c122437_g1	6.899208	-0.99455	3.08E-06	5.86E-05
c122451_g2	3.706232	1.703212	2.26E-11	1.08E-09
c122467_g1	-2.20278	1.963936	1.49E-07	3.68E-06
c122545_g1	2.003001	4.979516	2.92E-16	2.71E-14
c122610_g1	6.975959	-0.91989	1.78E-07	4.34E-06
c122745_g3	2.955317	0.31167	9.46E-09	2.93E-07
c122754_g1	5.454965	3.268259	6.92E-09	2.21E-07
c122767_g1	2.82874	1.661649	5.92E-12	3.10E-10
c123021_g1	-2.68276	-0.24187	5.84E-05	0.000814034
c123108_g1	-2.14904	2.163474	6.74E-09	2.16E-07
c123139_g1	3.717493	2.548966	2.75E-19	3.58E-17
c123324_g1	6.944811	-0.95445	7.23E-07	1.56E-05
c123374_g1	6.710799	-1.13054	1.75E-06	3.52E-05
c123443_g1	8.118882	0.059585	1.19E-14	9.15E-13
c123554_g2	3.847086	-0.44514	2.03E-06	4.00E-05
c123560_g1	-2.07652	8.470129	6.59E-16	5.86E-14
c123765_g1	6.782055	-1.08213	2.43E-06	4.73E-05
c123818_g1	7.137873	-0.79944	1.89E-07	4.61E-06
c123948_g1	7.224047	-0.72797	7.62E-08	1.99E-06
c124094_g1	2.672326	-0.54046	6.62E-05	0.000916425
c124117_g2	3.032524	2.419312	7.56E-08	1.98E-06
c124137_g1	2.482694	-0.08749	4.54E-05	0.000655849
c124169_g1	6.891363	-0.99604	8.84E-07	1.88E-05
c124171_g2	6.775441	-1.08364	9.83E-07	2.07E-05
c124340_g1	6.521152	-1.27409	6.90E-06	0.000121585
c124410_g1	6.659823	-1.17431	3.91E-06	7.31E-05
c124530_g1	6.820348	-1.04334	1.01E-06	2.12E-05
c124782_g1	8.175156	0.092178	1.63E-09	5.80E-08
c125081_g1	2.037598	0.581597	2.62E-06	5.05E-05
c125150_g1	9.515238	1.377806	1.218E-23	2.71473E-21
c125231_g1	2.307424	4.732441	5.13E-09	1.69E-07
c125630_g1	3.408889	1.502846	1.12E-08	3.41E-07
c125655_g1	6.375582	-1.37927	1.95E-05	0.000307722
c126086_g1	3.481331	-0.73488	3.48E-06	6.56E-05
c126171_g1	10.47625	2.327891	1.46298E-37	1.26082E-34
c126490_g1	2.013267	7.301783	1.78E-06	3.56E-05
c126600_g2	7.958684	-0.08798	7.36E-13	4.47E-11
c126644_g1	6.458941	-1.32344	1.84E-05	0.000292425
c126722_g1	2.725553	3.187583	7.65E-21	1.22E-18
c126878_g1	2.281131	5.766713	1.45E-15	1.23E-13
c127554_g1	7.34576	-0.62649	1.88E-08	5.49E-07
c127683_g2	2.181574	0.781	3.59E-05	0.000530469
c128577_g1	5.74566	-0.04616	2.43E-13	1.56E-11
c129225_g1	2.058712	3.044355	1.50E-10	6.33E-09
c129613_g1	6.526728	-1.27255	9.79E-06	0.000166512

c129764_g1	2.500798	4.180791	1.34E-16	1.31E-14
c130034_g1	4.678553	-0.21894	8.68E-09	2.71E-07
c130687_g2	-2.40243	1.611888	6.56E-09	2.11E-07
c130687_g3	-2.22316	2.168469	1.37E-10	5.81E-09
c130794_g1	9.615163	1.47862	4.94E-29	1.95E-26
c130909_g1	7.589111	-0.41545	3.44E-10	1.37E-08
c13099_g1	-2.57201	1.132098	8.02E-09	2.51E-07
c131077_g1	-7.78866	-0.04474	8.80E-06	0.000150935
c131236_g1	4.481548	0.431752	6.35E-14	4.36E-12
c131390_g1	3.406308	6.563138	1.19E-45	2.45E-42
c131771_g2	2.140759	5.141056	2.47E-21	4.18E-19
c131923_g1	-7.27205	-0.49523	1.23E-05	0.000204504
c131923_g2	-8.40169	0.501966	7.82E-06	0.000135652
c132145_g1	6.782057	-1.08213	2.44E-06	4.73E-05
c132215_g1	9.157081	1.033134	1.78E-23	3.91E-21
c132278_g1	6.38415	-1.37694	2.52E-05	0.000389277
c132307_g1	6.292844	-1.43556	5.57E-05	0.000779172
c132727_g1	8.238533	0.163689	5.56E-14	3.85E-12
c133101_g1	6.530367	-1.27178	1.48E-05	0.000240602
c133258_g1	10.12858	1.985459	3.23E-39	3.48E-36
c133321_g2	3.262762	3.167999	1.17E-19	1.60E-17
c133407_g1	3.577347	-1.09092	3.67E-05	0.000541688
c133497_g1	6.222518	-1.49009	5.10E-05	0.000724089
c134084_g1	7.753891	-0.29645	2.61E-06	5.05E-05
c134231_g1	6.45554	-1.32421	1.29E-05	0.00021358
c134292_g1	6.594707	-1.22262	7.39E-06	0.000129303
c134858_g1	-2.0809	8.521005	3.12E-24	7.47E-22
c135045_g1	7.702927	-0.29379	5.45E-08	1.47E-06
c135596_g1	2.835679	0.74671	2.17E-10	8.91E-09
c135893_g2	2.442311	3.276127	1.66E-16	1.60E-14
c135909_g1	6.722142	-1.12751	2.99E-06	5.71E-05
c135918_g1	2.189487	4.51914	3.30E-06	6.26E-05
c135970_g1	7.643983	-0.36347	3.60E-11	1.66E-09
c135971_g1	2.578364	0.924486	1.90E-09	6.70E-08
c136046_g1	3.062913	0.517462	3.89E-10	1.54E-08
c136133_g1	2.180147	7.515954	2.40E-29	9.86E-27
c136323_g1	-2.12069	8.88808	2.95E-08	8.32E-07
c136361_g1	2.491457	-0.03993	2.69E-05	0.000412494
c136372_g2	2.988508	-0.5087	2.86E-05	0.000435316
c136419_g1	6.969765	-0.92137	7.26E-07	1.56E-05
c136792_g1	2.397937	0.226768	1.03E-05	0.000174624
c136898_g1	2.64037	2.242169	1.35E-14	1.02E-12
c136978_g1	2.417384	0.322783	4.44E-07	1.00E-05
c137245_g1	6.380976	-1.37772	1.92E-05	0.000303523
c137475_g1	3.337216	1.493547	7.34E-12	3.78E-10
c137475_g2	3.503207	2.730058	1.76E-22	3.48E-20
c137531_g1	2.222694	5.729501	7.24E-13	4.40E-11
c137843_g1	2.258215	6.154386	3.88E-11	1.78E-09
c137949_g1	2.199273	3.083001	7.13E-12	3.67E-10
c138476_g1	-8.08574	0.217255	5.46E-06	9.84E-05
c138860_g1	8.256803	0.181742	1.30E-14	9.87E-13
c139508_g1	6.673366	-1.17202	2.36E-05	0.000366239
c139702_g1	2.015297	2.878643	5.80E-05	0.000808872
c139890_g1	2.399122	4.567079	1.05E-19	1.45E-17

c139890_g3	2.69253	4.013839	1.24E-13	8.30E-12
c140586_g3	2.537714	4.813835	6.65E-22	1.21E-19
c140775_g1	6.591546	-1.22339	5.28E-06	9.56E-05
c141742_g1	3.201334	1.988745	3.05E-13	1.93E-11
c141817_g1	6.305478	-1.43244	3.53E-05	0.000522258
c142316_g1	-2.16391	0.311668	1.28E-05	0.000212774
c142453_g1	2.070821	6.883618	9.11E-18	1.03E-15
c142651_g1	6.523751	-1.27332	7.54E-06	0.000131459
c142673_g1	3.358475	1.215018	1.31E-12	7.60E-11
c142819_g3	-2.07018	3.198326	9.40E-11	4.07E-09
c142939_g2	2.955246	0.912202	3.94E-10	1.56E-08
c143214_g1	-6.28384	0.765566	1.50E-06	3.05E-05
c143270_g1	7.078436	-0.84056	3.73E-08	1.04E-06
c143554_g1	2.301876	0.387637	1.52E-05	0.000245424
c143554_g2	2.947336	2.141336	5.30E-09	1.73E-07
c143650_g2	-3.87461	-0.6015	5.76E-06	0.000103407
c143678_g1	2.406516	1.373607	1.37E-08	4.10E-07
c143741_g1	7.574885	1.680038	4.72E-34	3.01E-31
c143890_g2	5.646076	1.151139	7.29E-16	6.44E-14
c144226_g1	3.936063	0.692658	3.05E-14	2.20E-12
c144243_g2	-2.65816	0.231472	6.29E-07	1.38E-05
c144256_g3	2.427183	1.989686	2.04E-13	1.33E-11
c144290_g1	2.930046	4.150598	5.42E-22	9.97E-20
c144461_g1	7.131834	-0.8009	6.08E-08	1.63E-06
c144662_g2	6.518551	-1.27486	7.57E-06	0.000131568
c144797_g1	2.315965	4.159099	3.41E-18	4.06E-16
c144904_g2	2.850354	8.014243	2.28E-17	2.47E-15
c145087_g2	2.661129	2.767504	3.34E-09	1.13E-07
c145361_g1	3.195368	3.622137	3.13E-19	4.06E-17
c145441_g1	7.087943	-0.83762	1.22E-07	3.08E-06
c145581_g1	3.288421	5.60629	7.42E-17	7.54E-15
c145770_g2	3.203046	0.224832	3.70E-09	1.24E-07
c145903_g1	2.673118	-0.34613	2.37E-05	0.000367524
c145972_g2	-2.44603	2.350363	2.24E-14	1.65E-12
c146068_g1	2.82576	1.54581	4.05E-14	2.87E-12
c146172_g4	6.447409	-1.32653	1.31E-05	0.000216236
c146257_g2	-2.64137	0.469186	4.78E-08	1.30E-06
c146349_g1	2.803509	3.788891	1.24E-14	9.43E-13
c146380_g1	3.061133	3.673284	7.52E-10	2.84E-08
c146380_g2	3.486744	2.979811	1.02E-14	7.88E-13
c146576_g1	6.659825	-1.17431	3.91E-06	7.31E-05
c146621_g1	9.148012	1.027261	4.24E-25	1.11E-22
c146638_g1	6.375581	-1.37927	1.96E-05	0.000307754
c146670_g3	9.403461	1.276121	2.85E-27	9.38E-25
c146694_g1	7.177126	-0.76436	5.05E-08	1.37E-06
c146711_g1	3.38338	1.249521	2.61E-12	1.45E-10
c146795_g3	6.450212	-1.32576	1.07E-05	0.000180255
c146875_g4	4.66534	1.525508	9.98E-23	2.02E-20
c146875_g6	5.831284	2.007857	2.21E-27	7.38E-25
c146879_g1	2.283213	4.986341	1.02E-05	0.000172055
c147016_g1	-2.49696	0.455554	4.60E-06	8.43E-05
c147061_g1	6.30252	-1.43322	2.91E-05	0.000441924
c147108_g1	7.587072	-0.41614	2.12E-10	8.76E-09
c147302_g1	6.378291	-1.3785	1.75E-05	0.000280861

c147475_g1	6.78206	-1.08213	2.44E-06	4.74E-05
c147648_g1	2.269354	1.371418	1.19E-09	4.35E-08
c147690_g1	9.267617	1.138253	5.13E-23	1.08E-20
c147691_g2	2.674984	2.186543	1.67E-14	1.24E-12
c147710_g1	8.584635	0.487335	9.03E-18	1.02E-15
c147819_g1	2.70727	-0.03052	1.05E-05	0.000177676
c147836_g1	-2.50503	-0.20737	5.67E-05	0.000792112
c147979_g1	7.216019	-0.73014	1.67E-08	4.91E-07
c147987_g2	3.039767	-0.07484	2.25E-07	5.36E-06
c148067_g1	6.305477	-1.43244	3.53E-05	0.000522258
c148117_g1	2.024281	1.606437	3.06E-08	8.62E-07
c148158_g1	6.775441	-1.08364	9.83E-07	2.07E-05
c148189_g1	8.536785	0.441465	8.30E-17	8.38E-15
c148354_g1	6.88529	-0.99754	3.64E-07	8.35E-06
c148469_g2	6.30924	-1.43166	5.11E-05	0.000724427
c148850_g1	7.091079	-0.83689	2.18E-07	5.21E-06
c149183_g3	4.682182	1.545082	2.22E-20	3.37E-18
c149234_g2	2.430425	6.942938	3.23E-21	5.41E-19
c149319_g1	-2.10498	1.867469	6.28E-10	2.40E-08
c149374_g1	6.299841	-1.434	2.96E-05	0.00044803
c149388_g2	8.544575	0.488752	4.05E-05	0.000588653
c149402_g3	2.690951	2.185971	6.80E-13	4.16E-11
c149473_g1	6.762452	-1.08741	2.05E-06	4.04E-05
c149487_g1	6.944808	-0.95445	7.21E-07	1.56E-05
c149565_g1	2.736457	8.548679	2.11E-30	9.57E-28
c149600_g1	6.219734	-1.49087	4.66E-05	0.000671041
c149641_g1	3.010521	6.133673	2.72E-25	7.41E-23
c149663_g1	2.322398	3.31455	6.39E-17	6.57E-15
c150161_g1	6.530367	-1.27178	1.48E-05	0.000240602
c150185_g2	6.990416	1.940946	3.92E-36	3.17E-33
c150233_g4	-2.0293	2.348942	2.96E-10	1.19E-08
c150251_g1	7.037059	-0.87677	1.01E-07	2.59E-06
c150264_g3	3.281785	0.11701	5.87E-08	1.58E-06
c150264_g5	4.592747	-1.00077	4.54E-06	8.33E-05
c150291_g1	2.909941	0.515908	5.90E-10	2.26E-08
c150320_g3	3.564644	0.935229	4.83E-12	2.55E-10
c150378_g3	2.256201	3.760428	1.15E-11	5.78E-10
c150389_g1	2.375864	6.670054	2.35E-21	4.00E-19
c150433_g1	6.835988	-1.03883	1.13E-06	2.34E-05
c150550_g1	3.48133	-0.73488	3.48E-06	6.56E-05
c150550_g2	2.198443	5.628503	1.01E-13	6.80E-12
c150613_g1	8.196519	0.124985	1.25E-13	8.32E-12
c150633_g1	-2.29143	0.306369	5.32E-05	0.000750756
c150634_g2	-2.3509	0.125416	1.76E-05	0.000282038
c150647_g1	8.288389	0.21471	3.50E-16	3.22E-14
c150774_g2	2.859664	2.910201	2.30E-12	1.29E-10
c150830_g1	3.509776	-0.70192	1.10E-05	0.000184571
c150982_g2	6.455542	-1.32421	1.29E-05	0.00021358
c151013_g1	7.039766	-0.87603	1.55E-07	3.83E-06
c151038_g1	2.373287	2.163115	8.84E-13	5.30E-11
c151042_g1	7.039765	-0.87603	1.55E-07	3.83E-06
c151100_g1	6.594704	-1.22262	7.38E-06	0.000129244
c151144_g1	9.29487	1.161624	2.91E-20	4.36E-18
c151177_g1	4.881846	-0.74022	4.71E-05	0.00067664

c151209_g1	7.799362	-0.23032	1.32E-11	6.55E-10
c151219_g1	6.305478	-1.43244	3.53E-05	0.000522258
c151233_g1	6.468229	-1.32189	3.68E-05	0.000541871
c151344_g1	11.78233	3.629932	7.60E-39	7.71E-36
c151421_g1	6.77544	-1.08364	9.82E-07	2.07E-05
c151430_g1	6.30252	-1.43322	2.91E-05	0.000441924
c151432_g2	2.00586	2.540238	3.14E-10	1.26E-08
c151696_g4	6.832827	-1.03958	7.17E-07	1.55E-05
c151753_g2	-2.39997	4.903277	2.37E-19	3.12E-17
c151796_g4	2.722501	0.475397	4.80E-08	1.30E-06
c151967_g1	4.302735	2.892132	1.20E-33	6.96E-31
c152001_g1	-2.06991	8.172617	1.20E-08	3.63E-07
c152254_g1	3.578626	3.550659	2.20E-22	4.25E-20
c152269_g1	6.53986	-1.27024	3.20E-05	0.00047879
c152320_g2	3.175495	-0.63161	3.62E-05	0.000533657
c152320_g3	2.256066	1.153594	2.09E-08	6.05E-07
c152423_g1	3.131066	6.830735	1.20E-34	8.41E-32
c152488_g1	6.463166	-1.32266	2.99E-05	0.00045029
c152651_g1	6.653674	-1.17583	3.11E-06	5.91E-05
c152653_g2	7.400188	-0.56979	2.43E-09	8.38E-08
c152678_g1	7.211842	-0.73158	1.04E-08	3.21E-07
c152697_g1	6.591545	-1.22339	5.27E-06	9.56E-05
c152753_g1	2.475225	1.386758	9.57E-08	2.47E-06
c152792_g2	6.305476	-1.43244	3.53E-05	0.000522258
c152826_g2	6.645848	-1.17812	3.89E-06	7.28E-05
c152842_g1	6.825376	-1.04184	4.29E-07	9.71E-06
c152953_g2	6.45894	-1.32344	1.84E-05	0.000292425
c152961_g1	6.651148	-1.17659	2.84E-06	5.43E-05
c153006_g1	6.216872	-1.49166	5.33E-05	0.000750756
c153065_g5	6.526727	-1.27255	9.78E-06	0.000166512
c153077_g1	6.99002	-0.91545	2.05E-07	4.92E-06
c153080_g2	3.561997	2.667837	6.27E-07	1.37E-05
c153088_g1	6.388164	-1.37617	3.87E-05	0.000565886
c153149_g1	3.11117	2.669974	8.64E-20	1.20E-17
c153321_g1	5.287999	-0.44588	1.11E-08	3.39E-07
c153414_g1	2.759149	2.047267	5.20E-15	4.16E-13
c153446_g2	-2.00125	2.928689	6.87E-11	3.03E-09
c153473_g2	3.469966	0.849585	5.86E-12	3.08E-10
c153485_g1	6.375579	-1.37927	1.96E-05	0.00030833
c153505_g1	3.540573	0.131068	1.19E-09	4.35E-08
c153597_g1	2.847655	-0.07453	5.95E-06	0.000106518
c153626_g1	-7.60103	-0.20811	1.30E-05	0.000214285
c153659_g1	8.265445	2.358193	1.31E-42	2.05E-39
c153665_g1	6.219734	-1.49087	4.67E-05	0.000671041
c153788_g1	6.384154	-1.37694	2.53E-05	0.000389681
c153802_g1	-2.44553	3.688286	1.80E-22	3.56E-20
c153802_g4	-2.23575	4.016682	2.68E-15	2.23E-13
c153804_g1	6.778495	-1.08288	1.46E-06	2.97E-05
c153875_g1	2.740667	5.565738	7.04E-27	2.23E-24
c153943_g1	7.211843	-0.73158	1.05E-08	3.21E-07
c153990_g3	-2.36279	0.132852	6.78E-06	0.000120051
c154015_g1	6.903686	-0.9938	6.08E-06	0.000108751
c154125_g1	8.29151	0.205708	6.28E-12	3.27E-10
c154142_g1	8.932602	0.810702	2.01E-16	1.90E-14

c154400_g1	6.225963	-1.4893	6.83E-05	0.000939196
c154431_g1	6.730726	-1.12599	1.00E-05	0.000169212
c154560_g1	7.514961	-0.4758	2.16E-10	8.89E-09
c154648_g1	6.839642	-1.03808	1.97E-06	3.91E-05
c154939_g4	7.252452	-0.69744	6.68E-09	2.14E-07
c154944_g1	7.00927	-0.88413	2.27E-06	4.45E-05
c155059_g1	2.537694	-0.45538	5.47E-05	0.000767049
c155142_g1	6.980741	-0.91841	1.05E-07	2.67E-06
c155178_g1	2.259922	1.891913	3.10E-08	8.72E-07
c155178_g2	2.277695	3.45421	1.85E-16	1.76E-14
c155258_g2	2.580475	3.363515	8.85E-18	1.01E-15
c155258_g3	3.237987	2.14962	1.17E-12	6.85E-11
c155293_g2	3.055996	2.162763	1.32E-19	1.79E-17
c155317_g1	6.645845	-1.17812	3.90E-06	7.29E-05
c155590_g1	2.266215	2.568005	6.99E-10	2.65E-08
c155592_g1	7.078436	-0.84056	3.73E-08	1.04E-06
c155643_g2	2.168102	1.398958	3.09E-08	8.70E-07
c155750_g3	2.332176	3.954525	1.19E-09	4.34E-08
c155775_g1	7.734039	-0.28436	9.84E-12	4.96E-10
c155825_g2	2.284001	3.193164	2.82E-16	2.63E-14
c155938_g1	7.439658	-0.53783	8.93E-10	3.34E-08
c156002_g1	6.598502	-1.22186	7.85E-06	0.00013588
c156063_g1	3.064492	0.260109	1.19E-08	3.61E-07
c156069_g3	-2.95054	0.83121	5.13E-10	1.99E-08
c156069_g6	-2.26887	7.233195	3.30E-28	1.19E-25
c156113_g1	8.956205	0.840276	1.45E-21	2.54E-19
c156192_g1	7.901565	-0.13655	7.71E-13	4.66E-11
c156243_g2	2.144005	0.714253	2.42E-07	5.75E-06
c156270_g1	3.169793	1.354376	5.92E-15	4.69E-13
c156270_g2	3.411218	0.91242	2.19E-10	8.98E-09
c156295_g1	8.063217	2.155446	7.30E-34	4.39E-31
c156341_g1	6.929295	-0.95891	1.82E-07	4.43E-06
c156360_g1	6.384148	-1.37694	2.52E-05	0.000389059
c156390_g1	6.288607	0.449302	2.21E-18	2.68E-16
c156430_g3	3.722811	4.890516	6.82E-21	1.10E-18
c156441_g1	2.140413	0.906581	1.93E-06	3.84E-05
c156472_g1	6.594703	-1.22262	7.37E-06	0.000129212
c156486_g1	8.181825	0.118119	4.36E-15	3.55E-13
c156489_g1	8.301716	0.220826	5.38E-14	3.74E-12
c156525_g1	7.000216	-0.91323	1.16E-06	2.40E-05
c156542_g1	6.443948	-1.3273	1.91E-05	0.000301976
c156553_g1	8.839588	0.72822	4.78E-20	6.94E-18
c156562_g1	7.207966	-0.73303	1.09E-08	3.33E-07
c156637_g1	6.225966	-1.4893	6.84E-05	0.000939589
c156643_g2	8.186601	0.120614	4.50E-15	3.65E-13
c156681_g1	5.254768	0.770061	1.38E-05	0.000226066
c156692_g1	6.726169	-1.12675	5.30E-06	9.58E-05
c156792_g1	8.021081	-0.0389	3.28E-11	1.52E-09
c156838_g1	2.190791	0.556673	1.33E-06	2.73E-05
c156853_g2	-3.66715	-0.28072	6.17E-06	0.000110056
c156853_g5	-2.54198	1.425979	4.40E-10	1.73E-08
c156856_g2	-2.18045	2.87583	8.25E-13	4.97E-11
c156860_g2	2.29952	7.514525	3.15E-28	1.15E-25
c156895_g1	6.458942	-1.32344	1.84E-05	0.000292482

c156899_g1	2.083841	2.42907	3.40E-11	1.57E-09
c156956_g4	-2.1125	0.27365	1.50E-05	0.000242424
c156976_g1	6.598503	-1.22186	7.85E-06	0.00013588
c156979_g4	-2.00307	3.024664	3.19E-05	0.000477349
c156986_g1	6.225963	-1.4893	6.83E-05	0.000939196
c156993_g1	8.810972	0.701386	5.83E-20	8.28E-18
c157013_g4	2.478419	7.124694	1.02E-22	2.05E-20
c157042_g1	6.782057	-1.08213	2.44E-06	4.73E-05
c157065_g1	6.309241	-1.43166	5.11E-05	0.000724427
c157091_g2	2.432872	1.70874	9.90E-11	4.27E-09
c157116_g1	3.094311	3.843083	2.99E-27	9.78E-25
c157116_g3	3.200005	5.598459	6.45E-45	1.19E-41
c157118_g1	4.243888	0.522594	3.06E-14	2.21E-12
c157128_g1	7.221083	-0.72869	4.18E-08	1.15E-06
c157257_g1	9.084365	0.960314	2.05E-20	3.13E-18
c157269_g2	2.279668	6.582845	6.75E-30	2.96E-27
c157274_g2	7.516697	-0.4751	2.58E-10	1.04E-08
c157307_g1	9.431953	1.301055	1.78E-28	6.66E-26
c157310_g1	6.594705	-1.22262	7.38E-06	0.000129271
c157328_g1	6.768021	-1.0859	1.15E-06	2.38E-05
c157328_g2	7.654768	-0.35936	2.57E-10	1.04E-08
c157335_g2	-2.47088	-0.06074	1.92E-05	0.000303737
c157336_g3	7.12701	-0.80237	2.85E-08	8.05E-07
c157364_g1	6.545551	-1.26947	6.21E-05	0.000863053
c157395_g1	6.375582	-1.37927	1.95E-05	0.000307722
c157397_g2	6.7108	-1.13054	1.75E-06	3.51E-05
c157411_g1	6.30252	-1.43322	2.91E-05	0.000441924
c157439_g1	6.8301	-1.04034	5.21E-07	1.16E-05
c157476_g1	2.173418	0.456199	1.87E-05	0.000297005
c157533_g1	7.168065	-0.76727	1.58E-08	4.67E-07
c157588_g1	7.922644	-0.11568	7.02E-13	4.28E-11
c157614_g1	2.513372	3.915344	7.52E-17	7.63E-15
c157621_g1	11.56858	3.415375	4.77E-34	3.01E-31
c157630_g2	6.786144	-1.08137	4.45E-06	8.17E-05
c157668_g1	4.644562	4.983214	8.77E-34	5.22E-31
c157698_g1	7.377374	-0.59604	3.01E-09	1.02E-07
c157743_g1	2.95273	0.566281	6.34E-07	1.39E-05
c157779_g1	6.305477	-1.43244	3.53E-05	0.000522258
c157801_g2	6.594703	-1.22262	7.37E-06	0.000129212
c157822_g1	6.878297	-0.99978	2.75E-07	6.43E-06
c157826_g1	6.936003	-0.95668	1.95E-07	4.73E-06
c157880_g1	9.406353	1.272126	2.70E-23	5.86E-21
c157896_g2	6.225958	-1.4893	6.82E-05	0.000938628
c157921_g2	6.594704	-1.22262	7.38E-06	0.000129214
c157933_g1	6.372313	-1.38005	2.65E-05	0.000406435
c157947_g2	2.086863	2.516196	4.16E-12	2.24E-10
c157964_g1	7.648895	-0.36142	6.16E-11	2.73E-09
c158000_g1	6.839641	-1.03808	1.97E-06	3.91E-05
c158110_g1	2.636492	-0.21652	6.25E-06	0.000111491
c158135_g1	7.564927	-0.44152	5.19E-09	1.70E-07
c158178_g1	-2.34324	1.420499	1.30E-07	3.26E-06
c158180_g1	7.42067	-0.56273	7.77E-09	2.45E-07
c158225_g1	8.967396	0.851522	4.08E-22	7.56E-20
c158227_g2	6.668313	-1.17278	1.23E-05	0.000204454

c158233_g1	7.106565	-0.83395	2.68E-06	5.16E-05
c158296_g1	7.004318	-0.91249	2.30E-06	4.50E-05
c158315_g2	4.653886	-0.95766	5.30E-06	9.58E-05
c158341_g1	6.651148	-1.17659	2.83E-06	5.43E-05
c158389_g1	6.899209	-0.99455	3.08E-06	5.86E-05
c158426_g1	7.790107	-0.23434	4.51E-12	2.41E-10
c158472_g1	3.485707	1.895024	2.92E-20	4.37E-18
c158509_g1	6.715766	-1.12902	1.34E-06	2.75E-05
c158510_g1	6.450211	-1.32576	1.08E-05	0.000180356
c158510_g2	7.028296	-0.87972	6.78E-08	1.79E-06
c158510_g4	6.987391	-0.91619	1.41E-07	3.50E-06
c158557_g1	6.452761	-1.32498	1.07E-05	0.000179552
c158566_g1	2.156881	2.162542	9.52E-11	4.11E-09
c158572_g3	6.296784	-1.43478	3.72E-05	0.000545913
c158572_g4	2.903924	3.191809	7.23E-08	1.90E-06
c158655_g1	6.648636	-1.17736	3.06E-06	5.83E-05
c158707_g1	6.936003	-0.95668	1.95E-07	4.73E-06
c158723_g1	7.787934	-0.25039	2.86E-09	9.77E-08
c158775_g1	6.713212	-1.12978	1.77E-06	3.54E-05
c158798_g3	2.18263	3.388049	1.29E-10	5.49E-09
c158832_g1	7.365854	-0.6003	2.27E-09	7.87E-08
c158848_g4	2.117866	0.810336	1.65E-05	0.000266016
c158947_g1	6.393133	-1.37539	6.65E-05	0.000919483
c158961_g4	6.299839	-1.434	2.97E-05	0.00044855
c158986_g2	-2.84975	-0.60805	4.72E-05	0.000677572
c158988_g1	7.270067	-0.6924	9.84E-08	2.53E-06
c158990_g1	-2.12378	5.065395	1.07E-20	1.68E-18
c159065_g1	2.942539	6.819811	2.93E-35	2.13E-32
c159065_g2	2.892727	4.484419	2.90E-25	7.82E-23
c159085_g1	7.094555	-0.83616	4.17E-07	9.48E-06
c159125_g1	6.775441	-1.08364	9.82E-07	2.07E-05
c159154_g1	7.578479	-0.41959	9.01E-11	3.91E-09
c159160_g1	9.700656	1.56243	3.40E-30	1.53E-27
c159185_g4	-2.52714	0.259391	4.16E-06	7.71E-05
c159205_g1	6.987389	-0.91619	1.41E-07	3.50E-06
c159299_g1	8.255353	0.181125	6.80E-15	5.35E-13
c159320_g1	3.110211	0.666872	1.27E-10	5.42E-09
c159340_g1	6.30252	-1.43322	2.91E-05	0.000441924
c159345_g1	6.450212	-1.32576	1.07E-05	0.000180255
c159364_g2	3.182647	0.84326	2.53E-10	1.03E-08
c159366_g1	6.292844	-1.43556	5.57E-05	0.000779172
c159388_g1	7.489395	-0.50231	1.73E-09	6.13E-08
c159390_g2	-3.08386	0.072347	1.04E-06	2.18E-05
c159433_g1	6.225957	-1.4893	6.81E-05	0.000938198
c159439_g1	6.891362	-0.99604	8.83E-07	1.88E-05
c159500_g1	6.518552	-1.27486	7.57E-06	0.000131529
c159509_g4	2.640924	-0.22288	1.28E-05	0.000212805
c159515_g1	6.99002	-0.91545	2.05E-07	4.92E-06
c159541_g1	7.078436	-0.84056	3.73E-08	1.04E-06
c159560_g1	2.471979	3.206696	1.85E-15	1.55E-13
c159571_g4	2.186989	5.182312	3.68E-18	4.36E-16
c159587_g5	2.797979	1.549023	2.29E-11	1.09E-09
c159600_g1	2.69953	2.874309	3.52E-19	4.57E-17
c159624_g1	10.53205	2.383547	5.77E-39	5.97E-36

c159635_g1	6.455543	-1.32421	1.29E-05	0.00021358
c159673_g1	6.83283	-1.03958	7.19E-07	1.56E-05
c159677_g1	6.663768	-1.17355	6.66E-06	0.000118292
c159683_g1	2.07808	0.746437	1.30E-06	2.67E-05
c159706_g1	2.816473	2.264246	3.59E-14	2.56E-12
c159751_g2	6.888112	-0.99679	5.36E-07	1.18E-05
c159805_g1	2.069458	1.530351	5.26E-05	0.000742961
c159870_g1	6.463167	-1.32266	2.99E-05	0.00045029
c159888_g5	6.468231	-1.32189	3.68E-05	0.000542132
c159948_g1	6.388166	-1.37617	3.87E-05	0.000565886
c159967_g2	10.60899	2.458477	5.40E-34	3.32E-31
c159975_g3	2.449276	1.919723	9.28E-12	4.70E-10
c159992_g1	2.173909	3.218828	1.01E-11	5.07E-10
c160005_g1	7.585184	-0.41683	1.44E-10	6.11E-09
c160033_g1	6.770399	-1.08515	1.08E-06	2.25E-05
c160057_g1	7.246512	-0.6996	1.02E-08	3.13E-07
c160075_g1	10.1411	1.996221	2.62E-35	1.94E-32
c160086_g1	6.219734	-1.49087	4.66E-05	0.000671041
c160139_g1	7.78534	-0.25107	1.29E-09	4.67E-08
c160139_g2	8.650736	0.548568	7.90E-18	9.06E-16
c160140_g1	3.874273	3.08161	1.05E-13	7.00E-12
c160140_g2	3.9755	3.786834	6.83E-31	3.24E-28
c160148_g3	2.492325	0.064056	1.88E-06	3.74E-05
c160214_g2	2.454232	0.430308	7.61E-07	1.63E-05
c160214_g4	3.035637	-0.73846	2.21E-05	0.000344928
c160290_g1	-3.32353	-0.2126	6.73E-05	0.00092877
c160313_g1	8.032979	-0.02132	2.81E-13	1.79E-11
c160327_g1	7.418046	-0.56344	4.19E-09	1.40E-07
c160333_g1	7.216017	-0.73014	1.67E-08	4.91E-07
c160391_g1	6.219733	-1.49087	4.67E-05	0.000671052
c160405_g1	2.533667	4.004099	1.05E-19	1.44E-17
c160449_g1	-2.20532	4.958118	1.25E-14	9.51E-13
c160449_g2	-2.55711	1.31428	8.54E-11	3.71E-09
c160508_g1	6.772798	-1.08439	7.60E-07	1.63E-05
c160520_g1	7.581739	-0.41821	9.06E-11	3.93E-09
c160553_g1	6.591545	-1.22339	5.27E-06	9.56E-05
c160557_g1	6.827676	-1.04109	4.37E-07	9.86E-06
c160631_g1	7.452926	-0.53291	1.93E-09	6.79E-08
c160723_g2	4.189801	6.684823	3.61E-16	3.32E-14
c160766_g1	6.219734	-1.49087	4.67E-05	0.000671041
c160773_g2	5.151783	-0.56435	1.91E-07	4.64E-06
c160861_g1	7.087943	-0.83762	1.22E-07	3.08E-06
c160894_g1	7.261109	-0.69457	2.10E-08	6.08E-07
c160895_g1	6.713211	-1.12978	1.76E-06	3.53E-05
c160941_g1	7.371351	-0.59817	1.85E-09	6.54E-08
c160950_g1	9.207391	1.082998	2.76E-25	7.47E-23
c160968_g1	6.948567	-0.9537	1.35E-06	2.77E-05
c160984_g3	6.219734	-1.49087	4.67E-05	0.000671041
c160995_g1	6.380978	-1.37772	1.92E-05	0.000303523
c161003_g1	6.713212	-1.12978	1.77E-06	3.54E-05
c161026_g1	8.280954	0.202019	5.47E-14	3.79E-12
c161034_g1	-6.45146	-1.172	5.64E-05	0.000787756
c161040_g1	6.515559	-1.27563	9.93E-06	0.000168199
c161058_g1	7.809708	-0.22698	2.49E-10	1.01E-08

c161070_g1	6.938556	-0.95593	2.68E-07	6.31E-06
c161075_g2	6.891367	-0.99604	8.86E-07	1.88E-05
c161112_g1	7.174517	-0.76509	3.17E-08	8.90E-07
c161119_g2	2.070218	4.400807	2.09E-16	1.97E-14
c161121_g1	7.338086	-0.62863	5.21E-09	1.70E-07
c161144_g1	7.514961	-0.4758	2.17E-10	8.89E-09
c161149_g1	8.797276	0.681158	1.94E-15	1.63E-13
c161166_g2	-2.29079	0.076577	1.78E-05	0.000284742
c161205_g1	7.039765	-0.87603	1.55E-07	3.83E-06
c161241_g1	6.673367	-1.17202	2.36E-05	0.000366239
c161247_g2	3.069461	7.628727	7.61E-19	9.55E-17
c161267_g1	8.122678	0.061481	2.23E-14	1.64E-12
c161274_g2	6.530367	-1.27178	1.48E-05	0.000240602
c161300_g1	6.993024	-0.91471	3.35E-07	7.73E-06
c161309_g1	7.639095	-0.36552	5.91E-11	2.63E-09
c161311_g2	6.296785	-1.43478	3.71E-05	0.000545913
c161317_g2	6.651148	-1.17659	2.83E-06	5.43E-05
c161327_g1	6.778494	-1.08288	1.46E-06	2.97E-05
c161334_g1	6.931532	-0.95817	1.60E-07	3.93E-06
c161372_g1	11.58008	5.668416	9.08E-97	1.56E-92
c161373_g1	7.647189	-0.3621	4.62E-11	2.10E-09
c161383_g1	6.521152	-1.27409	6.89E-06	0.000121585
c161384_g1	9.27576	1.142512	1.29E-19	1.75E-17
c161397_g2	6.586361	-1.22492	4.41E-06	8.16E-05
c161397_g3	6.882818	-0.99829	2.85E-07	6.64E-06
c161398_g1	6.88529	-0.99754	3.64E-07	8.35E-06
c161416_g2	6.523751	-1.27332	7.54E-06	0.000131459
c161422_g1	6.591545	-1.22339	5.28E-06	9.56E-05
c161436_g1	6.586361	-1.22492	4.42E-06	8.16E-05
c161437_g1	6.463172	-1.32266	2.99E-05	0.000450593
c161440_g2	6.920804	-0.96114	8.96E-07	1.90E-05
c161441_g1	6.882817	-0.99829	2.84E-07	6.64E-06
c161467_g1	2.413091	3.581551	7.95E-16	6.98E-14
c161480_g1	2.452817	1.870218	8.75E-13	5.25E-11
c161497_g1	7.293802	-0.66337	4.43E-09	1.47E-07
c161502_g1	6.710799	-1.13054	1.75E-06	3.52E-05
c161510_g1	7.901756	-0.10619	1.97E-06	3.91E-05
c161510_g2	5.967284	6.172553	3.44E-07	7.93E-06
c161530_g1	8.460563	0.373228	1.66E-17	1.83E-15
c161539_g1	7.030414	-0.87898	6.04E-08	1.62E-06
c161547_g1	7.963896	-0.08603	4.49E-12	2.40E-10
c161558_g1	6.715766	-1.12902	1.34E-06	2.75E-05
c161577_g1	6.653675	-1.17583	3.11E-06	5.91E-05
c161587_g1	8.337787	0.255929	5.35E-15	4.26E-13
c161616_g1	6.843767	-1.03733	3.71E-06	6.97E-05
c161617_g1	6.598501	-1.22186	7.85E-06	0.00013588
c161620_g1	10.08472	1.938523	6.16E-30	2.75E-27
c161622_g1	2.127101	1.061322	5.94E-06	0.00010641
c161686_g1	6.708278	-1.1313	2.03E-06	4.00E-05
c161704_g1	6.375582	-1.37927	1.95E-05	0.000307722
c161709_g1	6.832829	-1.03958	7.19E-07	1.56E-05
c161721_g1	6.931532	-0.95817	1.60E-07	3.93E-06
c161722_g1	-2.0654	0.988139	2.89E-06	5.52E-05
c161748_g1	2.66135	2.924723	4.03E-11	1.84E-09

c161750_g1	6.7054	-1.13206	2.75E-06	5.28E-05
c161751_g1	6.98505	-0.91693	1.11E-07	2.81E-06
c161767_g1	6.518552	-1.27486	7.57E-06	0.000131529
c161770_g1	7.51159	-0.47719	3.21E-10	1.28E-08
c161794_g1	7.481255	-0.50511	3.68E-10	1.46E-08
c161823_g3	2.413364	1.04778	6.93E-08	1.83E-06
c161824_g1	7.291934	-0.66409	4.23E-09	1.41E-07
c161832_g2	7.037059	-0.87677	1.01E-07	2.59E-06
c161844_g1	6.929296	-0.95891	1.81E-07	4.42E-06
c161863_g2	6.882817	-0.99829	2.85E-07	6.64E-06
c161868_g1	7.308366	-0.65907	4.26E-08	1.17E-06
c161870_g1	6.588864	-1.22415	4.43E-06	8.16E-05
c161881_g1	7.878831	-0.15843	1.44E-12	8.28E-11
c161897_g1	7.359167	-0.60242	8.13E-09	2.55E-07
c161902_g1	7.514961	-0.4758	2.17E-10	8.89E-09
c161917_g1	7.604605	-0.39402	2.40E-10	9.77E-09
c161932_g1	2.688107	2.273651	8.39E-13	5.05E-11
c161943_g1	6.880546	-0.99904	2.59E-07	6.11E-06
c161951_g1	6.526733	-1.27255	9.82E-06	0.000166584
c161965_g1	8.532011	0.439145	6.24E-18	7.26E-16
c161970_g1	8.869813	0.755652	2.72E-19	3.55E-17
c161994_g1	6.978481	-0.91915	1.27E-07	3.18E-06
c161999_g1	3.053522	7.869058	7.09E-40	8.33E-37
c162000_g2	2.393656	4.850985	1.09E-19	1.50E-17
c162050_g2	7.076405	-0.84129	3.88E-08	1.08E-06
c162068_g1	6.51556	-1.27563	9.92E-06	0.000168117
c162075_g1	6.526732	-1.27255	9.82E-06	0.000166584
c162086_g1	6.30924	-1.43166	5.11E-05	0.000724427
c162126_g1	6.21687	-1.49166	5.34E-05	0.000751038
c162137_g1	8.331222	0.252897	3.12E-16	2.89E-14
c162155_g1	6.778493	-1.08288	1.46E-06	2.96E-05
c162190_g1	2.417715	1.349978	1.60E-07	3.93E-06
c162191_g1	4.240308	0.507074	3.78E-14	2.69E-12
c162191_g2	3.026726	0.085521	3.25E-07	7.54E-06
c162195_g1	6.586361	-1.22492	4.42E-06	8.16E-05
c162255_g1	7.198425	-0.76	2.22E-06	4.36E-05
c162302_g1	2.630597	2.399671	2.50E-15	2.08E-13
c162381_g1	6.468237	-1.32189	3.69E-05	0.000542759
c162387_g1	6.305477	-1.43244	3.53E-05	0.000522258
c162391_g2	6.225951	-1.4893	6.79E-05	0.000936578
c162435_g1	6.526729	-1.27255	9.79E-06	0.000166512
c162463_g1	6.990021	-0.91545	2.05E-07	4.92E-06
c162485_g2	6.219734	-1.49087	4.66E-05	0.000671041
c162535_g1	6.29984	-1.434	2.97E-05	0.000448265
c162541_g1	6.613695	-1.21956	5.30E-05	0.000749005
c162558_g1	8.01438	-0.05611	1.68E-08	4.93E-07
c162568_g1	6.588865	-1.22415	4.44E-06	8.17E-05
c162587_g1	6.602995	-1.22109	1.42E-05	0.000232046
c162590_g2	6.715766	-1.12902	1.34E-06	2.75E-05
c162599_g1	6.822999	-1.04259	7.61E-07	1.63E-05
c162608_g1	8.777313	0.665146	6.41E-17	6.58E-15
c162616_g1	7.452925	-0.53291	1.93E-09	6.79E-08
c162656_g1	6.708276	-1.1313	2.03E-06	4.00E-05
c162660_g1	10.69601	2.546043	4.75E-38	4.31E-35

c162665_g1	8.63105	0.531791	1.18E-18	1.46E-16
c162691_g1	3.397554	0.932485	1.59E-13	1.04E-11
c162709_g1	6.526732	-1.27255	9.82E-06	0.000166584
c162721_g2	6.393135	-1.37539	6.65E-05	0.000919483
c162733_g1	2.694225	8.811188	1.20E-38	1.17E-35
c162736_g1	2.146188	6.082102	1.23E-14	9.37E-13
c162739_g3	5.814807	1.985195	4.25E-27	1.37E-24
c162742_g4	2.73074	4.491389	1.57E-21	2.73E-19
c162758_g1	7.277157	-0.69096	4.26E-07	9.66E-06
c162775_g1	6.474002	-1.32111	7.11E-05	0.000974213
c162787_g1	7.905921	-0.13458	1.13E-12	6.68E-11
c162806_g1	7.180043	-0.76364	8.89E-08	2.30E-06
c162838_g1	6.296785	-1.43478	3.71E-05	0.000545913
c162950_g2	6.790694	-1.08061	8.54E-06	0.000146818
c162951_g3	2.109859	1.947456	1.19E-08	3.60E-07
c162954_g2	-2.26176	0.171783	1.29E-05	0.00021358
c162980_g1	6.530367	-1.27178	1.48E-05	0.000240602
c162988_g1	6.30252	-1.43322	2.91E-05	0.000441924
c163008_g1	-2.86724	0.869672	8.00E-08	2.09E-06
c163018_g2	3.346673	0.669717	5.36E-05	0.000754259
c163074_g1	7.555201	-0.4443	4.19E-10	1.65E-08
c163088_g2	3.334705	-0.50872	5.29E-06	9.57E-05
c163088_g3	2.320487	-0.17166	3.53E-05	0.000522258
c163115_g2	7.285942	-0.66624	7.93E-09	2.49E-07
c163120_g1	7.549585	-0.44638	1.54E-10	6.47E-09
c163126_g1	7.520527	-0.47371	5.32E-10	2.06E-08
c163131_g1	8.783901	0.675157	2.40E-19	3.16E-17
c163175_g5	6.388172	-1.37617	3.88E-05	0.000566211
c163207_g1	6.668314	-1.17278	1.23E-05	0.000204454
c163223_g1	6.651148	-1.17659	2.83E-06	5.43E-05
c163226_g1	7.591314	-0.41476	6.06E-10	2.32E-08
c163227_g1	7.050089	-0.87382	9.79E-07	2.07E-05
c163241_g1	2.15631	0.572017	1.67E-05	0.000268939
c163263_g1	6.762453	-1.08741	2.05E-06	4.04E-05
c163271_g6	2.039382	5.376263	1.29E-12	7.50E-11
c163276_g1	7.080494	-0.83982	4.11E-08	1.14E-06
c163305_g1	7.23077	-0.72652	2.23E-07	5.31E-06
c163311_g1	8.993539	0.863954	6.21E-14	4.28E-12
c163328_g7	2.838674	4.544202	3.17E-23	6.83E-21
c163328_g8	2.458592	4.512801	8.17E-23	1.68E-20
c163330_g1	8.322199	0.2394	4.21E-14	2.97E-12
c163339_g3	2.71983	2.130568	1.98E-07	4.79E-06
c163346_g1	6.393135	-1.37539	6.65E-05	0.000919483
c163362_g1	-2.03204	0.793347	1.84E-05	0.000292162
c163371_g4	2.958088	2.558641	1.21E-16	1.18E-14
c163372_g1	2.005891	6.441592	7.68E-22	1.38E-19
c163384_g1	7.705151	-0.31003	1.54E-11	7.58E-10
c163393_g1	6.518552	-1.27486	7.57E-06	0.000131529
c163412_g1	6.825377	-1.04184	4.29E-07	9.70E-06
c163431_g1	2.11408	5.492579	1.76E-17	1.93E-15
c163442_g1	7.426536	-0.56131	3.18E-08	8.93E-07
c163448_g5	5.285579	-0.44657	7.19E-09	2.28E-07
c163448_g6	3.488135	0.454238	2.62E-11	1.24E-09
c163494_g1	6.30252	-1.43322	2.91E-05	0.000441924

c163550_g1	6.521152	-1.27409	6.90E-06	0.000121585
c163551_g1	2.570099	4.570242	6.61E-21	1.07E-18
c163615_g1	7.076406	-0.84129	3.87E-08	1.08E-06
c163617_g1	6.786143	-1.08137	4.45E-06	8.17E-05
c163617_g2	6.648636	-1.17736	3.06E-06	5.83E-05
c163624_g1	7.034665	-0.87751	7.42E-08	1.95E-06
c163625_g1	7.039766	-0.87603	1.55E-07	3.83E-06
c163636_g1	6.222518	-1.49009	5.10E-05	0.000724089
c163650_g4	-2.00817	2.358219	3.25E-09	1.10E-07
c163655_g1	8.584635	0.487335	9.04E-18	1.02E-15
c163667_g1	8.754152	0.647342	2.34E-19	3.11E-17
c163669_g1	9.235546	1.107621	4.58E-23	9.79E-21
c163678_g1	6.993025	-0.91471	3.35E-07	7.73E-06
c163683_g3	2.688281	0.613949	1.28E-05	0.000212044
c163715_g1	7.553194	-0.44499	2.70E-10	1.09E-08
c163717_g1	6.97848	-0.91915	1.27E-07	3.18E-06
c163755_g3	6.656495	-1.17507	3.98E-06	7.41E-05
c163755_g7	6.526725	-1.27255	9.77E-06	0.000166512
c163757_g3	7.034665	-0.87751	7.41E-08	1.95E-06
c163759_g1	6.598504	-1.22186	7.86E-06	0.00013588
c163778_g1	6.715765	-1.12902	1.34E-06	2.75E-05
c163778_g4	6.895075	-0.9953	1.60E-06	3.24E-05
c163800_g3	6.216871	-1.49166	5.33E-05	0.000750888
c163815_g1	7.338085	-0.62863	5.20E-09	1.70E-07
c163818_g1	6.933713	-0.95742	1.64E-07	4.02E-06
c163851_g1	8.463966	0.374996	4.87E-17	5.06E-15
c163860_g1	7.788597	-0.23501	5.52E-12	2.91E-10
c163873_g1	6.642543	-1.17888	5.76E-06	0.000103407
c163875_g1	7.489395	-0.50231	1.73E-09	6.13E-08
c163931_g1	4.928856	-0.73083	2.71E-07	6.37E-06
c163970_g3	7.174517	-0.76509	3.17E-08	8.90E-07
c163973_g1	6.534755	-1.27101	1.70E-05	0.000273657
c163979_g1	6.378291	-1.3785	1.76E-05	0.000281493
c163990_g1	7.787017	-0.23567	7.53E-12	3.86E-10
c163993_g1	8.096844	0.039317	1.88E-14	1.39E-12
c163994_g4	4.489954	2.52914	1.22E-20	1.90E-18
c163996_g1	6.434374	-1.32884	5.87E-05	0.000817232
c163997_g1	6.598504	-1.22186	7.86E-06	0.00013588
c164019_g1	6.659824	-1.17431	3.91E-06	7.31E-05
c164023_g1	2.692267	5.447451	7.53E-15	5.89E-13
c164024_g1	7.647189	-0.3621	4.62E-11	2.10E-09
c164104_g2	6.29984	-1.434	2.96E-05	0.000448141
c164104_g3	6.530367	-1.27178	1.48E-05	0.000240602
c164111_g1	6.302519	-1.43322	2.91E-05	0.000441924
c164119_g1	8.720472	0.610194	6.08E-16	5.44E-14
c164169_g1	7.288081	-0.66552	5.68E-09	1.85E-07
c164171_g1	7.738648	-0.28234	1.45E-11	7.13E-10
c164173_g1	6.880545	-0.99904	2.59E-07	6.11E-06
c164177_g1	6.878297	-0.99978	2.74E-07	6.43E-06
c164205_g1	7.624527	-0.38646	5.39E-10	2.08E-08
c164205_g2	8.227715	0.158736	1.73E-15	1.46E-13
c164205_g3	6.521152	-1.27409	6.90E-06	0.000121585
c164214_g1	7.120941	-0.80455	2.57E-08	7.33E-07
c164227_g1	6.530366	-1.27178	1.48E-05	0.000240602

c164230_g3	6.216872	-1.49166	5.33E-05	0.000750756
c164233_g1	6.827676	-1.04109	4.38E-07	9.86E-06
c164233_g2	8.006615	-0.04407	2.07E-13	1.35E-11
c164245_g1	7.06293	-0.8716	6.43E-06	0.000114536
c164258_g2	6.219734	-1.49087	4.66E-05	0.000671041
c164262_g2	6.518552	-1.27486	7.56E-06	0.000131529
c164270_g1	7.989665	-0.06326	4.21E-12	2.26E-10
c164288_g2	7.03249	-0.87824	6.21E-08	1.66E-06
c164292_g1	6.800869	-1.0791	3.35E-05	0.000499739
c164297_g1	9.015644	0.892912	9.87E-19	1.23E-16
c164303_g1	7.172181	-0.76582	2.21E-08	6.39E-07
c164306_g1	6.668312	-1.17278	1.23E-05	0.000204454
c164327_g2	6.713213	-1.12978	1.77E-06	3.54E-05
c164346_g3	-2.47946	4.295264	1.58E-23	3.49E-21
c164346_g4	-2.08208	1.365772	1.98E-07	4.80E-06
c164349_g5	5.696044	1.889382	4.26E-31	2.04E-28
c164368_g1	6.225953	-1.4893	6.80E-05	0.000937178
c164369_g1	6.384156	-1.37694	2.53E-05	0.000389761
c164381_g1	9.729853	1.591283	8.55E-31	4.02E-28
c164390_g2	7.796039	-0.23166	6.05E-12	3.16E-10
c164407_g2	6.591544	-1.22339	5.27E-06	9.56E-05
c164430_g1	6.772799	-1.08439	7.60E-07	1.63E-05
c164435_g1	8.314815	0.236354	1.52E-15	1.29E-13
c164437_g1	7.258652	-0.69529	1.34E-08	4.01E-07
c164454_g2	6.598508	-1.22186	7.87E-06	0.000135998
c164460_g2	6.832826	-1.03958	7.16E-07	1.55E-05
c164484_g2	6.468229	-1.32189	3.68E-05	0.000541871
c164509_g1	9.238427	1.113138	8.79E-26	2.48E-23
c164516_g1	6.305478	-1.43244	3.53E-05	0.000522258
c164521_g1	6.663769	-1.17355	6.67E-06	0.000118305
c164524_g2	6.518552	-1.27486	7.56E-06	0.000131529
c164525_g1	8.27937	0.201405	2.56E-14	1.87E-12
c164527_g1	8.227715	0.158736	1.73E-15	1.46E-13
c164530_g1	6.521152	-1.27409	6.90E-06	0.000121585
c164532_g2	6.832829	-1.03958	7.19E-07	1.56E-05
c164534_g1	6.219734	-1.49087	4.67E-05	0.000671041
c164546_g1	7.085162	-0.83836	7.54E-08	1.98E-06
c164561_g1	7.485012	-0.50371	6.55E-10	2.49E-08
c164565_g1	2.023426	4.54722	6.62E-15	5.22E-13
c164567_g2	6.8301	-1.04034	5.21E-07	1.16E-05
c164578_g1	7.183258	-0.76291	1.68E-07	4.10E-06
c164592_g1	6.990021	-0.91545	2.05E-07	4.92E-06
c164594_g1	6.384158	-1.37694	2.53E-05	0.000389906
c164603_g1	6.98739	-0.91619	1.41E-07	3.50E-06
c164619_g1	7.091076	-0.83689	2.18E-07	5.21E-06
c164623_g1	6.820349	-1.04334	1.01E-06	2.12E-05
c164627_g1	7.826315	-0.20596	6.84E-12	3.53E-10
c164630_g3	3.375092	5.139804	1.51E-38	1.42E-35
c164630_g8	4.471302	-1.08916	2.56E-05	0.000393036
c164637_g1	7.96785	-0.08472	1.93E-11	9.34E-10
c164642_g7	6.225964	-1.4893	6.83E-05	0.000939196
c164669_g3	2.601175	5.273144	1.49E-12	8.56E-11
c164682_g1	7.734039	-0.28436	9.85E-12	4.96E-10
c164684_g1	8.156777	0.086501	1.25E-12	7.31E-11

c164761_g1	7.986063	-0.06456	1.12E-12	6.61E-11
c164767_g1	6.638603	-1.17964	9.62E-06	0.000164474
c164816_g1	7.023419	-0.88119	1.31E-07	3.27E-06
c164833_g1	2.078913	1.050627	1.02E-07	2.62E-06
c164851_g1	7.209913	-0.73231	1.00E-08	3.09E-07
c164853_g1	10.25544	2.108976	1.67E-35	1.26E-32
c164872_g1	7.75813	1.853997	9.01E-30	3.85E-27
c164874_g1	6.521152	-1.27409	6.89E-06	0.000121585
c164901_g3	6.450213	-1.32576	1.07E-05	0.000180189
c164903_g1	7.516697	-0.4751	2.58E-10	1.04E-08
c164911_g5	6.594702	-1.22262	7.37E-06	0.000129212
c164931_g1	6.225955	-1.4893	6.81E-05	0.000937669
c164938_g2	7.706685	-0.30936	1.65E-11	8.07E-10
c164952_g1	6.770399	-1.08515	1.08E-06	2.25E-05
c164953_g1	7.321918	-0.63433	9.64E-09	2.98E-07
c164954_g1	7.352033	-0.62506	7.90E-08	2.06E-06
c164984_g1	6.222518	-1.49009	5.10E-05	0.000724089
c165004_g1	7.31161	-0.65835	8.74E-08	2.27E-06
c165024_g1	6.642542	-1.17888	5.76E-06	0.000103407
c165036_g2	6.534437	1.490837	4.09E-29	1.63E-26
c165044_g1	6.98505	-0.91693	1.11E-07	2.81E-06
c165084_g1	2.617082	1.273181	1.42E-06	2.90E-05
c165085_g2	6.22596	-1.4893	6.82E-05	0.000938885
c165089_g1	2.343896	4.625767	3.32E-21	5.54E-19
c165091_g2	6.656496	-1.17507	3.98E-06	7.41E-05
c165103_g2	7.316339	-0.63575	3.39E-08	9.50E-07
c165109_g1	8.328934	0.251684	1.81E-16	1.73E-14
c165148_g1	6.88529	-0.99754	3.64E-07	8.35E-06
c165154_g1	6.938551	-0.95593	2.67E-07	6.28E-06
c165182_g2	6.309243	-1.43166	5.12E-05	0.00072452
c165185_g3	6.450212	-1.32576	1.07E-05	0.000180255
c165192_g1	9.463904	1.330595	8.12E-27	2.56E-24
c165193_g1	9.165241	1.037539	2.13E-20	3.24E-18
c165198_g2	6.778492	-1.08288	1.46E-06	2.96E-05
c165207_g1	7.516697	-0.4751	2.58E-10	1.04E-08
c165214_g2	6.715767	-1.12902	1.34E-06	2.75E-05
c165215_g1	6.378291	-1.3785	1.75E-05	0.000280849
c165222_g1	7.124938	-0.80309	2.39E-08	6.85E-07
c165222_g2	6.468236	-1.32189	3.69E-05	0.00054269
c165226_g2	6.309241	-1.43166	5.11E-05	0.000724427
c165227_g2	6.511891	-1.2764	1.52E-05	0.000245739
c165236_g2	6.7054	-1.13206	2.75E-06	5.28E-05
c165238_g1	10.60439	2.456067	3.23E-42	4.64E-39
c165246_g1	7.982838	-0.06586	3.84E-13	2.41E-11
c165248_g1	8.957967	0.835197	9.00E-17	9.02E-15
c165254_g1	6.216873	-1.49166	5.32E-05	0.000750756
c165257_g1	8.0479	-0.00401	4.33E-14	3.04E-12
c165264_g1	7.224045	-0.72797	7.60E-08	1.99E-06
c165268_g1	6.775442	-1.08364	9.84E-07	2.07E-05
c165272_g1	7.428119	-0.54134	1.09E-08	3.33E-07
c165273_g1	8.698486	0.592916	9.55E-18	1.08E-15
c165285_g1	7.094554	-0.83616	4.16E-07	9.47E-06
c165294_g1	6.936003	-0.95668	1.95E-07	4.73E-06
c165298_g1	8.160585	0.087762	6.48E-12	3.36E-10

c165312_g1	8.277846	0.200791	1.24E-14	9.43E-13
c165322_g1	6.302519	-1.43322	2.91E-05	0.000441924
c165334_g1	6.530365	-1.27178	1.48E-05	0.000240602
c165350_g1	7.5004	-0.4995	2.74E-08	7.78E-07
c165355_g1	6.835987	-1.03883	1.13E-06	2.34E-05
c165358_g1	6.384155	-1.37694	2.53E-05	0.000389734
c165361_g1	8.762319	0.657483	1.01E-20	1.60E-18
c165365_g1	7.429725	-0.56061	6.71E-08	1.77E-06
c165375_g2	7.16406	-0.76872	1.92E-08	5.60E-07
c165389_g1	7.513276	-0.4765	2.05E-10	8.48E-09
c165389_g2	7.375207	-0.59675	2.91E-09	9.94E-08
c165391_g1	6.645848	-1.17812	3.89E-06	7.28E-05
c165395_g1	6.588864	-1.22415	4.43E-06	8.16E-05
c165398_g1	7.373216	-0.59746	2.18E-09	7.61E-08
c165400_g1	6.521152	-1.27409	6.90E-06	0.000121585
c165424_g1	7.862162	-0.17868	4.97E-11	2.24E-09
c165425_g1	6.380977	-1.37772	1.92E-05	0.000303523
c165430_g2	7.388192	-0.5932	3.51E-08	9.83E-07
c165434_g2	2.181292	4.558676	1.12E-21	1.98E-19
c165435_g1	6.72617	-1.12675	5.30E-06	9.58E-05
c165446_g1	6.713213	-1.12978	1.77E-06	3.54E-05
c165451_g2	7.131833	-0.8009	6.08E-08	1.63E-06
c165474_g1	8.146983	0.082721	2.85E-14	2.07E-12
c165475_g1	7.653289	1.751825	7.75E-30	3.34E-27
c165488_g1	9.634963	1.496677	3.49E-27	1.13E-24
c165491_g1	8.121376	0.060849	1.64E-14	1.23E-12
c165492_g1	6.888113	-0.99679	5.36E-07	1.18E-05
c165505_g1	9.208137	1.083479	4.48E-25	1.16E-22
c165515_g1	9.226448	1.09823	2.30E-22	4.43E-20
c165527_g1	6.37558	-1.37927	1.96E-05	0.000308214
c165538_g1	6.782055	-1.08213	2.44E-06	4.73E-05
c165540_g1	7.297808	-0.66194	6.94E-09	2.21E-07
c165544_g1	7.87883	-0.15843	1.44E-12	8.28E-11
c165547_g1	7.405956	-0.56767	1.17E-09	4.27E-08
c165556_g1	7.379741	-0.59533	4.95E-09	1.63E-07
c165559_g1	6.843767	-1.03733	3.71E-06	6.97E-05
c165562_g2	6.305478	-1.43244	3.53E-05	0.000522258
c165565_g1	4.051215	-0.7325	2.74E-06	5.26E-05
c165577_g1	10.64477	2.494198	1.22E-34	8.43E-32
c165585_g1	7.799363	-0.23032	1.32E-11	6.56E-10
c165601_g1	7.36161	-0.60171	4.79E-09	1.58E-07
c165617_g1	7.684432	-0.33276	1.26E-10	5.37E-09
c165633_g1	7.652668	-0.36005	1.47E-10	6.22E-09
c165639_g1	9.258339	1.124094	3.16E-18	3.78E-16
c165640_g1	6.895075	-0.9953	1.60E-06	3.24E-05
c165647_g1	7.332172	-0.63077	2.63E-09	9.03E-08
c165649_g2	6.830099	-1.04034	5.20E-07	1.16E-05
c165659_g1	9.410513	1.274386	1.96E-21	3.38E-19
c165660_g1	6.982881	-0.91767	1.00E-07	2.57E-06
c165660_g4	7.194378	-0.76073	1.07E-06	2.24E-05
c165672_g1	14.37989	6.231625	1.03E-88	1.06E-84
c165678_g1	6.378291	-1.3785	1.75E-05	0.000280849
c165679_g3	6.380977	-1.37772	1.92E-05	0.000303523
c165687_g1	7.074274	-0.84202	4.67E-08	1.28E-06

c165693_g1	6.594702	-1.22262	7.37E-06	0.000129212
c165705_g2	7.797651	-0.23099	8.54E-12	4.36E-10
c165719_g2	6.216872	-1.49166	5.33E-05	0.000750756
c165726_g1	8.471526	0.385872	1.01E-17	1.14E-15
c165727_g1	11.30262	3.152939	3.59E-57	1.32E-53
c165742_g2	2.109335	3.604872	2.10E-15	1.76E-13
c165742_g3	2.348528	5.692525	1.90E-22	3.70E-20
c165743_g1	8.071947	0.017486	2.79E-14	2.03E-12
c165747_g1	8.393951	0.308499	1.15E-15	9.92E-14
c165748_g1	7.382336	-0.59462	8.92E-09	2.77E-07
c165764_g1	8.29572	0.218379	3.06E-15	2.52E-13
c165768_g1	6.980742	-0.91841	1.04E-07	2.67E-06
c165777_g1	6.518552	-1.27486	7.56E-06	0.000131529
c165785_g1	6.778496	-1.08288	1.46E-06	2.97E-05
c165791_g1	6.588865	-1.22415	4.43E-06	8.16E-05
c165792_g1	7.902965	-0.1359	8.92E-13	5.33E-11
c165795_g2	7.444934	-0.53572	7.31E-10	2.76E-08
c165796_g1	6.586361	-1.22492	4.41E-06	8.16E-05
c165801_g9	8.15694	0.096785	1.47E-14	1.10E-12
c165816_g1	6.393136	-1.37539	6.66E-05	0.000919483
c165853_g2	6.225964	-1.4893	6.83E-05	0.000939196
c165856_g1	7.270065	-0.6924	9.83E-08	2.53E-06
c165864_g1	8.491788	0.395851	1.03E-14	7.90E-13
c165879_g5	6.463169	-1.32266	2.99E-05	0.00045029
c165886_g1	8.15694	0.096785	1.47E-14	1.10E-12
c165890_g1	9.606209	1.470148	2.12E-29	8.77E-27
c165911_g1	7.65702	-0.35868	4.82E-10	1.87E-08
c165913_g1	7.404152	-0.56838	1.31E-09	4.74E-08
c165915_g1	-6.50699	-1.12884	4.37E-05	0.000633255
c165916_g1	7.248608	-0.69888	7.68E-09	2.42E-07
c165922_g1	10.00974	1.867244	1.69E-35	1.26E-32
c165937_g1	6.839643	-1.03808	1.98E-06	3.91E-05
c165941_g1	6.30924	-1.43166	5.11E-05	0.000724427
c165942_g1	6.770399	-1.08515	1.08E-06	2.25E-05
c165942_g2	7.562239	-0.44222	2.53E-09	8.71E-08
c165946_g1	7.766845	-0.25712	8.30E-12	4.25E-10
c165948_g1	8.158264	0.097411	9.76E-15	7.55E-13
c165952_g1	7.711664	-0.30732	3.90E-11	1.79E-09
c165963_g3	7.413434	-0.56485	1.56E-09	5.58E-08
c165983_g1	7.622297	-0.38715	2.96E-10	1.19E-08
c165985_g1	6.936003	-0.95668	1.95E-07	4.73E-06
c165992_g1	7.67093	-0.33821	3.27E-11	1.52E-09
c165999_g1	7.51159	-0.47719	3.21E-10	1.28E-08
c166006_g1	8.027323	-0.02389	6.83E-14	4.66E-12
c166011_g2	6.299841	-1.434	2.96E-05	0.00044803
c166013_g1	6.987391	-0.91619	1.41E-07	3.50E-06
c166019_g1	7.848318	-0.18399	1.79E-12	1.01E-10
c166022_g1	7.085164	-0.83836	7.56E-08	1.98E-06
c166034_g2	6.710799	-1.13054	1.75E-06	3.52E-05
c166038_g1	6.378291	-1.3785	1.75E-05	0.000280861
c166053_g1	6.372313	-1.38005	2.65E-05	0.000406435
c166057_g3	6.530366	-1.27178	1.48E-05	0.000240602
c166058_g1	6.770399	-1.08515	1.08E-06	2.25E-05
c166083_g1	6.309242	-1.43166	5.11E-05	0.000724427

c166086_g1	4.692269	-0.92161	2.56E-06	4.96E-05
c166100_g1	7.624529	-0.38646	5.40E-10	2.08E-08
c166104_g1	6.926843	-0.95965	2.39E-07	5.67E-06
c166109_g1	7.497399	-0.5002	1.30E-08	3.90E-07
c166128_g1	7.213849	-0.73086	1.24E-08	3.75E-07
c166135_g1	8.386653	0.304905	6.15E-17	6.36E-15
c166140_g2	6.521152	-1.27409	6.90E-06	0.000121585
c166143_g1	6.839641	-1.03808	1.97E-06	3.91E-05
c166157_g1	6.730726	-1.12599	1.00E-05	0.000169212
c166171_g1	7.087945	-0.83762	1.22E-07	3.08E-06
c166181_g1	6.668314	-1.17278	1.23E-05	0.000204454
c166195_g1	-2.08212	0.527814	3.37E-05	0.000501947
c166206_g1	7.026003	-0.88045	8.79E-08	2.28E-06
c166208_g3	-2.81984	-0.14411	7.31E-06	0.000128456
c166224_g1	8.510315	0.420604	3.17E-18	3.78E-16
c166232_g1	7.85124	-0.18267	2.60E-12	1.45E-10
c166248_g2	7.046296	-0.87456	4.97E-07	1.11E-05
c166254_g1	8.024743	-0.02518	6.27E-14	4.31E-12
c166272_g1	6.987388	-0.91619	1.41E-07	3.50E-06
c166274_g1	6.526725	-1.27255	9.77E-06	0.000166512
c166275_g1	9.091566	0.969166	4.06E-22	7.55E-20
c166280_g1	6.586361	-1.22492	4.42E-06	8.16E-05
c166291_g1	6.588864	-1.22415	4.43E-06	8.16E-05
c166302_g1	6.608098	-1.22032	2.71E-05	0.00041405
c166304_g5	3.346543	0.353683	3.37E-09	1.14E-07
c166305_g1	6.598509	-1.22186	7.88E-06	0.000136087
c166318_g3	4.032811	3.483978	1.89E-42	2.79E-39
c166329_g1	7.129295	-0.80164	3.98E-08	1.10E-06
c166341_g2	6.463169	-1.32266	2.99E-05	0.00045029
c166343_g1	2.313002	5.499406	5.97E-21	9.71E-19
c166345_g1	6.225949	-1.4893	6.79E-05	0.000936055
c166351_g1	2.455883	5.418188	1.29E-21	2.27E-19
c166362_g1	8.146983	0.082721	2.85E-14	2.07E-12
c166380_g1	8.060767	0.001753	8.66E-13	5.20E-11
c166400_g1	6.455543	-1.32421	1.29E-05	0.000213581
c166404_g1	8.340816	0.257143	1.91E-14	1.41E-12
c166405_g1	7.815812	-0.21062	4.04E-12	2.18E-10
c166420_g1	7.437736	-0.53853	1.21E-09	4.40E-08
c166422_g2	3.036607	3.39278	6.17E-26	1.76E-23
c166423_g1	8.668675	0.55591	4.28E-13	2.68E-11
c166427_g1	6.659824	-1.17431	3.91E-06	7.31E-05
c166430_g1	7.409524	-0.56626	1.29E-09	4.68E-08
c166431_g2	7.32848	-0.63219	3.09E-09	1.05E-07
c166446_g1	2.450378	-0.105	2.60E-05	0.000399621
c166456_g1	6.588866	-1.22415	4.45E-06	8.17E-05
c166457_g1	9.248387	1.12289	5.17E-26	1.50E-23
c166468_g1	6.765436	-1.08665	1.43E-06	2.91E-05
c166473_g1	8.008115	-0.04342	3.25E-13	2.05E-11
c166483_g1	6.931532	-0.95817	1.60E-07	3.93E-06
c166486_g1	8.183236	0.108071	6.68E-12	3.45E-10
c166488_g1	6.825377	-1.04184	4.28E-07	9.70E-06
c166501_g1	8.483408	0.392327	8.70E-17	8.75E-15
c166506_g2	7.444935	-0.53572	7.32E-10	2.76E-08
c166510_g1	6.368152	-1.38083	4.20E-05	0.000609266

c166512_g1	8.330062	0.25229	2.26E-16	2.12E-14
c166514_g1	6.642544	-1.17888	5.76E-06	0.000103407
c166520_g1	6.837054	0.958744	2.09E-20	3.19E-18
c166522_g1	7.818785	-0.20929	2.72E-12	1.51E-10
c166523_g1	6.393136	-1.37539	6.66E-05	0.000919483
c166524_g3	6.830099	-1.04034	5.20E-07	1.16E-05
c166524_g4	6.219733	-1.49087	4.67E-05	0.000671061
c166524_g5	6.375582	-1.37927	1.95E-05	0.000307722
c166525_g1	7.791574	-0.23367	4.11E-12	2.22E-10
c166529_g3	7.342962	-0.6272	9.95E-09	3.06E-07
c166530_g2	7.074273	-0.84202	4.69E-08	1.28E-06
c166530_g3	7.420673	-0.56273	7.79E-09	2.45E-07
c166539_g1	7.280924	-0.69024	8.86E-07	1.88E-05
c166540_g1	7.573149	-0.42166	3.04E-10	1.22E-08
c166541_g2	6.302519	-1.43322	2.91E-05	0.000441924
c166543_g1	8.136882	0.077686	1.25E-14	9.50E-13
c166544_g1	8.112286	0.045669	2.80E-12	1.55E-10
c166554_g1	6.980742	-0.91841	1.04E-07	2.67E-06
c166568_g1	7.365853	-0.6003	2.27E-09	7.87E-08
c166569_g1	8.023444	-0.02582	7.05E-14	4.81E-12
c166579_g1	6.653674	-1.17583	3.11E-06	5.91E-05
c166586_g1	6.299841	-1.434	2.96E-05	0.00044803
c166594_g1	9.891415	1.749964	1.24E-32	6.60E-30
c166599_g1	7.382332	-0.59462	8.88E-09	2.76E-07
c166600_g1	10.09072	1.944404	7.12E-30	3.10E-27
c166610_g1	10.55075	2.401696	1.81E-37	1.54E-34
c166624_g2	6.817269	-1.04409	1.53E-06	3.11E-05
c166625_g1	8.382026	0.293792	4.58E-14	3.21E-12
c166627_g1	6.713212	-1.12978	1.77E-06	3.54E-05
c166636_g1	8.894282	0.779968	4.06E-20	5.98E-18
c166647_g1	8.146983	0.082721	2.84E-14	2.07E-12
c166652_g1	8.978551	0.862676	1.81E-22	3.56E-20
c166658_g1	6.594708	-1.22262	7.40E-06	0.000129311
c166679_g1	8.428098	0.341536	1.42E-16	1.39E-14
c166681_g1	7.709911	-0.308	2.64E-11	1.25E-09
c166688_g1	10.14734	2.002208	7.18E-35	5.16E-32
c166689_g18	5.50785	0.517394	3.21E-05	0.00048011
c166692_g1	7.375208	-0.59675	2.92E-09	9.95E-08
c166693_g1	10.76809	2.619171	4.18E-45	8.00E-42
c166697_g1	7.614821	-0.3899	6.34E-11	2.81E-09
c166702_g1	6.588864	-1.22415	4.43E-06	8.16E-05
c166703_g1	6.292845	-1.43556	5.56E-05	0.000779172
c166716_g3	6.931532	-0.95817	1.60E-07	3.93E-06
c166717_g1	8.935768	0.81855	1.17E-19	1.60E-17
c166724_g1	7.026002	-0.88045	8.81E-08	2.28E-06
c166725_g1	8.600266	0.502307	4.58E-18	5.37E-16
c166728_g1	7.216019	-0.73014	1.67E-08	4.91E-07
c166730_g1	6.372312	-1.38005	2.65E-05	0.000406435
c166730_g2	6.895077	-0.9953	1.60E-06	3.24E-05
c166736_g1	4.036995	-0.31057	1.09E-07	2.78E-06
c166736_g2	6.149027	0.324241	6.61E-15	5.22E-13
c166737_g3	6.455541	-1.32421	1.29E-05	0.00021358
c166745_g1	7.724949	-0.30325	1.40E-09	5.03E-08
c166746_g1	6.222517	-1.49009	5.09E-05	0.000724089

c166752_g1	8.598671	0.48373	1.43E-10	6.07E-09
c166752_g2	9.99777	1.844479	1.69E-17	1.86E-15
c166769_g1	6.772799	-1.08439	7.60E-07	1.63E-05
c166770_g1	7.258652	-0.69529	1.34E-08	4.01E-07
c166775_g2	7.477806	-0.50651	4.88E-10	1.89E-08
c166777_g1	6.713213	-1.12978	1.77E-06	3.54E-05
c166778_g1	6.843765	-1.03733	3.71E-06	6.97E-05
c166781_g1	6.384152	-1.37694	2.53E-05	0.000389563
c166783_g1	6.463168	-1.32266	2.99E-05	0.00045029
c166789_g1	7.170059	-0.76654	1.75E-08	5.14E-07
c166797_g1	6.827676	-1.04109	4.37E-07	9.86E-06
c166808_g1	8.36076	0.275274	1.97E-14	1.45E-12
c166814_g1	6.775442	-1.08364	9.83E-07	2.07E-05
c166821_g1	7.688836	-0.3314	4.42E-10	1.73E-08
c166828_g3	7.000215	-0.91323	1.16E-06	2.40E-05
c166833_g1	6.741092	-1.12447	3.86E-05	0.000565304
c166834_g1	6.216872	-1.49166	5.33E-05	0.000750756
c166835_g2	7.397933	-0.5705	3.87E-09	1.30E-07
c166837_g1	2.583926	2.10468	2.00E-12	1.13E-10
c166837_g3	3.008429	5.508504	9.77E-34	5.74E-31
c166849_g5	-2.19194	3.609089	1.44E-15	1.23E-13
c166859_g1	6.388167	-1.37617	3.87E-05	0.000565886
c166868_g4	7.087943	-0.83762	1.22E-07	3.08E-06
c166872_g1	6.38817	-1.37617	3.88E-05	0.000565886
c166886_g1	6.526726	-1.27255	9.77E-06	0.000166512
c166889_g1	7.091078	-0.83689	2.18E-07	5.21E-06
c166890_g1	6.882818	-0.99829	2.85E-07	6.64E-06
c166893_g1	7.647189	-0.3621	4.61E-11	2.10E-09
c166894_g1	8.045993	-0.01681	3.28E-11	1.52E-09
c166898_g1	6.452761	-1.32498	1.07E-05	0.000179713
c166902_g1	8.72712	0.620666	2.11E-18	2.57E-16
c166907_g1	8.381279	0.301912	6.83E-17	6.98E-15
c166908_g1	8.045994	-0.01681	3.29E-11	1.52E-09
c166918_g1	8.227715	0.158736	1.73E-15	1.46E-13
c166921_g2	7.620225	-0.38783	1.75E-10	7.32E-09
c166927_g2	2.703942	5.617667	1.44E-32	7.51E-30
c166927_g6	2.729936	1.616055	4.13E-12	2.23E-10
c166928_g5	6.858271	-1.03507	2.89E-05	0.000440219
c166929_g1	7.553194	-0.44499	2.70E-10	1.09E-08
c166935_g1	6.458939	-1.32344	1.84E-05	0.000292425
c166955_g1	4.478613	2.867714	7.67E-18	8.82E-16
c166962_g1	6.933713	-0.95742	1.64E-07	4.02E-06
c166963_g1	6.523751	-1.27332	7.53E-06	0.000131459
c166968_g1	7.553194	-0.44499	2.70E-10	1.09E-08
c166979_g1	6.822999	-1.04259	7.61E-07	1.63E-05
c166985_g2	7.087945	-0.83762	1.22E-07	3.08E-06
c166987_g1	6.895074	-0.9953	1.60E-06	3.24E-05
c166990_g4	6.782055	-1.08213	2.44E-06	4.73E-05
c166993_g1	6.775442	-1.08364	9.84E-07	2.07E-05
c166994_g1	7.993631	-0.06196	1.88E-11	9.11E-10
c166995_g1	7.074274	-0.84202	4.68E-08	1.28E-06
c166997_g3	6.993021	-0.91471	3.34E-07	7.72E-06
c166999_g2	7.367739	-0.59959	1.86E-09	6.55E-08
c167004_g1	6.530365	-1.27178	1.48E-05	0.000240602

c167005_g1	6.447408	-1.32653	1.31E-05	0.000216301
c167022_g1	2.003761	1.715426	2.06E-09	7.21E-08
c167027_g3	7.258644	-0.69529	1.32E-08	3.98E-07
c167030_g1	6.458941	-1.32344	1.84E-05	0.000292425
c167040_g22	-3.0688	-0.16212	2.09E-05	0.00032673
c167040_g7	-3.93533	-0.52953	5.26E-06	9.55E-05
c167044_g1	6.384159	-1.37694	2.54E-05	0.000389973
c167047_g2	2.522383	6.574204	7.35E-19	9.27E-17
c167054_g1	7.85124	-0.18267	2.60E-12	1.45E-10
c167063_g1	7.074275	-0.84202	4.67E-08	1.28E-06
c167068_g7	-2.25386	1.017709	1.47E-07	3.65E-06
c167079_g1	6.452761	-1.32498	1.07E-05	0.000179552
c167087_g1	11.17391	3.01913	9.00E-27	2.82E-24
c167087_g2	7.145128	-0.79798	5.64E-07	1.24E-05
c167098_g2	6.722144	-1.12751	2.99E-06	5.72E-05
c167114_g1	7.252452	-0.69744	6.66E-09	2.14E-07
c167115_g1	7.974609	-0.06975	2.31E-13	1.49E-11
c167120_g1	7.216018	-0.73014	1.67E-08	4.91E-07
c167134_g2	7.973238	-0.0704	2.90E-13	1.84E-11
c167135_g1	6.830099	-1.04034	5.20E-07	1.16E-05
c167139_g1	7.326488	-0.63291	4.04E-09	1.35E-07
c167141_g1	7.518549	-0.47441	3.58E-10	1.42E-08
c167142_g1	6.770399	-1.08515	1.08E-06	2.25E-05
c167146_g1	6.990019	-0.91545	2.04E-07	4.92E-06
c167149_g1	6.668311	-1.17278	1.23E-05	0.000204454
c167152_g1	7.094556	-0.83616	4.17E-07	9.48E-06
c167156_g1	6.368148	-1.38083	4.20E-05	0.000609738
c167161_g1	8.163161	0.099917	5.27E-15	4.20E-13
c167165_g1	6.521152	-1.27409	6.89E-06	0.000121585
c167166_g4	3.026207	-0.25918	7.74E-06	0.000134298
c167170_g2	7.476066	-0.50721	5.49E-10	2.11E-08
c167173_g1	9.718706	1.579144	1.07E-28	4.05E-26
c167176_g1	7.26111	-0.69457	2.10E-08	6.08E-07
c167177_g1	8.198204	0.12561	2.65E-13	1.70E-11
c167180_g1	6.598504	-1.22186	7.86E-06	0.00013588
c167185_g1	7.549584	-0.44638	1.53E-10	6.45E-09
c167188_g1	7.124938	-0.80309	2.39E-08	6.85E-07
c167193_g2	6.305478	-1.43244	3.53E-05	0.000522258
c167197_g2	6.388169	-1.37617	3.88E-05	0.000565886
c167203_g1	7.315043	-0.65764	1.83E-07	4.45E-06
c167212_g1	8.181312	0.107443	2.91E-12	1.60E-10
c167225_g1	7.677241	-0.33549	2.72E-11	1.29E-09
c167227_g1	6.710799	-1.13054	1.75E-06	3.52E-05
c167241_g1	6.598506	-1.22186	7.86E-06	0.000135917
c167246_g1	7.216018	-0.73014	1.67E-08	4.91E-07
c167249_g1	6.388169	-1.37617	3.88E-05	0.000565886
c167250_g1	4.974659	-0.69982	1.29E-07	3.22E-06
c167251_g1	7.742095	-0.28099	3.17E-11	1.48E-09
c167261_g1	6.944809	-0.95445	7.22E-07	1.56E-05
c167273_g1	7.420672	-0.56273	7.78E-09	2.45E-07
c167274_g1	7.624528	-0.38646	5.40E-10	2.08E-08
c167283_g1	8.431231	0.350377	1.60E-16	1.55E-14
c167284_g2	6.827676	-1.04109	4.38E-07	9.86E-06
c167289_g1	9.047955	0.930282	7.41E-24	1.70E-21

c167291_g1	8.626054	0.528959	1.79E-19	2.39E-17
c167298_g1	7.373216	-0.59746	2.19E-09	7.61E-08
c167299_g1	6.7108	-1.13054	1.75E-06	3.51E-05
c167301_g1	6.380977	-1.37772	1.92E-05	0.000303523
c167307_g2	6.705399	-1.13206	2.75E-06	5.28E-05
c167308_g2	7.170059	-0.76654	1.75E-08	5.13E-07
c167311_g1	7.161845	-0.76945	2.59E-08	7.38E-07
c167312_g1	7.686562	-0.33208	2.29E-10	9.33E-09
c167313_g1	7.715552	-0.30597	1.11E-10	4.75E-09
c167319_g1	7.050088	-0.87382	9.78E-07	2.07E-05
c167334_g1	7.814217	-0.21129	5.78E-12	3.04E-10
c167335_g1	7.455367	-0.5322	3.44E-09	1.16E-07
c167340_g1	6.52673	-1.27255	9.81E-06	0.000166514
c167351_g1	7.203648	-0.73447	1.94E-08	5.65E-07
c167358_g1	8.882612	0.768128	1.48E-19	2.00E-17
c167370_g3	7.078436	-0.84056	3.72E-08	1.04E-06
c167378_g1	9.029858	0.910983	1.99E-22	3.86E-20
c167379_g1	9.969957	1.82703	9.49E-33	5.17E-30
c167388_g1	7.085163	-0.83836	7.55E-08	1.98E-06
c167393_g1	9.238296	1.120274	7.01E-21	1.13E-18
c167395_g3	6.222518	-1.49009	5.10E-05	0.000724089
c167399_g1	9.289748	1.165541	3.92E-26	1.15E-23
c167404_g1	10.25702	2.106846	1.18E-26	3.61E-24
c167416_g1	7.290053	-0.6648	4.61E-09	1.52E-07
c167428_g1	6.8301	-1.04034	5.21E-07	1.16E-05
c167429_g1	8.074467	0.018761	3.10E-14	2.23E-12
c167436_g1	7.028296	-0.87972	6.78E-08	1.79E-06
c167440_g1	7.680602	-0.33413	4.82E-11	2.17E-09
c167444_g1	7.082704	-0.83909	5.24E-08	1.42E-06
c167452_g1	6.222518	-1.49009	5.10E-05	0.000724089
c167460_g2	6.993022	-0.91471	3.34E-07	7.72E-06
c167472_g1	6.51556	-1.27563	9.93E-06	0.000168158
c167476_g1	9.536879	1.397088	1.06E-21	1.89E-19
c167479_g2	7.297809	-0.66194	6.95E-09	2.21E-07
c167481_g1	7.085164	-0.83836	7.55E-08	1.98E-06
c167486_g1	8.665642	0.562934	4.02E-18	4.75E-16
c167487_g1	7.82805	-0.2053	7.93E-12	4.06E-10
c167489_g1	6.936004	-0.95668	1.95E-07	4.73E-06
c167509_g1	12.36315	4.213811	3.00E-82	2.21E-78
c167510_g1	7.955674	-0.08929	3.31E-13	2.08E-11
c167514_g3	8.639538	0.535758	1.91E-16	1.81E-14
c167517_g2	2.415717	0.811259	2.27E-08	6.56E-07
c167518_g1	7.746032	-0.27964	7.05E-11	3.10E-09
c167518_g2	6.588866	-1.22415	4.45E-06	8.17E-05
c167535_g1	7.224046	-0.72797	7.61E-08	1.99E-06
c167541_g2	4.826916	0.7541	2.32E-17	2.51E-15
c167544_g1	6.299841	-1.434	2.96E-05	0.00044803
c167552_g1	6.656495	-1.17507	3.98E-06	7.41E-05
c167556_g1	9.394286	1.261496	1.61E-24	3.96E-22
c167558_g1	6.862227	-1.00351	3.91E-06	7.31E-05
c167562_g1	6.608096	-1.22032	2.71E-05	0.000413964
c167565_g1	6.98739	-0.91619	1.41E-07	3.50E-06
c167571_g1	6.299839	-1.434	2.97E-05	0.000448392
c167580_g1	5.056337	-0.6286	1.24E-07	3.11E-06

c167581_g1	6.455541	-1.32421	1.29E-05	0.00021358
c167582_g1	7.54282	-0.44915	2.85E-10	1.15E-08
c167583_g1	7.338085	-0.62863	5.21E-09	1.70E-07
c167586_g1	8.789099	0.683444	3.95E-21	6.53E-19
c167590_g3	6.668313	-1.17278	1.23E-05	0.000204454
c167594_g2	6.708278	-1.1313	2.02E-06	4.00E-05
c167598_g1	8.445544	0.358065	7.36E-17	7.49E-15
c167600_g1	7.15006	-0.77235	2.54E-07	6.01E-06
c167606_g1	7.544572	-0.44846	1.55E-10	6.52E-09
c167612_g1	6.832825	-1.03958	7.16E-07	1.55E-05
c167615_g1	7.799362	-0.23032	1.32E-11	6.55E-10
c167621_g1	8.398302	0.310297	8.81E-15	6.84E-13
c167622_g1	7.085163	-0.83836	7.55E-08	1.98E-06
c167627_g1	7.902964	-0.1359	8.91E-13	5.33E-11
c167627_g2	6.305477	-1.43244	3.53E-05	0.000522258
c167632_g1	9.496883	1.363496	3.07E-28	1.12E-25
c167633_g1	2.170873	4.050001	3.09E-15	2.54E-13
c167634_g1	6.588865	-1.22415	4.44E-06	8.17E-05
c167637_g2	6.651148	-1.17659	2.83E-06	5.43E-05
c167639_g1	8.036279	-0.02003	9.44E-13	5.63E-11
c167646_g1	8.813081	0.702469	2.58E-19	3.38E-17
c167668_g4	6.450212	-1.32576	1.07E-05	0.000180255
c167690_g2	3.815569	-0.48126	1.06E-07	2.70E-06
c167701_g2	2.026311	2.460359	7.16E-11	3.15E-09
c167707_g1	7.411407	-0.56556	1.10E-09	4.05E-08
c167715_g1	6.580546	-1.22645	7.34E-06	0.000128839
c167717_g1	6.843766	-1.03733	3.71E-06	6.97E-05
c167727_g1	7.122954	-0.80382	2.31E-08	6.64E-07
c167732_g1	6.450212	-1.32576	1.07E-05	0.000180256
c167743_g1	9.216949	1.095673	1.43E-24	3.57E-22
c167745_g1	6.526733	-1.27255	9.82E-06	0.000166584
c167745_g2	6.455542	-1.32421	1.29E-05	0.00021358
c167759_g1	8.244377	0.175578	1.43E-15	1.22E-13
c167760_g1	7.737066	-0.28301	1.14E-11	5.71E-10
c167766_g2	7.071926	-0.84276	6.46E-08	1.72E-06
c167769_g1	7.124938	-0.80309	2.39E-08	6.85E-07
c167770_g1	7.669219	-0.33889	4.54E-11	2.07E-09
c167770_g2	7.297809	-0.66194	6.95E-09	2.21E-07
c167770_g3	6.941473	-0.95519	4.18E-07	9.49E-06
c167773_g1	12.33819	4.186757	2.49E-43	4.15E-40
c167773_g2	12.21544	4.06171	5.03E-25	1.30E-22
c167775_g1	6.891365	-0.99604	8.85E-07	1.88E-05
c167780_g1	8.554183	0.457496	8.85E-17	8.89E-15
c167785_g1	6.292844	-1.43556	5.57E-05	0.000779172
c167787_g1	7.60172	-0.412	7.87E-09	2.47E-07
c167788_g1	8.838567	0.727683	2.98E-20	4.44E-18
c167794_g1	10.2272	2.080102	1.06E-32	5.73E-30
c167795_g2	7.901652	-0.15116	2.10E-09	7.36E-08
c167800_g2	7.407726	-0.56697	1.16E-09	4.24E-08
c167817_g2	6.651148	-1.17659	2.83E-06	5.43E-05
c167820_g2	6.384154	-1.37694	2.53E-05	0.000389712
c167820_g3	7.608302	-0.39264	7.81E-11	3.41E-09
c167820_g4	8.054449	-0.00081	9.13E-14	6.19E-12
c167824_g1	7.477806	-0.50651	4.87E-10	1.89E-08

c167826_g1	8.271161	0.19772	8.66E-16	7.58E-14
c167839_g1	6.539859	-1.27024	3.20E-05	0.000478776
c167841_g1	7.738648	-0.28234	1.45E-11	7.13E-10
c167842_g1	7.17218	-0.76582	2.21E-08	6.39E-07
c167848_g1	5.145364	-0.56576	6.09E-08	1.63E-06
c167853_g1	7.371351	-0.59817	1.85E-09	6.54E-08
c167857_g1	6.72617	-1.12675	5.30E-06	9.58E-05
c167866_g1	2.654454	5.055953	2.58E-20	3.89E-18
c167867_g1	7.518548	-0.47441	3.57E-10	1.42E-08
c167868_g5	7.54282	-0.44915	2.85E-10	1.15E-08
c167869_g1	6.770399	-1.08515	1.08E-06	2.25E-05
c167872_g2	6.656495	-1.17507	3.98E-06	7.41E-05
c167873_g7	-2.13456	0.296779	5.25E-05	0.000742696
c167879_g2	6.384158	-1.37694	2.53E-05	0.000389906
c167885_g1	7.693797	-0.33004	1.45E-09	5.22E-08
c167891_g1	7.842496	-0.18664	3.10E-12	1.70E-10
c167897_g1	7.131835	-0.8009	6.08E-08	1.63E-06
c167899_g1	6.586361	-1.22492	4.41E-06	8.16E-05
c167910_g3	6.880546	-0.99904	2.59E-07	6.11E-06
c167917_g6	-2.76587	1.07054	1.76E-08	5.16E-07
c167925_g1	7.409524	-0.56626	1.29E-09	4.68E-08
c167936_g1	6.566943	-1.22874	4.02E-05	0.000584872
c167943_g1	6.931532	-0.95817	1.60E-07	3.93E-06
c167947_g3	7.487112	-0.50301	1.02E-09	3.78E-08
c167957_g1	6.944808	-0.95445	7.21E-07	1.56E-05
c167958_g1	-2.23262	1.511167	4.61E-08	1.26E-06
c167961_g2	7.900188	-0.13721	7.42E-13	4.49E-11
c167967_g1	7.034664	-0.87751	7.40E-08	1.95E-06
c167971_g1	8.004786	-0.05872	8.92E-10	3.34E-08
c167973_g1	7.168065	-0.76727	1.58E-08	4.68E-07
c167974_g1	6.98505	-0.91693	1.11E-07	2.81E-06
c167975_g1	9.601093	1.464156	9.10E-28	3.12E-25
c167977_g1	8.183025	0.118742	3.75E-15	3.07E-13
c167984_g1	9.095353	0.971165	9.77E-21	1.55E-18
c167986_g1	7.524981	-0.47232	1.52E-09	5.44E-08
c167993_g1	8.776206	0.670796	4.78E-21	7.84E-19
c167996_g1	6.388168	-1.37617	3.88E-05	0.000565886
c167999_g1	7.738648	-0.28234	1.45E-11	7.13E-10
c168002_g2	6.782056	-1.08213	2.44E-06	4.73E-05
c168006_g2	8.143028	0.080832	9.69E-15	7.51E-13
c168009_g2	7.250553	-0.69816	6.84E-09	2.19E-07
c168011_g1	7.263831	-0.69384	2.57E-08	7.34E-07
c168012_g1	-2.34047	3.107575	1.85E-06	3.69E-05
c168013_g3	3.024189	1.904194	3.28E-11	1.52E-09
c168019_g2	9.351943	1.221978	5.37E-26	1.55E-23
c168019_g5	7.580108	-0.4189	8.53E-11	3.71E-09
c168022_g3	7.122954	-0.80382	2.31E-08	6.64E-07
c168023_g1	7.076405	-0.84129	3.88E-08	1.08E-06
c168032_g1	8.055904	-0.00017	1.43E-13	9.43E-12
c168036_g1	7.411407	-0.56556	1.10E-09	4.05E-08
c168037_g1	7.221084	-0.72869	4.18E-08	1.15E-06
c168039_g2	6.22595	-1.4893	6.79E-05	0.000936129
c168041_g1	7.926789	-0.11372	4.78E-13	2.98E-11
c168045_g1	7.570718	-0.44013	1.85E-08	5.40E-07

c168048_g1	8.625116	0.528393	1.60E-19	2.15E-17
c168051_g1	6.880546	-0.99904	2.59E-07	6.11E-06
c168052_g2	7.266822	-0.69312	4.89E-08	1.33E-06
c168052_g3	6.523751	-1.27332	7.54E-06	0.000131459
c168057_g1	6.722142	-1.12751	2.99E-06	5.71E-05
c168058_g1	6.222518	-1.49009	5.09E-05	0.000724089
c168059_g1	6.823	-1.04259	7.61E-07	1.63E-05
c168062_g1	6.452762	-1.32498	1.07E-05	0.000179729
c168065_g1	7.273524	-0.69168	2.04E-07	4.91E-06
c168069_g1	10.37906	2.231598	3.82E-37	3.18E-34
c168070_g1	8.165595	0.10117	7.24E-15	5.68E-13
c168073_g1	6.825376	-1.04184	4.29E-07	9.71E-06
c168074_g1	8.922284	0.810699	2.59E-22	4.94E-20
c168075_g5	2.599453	4.390083	2.63E-27	8.73E-25
c168081_g1	9.619829	1.48155	1.46E-26	4.44E-24
c168082_g1	6.866354	-1.00277	2.05E-06	4.04E-05
c168083_g1	8.263229	0.18421	2.67E-13	1.71E-11
c168084_g1	6.775442	-1.08364	9.83E-07	2.07E-05
c168085_g1	6.94481	-0.95445	7.22E-07	1.56E-05
c168092_g1	7.678875	-0.33481	3.45E-11	1.59E-09
c168100_g1	6.645847	-1.17812	3.89E-06	7.29E-05
c168105_g1	7.766846	-0.25712	8.31E-12	4.25E-10
c168125_g1	8.260448	0.192199	4.04E-15	3.29E-13
c168141_g1	7.373216	-0.59746	2.18E-09	7.61E-08
c168144_g1	9.957958	1.815011	3.36E-32	1.72E-29
c168151_g1	6.875895	-1.00053	5.16E-07	1.15E-05
c168152_g1	8.444454	0.348076	5.29E-13	3.27E-11
c168155_g1	8.054382	0.010483	1.38E-11	6.81E-10
c168156_g1	9.447637	1.310428	1.48E-21	2.59E-19
c168158_g2	7.295735	-0.66266	5.22E-09	1.71E-07
c168159_g1	7.794508	-0.23233	4.77E-12	2.53E-10
c168159_g4	6.944812	-0.95445	7.23E-07	1.56E-05
c168165_g1	6.775441	-1.08364	9.83E-07	2.07E-05
c168178_g1	2.357067	2.801065	1.33E-08	4.00E-07
c168178_g2	7.527483	-0.47162	2.87E-09	9.82E-08
c168181_g1	6.765437	-1.08665	1.43E-06	2.91E-05
c168183_g1	7.544572	-0.44846	1.55E-10	6.52E-09
c168199_g1	7.191728	-0.73736	2.19E-07	5.23E-06
c168202_g2	7.455368	-0.5322	3.44E-09	1.16E-07
c168202_g3	4.43086	-1.12928	5.36E-05	0.000753766
c168203_g1	6.219734	-1.49087	4.66E-05	0.000670816
c168203_g2	6.782055	-1.08213	2.43E-06	4.73E-05
c168211_g1	6.673367	-1.17202	2.36E-05	0.000366239
c168212_g3	5.99876	4.254528	1.75E-31	8.53E-29
c168214_g1	9.144839	1.021767	8.87E-24	2.00E-21
c168234_g1	-4.21887	-1.02728	3.14E-05	0.000471207
c168239_g1	7.583418	-0.41752	1.08E-10	4.67E-09
c168244_g1	7.263832	-0.69384	2.57E-08	7.34E-07
c168246_g1	7.297808	-0.66194	6.94E-09	2.21E-07
c168252_g1	8.752086	0.646244	6.51E-20	9.22E-18
c168254_g1	7.546252	-0.44777	1.38E-10	5.86E-09
c168256_g1	8.268828	0.196493	7.84E-16	6.89E-14
c168260_g3	6.588865	-1.22415	4.44E-06	8.17E-05
c168264_g1	7.986064	-0.06456	1.12E-12	6.61E-11

c168268_g1	6.222518	-1.49009	5.10E-05	0.000724089
c168273_g1	8.079974	0.021311	1.27E-13	8.43E-12
c168288_g1	6.718691	-1.12827	1.87E-06	3.73E-05
c168306_g1	8.316173	0.236963	2.81E-15	2.33E-13
c168307_g1	8.284287	0.203248	2.12E-13	1.38E-11
c168307_g2	8.8537	0.74146	5.11E-20	7.36E-18
c168310_g1	7.88041	-0.15777	2.14E-12	1.21E-10
c168314_g1	7.455369	-0.5322	3.45E-09	1.16E-07
c168317_g1	7.091077	-0.83689	2.18E-07	5.21E-06
c168318_g1	7.699205	-0.32867	7.03E-09	2.23E-07
c168321_g1	7.373216	-0.59746	2.18E-09	7.61E-08
c168323_g2	6.511893	-1.2764	1.52E-05	0.000245676
c168327_g1	8.133764	0.06591	1.24E-12	7.24E-11
c168328_g3	6.591546	-1.22339	5.28E-06	9.56E-05
c168331_g2	6.526729	-1.27255	9.79E-06	0.000166512
c168333_g1	6.996429	-0.91397	6.02E-07	1.32E-05
c168333_g2	6.80087	-1.0791	3.35E-05	0.000499739
c168344_g2	7.25438	-0.69672	7.40E-09	2.34E-07
c168345_g1	6.642542	-1.17888	5.76E-06	0.000103407
c168347_g2	7.207967	-0.73303	1.09E-08	3.33E-07
c168349_g1	6.827676	-1.04109	4.38E-07	9.86E-06
c168349_g2	6.443948	-1.3273	1.91E-05	0.000301976
c168350_g1	9.134995	1.011795	2.59E-23	5.66E-21
c168356_g1	7.16406	-0.76872	1.92E-08	5.60E-07
c168360_g1	6.770399	-1.08515	1.08E-06	2.25E-05
c168361_g1	7.858084	-0.18001	1.26E-11	6.28E-10
c168370_g1	8.664491	0.562372	1.95E-18	2.39E-16
c168372_g3	2.259777	5.44252	7.11E-20	9.99E-18
c168375_g1	9.068441	0.946864	7.38E-22	1.34E-19
c168377_g1	8.2202	0.145397	2.68E-13	1.71E-11
c168388_g14	-2.55252	0.154243	1.67E-05	0.000268428
c168391_g1	9.298169	1.167805	3.16E-23	6.83E-21
c168397_g2	6.52673	-1.27255	9.80E-06	0.000166512
c168409_g1	8.286145	0.213488	5.19E-16	4.70E-14
c168410_g1	3.752769	4.693648	1.14E-24	2.89E-22
c168412_g1	6.222517	-1.49009	5.09E-05	0.000724089
c168412_g2	6.534756	-1.27101	1.71E-05	0.000273657
c168415_g1	9.303636	1.17488	3.87E-25	1.02E-22
c168428_g1	8.212401	0.142287	8.45E-15	6.57E-13
c168455_g1	7.129271	-0.80164	3.86E-08	1.07E-06
c168468_g2	8.207304	0.1398	2.76E-15	2.29E-13
c168472_g1	8.015007	-0.04084	4.04E-12	2.18E-10
c168474_g1	7.626916	-0.38577	1.04E-09	3.87E-08
c168477_g1	7.042841	-0.8753	2.66E-07	6.27E-06
c168478_g1	7.667388	-0.33957	6.99E-11	3.08E-09
c168479_g1	8.924557	0.807023	4.75E-19	6.07E-17
c168481_g1	8.419342	0.336784	2.40E-17	2.59E-15
c168489_g2	6.978481	-0.91915	1.27E-07	3.18E-06
c168492_g1	7.326489	-0.63291	4.03E-09	1.35E-07
c168496_g1	7.285942	-0.66624	7.93E-09	2.49E-07
c168502_g1	8.410451	0.324815	2.81E-16	2.63E-14
c168504_g1	6.305479	-1.43244	3.53E-05	0.000522258
c168505_g3	6.299841	-1.434	2.96E-05	0.00044803
c168509_g1	7.775919	-0.25376	6.39E-11	2.82E-09

c168512_g1	7.367739	-0.59959	1.85E-09	6.55E-08
c168515_g1	6.980742	-0.91841	1.04E-07	2.67E-06
c168519_g1	7.131833	-0.8009	6.07E-08	1.63E-06
c168520_g1	7.95714	-0.08863	4.72E-13	2.94E-11
c168528_g1	2.842751	1.45907	1.30E-13	8.62E-12
c168534_g1	7.373217	-0.59746	2.19E-09	7.62E-08
c168537_g1	7.555201	-0.4443	4.19E-10	1.65E-08
c168538_g1	7.045911	1.163069	1.23E-25	3.39E-23
c168539_g1	6.944807	-0.95445	7.21E-07	1.56E-05
c168544_g1	6.710799	-1.13054	1.76E-06	3.52E-05
c168550_g1	8.405756	0.322429	4.68E-17	4.88E-15
c168559_g1	7.000214	-0.91323	1.16E-06	2.40E-05
c168567_g1	8.596728	0.492497	1.18E-14	9.07E-13
c168575_g1	7.907519	-0.13392	1.77E-12	1.00E-10
c168579_g1	7.768441	-0.25645	1.11E-11	5.57E-10
c168588_g1	6.97848	-0.91915	1.27E-07	3.18E-06
c168590_g1	7.551332	-0.44569	1.92E-10	8.00E-09
c168591_g2	8.100747	0.041221	4.04E-14	2.86E-12
c168597_g1	6.380977	-1.37772	1.92E-05	0.000303523
c168599_g1	7.218414	-0.72941	2.51E-08	7.19E-07
c168600_g3	6.526727	-1.27255	9.78E-06	0.000166512
c168602_g1	6.827676	-1.04109	4.37E-07	9.86E-06
c168604_g1	9.314649	1.18095	1.65E-20	2.53E-18
c168605_g1	3.973132	2.466817	1.03E-26	3.18E-24
c168605_g5	5.29383	2.278553	1.87E-34	1.25E-31
c168606_g1	6.309242	-1.43166	5.11E-05	0.000724427
c168606_g2	6.715766	-1.12902	1.34E-06	2.75E-05
c168617_g1	8.145608	0.082091	1.82E-14	1.35E-12
c168622_g1	8.473549	0.379123	9.73E-15	7.53E-13
c168629_g1	6.980742	-0.91841	1.05E-07	2.67E-06
c168631_g1	7.947445	-0.09319	5.23E-13	3.23E-11
c168633_g1	8.998767	0.878881	2.70E-20	4.06E-18
c168639_g1	7.934146	-0.11045	1.46E-12	8.38E-11
c168645_g1	9.97437	1.831912	4.95E-34	3.09E-31
c168651_g2	6.523751	-1.27332	7.54E-06	0.000131459
c168652_g1	8.349935	0.270441	1.83E-16	1.75E-14
c168662_g1	7.076405	-0.84129	3.89E-08	1.08E-06
c168663_g1	8.772455	0.662956	2.51E-18	3.03E-16
c168669_g1	10.19592	2.050457	5.34E-36	4.25E-33
c168672_g1	9.763708	1.625274	1.40E-32	7.40E-30
c168683_g2	7.082704	-0.83909	5.23E-08	1.42E-06
c168690_g1	8.331222	0.252897	3.12E-16	2.89E-14
c168694_g2	7.369547	-0.59888	1.72E-09	6.12E-08
c168695_g1	6.225963	-1.4893	6.83E-05	0.000939196
c168696_g1	6.458941	-1.32344	1.84E-05	0.000292425
c168697_g1	7.835379	-0.18929	2.58E-11	1.22E-09
c168699_g1	7.825201	-0.22295	2.34E-08	6.74E-07
c168700_g1	6.730725	-1.12599	1.00E-05	0.00016921
c168701_g1	8.18912	0.121863	8.40E-15	6.54E-13
c168703_g1	6.888111	-0.99679	5.35E-07	1.18E-05
c168715_g1	6.715767	-1.12902	1.34E-06	2.75E-05
c168718_g1	7.824684	-0.20663	4.62E-12	2.46E-10
c168729_g2	6.305475	-1.43244	3.52E-05	0.000522258
c168729_g3	7.218415	-0.72941	2.51E-08	7.19E-07

c168734_g1	6.853129	-1.03583	1.43E-05	0.000233536
c168735_g1	9.346397	1.218303	1.15E-27	3.87E-25
c168740_g1	8.156776	0.086501	1.25E-12	7.31E-11
c168742_g1	6.222517	-1.49009	5.09E-05	0.000724089
c168742_g3	6.84829	-1.03658	7.24E-06	0.000127291
c168742_g4	7.858084	-0.18001	1.26E-11	6.28E-10
c168746_g1	9.198674	1.073938	1.40E-24	3.54E-22
c168748_g1	8.564352	0.469905	1.88E-18	2.32E-16
c168750_g1	8.927918	0.8086	6.41E-18	7.45E-16
c168753_g1	6.938551	-0.95593	2.67E-07	6.28E-06
c168755_g1	11.6343	3.483185	2.46E-49	6.36E-46
c168757_g2	7.308366	-0.65907	4.26E-08	1.17E-06
c168759_g1	7.698701	-0.31274	3.54E-11	1.63E-09
c168772_g1	8.610623	0.506877	2.24E-15	1.87E-13
c168775_g4	7.335971	-0.62934	3.67E-09	1.23E-07
c168776_g1	10.672	2.523258	1.49E-42	2.27E-39
c168778_g1	-2.41841	0.700668	1.14E-07	2.88E-06
c168786_g1	10.80825	2.658231	6.12E-41	7.71E-38
c168790_g1	8.144292	0.081462	1.26E-14	9.52E-13
c168798_g1	8.135642	0.066543	2.76E-12	1.53E-10
c168799_g1	5.276181	-0.45004	4.77E-09	1.57E-07
c168800_g1	6.782055	-1.08213	2.44E-06	4.73E-05
c168808_g1	7.479518	-0.50581	3.28E-10	1.31E-08
c168813_g1	8.215317	0.143531	2.91E-14	2.10E-12
c168814_g1	8.584635	0.487335	9.02E-18	1.02E-15
c168815_g1	8.159533	0.098037	7.18E-15	5.64E-13
c168818_g28	2.597476	7.97904	1.99E-30	9.12E-28
c168822_g1	7.647189	-0.3621	4.61E-11	2.10E-09
c168823_g1	8.570083	0.472782	3.94E-17	4.15E-15
c168824_g2	6.648636	-1.17736	3.06E-06	5.83E-05
c168827_g1	9.877902	1.738361	1.62E-35	1.25E-32
c168836_g1	6.21687	-1.49166	5.33E-05	0.000751019
c168838_g1	8.754152	0.647342	2.35E-19	3.11E-17
c168844_g1	7.080494	-0.83982	4.11E-08	1.14E-06
c168845_g1	8.873201	0.757252	3.74E-18	4.42E-16
c168851_g1	2.216654	4.249604	3.28E-14	2.35E-12
c168853_g1	6.882818	-0.99829	2.85E-07	6.64E-06
c168863_g1	7.458017	-0.5315	6.61E-09	2.12E-07
c168863_g3	8.567166	0.4627	1.44E-13	9.51E-12
c168865_g1	7.470151	-0.5093	1.55E-09	5.57E-08
c168868_g1	7.52498	-0.47232	1.52E-09	5.44E-08
c168875_g2	7.340408	-0.62792	5.70E-09	1.85E-07
c168879_g1	10.03827	1.894215	5.09E-33	2.86E-30
c168884_g5	2.278448	6.739979	5.54E-24	1.30E-21
c168887_g1	8.676002	0.567433	2.69E-15	2.23E-13
c168899_g1	8.311082	0.234528	3.86E-16	3.54E-14
c168911_g1	7.686562	-0.33208	2.29E-10	9.33E-09
c168912_g1	8.064429	0.003035	2.84E-12	1.57E-10
c168933_g1	8.032979	-0.02132	2.81E-13	1.79E-11
c168933_g2	9.408998	1.277284	2.12E-26	6.33E-24
c168933_g3	6.878297	-0.99978	2.75E-07	6.43E-06
c168938_g2	3.150947	3.894515	1.03E-15	8.98E-14
c168942_g2	7.955674	-0.08929	3.31E-13	2.08E-11
c168943_g1	7.897392	-0.13852	9.65E-13	5.75E-11

c168946_g1	9.470665	1.334574	1.29E-23	2.86E-21
c168947_g1	7.821661	-0.20796	2.85E-12	1.57E-10
c168948_g2	7.626917	-0.38577	1.04E-09	3.87E-08
c168949_g3	3.542567	1.763885	4.12E-14	2.90E-12
c168950_g1	7.842497	-0.18664	3.09E-12	1.69E-10
c168951_g1	9.590706	1.454754	7.55E-29	2.91E-26
c168955_g1	7.458018	-0.5315	6.61E-09	2.12E-07
c168956_g2	6.713213	-1.12978	1.77E-06	3.54E-05
c168957_g4	8.243608	0.165548	5.84E-13	3.59E-11
c168960_g1	6.656494	-1.17507	3.98E-06	7.41E-05
c168962_g1	6.938558	-0.95593	2.69E-07	6.32E-06
c168970_g1	8.055904	-0.00017	1.43E-13	9.43E-12
c168982_g1	6.594706	-1.22262	7.39E-06	0.000129276
c168987_g1	7.941619	-0.10783	2.01E-11	9.67E-10
c168991_g1	10.90962	5.839504	5.08E-86	4.37E-82
c168992_g1	7.082705	-0.83909	5.24E-08	1.42E-06
c168998_g1	9.603139	1.465419	1.00E-26	3.11E-24
c168999_g12	4.053228	0.352574	1.62E-12	9.23E-11
c168999_g3	3.916032	2.871656	1.08E-30	5.02E-28
c169002_g1	6.296784	-1.43478	3.72E-05	0.000545913
c169008_g2	2.936415	-0.56894	5.58E-05	0.000781252
c169012_g1	7.926789	-0.11372	4.78E-13	2.98E-11
c169013_g1	8.236963	0.16307	2.69E-14	1.97E-12
c169017_g1	8.606482	0.505163	1.71E-16	1.64E-14
c169024_g1	7.803139	-0.22899	2.94E-11	1.38E-09
c169030_g1	6.468227	-1.32189	3.68E-05	0.000541871
c169035_g5	2.74963	2.311327	1.24E-16	1.22E-14
c169035_g6	3.499092	0.296564	1.84E-10	7.66E-09
c169038_g1	9.216844	1.092475	1.10E-25	3.03E-23
c169041_g30	2.350362	3.949353	5.81E-21	9.47E-19
c169041_g47	-8.33043	0.392669	5.51E-13	3.40E-11
c169047_g1	8.529131	0.429342	2.25E-14	1.65E-12
c169053_g1	9.413541	1.279987	2.11E-24	5.17E-22
c169056_g1	6.521152	-1.27409	6.90E-06	0.000121585
c169056_g2	9.779209	3.872197	4.37E-55	1.41E-51
c169068_g1	8.096845	0.039317	1.88E-14	1.40E-12
c169068_g2	7.803139	-0.22899	2.94E-11	1.38E-09
c169069_g2	6.468234	-1.32189	3.69E-05	0.000542465
c169069_g4	7.775919	-0.25376	6.39E-11	2.82E-09
c169071_g1	8.299297	0.228444	3.02E-15	2.49E-13
c169072_g1	8.65582	0.550826	2.22E-16	2.09E-14
c169074_g1	7.487111	-0.50301	1.02E-09	3.78E-08
c169075_g1	10.82213	2.67211	3.00E-41	3.98E-38
c169076_g1	8.326731	0.250472	1.54E-16	1.50E-14
c169077_g1	7.039764	-0.87603	1.55E-07	3.83E-06
c169078_g1	7.805209	-0.22832	5.71E-11	2.55E-09
c169080_g1	7.418045	-0.56344	4.18E-09	1.40E-07
c169081_g1	8.3088	0.23331	2.46E-16	2.31E-14
c169082_g2	2.478375	4.81996	5.47E-17	5.66E-15
c169082_g4	3.026209	1.002081	2.08E-10	8.61E-09
c169084_g1	9.622643	1.483223	4.44E-25	1.16E-22
c169085_g1	9.032583	0.912509	1.33E-21	2.33E-19
c169089_g3	6.656494	-1.17507	3.98E-06	7.41E-05
c169090_g1	9.212085	1.085885	7.41E-24	1.70E-21

c169100_g1	7.687965	-0.31613	1.07E-09	3.94E-08
c169102_g2	6.718691	-1.12827	1.87E-06	3.73E-05
c169104_g1	6.450212	-1.32576	1.07E-05	0.000180295
c169108_g1	8.572674	0.473934	1.94E-16	1.84E-14
c169108_g3	7.524981	-0.47232	1.52E-09	5.44E-08
c169109_g1	9.282919	1.155043	3.20E-25	8.57E-23
c169112_g1	8.011378	-0.04213	1.02E-12	6.06E-11
c169117_g1	8.508311	0.411809	4.65E-15	3.74E-13
c169119_g1	7.82022	-0.20863	2.64E-12	1.47E-10
c169121_g1	7.080495	-0.83982	4.12E-08	1.14E-06
c169126_g1	9.103996	0.984685	1.42E-24	3.56E-22
c169127_g1	8.266557	0.195266	7.70E-16	6.79E-14
c169130_g1	7.987817	-0.06391	2.12E-12	1.19E-10
c169130_g2	6.447409	-1.32653	1.31E-05	0.000216236
c169131_g1	-2.35882	0.900448	2.70E-05	0.000412827
c169139_g2	6.993023	-0.91471	3.35E-07	7.73E-06
c169139_g3	6.99002	-0.91545	2.04E-07	4.92E-06
c169139_g4	6.659825	-1.17431	3.91E-06	7.31E-05
c169141_g1	9.697144	1.559998	8.27E-32	4.07E-29
c169142_g1	6.656495	-1.17507	3.98E-06	7.41E-05
c169153_g2	7.315042	-0.65764	1.83E-07	4.45E-06
c169162_g1	7.428117	-0.54134	1.10E-08	3.34E-07
c169163_g1	11.18137	3.031707	9.03E-54	2.75E-50
c169163_g2	9.631625	1.491636	1.11E-24	2.82E-22
c169170_g1	7.087944	-0.83762	1.22E-07	3.08E-06
c169170_g2	7.702061	-0.31139	1.87E-11	9.07E-10
c169174_g2	2.922443	1.074789	2.22E-12	1.25E-10
c169177_g1	6.982881	-0.91767	1.00E-07	2.57E-06
c169179_g2	7.046295	-0.87456	4.97E-07	1.11E-05
c169183_g1	7.921526	-0.12932	2.22E-10	9.11E-09
c169195_g1	6.726168	-1.12675	5.30E-06	9.58E-05
c169201_g1	8.814528	0.708395	2.53E-21	4.27E-19
c169202_g4	-2.06141	4.113181	8.83E-10	3.31E-08
c169202_g7	2.756101	6.105195	1.08E-35	8.49E-33
c169205_g1	7.635347	-0.36689	1.40E-10	5.93E-09
c169208_g1	8.323839	0.24001	9.54E-14	6.43E-12
c169211_g4	6.521152	-1.27409	6.89E-06	0.000121585
c169214_g1	6.987389	-0.91619	1.41E-07	3.50E-06
c169218_g1	8.070682	0.016849	3.09E-14	2.23E-12
c169225_g1	7.308366	-0.65907	4.26E-08	1.17E-06
c169227_g1	8.939019	0.82012	1.25E-18	1.55E-16
c169230_g1	-2.75358	1.856599	2.84E-09	9.71E-08
c169231_g2	7.914931	-0.13129	2.15E-11	1.03E-09
c169232_g1	13.05297	4.903194	4.63E-60	2.00E-56
c169235_g1	7.295735	-0.66266	5.21E-09	1.71E-07
c169240_g1	7.967851	-0.08472	1.94E-11	9.34E-10
c169253_g1	3.347364	8.712744	3.12E-24	7.47E-22
c169260_g1	7.161845	-0.76945	2.59E-08	7.38E-07
c169263_g2	8.345521	0.268027	1.10E-16	1.09E-14
c169263_g3	6.888111	-0.99679	5.35E-07	1.18E-05
c169268_g1	8.654502	0.550262	9.42E-17	9.40E-15
c169271_g1	7.845479	-0.18532	1.89E-12	1.07E-10
c169272_g1	7.258645	-0.69529	1.33E-08	3.98E-07
c169273_g1	7.57315	-0.42166	3.04E-10	1.22E-08

c169274_g1	2.12226	4.53553	4.92E-20	7.12E-18
c169276_g4	2.136668	1.938822	6.33E-11	2.80E-09
c169281_g1	7.458016	-0.5315	6.60E-09	2.12E-07
c169282_g1	8.666829	0.563496	8.69E-18	9.92E-16
c169284_g1	9.057831	0.940322	4.77E-24	1.13E-21
c169286_g1	8.476636	0.388805	7.61E-18	8.76E-16
c169289_g1	8.875748	0.76441	1.73E-21	2.99E-19
c169289_g2	6.384157	-1.37694	2.53E-05	0.000389795
c169290_g1	7.883867	-0.15645	6.17E-12	3.22E-10
c169299_g2	6.222517	-1.49009	5.09E-05	0.000724089
c169304_g1	7.524982	-0.47232	1.52E-09	5.45E-08
c169305_g2	9.244828	3.333739	9.34E-63	4.83E-59
c169306_g1	7.377374	-0.59604	3.01E-09	1.02E-07
c169306_g2	8.505251	0.417695	6.68E-18	7.74E-16
c169314_g1	8.822388	0.713246	8.80E-21	1.41E-18
c169315_g1	11.00595	2.855398	3.58E-43	5.79E-40
c169317_g1	8.635624	0.540106	4.85E-19	6.20E-17
c169320_g1	7.302583	-0.66051	1.19E-08	3.60E-07
c169320_g6	6.782055	-1.08213	2.43E-06	4.73E-05
c169323_g1	7.359166	-0.60242	8.14E-09	2.55E-07
c169325_g1	7.680602	-0.33413	4.82E-11	2.17E-09
c169328_g1	7.503859	-0.47998	1.25E-09	4.53E-08
c169336_g1	7.252452	-0.69744	6.67E-09	2.14E-07
c169337_g1	9.621556	1.485378	6.36E-30	2.81E-27
c169341_g1	9.688928	1.546842	7.71E-24	1.76E-21
c169344_g1	8.462788	0.374406	3.80E-17	4.02E-15
c169346_g1	8.332425	0.253503	4.71E-16	4.28E-14
c169352_g1	7.48307	-0.50441	4.64E-10	1.81E-08
c169364_g1	7.491873	-0.50161	3.19E-09	1.08E-07
c169365_g1	7.291934	-0.66409	4.23E-09	1.41E-07
c169367_g3	-2.18948	2.277095	2.91E-05	0.000441924
c169368_g1	7.420671	-0.56273	7.77E-09	2.45E-07
c169374_g1	8.624183	0.527827	1.59E-19	2.14E-17
c169376_g1	8.272405	0.187297	1.45E-11	7.13E-10
c169381_g1	7.982838	-0.06586	3.84E-13	2.41E-11
c169381_g3	6.827676	-1.04109	4.37E-07	9.86E-06
c169384_g1	11.09846	2.948797	3.67E-51	1.06E-47
c169385_g1	10.03776	1.893861	2.24E-33	1.27E-30
c169386_g1	7.677241	-0.33549	2.73E-11	1.29E-09
c169386_g2	8.561103	0.460386	4.54E-15	3.67E-13
c169387_g1	7.986063	-0.06456	1.12E-12	6.61E-11
c169390_g1	7.734039	-0.28436	9.84E-12	4.96E-10
c169390_g2	7.008672	-0.91174	4.61E-06	8.43E-05
c169394_g1	7.120941	-0.80455	2.57E-08	7.33E-07
c169398_g1	9.442759	1.30775	6.96E-24	1.61E-21
c169404_g1	8.452174	0.368516	2.69E-17	2.90E-15
c169405_g1	9.222185	1.095832	4.64E-24	1.10E-21
c169408_g1	14.45495	6.306782	1.22E-96	1.58E-92
c169415_g2	7.030414	-0.87898	6.03E-08	1.62E-06
c169419_g1	7.338085	-0.62863	5.20E-09	1.70E-07
c169421_g1	9.458328	1.323912	3.68E-25	9.75E-23
c169422_g2	7.542819	-0.44915	2.86E-10	1.15E-08
c169423_g1	6.216872	-1.49166	5.33E-05	0.000750756
c169423_g2	7.137875	-0.79944	1.90E-07	4.61E-06

c169427_g1	9.194324	1.067237	3.85E-22	7.18E-20
c169430_g2	11.01569	2.864355	2.99E-39	3.29E-36
c169433_g1	8.974494	0.860097	6.50E-23	1.36E-20
c169439_g1	8.527851	0.436825	2.26E-18	2.74E-16
c169443_g1	8.308565	0.223274	1.17E-12	6.85E-11
c169449_g2	7.474238	-0.50791	6.99E-10	2.65E-08
c169452_g1	6.718691	-1.12827	1.87E-06	3.73E-05
c169454_g1	6.378291	-1.3785	1.75E-05	0.000280861
c169460_g5	9.672081	1.537794	1.85E-31	8.96E-29
c169463_g1	7.790106	-0.23434	4.52E-12	2.41E-10
c169465_g1	7.054161	-0.87308	1.97E-06	3.91E-05
c169469_g1	7.211842	-0.73158	1.04E-08	3.21E-07
c169471_g1	8.207303	0.1398	2.76E-15	2.29E-13
c169475_g1	6.384155	-1.37694	2.53E-05	0.000389734
c169477_g1	8.21688	0.144153	5.88E-14	4.06E-12
c169479_g1	7.034665	-0.87751	7.41E-08	1.95E-06
c169481_g1	6.588865	-1.22415	4.43E-06	8.16E-05
c169485_g1	7.874488	-0.16041	1.15E-12	6.76E-11
c169487_g1	9.446229	1.315928	2.65E-29	1.07E-26
c169492_g1	9.029253	0.899062	1.65E-14	1.23E-12
c169492_g2	9.196784	1.063404	4.45E-17	4.68E-15
c169493_g1	8.247261	0.166788	2.49E-12	1.39E-10
c169495_g1	7.132828	1.243185	3.37E-23	7.23E-21
c169495_g2	8.605911	2.695857	4.29E-46	9.25E-43
c169499_g1	8.077099	0.020036	5.17E-14	3.62E-12
c169500_g1	6.713212	-1.12978	1.76E-06	3.54E-05
c169501_g9	2.950794	3.793247	6.16E-27	1.97E-24
c169504_g1	7.411407	-0.56556	1.10E-09	4.05E-08
c169505_g1	6.944809	-0.95445	7.22E-07	1.56E-05
c169506_g1	7.587072	-0.41614	2.12E-10	8.76E-09
c169513_g1	6.933713	-0.95742	1.64E-07	4.02E-06
c169516_g1	8.772722	0.668616	6.79E-21	1.10E-18
c169519_g1	7.887758	-0.15513	2.32E-11	1.10E-09
c169524_g1	7.071927	-0.84276	6.45E-08	1.71E-06
c169537_g1	7.883866	-0.15645	6.15E-12	3.21E-10
c169538_g17	2.163383	4.562203	1.73E-10	7.23E-09
c169539_g1	7.735541	-0.28369	1.00E-11	5.03E-10
c169563_g1	9.501033	1.366116	3.01E-26	8.84E-24
c169565_g1	8.311082	0.234528	3.86E-16	3.54E-14
c169568_g2	7.468422	1.573879	5.35E-32	2.68E-29
c169570_g1	8.034584	-0.02067	4.98E-13	3.09E-11
c169571_g1	8.516743	0.424098	2.16E-17	2.34E-15
c169573_g18	9.31647	1.181884	9.26E-20	1.28E-17
c169573_g5	7.373216	-0.59746	2.18E-09	7.61E-08
c169573_g9	10.59921	2.450227	2.92E-39	3.28E-36
c169575_g4	2.44072	2.512121	6.47E-16	5.76E-14
c169576_g1	7.221087	-0.72869	4.19E-08	1.15E-06
c169579_g1	8.673251	0.566308	5.79E-16	5.20E-14
c169583_g1	9.031654	0.912	6.25E-22	1.14E-19
c169585_g1	6.993025	-0.91471	3.35E-07	7.73E-06
c169585_g2	7.252452	-0.69744	6.67E-09	2.14E-07
c169587_g2	6.530366	-1.27178	1.48E-05	0.000240602
c169588_g1	8.738094	0.638008	2.03E-19	2.71E-17
c169590_g2	-2.89371	-0.0986	3.59E-06	6.77E-05

c169591_g1	7.883867	-0.15645	6.16E-12	3.22E-10
c169595_g1	7.428117	-0.54134	1.09E-08	3.34E-07
c169598_g3	-2.10727	0.169653	2.91E-05	0.00044215
c169599_g1	8.688007	0.587338	5.33E-20	7.66E-18
c169604_g1	7.026002	-0.88045	8.80E-08	2.28E-06
c169604_g2	7.293801	-0.66337	4.43E-09	1.47E-07
c169611_g1	7.684432	-0.33276	1.26E-10	5.37E-09
c169616_g1	10.35159	2.207839	5.91E-42	8.27E-39
c169617_g1	6.296784	-1.43478	3.72E-05	0.000545913
c169624_g1	8.488494	0.401871	7.99E-18	9.14E-16
c169625_g1	8.145608	0.082091	1.82E-14	1.35E-12
c169637_g2	8.907882	0.792761	4.31E-20	6.30E-18
c169638_g1	7.907518	-0.13392	1.76E-12	1.00E-10
c169652_g1	10.15105	2.002984	3.61E-28	1.29E-25
c169661_g1	7.263831	-0.69384	2.57E-08	7.34E-07
c169668_g1	7.334022	-0.63006	2.92E-09	9.96E-08
c169670_g1	7.256422	-0.69601	9.37E-09	2.91E-07
c169672_g1	10.14056	1.997143	4.56E-39	4.81E-36
c169675_g1	8.388901	0.306102	1.17E-16	1.16E-14
c169677_g1	11.6245	3.473038	3.97E-46	8.92E-43
c169678_g1	8.198205	0.12561	2.66E-13	1.70E-11
c169684_g1	10.64169	2.496171	1.61E-45	3.21E-42
c169691_g1	9.192548	1.066268	6.96E-23	1.44E-20
c169694_g1	7.227283	-0.72724	1.08E-07	2.75E-06
c169705_g1	7.8528	-0.182	2.59E-12	1.45E-10
c169715_g2	8.333677	0.254109	7.73E-16	6.81E-14
c169715_g3	7.437736	-0.53853	1.21E-09	4.40E-08
c169716_g1	7.724949	-0.30325	1.40E-09	5.03E-08
c169722_g1	7.170059	-0.76654	1.75E-08	5.14E-07
c169726_g1	8.671062	0.578423	1.61E-16	1.56E-14
c169730_g3	8.841716	0.729295	2.34E-19	3.11E-17
c169737_g1	8.196519	0.124985	1.25E-13	8.32E-12
c169740_g1	8.970135	0.853075	2.78E-21	4.68E-19
c169744_g1	7.039765	-0.87603	1.55E-07	3.83E-06
c169744_g2	8.386653	0.304905	6.17E-17	6.37E-15
c169745_g5	3.595888	2.485647	2.97E-05	0.000449059
c169746_g1	11.56087	6.49092	7.31E-104	1.89E-99
c169749_g1	8.181824	0.118119	4.37E-15	3.55E-13
c169749_g2	6.882818	-0.99829	2.85E-07	6.64E-06
c169751_g1	7.4974	-0.5002	1.30E-08	3.91E-07
c169754_g2	7.54095	-0.44985	4.01E-10	1.58E-08
c169755_g1	7.601721	-0.412	7.88E-09	2.47E-07
c169756_g2	6.452761	-1.32498	1.07E-05	0.000179552
c169759_g1	7.443199	-0.53642	6.93E-10	2.63E-08
c169768_g6	-2.08489	1.00001	1.70E-06	3.43E-05
c169775_g1	8.36841	0.287776	1.07E-16	1.06E-14
c169775_g2	6.375579	-1.37927	1.96E-05	0.00030825
c169780_g1	8.261536	0.183593	1.21E-13	8.06E-12
c169782_g1	7.717702	-0.30529	2.06E-10	8.54E-09
c169783_g1	6.388167	-1.37617	3.87E-05	0.000565886
c169783_g2	7.824684	-0.20663	4.62E-12	2.46E-10
c169792_g1	7.79604	-0.23166	6.05E-12	3.16E-10
c169794_g20	2.057691	2.874939	9.67E-09	2.99E-07
c169800_g1	8.563322	0.46933	1.29E-18	1.60E-16

c169801_g1	10.67026	2.520092	1.36E-36	1.12E-33
c169805_g1	8.246697	0.17681	1.08E-15	9.39E-14
c169806_g1	8.395349	0.309098	1.81E-15	1.52E-13
c169809_g1	9.029293	0.905302	2.24E-18	2.72E-16
c169809_g2	8.11041	0.045033	1.28E-12	7.44E-11
c169810_g2	6.726167	-1.12675	5.29E-06	9.58E-05
c169817_g2	11.37406	3.223046	3.38E-47	7.96E-44
c169817_g3	6.380976	-1.37772	1.92E-05	0.000303523
c169818_g1	7.423509	-0.56202	1.54E-08	4.57E-07
c169821_g1	8.736022	0.631586	4.16E-20	6.11E-18
c169823_g2	6.309242	-1.43166	5.11E-05	0.000724427
c169824_g3	10.40797	2.257403	3.20E-29	1.28E-26
c169826_g1	9.595423	1.45771	2.08E-26	6.24E-24
c169828_g1	8.354905	0.272857	1.32E-15	1.13E-13
c169829_g3	7.413434	-0.56485	1.56E-09	5.58E-08
c169834_g1	8.353583	0.272253	7.17E-16	6.35E-14
c169835_g1	7.901566	-0.13655	7.72E-13	4.66E-11
c169835_g2	6.708278	-1.1313	2.03E-06	4.00E-05
c169837_g1	7.67093	-0.33821	3.28E-11	1.52E-09
c169838_g1	9.343155	1.216006	7.87E-28	2.71E-25
c169840_g1	9.036497	0.914543	3.73E-20	5.52E-18
c169843_g1	7.60652	-0.39333	1.09E-10	4.69E-09
c169847_g6	2.311892	4.203546	5.23E-14	3.65E-12
c169848_g1	8.587069	0.488482	3.93E-17	4.15E-15
c169849_g1	10.48693	2.340098	6.54E-44	1.13E-40
c169851_g1	8.976894	0.861645	8.51E-23	1.74E-20
c169870_g2	9.215522	1.08781	2.04E-22	3.94E-20
c169871_g2	7.407726	-0.56697	1.16E-09	4.24E-08
c169874_g1	10.40698	2.259376	1.07E-37	9.41E-35
c169879_g1	7.549585	-0.44638	1.54E-10	6.48E-09
c169887_g4	8.422211	0.329591	1.09E-13	7.26E-12
c169889_g1	8.220201	0.145397	2.68E-13	1.71E-11
c169889_g2	8.810972	0.701386	5.82E-20	8.28E-18
c169891_g1	9.764813	1.626064	4.45E-32	2.26E-29
c169892_g1	7.734039	-0.28436	9.84E-12	4.96E-10
c169892_g2	8.845379	0.736645	1.10E-21	1.95E-19
c169897_g2	7.487111	-0.50301	1.02E-09	3.78E-08
c169898_g2	6.817269	-1.04409	1.53E-06	3.11E-05
c169900_g1	11.77067	3.620227	6.46E-58	2.57E-54
c169904_g3	8.306785	0.222662	4.98E-13	3.09E-11
c169904_g4	7.7505	-0.27828	2.83E-10	1.14E-08
c169904_g6	7.691252	-0.33072	6.78E-10	2.58E-08
c169909_g1	8.876652	0.764941	2.83E-21	4.75E-19
c169913_g1	8.070682	0.016849	3.10E-14	2.23E-12
c169916_g1	8.036278	-0.02003	9.43E-13	5.63E-11
c169917_g12	2.428198	0.597315	6.56E-08	1.74E-06
c169918_g1	9.417105	1.285555	5.35E-27	1.72E-24
c169918_g2	6.468233	-1.32189	3.68E-05	0.00054236
c169918_g4	7.407726	-0.56697	1.16E-09	4.24E-08
c169920_g1	9.135851	1.012288	5.42E-23	1.14E-20
c169921_g1	7.794508	-0.23233	4.76E-12	2.53E-10
c169922_g1	6.941473	-0.95519	4.18E-07	9.49E-06
c169923_g1	8.293089	0.217156	1.03E-15	8.99E-14
c169923_g2	6.993021	-0.91471	3.34E-07	7.72E-06

c169927_g1	7.361609	-0.60171	4.80E-09	1.58E-07
c169927_g2	8.347698	0.269234	1.16E-16	1.15E-14
c169928_g1	8.497815	0.40713	1.33E-17	1.47E-15
c169931_g1	8.605874	0.511439	4.61E-19	5.94E-17
c169932_g1	5.381142	-0.36365	1.21E-09	4.41E-08
c169934_g2	8.780815	0.673522	3.25E-20	4.83E-18
c169935_g1	7.648895	-0.36142	6.15E-11	2.73E-09
c169937_g1	9.171891	1.045883	2.87E-22	5.45E-20
c169939_g1	7.950642	2.05179	2.38E-34	1.58E-31
c169939_g2	10.81706	2.664885	1.71E-32	8.83E-30
c169946_g1	6.775443	-1.08364	9.84E-07	2.07E-05
c169946_g2	6.933713	-0.95742	1.64E-07	4.02E-06
c169947_g1	9.616458	1.479457	2.40E-28	8.94E-26
c169948_g1	8.187837	0.121238	5.86E-15	4.65E-13
c169954_g9	2.97434	0.860265	5.81E-11	2.58E-09
c169958_g2	7.518549	-0.47441	3.58E-10	1.42E-08
c169958_g3	7.510049	-0.4974	2.10E-07	5.04E-06
c169958_g5	7.365853	-0.6003	2.27E-09	7.87E-08
c169961_g1	7.589111	-0.41545	3.44E-10	1.37E-08
c169962_g1	8.081528	0.021949	2.25E-13	1.46E-11
c169970_g1	9.166213	1.038029	4.34E-20	6.32E-18
c169971_g1	7.087944	-0.83762	1.22E-07	3.08E-06
c169971_g2	9.158786	1.034113	5.12E-23	1.08E-20
c169972_g2	8.459501	0.372639	1.26E-17	1.40E-15
c169978_g1	8.039926	-0.01874	2.96E-12	1.63E-10
c169979_g2	7.817325	-0.20996	3.14E-12	1.72E-10
c169986_g19	3.608161	0.881661	4.33E-12	2.32E-10
c169991_g1	6.455543	-1.32421	1.29E-05	0.00021358
c169991_g2	7.885757	-0.15579	1.16E-11	5.80E-10
c169993_g1	8.571359	0.473358	8.61E-17	8.68E-15
c169995_g1	6.98505	-0.91693	1.11E-07	2.81E-06
c170004_g1	12.92404	4.775339	9.59E-108	4.96E-103
c170009_g2	10.03317	1.891793	4.72E-38	4.31E-35
c170009_g3	8.125469	0.062746	5.47E-14	3.79E-12
c170013_g3	7.514961	-0.4758	2.17E-10	8.89E-09
c170015_g1	9.172801	1.046371	6.83E-22	1.24E-19
c170017_g1	8.63105	0.531791	1.18E-18	1.46E-16
c170018_g7	2.290406	3.033158	1.63E-15	1.39E-13
c170022_g1	7.170058	-0.76654	1.75E-08	5.13E-07
c170022_g6	8.096845	0.039317	1.89E-14	1.40E-12
c170024_g1	8.972075	0.854111	1.26E-20	1.96E-18
c170025_g1	8.411749	0.325412	5.35E-16	4.83E-14
c170028_g1	9.864188	1.721103	2.81E-28	1.04E-25
c170030_g2	7.771935	-0.2551	2.66E-11	1.25E-09
c170030_g3	8.388902	0.306102	1.17E-16	1.16E-14
c170035_g3	7.763832	-0.25846	6.42E-12	3.33E-10
c170035_g4	8.898573	0.782086	1.10E-18	1.37E-16
c170040_g1	6.305475	-1.43244	3.52E-05	0.000522258
c170040_g2	9.306826	1.176748	6.47E-24	1.51E-21
c170047_g2	9.181042	1.059001	1.26E-25	3.45E-23
c170051_g1	8.801939	0.690505	2.34E-18	2.83E-16
c170052_g1	8.534307	0.440305	2.00E-17	2.18E-15
c170059_g1	8.169583	0.103051	2.44E-14	1.79E-12
c170059_g2	7.382331	-0.59462	8.87E-09	2.76E-07

c170069_g1	7.131833	-0.8009	6.07E-08	1.63E-06
c170069_g2	7.743999	-0.28031	5.34E-11	2.40E-09
c170069_g3	7.642406	-0.36415	3.79E-11	1.74E-09
c170070_g1	7.183257	-0.76291	1.67E-07	4.10E-06
c170070_g2	7.740317	-0.28166	2.04E-11	9.81E-10
c170077_g1	9.891808	1.745595	3.56E-24	8.49E-22
c170078_g1	10.01205	1.864903	5.69E-26	1.64E-23
c170079_g1	9.36572	1.233783	2.99E-24	7.22E-22
c170080_g3	8.514464	0.422933	7.26E-18	8.40E-16
c170081_g1	8.374558	0.290783	1.24E-15	1.07E-13
c170081_g3	9.461154	1.328827	6.15E-28	2.15E-25
c170090_g1	9.227673	1.102842	9.64E-26	2.70E-23
c170092_g2	8.884804	0.769191	8.13E-19	1.02E-16
c170093_g1	7.046294	-0.87456	4.97E-07	1.11E-05
c170093_g2	7.483071	-0.50441	4.64E-10	1.81E-08
c170095_g1	10.36489	2.216153	8.20E-33	4.51E-30
c170104_g1	10.24069	2.094128	1.13E-34	7.99E-32
c170107_g1	9.117094	0.992624	2.21E-21	3.77E-19
c170107_g2	8.235461	0.16245	1.38E-14	1.04E-12
c170109_g1	8.423535	0.339159	2.73E-17	2.93E-15
c170111_g1	10.51165	2.364037	9.43E-42	1.28E-38
c170119_g1	8.789099	0.683444	3.95E-21	6.53E-19
c170121_g9	-2.06944	3.45064	9.43E-16	8.24E-14
c170126_g1	9.388896	1.260824	3.29E-28	1.19E-25
c170127_g1	9.268484	1.138726	1.25E-22	2.48E-20
c170132_g1	8.297127	0.21899	5.84E-15	4.64E-13
c170137_g1	9.113101	3.203304	3.98E-66	2.57E-62
c170146_g1	8.961099	0.842875	5.04E-20	7.28E-18
c170146_g2	6.651148	-1.17659	2.83E-06	5.43E-05
c170157_g1	7.958685	-0.08798	7.38E-13	4.47E-11
c170157_g2	7.909216	-0.13327	3.00E-12	1.65E-10
c170165_g1	7.907519	-0.13392	1.77E-12	1.00E-10
c170181_g1	10.29403	2.144876	9.85E-30	4.17E-27
c170182_g1	10.86737	2.716321	1.06E-37	9.41E-35
c170185_g1	-2.56632	1.495644	1.38E-05	0.000226831
c170189_g4	7.887759	-0.15513	2.32E-11	1.10E-09
c170193_g2	7.423509	-0.56202	1.54E-08	4.56E-07
c170193_g3	8.133764	0.06591	1.24E-12	7.24E-11
c170195_g4	-2.14599	0.56909	2.50E-06	4.85E-05
c170202_g1	9.159662	1.034602	1.14E-22	2.28E-20
c170211_g3	10.80229	2.65354	3.07E-47	7.57E-44
c170216_g1	6.772799	-1.08439	7.61E-07	1.63E-05
c170216_g2	9.078498	0.951897	3.49E-18	4.15E-16
c170230_g3	6.375583	-1.37927	1.95E-05	0.000307722
c170242_g2	8.482173	0.391739	4.54E-17	4.74E-15
c170357_g1	3.018383	1.321732	2.02E-10	8.39E-09
c170367_g1	-2.16298	5.102858	3.14E-22	5.92E-20
c170495_g1	3.092002	8.314244	2.01E-20	3.09E-18
c170576_g1	3.343919	5.440399	3.58E-34	2.34E-31
c170743_g1	6.594707	-1.22262	7.39E-06	0.000129303
c170752_g1	-2.38194	3.615858	6.81E-14	4.66E-12
c170836_g1	-2.51151	3.575129	3.99E-20	5.90E-18
c170859_g1	-2.45852	5.612867	1.59E-20	2.45E-18
c170895_g1	2.316934	1.10675	1.83E-05	0.000291735

c170955_g1	-7.55687	-0.25072	5.92E-06	0.000106157
c170956_g1	4.398696	1.767074	5.88E-14	4.06E-12
c170960_g1	3.915037	2.143775	3.50E-17	3.71E-15
c170980_g1	2.51228	5.66013	5.24E-15	4.19E-13
c171076_g1	4.704795	-0.9132	2.19E-05	0.000340747
c171149_g1	2.887308	4.131317	6.19E-19	7.83E-17
c171348_g1	2.85556	2.877444	4.11E-14	2.90E-12
c171499_g1	-2.33693	3.320022	2.70E-12	1.50E-10
c172271_g1	7.454819	-0.55565	1.10E-05	0.000184355
c172779_g1	7.631707	1.731265	1.69E-30	7.81E-28
c173039_g1	4.471956	-1.08436	1.35E-05	0.00022239
c175241_g2	2.453294	0.905997	1.01E-07	2.60E-06
c175296_g1	2.974663	2.316975	2.90E-13	1.84E-11
c175299_g1	2.393028	5.285224	1.58E-24	3.92E-22
c175319_g1	2.208005	7.801525	1.78E-29	7.50E-27
c175413_g1	3.526082	6.793201	8.40E-22	1.51E-19
c175414_g1	2.206763	4.362274	2.36E-08	6.77E-07
c175452_g1	7.356478	-0.60313	1.51E-08	4.48E-07
c175455_g1	2.010974	5.737761	6.62E-23	1.37E-20
c175468_g1	-2.32031	5.87072	2.14E-24	5.22E-22
c175576_g1	2.065261	4.663269	5.42E-12	2.85E-10
c175580_g1	2.859363	5.957217	4.92E-41	6.36E-38
c175582_g1	-2.0207	3.534446	7.48E-12	3.84E-10
c176080_g1	2.383868	1.352115	1.91E-08	5.56E-07
c176186_g1	4.164705	0.17875	1.69E-10	7.08E-09
c176399_g1	2.511418	4.689782	1.54E-24	3.84E-22
c176464_g1	4.935982	0.495967	3.87E-16	3.54E-14
c176526_g1	2.838685	2.730551	6.96E-20	9.81E-18
c176622_g1	2.017726	2.712571	1.07E-08	3.29E-07
c176748_g1	2.537792	2.815055	2.75E-17	2.94E-15
c176754_g1	-3.59558	-0.8043	1.45E-05	0.000236011
c176834_g1	2.144013	1.18593	2.34E-08	6.74E-07
c177109_g1	2.318021	0.185292	2.51E-05	0.000387599
c178100_g1	-2.42409	1.147157	1.17E-09	4.27E-08
c178542_g1	6.305478	-1.43244	3.53E-05	0.000522258
c17872_g1	2.528864	2.902472	1.35E-14	1.02E-12
c18002_g2	3.548217	0.835908	1.80E-11	8.77E-10
c180211_g1	3.531036	6.877819	1.08E-56	3.72E-53
c180412_g1	-2.21986	0.253978	8.98E-06	0.000153876
c180491_g1	-2.48111	5.694113	1.36E-21	2.39E-19
c180504_g1	2.407191	6.252237	4.97E-28	1.76E-25
c180612_g1	2.449759	5.9346	3.66E-25	9.75E-23
c180622_g1	-2.56345	2.492272	1.06E-05	0.000178696
c180626_g1	-2.29755	4.051875	9.42E-21	1.50E-18
c180741_g1	6.8301	-1.04034	5.21E-07	1.16E-05
c180820_g1	7.514961	-0.4758	2.16E-10	8.89E-09
c180830_g1	2.227264	4.193327	1.62E-16	1.57E-14
c180919_g1	4.424508	-0.44238	2.60E-06	5.03E-05
c180950_g1	2.282609	1.293714	3.99E-08	1.11E-06
c181012_g1	2.502851	4.999126	7.82E-26	2.22E-23
c181074_g1	8.021827	-0.05416	1.60E-07	3.93E-06
c181140_g1	4.911277	1.575456	3.55E-21	5.90E-19
c181204_g1	3.823719	3.086106	5.30E-24	1.25E-21
c181311_g1	5.637493	1.194461	7.32E-10	2.76E-08

c181328_g1	-5.23871	4.136299	7.06E-24	1.63E-21
c181701_g1	6.588864	-1.22415	4.43E-06	8.16E-05
c181837_g1	3.861796	-0.42021	2.48E-07	5.87E-06
c182311_g1	4.960968	2.49884	9.30E-14	6.29E-12
c182811_g1	6.782058	-1.08213	2.44E-06	4.74E-05
c183610_g1	2.637454	2.235733	7.23E-17	7.37E-15
c185596_g1	3.048424	0.762925	2.69E-06	5.17E-05
c185671_g1	2.254198	4.703856	3.94E-19	5.09E-17
c185750_g1	-2.42809	1.524984	7.14E-08	1.88E-06
c185794_g1	2.84125	4.542345	2.87E-16	2.67E-14
c185923_g1	4.392022	6.062938	1.97E-21	3.39E-19
c185995_g1	5.524745	6.190594	1.18E-07	2.99E-06
c185998_g1	2.600347	4.897992	6.58E-29	2.56E-26
c186006_g1	-2.06623	2.692373	1.24E-05	0.000205209
c186097_g1	-2.34079	2.05706	3.57E-12	1.94E-10
c186187_g1	-2.35315	2.696738	5.36E-12	2.83E-10
c186197_g1	2.519638	4.012844	2.43E-09	8.38E-08
c186391_g1	2.825586	1.97238	7.20E-13	4.38E-11
c186440_g1	-2.04662	2.089785	1.20E-08	3.64E-07
c186728_g1	6.384151	-1.37694	2.52E-05	0.000389518
c186746_g1	4.653743	2.698273	9.35E-14	6.32E-12
c186829_g1	6.832828	-1.03958	7.18E-07	1.56E-05
c187141_g1	4.815185	1.491289	4.16125E-21	6.85268E-19
c189679_g1	7.494548	-0.50091	6.2952E-09	2.03182E-07
c190703_g1	3.088213	9.437289	1.03E-20	1.62E-18
c190734_g1	-2.85869	1.219227	8.70E-12	4.43E-10
c190821_g1	2.013326	5.486825	6.19E-17	6.37E-15
c190829_g1	2.064532	7.514996	1.06E-23	2.39E-21
c190891_g1	-2.69202	0.826028	5.93E-09	1.92E-07
c190903_g1	2.488405	3.214284	5.47E-15	4.35E-13
c190906_g1	2.008206	7.787381	2.34E-14	1.72E-12
c190910_g1	3.81868	4.448153	3.89E-34	2.51E-31
c190941_g1	2.854107	4.579136	1.11E-17	1.24E-15
c191092_g1	2.004668	5.99279	5.55E-23	1.16E-20
c191102_g1	2.515178	2.401802	1.01E-11	5.07E-10
c191245_g1	-2.36761	6.350965	7.48E-20	1.05E-17
c191333_g1	2.561154	4.747196	1.16E-10	4.96E-09
c191395_g1	11.7684	3.61847	6.44E-65	3.70E-61
c191491_g1	2.813318	2.038416	1.44E-15	1.23E-13
c191647_g1	6.656496	-1.17507	3.98E-06	7.41E-05
c191800_g1	7.760766	-0.27558	6.57E-09	2.11E-07
c191940_g1	3.397224	0.520814	4.19E-10	1.65E-08
c192079_g1	3.788174	-0.16701	8.24E-09	2.57E-07
c192247_g1	3.706578	-1.00176	9.78E-06	0.000166512
c192506_g1	2.595414	2.942128	5.40E-20	7.73E-18
c192609_g1	6.393132	-1.37539	6.65E-05	0.000919483
c192683_g1	6.588865	-1.22415	4.44E-06	8.17E-05
c193102_g1	5.358634	0.397243	4.24E-13	2.66E-11
c193104_g1	2.39044	1.317581	1.67E-09	5.95E-08
c193965_g1	5.091086	-0.59814	4.12371E-08	1.13825E-06
c195758_g1	-2.28297	5.732098	2.51E-13	1.61E-11
c195769_g1	-3.32386	0.032592	7.56E-08	1.98E-06
c195797_g1	2.081319	5.919491	4.17E-10	1.64E-08
c195903_g1	-2.14981	5.201189	2.05E-21	3.51E-19

c195967_g1	6.530367	-1.27178	1.48E-05	0.000240602
c195994_g1	2.485463	5.231062	1.34E-12	7.73E-11
c196047_g1	4.170984	2.088822	9.33E-23	1.89E-20
c196088_g1	3.650912	-0.60281	5.22E-07	1.16E-05
c196180_g1	7.216018	-0.73014	1.67E-08	4.91E-07
c196208_g1	-2.18057	2.797642	3.58E-12	1.95E-10
c196251_g1	3.088777	6.073799	5.01E-29	1.96E-26
c196326_g1	2.075115	3.43151	1.01E-12	6.03E-11
c196416_g1	3.111753	6.464198	1.68E-40	2.07E-37
c196424_g1	-4.26432	-0.98722	1.69E-05	0.000270838
c196474_g1	2.063878	5.330746	4.04E-13	2.53E-11
c196504_g1	2.64204	4.425728	9.59E-27	2.99E-24
c196563_g1	2.642062	1.965562	1.76E-12	9.98E-11
c196726_g1	4.360886	-1.17685	6.70E-05	0.000925051
c196860_g1	6.648636	-1.17736	3.06E-06	5.83E-05
c196900_g1	3.471653	3.600429	5.55E-22	1.02E-19
c197065_g1	9.364838	1.236384	5.7967E-28	2.03906E-25
c197170_g1	3.680348	-0.2351	9.30E-08	2.40E-06
c197372_g1	2.177845	4.252552	9.51E-09	2.94E-07
c198496_g1	6.222518	-1.49009	5.09E-05	0.000724089
c198680_g1	4.196838	-0.60614	4.04E-06	7.50E-05
c20998_g1	2.224543	5.469933	2.75E-18	3.30E-16
c21093_g1	3.271676	-0.88137	6.23E-05	0.000866287
c21858_g1	6.656495	-1.17507	3.98E-06	7.41E-05
c22576_g1	2.04	4.571789	1.89E-09	6.67E-08
c22651_g1	2.163196	2.918206	1.66E-12	9.45E-11
c23797_g1	3.482648	3.906036	1.31E-20	2.03E-18
c24145_g1	7.227284	-0.72724	1.08E-07	2.75E-06
c24584_g1	2.377619	5.098934	2.01E-13	1.31E-11
c24640_g1	4.752345	1.928473	2.71E-26	8.00E-24
c24823_g1	-2.42921	5.351514	8.05E-25	2.06E-22
c24923_g1	2.02806	7.442925	1.28E-08	3.87E-07
c24969_g1	-2.59386	1.625966	1.97E-09	6.91E-08
c25531_g1	2.089639	1.90742	6.46E-10	2.46E-08
c26096_g1	4.743546	2.171362	7.80E-28	2.71E-25
c26141_g1	6.591545	-1.22339	5.27E-06	9.56E-05
c26182_g1	2.045268	3.778316	1.61E-16	1.56E-14
c26450_g1	4.060181	1.723848	1.84E-22	3.60E-20
c26597_g1	3.317687	7.808488	5.51E-34	3.35E-31
c26872_g1	3.006665	1.209911	1.25E-13	8.32E-12
c27310_g1	9.528599	3.614663	1.31251E-50	3.57202E-47
c27690_g1	3.017964	0.831854	6.82E-10	2.60E-08
c27848_g1	7.168065	-0.76727	1.57912E-08	4.66817E-07
c27898_g1	6.839643	-1.03808	1.97515E-06	3.91078E-05
c28300_g1	-2.21788	0.709231	3.02E-05	0.000454726
c28300_g2	-2.17556	1.925864	7.32E-09	2.32E-07
c28503_g1	-2.05211	1.286647	8.43E-08	2.19E-06
c29329_g1	2.452935	2.054376	3.73E-12	2.03E-10
c30023_g1	4.480578	6.402288	2.48E-29	1.01E-26
c31778_g1	3.992174	2.028048	1.04E-19	1.44E-17
c34762_g1	5.644259	6.744721	1.89E-40	2.27E-37
c36165_g1	6.990019	-0.91545	2.04E-07	4.92E-06
c37965_g1	4.362606	4.800126	7.82E-23	1.61E-20
c38525_g1	2.378491	1.765851	1.95E-11	9.39E-10

c39259_g1	2.451836	0.908115	3.68E-08	1.03E-06
c39320_g1	8.942106	3.027448	9.94E-39	9.88E-36
c39816_g1	3.28655	0.132031	4.23E-09	1.41E-07
c39931_g1	2.840148	7.04231	9.80E-26	2.72E-23
c40378_g1	2.931587	3.971309	4.17E-20	6.11E-18
c40571_g1	-2.41325	2.885214	3.36E-14	2.40E-12
c41405_g1	2.287374	2.466167	2.33E-09	8.08E-08
c41432_g1	3.114735	3.339041	8.47E-29	3.22E-26
c41432_g2	3.271633	1.516347	4.55E-16	4.14E-14
c41587_g1	8.298598	0.219602	1.18E-14	9.05E-13
c41664_g1	6.302519	-1.43322	2.91E-05	0.000441924
c41753_g1	6.388167	-1.37617	3.87E-05	0.000565886
c42358_g1	2.746684	6.802795	2.46E-22	4.72E-20
c42466_g1	2.005857	2.277586	3.33E-07	7.70E-06
c42787_g1	2.193726	3.472664	7.17E-14	4.88E-12
c42811_g1	11.03074	2.874474	3.1784E-22	5.97644E-20
c42912_g1	6.523751	-1.27332	7.53819E-06	0.000131459
c43062_g1	2.202271	3.962274	1.52E-17	1.68E-15
c43184_g1	2.236477	1.395739	7.95E-10	2.99E-08
c4366_g1	2.669678	0.534209	6.71E-06	0.00011889
c45337_g1	8.887079	0.770254	4.655E-18	5.44582E-16
c46749_g1	6.388164	-1.37617	3.87E-05	0.000565886
c46769_g1	9.474381	1.342967	1.89566E-29	7.90507E-27
c46919_g1	3.545221	4.327737	1.60E-34	1.09E-31
c47305_g1	2.710367	5.386581	1.69E-12	9.62E-11
c47372_g1	5.616069	3.933316	7.93E-29	3.04E-26
c48500_g1	6.843765	-1.03733	3.7094E-06	6.96981E-05
c48518_g2	3.496675	1.274316	2.34E-11	1.11E-09
c48539_g1	3.154312	0.920676	6.30E-12	3.27E-10
c48567_g1	2.077458	4.119897	2.80E-09	9.59E-08
c48817_g1	-2.82874	1.685279	7.28E-05	0.00099632
c48834_g1	2.727998	4.997729	8.81E-24	2.00E-21
c48886_g1	-2.13156	6.334939	2.21E-26	6.56E-24
c48978_g1	2.942958	7.243605	1.26E-33	7.25E-31
c49140_g1	2.011939	5.682035	1.36E-17	1.51E-15
c49705_g1	2.119193	1.124611	5.90E-07	1.30E-05
c49768_g1	3.194489	1.591453	2.49E-16	2.33E-14
c49768_g2	3.372852	-0.19399	9.86E-06	0.000167118
c49835_g1	2.210427	7.666407	5.57E-20	7.96E-18
c49973_g1	8.665643	0.562934	4.04E-18	4.76E-16
c50161_g1	3.83973	-0.44791	3.99E-07	9.10E-06
c51126_g1	3.213393	4.96005	1.20E-16	1.18E-14
c51126_g2	2.973688	1.124751	4.35E-11	1.98E-09
c51344_g1	-2.62936	-0.30273	5.82E-05	0.000811668
c53049_g1	6.586361	-1.22492	4.41381E-06	8.15578E-05
c54473_g1	6.380977	-1.37772	1.92167E-05	0.000303523
c56891_g1	2.783586	0.424265	2.77E-08	7.86E-07
c57646_g1	2.74646	-0.13908	2.39E-05	0.000370241
c57729_g1	2.898891	5.765074	5.68E-19	7.20E-17
c57824_g1	3.466322	6.539205	7.88E-33	4.38E-30
c5829_g1	6.222517	-1.49009	5.09E-05	0.000724089
c58347_g1	6.598505	-1.22186	7.86E-06	0.00013589
c5873_g1	6.948567	-0.9537	1.35E-06	2.77E-05
c6033_g1	-2.7554	2.322823	1.30E-10	5.53E-09

c62934_g1	5.78644	-0.00207	1.56E-12	8.94E-11
c6298_g1	-9.31631	1.332878	2.71E-07	6.36E-06
c63206_g2	3.379791	0.629788	1.15E-06	2.39E-05
c63439_g1	4.106573	0.83276	2.15E-15	1.80E-13
c63509_g1	-2.10852	3.225149	4.18E-07	9.49E-06
c636_g1	4.292471	1.560637	8.29E-10	3.12E-08
c64475_g1	-7.37122	-0.41565	4.15E-06	7.70E-05
c64492_g1	5.533159	0.566662	5.24E-08	1.42E-06
c64947_g1	2.74934	5.536006	1.68E-10	7.03E-09
c65107_g1	3.421242	-0.46099	1.81E-05	0.000289071
c65279_g1	2.408811	5.832498	1.87E-23	4.09E-21
c66040_g1	9.477135	1.338111	1.1655E-20	1.82076E-18
c66164_g1	2.396949	0.95248	8.76E-08	2.27E-06
c66164_g2	3.000824	0.961814	8.06E-11	3.52E-09
c66386_g1	2.934103	4.556902	4.97E-21	8.13E-19
c66412_g1	2.12659	4.233728	2.80E-09	9.59E-08
c66745_g1	7.770131	-0.25577	1.64E-11	8.03E-10
c67372_g1	6.895077	-0.9953	1.60307E-06	3.24273E-05
c67748_g1	7.527484	-0.47162	2.88E-09	9.82E-08
c68021_g1	2.169744	8.296273	5.43E-08	1.47E-06
c68169_g1	3.688645	1.955175	9.63E-22	1.72E-19
c68182_g1	2.485161	5.032204	1.10E-27	3.71E-25
c68226_g1	3.144532	3.174073	8.96E-23	1.82E-20
c68709_g1	6.518552	-1.27486	7.56E-06	0.000131529
c68981_g1	6.216872	-1.49166	5.33E-05	0.000750756
c69763_g1	2.873823	-0.21826	3.25E-06	6.16E-05
c70690_g1	2.382522	4.271912	2.22E-07	5.29E-06
c7171_g2	2.624627	5.952781	5.65E-32	2.81E-29
c72103_g1	-2.86405	0.06226	2.10E-06	4.13E-05
c76029_g1	4.210739	2.767818	2.29E-24	5.55E-22
c77884_g1	-2.53214	0.674189	5.48E-06	9.88E-05
c80179_g1	3.243419	4.510083	1.12E-08	3.40E-07
c81124_g2	2.687277	1.574144	1.98E-10	8.23E-09
c81244_g1	8.851936	0.773442	4.47E-07	1.01E-05
c86193_g1	3.866742	1.58317	3.38E-11	1.56E-09
c86591_g1	-2.42927	0.876037	2.54E-06	4.91E-05
c88775_g1	7.207966	-0.73303	1.09E-08	3.33E-07
c89444_g1	7.684431	-0.33276	1.25431E-10	5.37054E-09
c89573_g1	-4.13699	-1.07654	4.61E-05	0.00066512
c91879_g1	2.590029	2.845537	4.67E-19	5.99E-17
c97782_g2	-2.22664	4.449382	9.67E-16	8.43E-14
c9993_g1	2.898741	7.597363	1.87E-39	2.15E-36

Supplementary Tables S2

The GO, KEGG and KOG annotation for DEUs. For reducing the list length, we only display the best hits of GO and KEGG. The abbreviations of KOG corresponding subcategories were listed follow:

A	RNA processing and modification
B	Chromatin structure and dynamics
C	Energy production and conversion
D	Cell cycle control, cell division, chromosome partitioning
E	Amino acid transport and metabolism
F	Nucleotide transport and metabolism
G	Carbohydrate transport and metabolism
H	Coenzyme transport and metabolism
I	Lipid transport and metabolism
J	Translation, ribosomal structure and biogenesis
K	Transcription
L	Replication, recombination and repair
M	Cell wall/membrane/envelope biogenesis
N	Cell motility
O	Posttranslational modification, protein turnover, chaperones
P	Inorganic ion transport and metabolism
Q	Secondary metabolites biosynthesis, transport and catabolism
R	General function prediction only
S	Function unknown
T	Signal transduction mechanisms
U	Intracellular trafficking, secretion, and vesicular transport
V	Defense mechanisms
W	Extracellular structures
X	--
Y	Nuclear structure
Z	Cytoskeleton

geneid	GO	KEGG	KOG	geneid	GO	KEGG	KOG
c163615_g1	GO:0006811	K02127	C	c162590_g2	GO:0006468	K00907	T
c167114_g1	GO:0008286	K02728	O	c148599_g1	GO:0016020	K00517	QI
c167717_g1	GO:0007275	K02355	J	c123554_g2	GO:0005886	-	R
c129764_g1	-	-	S	c165963_g3	GO:0006996	-	-
c169038_g1	GO:0007595	K00504	O	c166272_g1	GO:0005789	K01062	YU
c151100_g1	GO:0008021	K04557	DTZ	c161951_g1	GO:0005654	-	-
c160061_g1	GO:0003700	-	KT	c154015_g1	GO:0034315	K07977	U
c167203_g1	GO:0008299	K10661	A	c169282_g1	GO:0071287	K09490	O
c136372_g2	GO:0031012	K01342	OR	c148189_g1	GO:0046677	K02929	J
c66164_g1	GO:0003700	-	KT	c148158_g1	GO:0005488	K06945	U
c161372_g1	GO:0044444	K09542	-	c168799_g1	GO:0050789	-	OU
c167298_g1	GO:0016020	-	S	c167404_g1	GO:0022627	K02998	J
c169202_g7	GO:0008234	-	DO	c170078_g1	GO:0031072	K04043	O
c13099_g1	-	K01244	X	c165890_g1	GO:0006614	K02883	J
c54473_g1	GO:0030335	K07974	U	c176526_g1	GO:0044464	-	M
c167583_g1	GO:0010033	K01920	Q	c167957_g1	-	-	X
c126644_g1	GO:0005739	-	S	c169130_g1	GO:0031012	K09487	O
c161932_g1	GO:0050779	-	R	c168045_g1	GO:0048731	K04706	K
c167961_g2	GO:0005689	-	A	c170121_g9	GO:0044248	K00828	R
c165792_g1	GO:0042593	K00688	G	c170256_g1	GO:0031399	K08900	O
c159345_g1	GO:0070991	K00249	EI	c175455_g1	GO:0008152	K00307	QG
c167193_g2	GO:0006644	K08288	T	c169089_g3	GO:0042826	-	X
c169645_g3	GO:0005739	K03231	J	c166902_g1	GO:0005634	K01803	G
c168947_g1	GO:0030687	K03264	J	c161622_g1	GO:0005886	-	S
c168776_g1	GO:0019843	K02932	R	c169081_g1	GO:0005739	K09595	S
c167974_g1	GO:0031985	-	R	c169972_g2	GO:0031647	K01072	O
c166921_g2	GO:0042254	K03254	J	c156860_g2	GO:0005739	-	X
c167358_g1	GO:0015630	K09542	O	c165601_g1	GO:0046352	K06519	G
c163494_g1	GO:0005739	K01069	R	c168360_g1	GO:0044237	K00799	O
c123021_g1	GO:0090406	K01051	M	c169573_g18	GO:0005524	-	-
c195994_g1	GO:0010183	K09422	K	c159433_g1	GO:0005634	-	U
c156899_g1	-	-	R	c164516_g1	GO:0050896	-	X
c167839_g1	GO:0016020	-	-	c146068_g1	-	-	X
c163175_g5	GO:0005637	K06013	R	c176080_g1	GO:0005886	K02183	-
c169364_g1	GO:0003779	K05700	W	c156895_g1	GO:1903543	-	R
c168822_g1	GO:0043167	K08630	W	c166717_g1	GO:0008152	K00661	E
c165435_g1	GO:0008134	-	-	c118814_g1	GO:0003700	-	KT
c165678_g1	-	K03469	L	c170242_g2	GO:0047776	K01637	C
c161704_g1	GO:1990234	-	K	c175822_g1	GO:0042359	-	-
c166716_g3	GO:0016020	K08789	TR	c88775_g1	GO:0070062	-	S
c169573_g5	GO:0005200	-	-	c165719_g2	GO:0008152	K03419	S
c5873_g1	GO:0006426	K01880	J	c159624_g1	GO:0043537	-	-
c167399_g1	GO:0040035	-	-	c121922_g1	GO:0055085	K03454	C
c167826_g1	GO:0046318	K07198	T	c165937_g1	GO:0005488	K07521	T
c155178_g2	GO:0005634	K00485	Q	c175296_g1	GO:0005515	-	W
c186829_g1	GO:0006426	K01880	J	c161881_g1	GO:0018279	K00820	M
c159240_g3	GO:0005886	K00696	M	c167177_g1	GO:0005886	K05607	I
c165109_g1	GO:0022627	K02960	J	c169231_g2	GO:0030529	-	-
c148850_g1	GO:0031124	-	A	c165389_g2	-	-	X
c156002_g1	GO:0097060	K01049	R	c166697_g1	GO:0044444	K01363	O
c168629_g1	GO:0005179	K05236	A	c167307_g2	GO:0007623	K00207	F
c166522_g1	GO:0006986	K09511	O	c166907_g1	GO:0001101	K03781	P
c162587_g1	-	-	S	c68226_g1	GO:0048046	-	RW

c168492_g1	GO:0036374	K00681	E	c161003_g1	GO:0005515	-	X
c132215_g1	GO:0048255	K03258	A	c42648_g1	GO:0044444	K01166	-
c162806_g1	GO:0044237	K03965	C	c158510_g1	GO:0003779	K05390	-
c160887_g1	GO:0005488	K03283	O	c190821_g1	GO:0048046	K05349	GRV
c163226_g1	GO:0003729	K02163	A	c169806_g1	GO:0070095	K00850	G
c166962_g1	GO:0005783	-	U	c47372_g1	GO:0016049	K09286	K
c167891_g1	GO:0044421	K08752	I	c170195_g4	GO:0003700	-	KT
c169746_g1	GO:0007275	-	W	c170030_g2	GO:0010604	K02156	J
c159515_g1	GO:0043197	-	-	c165911_g1	GO:0045773	K07976	U
c166086_g1	GO:0015171	-	E	c166728_g1	GO:0035987	K05636	O
c156637_g1	GO:0030214	K06268	T	c170093_g1	GO:0016328	K10380	M
c162191_g2	GO:0005886	K00924	T	c167428_g1	GO:0005978	K00693	G
c169928_g1	GO:0006096	K01834	G	c169421_g1	GO:0009113	K00288	H
c166868_g4	GO:0034475	K01529	A	c167649_g6	GO:0016165	K00454	I
c169469_g1	GO:0009737	K07	G	c166343_g1	-	K02551	R
c166724_g1	-	-	X	c167152_g1	GO:0097009	K01946	I
c169394_g1	GO:0006355	K06106	T	c167615_g1	GO:0005634	K05915	S
c162255_g1	GO:0007608	K02199	R	c165922_g1	GO:0006614	K02912	J
c132278_g1	GO:0008324	K03450	E	c166746_g1	GO:0048568	K09355	K
c125630_g1	GO:0016328	K06573	P	c185995_g1	GO:0044533	K01209	-
c168321_g1	GO:0000387	-	K	c168987_g1	GO:0019438	K03295	X
c160075_g1	GO:0022627	K02974	J	c151042_g1	GO:1904294	-	R
c157013_g4	GO:0098542	K01723	I	c167115_g1	GO:0044767	-	-
c161075_g2	GO:0019752	K01957	JIT	c164460_g2	GO:0070062	K01870	J
c136323_g1	GO:0005886	K09873	G	c166529_g3	GO:0008286	-	-
c158135_g1	GO:0070125	K02935	J	c134638_g1	GO:0003824	-	-
c170081_g3	GO:0016568	-	S	c111101_g1	GO:0008276	-	-
c1785_g1	GO:0044421	K07761	OW	c165222_g1	GO:0004300	-	-
c169433_g1	GO:0016020	-	-	c150264_g5	GO:0016787	-	O
c169726_g1	-	-	W	c165816_g1	GO:0005524	-	-
c192079_g1	GO:0050896	K01199	MVG	c165747_g1	GO:0005634	K01090	R
c166835_g2	GO:0043266	K00844	G	c167582_g1	GO:0006626	K01412	C
c169958_g5	GO:0015630	K10388	Z	c169519_g1	GO:0000165	K02739	O
c169740_g1	GO:0016607	K08826	T	c120737_g1	GO:0009833	K00694	MG
c153802_g1	GO:0008171	K05279	R	c169415_g2	GO:0003008	K01187	G
c132307_g1	GO:0051082	K00754	G	c150564_g1	GO:0090602	-	S
c168759_g1	GO:0098805	K04645	-	c167633_g1	GO:0005739	K01342	OR
c160451_g1	GO:0003700	-	KT	c168606_g1	GO:0035072	K01529	A
c159031_g1	GO:0003700	K09422	K	c169576_g1	GO:0043537	K09272	R
c168818_g28	GO:0044444	-	T	c169638_g1	-	-	TZ
c138860_g1	GO:0005634	K03898	-	c169475_g1	GO:0005524	-	-
c157116_g1	GO:0005634	-	G	c159587_g5	GO:0005886	-	E
c76029_g1	GO:0048046	K00368	Q	c164262_g2	GO:0016020	-	X
c144077_g2	GO:0015770	K08139	G	c160553_g1	GO:0086004	K08583	T
c166702_g1	GO:0005634	-	S	c197170_g1	GO:0003700	K09286	K
c168203_g2	GO:0019005	-	-	c168813_g1	GO:0042030	K09553	O
c10276_g1	GO:0003700	K09522	O	c124094_g1	GO:0042254	K02941	J
c170104_g1	GO:0005739	K01648	C	c147987_g2	GO:0005634	-	S
c166837_g1	GO:0016787	K00140	R	c150348_g3	GO:0016787	-	-
c169817_g3	GO:0006355	K03231	J	c168599_g1	GO:0019538	K01516	O
c118265_g1	GO:0019538	K01285	OR	c165248_g1	GO:0003729	K02937	J
c155592_g1	GO:0042593	K07297	RT	c163018_g2	GO:0003779	K10456	TR
c166302_g1	GO:0008152	-	OT	c163271_g6	GO:0001101	K00924	R
c161191_g3	GO:0010091	K09422	K	c114696_g1	GO:0001701	-	-

c161863_g2	GO:1900445	K09448	K	c41587_g1	GO:0005739	K09580	O
c148469_g2	GO:0097320	K00680	IT	c169218_g1	GO:0005515	K02358	J
c168349_g1	GO:0007021	K09493	O	c168052_g3	GO:0005085	K08810	R
c163115_g2	GO:0005524	K01874	J	c166524_g5	GO:0008013	K06805	S
c153597_g1	GO:0055114	K03676	O	c168144_g1	GO:0016020	K00383	C
c169041_g18	GO:0005886	-	U	c43818_g1	GO:0080046	K08236	GC
c169918_g4	GO:0005905	K07191	R	c167973_g1	GO:0004722	K01090	T
c168512_g1	GO:0048041	K06106	Z	c169080_g1	GO:0044767	K06691	R
c161267_g1	-	-	X	c168951_g1	GO:0042169	K00567	S
c161832_g2	GO:0023021	K01117	I	c166928_g5	GO:0006325	-	-
c170090_g1	GO:0048468	K09027	-	c168105_g1	GO:0008233	K01412	O
c167580_g1	GO:0065002	K08509	U	c168742_g1	GO:0009887	K06237	W
c163131_g1	GO:0022627	-	-	c168041_g1	-	-	X
c164435_g1	-	K09935	-	c169082_g2	GO:0005488	K04733	T
c169871_g2	GO:0044444	K06813	W	c167311_g1	GO:0016568	K05610	O
c190703_g1	GO:0048046	-	X	c197065_g1	GO:0022627	K02993	J
c168504_g1	GO:0016020	-	-	c170157_g2	GO:0034260	K07817	T
c163931_g1	GO:0061630	K10634	O	c25409_g1	GO:0044444	K09587	QI
c165036_g2	-	-	R	c161770_g1	GO:0032940	K07977	UZ
c170182_g1	GO:0005634	K03234	J	c169289_g1	GO:0048731	K08568	O
c105379_g1	GO:0030433	K09481	O	c170077_g1	GO:0005488	K00505	E
c168375_g1	GO:0009617	K10092	-	c169460_g5	GO:0008021	K05639	R
c160723_g2	GO:0003700	K09286	K	c161547_g1	-	-	X
c156792_g1	GO:0045333	K02266	-	c166422_g2	GO:0009793	-	S
c126175_g1	GO:1901700	-	-	c169568_g2	GO:0030182	K04372	T
c169921_g1	GO:0006644	K01294	R	c170093_g2	GO:0030315	K10380	R
c169449_g2	GO:0030331	K01529	K	c168605_g5	GO:0009887	K00924	T
c169780_g1	-	K01497	X	c164594_g1	GO:0071458	K07564	A
c165475_g1	GO:0022627	K02951	J	c161868_g1	GO:0031124	K01173	A
c165547_g1	GO:0008152	K05360	S	c84912_g2	GO:0009408	-	X
c168652_g1	GO:0005789	K01204	G	c169002_g1	GO:0003700	-	-
c170027_g12	GO:0005886	K00924	R	c168651_g2	GO:0008593	K10601	O
c169716_g1	GO:0005886	-	-	c166135_g1	GO:0005783	K00457	E
c167745_g2	GO:0012506	K01537	P	c126086_g1	GO:0098542	K00924	T
c168863_g3	GO:0048598	K03909	O	c166693_g1	-	-	X
c170025_g1	GO:0071398	K00128	E	c169737_g1	GO:0016020	K04523	OR
c169286_g1	-	K09	O	c161844_g1	GO:0016568	-	BK
c158848_g4	GO:0048437	K00924	T	c165391_g1	GO:0005739	K01529	-
c123818_g1	GO:0016568	-	K	c167745_g1	GO:0012506	K01537	P
c12024_g1	GO:0044249	-	RQ	c169163_g1	GO:0009507	K09580	O
c169041_g30	-	-	S	c164303_g1	-	K03962	C
c169075_g1	GO:0033198	K04077	O	c154939_g4	GO:0035556	-	-
c169817_g2	GO:0006355	K03231	J	c167770_g2	GO:0030315	K05850	P
c159205_g1	GO:0008152	K07055	S	c114366_g1	-	-	X
c169487_g1	GO:0018279	K08056	O	c168178_g1	GO:0009611	-	S
c167899_g1	GO:0070991	K00249	R	c123324_g1	GO:0042254	K02922	J
c167766_g2	GO:0007269	-	-	c169870_g2	GO:0034199	K04739	T
c165856_g1	GO:0042589	K07889	T	c165492_g1	GO:0044249	K00065	Q
c165633_g1	GO:0007275	-	-	c169595_g1	GO:0040035	K09498	O
c169923_g1	GO:0008152	-	-	c169616_g1	GO:0070887	K07611	Z
c169991_g1	GO:0048856	K02156	J	c168156_g1	GO:0016020	K01509	A
c169273_g1	GO:0005634	K01373	O	c163592_g9	GO:0005634	-	KT
c166894_g1	GO:0048856	K01931	O	c118034_g1	GO:0006289	-	-
c166405_g1	GO:0010124	K00022	I	c68169_g1	GO:0005618	-	T

c196088_g1	GO:0009835	K01184	R	c169834_g1	GO:0071944	K00292	S
c164245_g1	GO:0005739	-	R	c196251_g1	GO:0010183	K09422	K
c122210_g1	GO:0007623	-	-	c150161_g1	GO:0048568	K05409	U
c166736_g2	-	-	S	c156430_g3	GO:0005829	K10712	S
c151432_g2	GO:0009901	K01184	R	c134084_g1	GO:0031012	K09377	TZ
c176748_g1	GO:0005576	-	R	c167180_g1	GO:0006397	-	A
c168528_g1	GO:0005886	K00517	Q	c141318_g1	GO:0005886	-	-
c166736_g1	-	-	S	c167842_g1	GO:0039529	-	U
c169756_g2	GO:0045167	K08798	TR	c165425_g1	GO:0048856	K02152	-
c163755_g7	GO:1902589	-	U	c161317_g2	GO:0043515	-	R
c49835_g1	-	-	S	c169179_g2	GO:0009306	K08875	R
c163372_g1	GO:0005739	K01102	T	c165764_g1	GO:0003729	K03102	A
c114351_g2	GO:0005773	K00505	-	c167514_g3	GO:0050794	-	-
c169183_g1	GO:0040029	-	A	c168478_g1	GO:0070991	K00257	R
c145742_g1	GO:0048443	K09587	QI	c166541_g2	GO:0030033	K08282	T
c162086_g1	GO:0044444	-	-	c165693_g1	GO:0005102	K07295	K
c161058_g1	GO:0031124	-	A	c161309_g1	GO:0008286	K03033	O
c154754_g1	GO:0022627	K02980	J	c191047_g1	GO:0090406	K01051	M
c7171_g2	GO:0009860	K00924	T	c165268_g1	GO:0031012	K04565	P
c165044_g1	GO:0008988	K00599	OK	c169385_g1	GO:0019538	K01628	TZ
c168808_g1	GO:0035038	-	T	c165777_g1	GO:0005085	K07582	J
c180504_g1	GO:0009535	K01807	G	c167775_g1	GO:0004721	K01112	R
c166625_g1	GO:0071539	K08957	T	geneid	-	ko	-
c165540_g1	GO:0030448	K08272	S	c165742_g2	GO:0048046	-	T
c160846_g1	-	-	X	c168421_g6	GO:0005886	-	T
c186006_g1	GO:0048046	K00430	R	c168579_g1	GO:0046135	K00757	F
c153321_g1	GO:0055085	K02180	D	c166935_g1	GO:0030155	K01104	T
c168735_g1	GO:0005886	-	-	c169611_g1	GO:1903382	K09486	O
c164578_g1	GO:0007519	K09377	TZ	c168875_g2	GO:0001755	K01124	U
c168372_g3	GO:0005829	K10712	S	c68182_g2	GO:0019752	K01747	Q
c165488_g1	GO:0005634	K02908	J	c48539_g1	GO:0005886	K00924	T
c168520_g1	GO:0007595	K00504	O	c168489_g2	GO:0090280	K01537	P
c159340_g1	GO:0070201	K07525	T	c162541_g1	GO:0001764	K01062	R
c170095_g1	GO:0000212	K07375	Z	c161144_g1	GO:0051926	K04534	T
c165743_g1	GO:0061133	K02735	O	c169691_g1	GO:0004349	K00147	E
c180626_g1	GO:0044444	K08237	GC	c113614_g1	GO:0030033	K07974	R
c157257_g1	GO:0016020	K02966	J	c139508_g1	GO:0086004	K01539	P
c135909_g1	-	K03938	-	c170015_g1	GO:0086004	K01539	P
c186187_g1	GO:0006355	-	TK	c159600_g1	GO:0006813	K03549	P
c169947_g1	GO:0005212	K01689	G	c195903_g1	GO:0005634	K09414	K
c167166_g4	GO:0010091	-	-	c167472_g1	GO:0005737	-	-
c150264_g3	-	-	O	c166544_g1	GO:0061732	K00161	C
c121181_g1	GO:0005783	K06238	O	c162954_g2	GO:0005634	-	R
c169499_g1	GO:0009408	K01417	O	c171149_g1	GO:0044464	-	V
c168062_g1	GO:0040029	K05302	-	c165298_g1	GO:0005739	K08341	Z
c152001_g1	GO:0044444	-	-	c165585_g1	GO:0022627	K02958	J
c169419_g1	GO:0044446	K02988	J	c128577_g1	GO:0003700	K09286	-
c157668_g1	GO:0003700	-	RK	c157533_g1	GO:0016020	-	-
c169144_g1	GO:0070062	-	-	c167416_g1	GO:0005488	K00866	I
c168318_g1	GO:0032501	K10758	D	c169272_g1	GO:0004054	K00934	C
c161467_g1	GO:1901137	K10157	O	c192506_g1	GO:0031225	K01184	R
c168211_g1	GO:0034626	K10203	I	c144226_g1	GO:0016020	-	F
c166610_g1	GO:0005739	K03768	O	c161112_g1	GO:0034599	K06174	A
c168455_g1	GO:0005634	K07197	K	c191092_g1	GO:0080187	K01376	O

c169918_g2	GO:0008152	-	-	c163371_g4	-	-	R
c166745_g1	GO:0044297	K07972	T	c170051_g1	GO:0005634	-	JA
Query_id	-	-	Clust	c168534_g1	GO:0003729	-	A
c169670_g1	GO:0035617	K10407	Z	c169783_g1	-	K10592	-
c169946_g1	GO:0008286	-	-	c169131_g1	GO:0008152	-	X
c169336_g1	GO:0048568	K00162	-	c168472_g1	-	-	X
c168868_g1	GO:0060548	K04501	TK	c161970_g1	GO:0071398	K00940	F
c168331_g2	GO:0005886	K01529	K	c118055_g1	-	-	R
c168159_g1	GO:0048856	K02183	T	c168550_g1	GO:1902589	-	K
c167481_g1	GO:0044444	K01757	R	c168092_g1	GO:0005515	K09292	-
c169315_g1	GO:0055085	K05863	F	c167518_g2	-	-	V
c168539_g1	-	-	S	c118780_g2	GO:0009860	K03305	E
c167760_g1	GO:0048468	-	-	c168718_g1	GO:0001652	K04795	A
c186097_g1	GO:0003700	K09060	-	c175468_g1	GO:0005886	-	P
c20998_g1	GO:0005634	-	S	c118140_g1	GO:0016020	-	M
c167770_g3	GO:0030315	K05850	P	c167135_g1	GO:0042542	-	P
c121360_g1	GO:0006325	-	B	c118439_g1	GO:0003700	-	S
c169375_g1	GO:0045490	K01728	-	c157128_g1	GO:0008233	K01423	U
c149487_g1	GO:0004462	K01759	G	c57824_g1	GO:0005634	-	R
c163263_g1	GO:0005739	K01580	E	c170576_g1	GO:0048046	K01569	V
c149641_g1	GO:0008152	K01051	M	c152423_g1	GO:0005634	-	-
c46919_g1	GO:0050793	-	K	c158798_g3	GO:0048441	K09587	QI
c159975_g3	-	-	S	c167600_g1	GO:0016881	-	-
c169948_g1	GO:0003774	K10352	Z	c134231_g1	GO:0071013	K01802	O
c159751_g2	GO:0030335	K06704	W	c166404_g1	GO:0006468	K08827	R
c137475_g2	GO:0031012	K01342	OR	c166599_g1	GO:0008286	K02725	O
c163778_g1	GO:0098771	-	P	c169579_g1	GO:0002199	K09497	O
c158296_g1	GO:0016020	K01529	A	c156390_g1	GO:0005634	K02901	J
c136046_g1	GO:0071555	K09833	X	c166011_g2	GO:0005938	K02218	T
c165983_g1	GO:0010770	K04440	R	c169405_g1	GO:0007623	K02177	A
c166979_g1	GO:0004029	K00294	EG	c170137_g1	GO:0008340	K04079	O
c151233_g1	GO:0005739	K05309	R	c170568_g1	GO:0048046	K01569	V
c39259_g1	GO:0050779	-	R	c191395_g1	GO:0005739	K01386	O
c165322_g1	GO:0005739	K06158	R	c157698_g1	GO:1902589	K10420	-
c168314_g1	GO:0044271	K01528	UR	c169376_g1	GO:0070062	K09560	OT
c148354_g1	GO:0005654	-	AR	c124530_g1	GO:0006355	K01072	O
c191333_g1	-	-	X	c167788_g1	GO:0071267	K08963	G
c168502_g1	GO:0018279	-	U	c165312_g1	GO:0030479	K10382	Z
c169386_g2	GO:0016032	K06240	-	c167639_g1	GO:0032780	K05767	Z
c170146_g2	GO:0006631	K01959	I	c167308_g2	GO:0015421	K05656	Q
c162616_g1	GO:0090316	K08510	U	c165091_g2	GO:0007030	-	R
c165864_g1	GO:0005634	K08753	I	c131077_g1	GO:0003824	-	-
c167250_g1	-	-	X	c162656_g1	GO:0032940	-	U
c158655_g1	GO:0031012	K01349	O	c168946_g1	GO:0042254	K02885	J
c169068_g2	GO:0005882	K07611	DY	c164525_g1	GO:0019005	K03094	O
c166778_g1	GO:0043981	-	-	c126171_g1	GO:0005488	K02942	-
c156993_g1	GO:0016020	K03102	R	c162988_g1	GO:0006734	K00164	G
c165577_g1	GO:0005739	-	-	c168203_g1	GO:0019005	K03094	O
c149183_g3	GO:0003700	-	-	c169749_g2	GO:0009860	-	YU
c157274_g2	GO:0030331	K01529	A	c163311_g1	GO:0005634	K02943	-
c113541_g1	GO:0005886	K03265	J	c133258_g1	GO:0022627	-	-
c191178_g1	GO:0005634	-	-	c147691_g2	GO:0005634	K02488	K
c166473_g1	GO:0006614	K03076	UO	c167897_g1	GO:0005789	K00626	I
c168349_g2	GO:0007021	K09493	O	c113949_g1	GO:0005634	K01802	R

c164233_g1	GO:0042826	-	NI	c161767_g1	GO:0005768	-	-
c68182_g1	GO:0019752	K10775	Q	c153804_g1	GO:0005200	K05754	Z
c84395_g1	GO:0005507	K09841	Q	c166143_g1	-	-	U
c167925_g1	GO:1901363	K01893	J	c166329_g1	GO:0009910	-	R
c160766_g1	GO:0015036	K03671	O	c113156_g1	GO:0005764	K05728	T
c169084_g1	GO:0040011	K08568	R	c170836_g1	GO:0042826	-	S
c166034_g2	GO:0042255	K03253	J	c153665_g1	GO:0030433	K07151	O
c168663_g1	GO:0005789	K00626	I	c168639_g1	GO:2001046	K01376	T
c168125_g1	GO:0005634	K05862	P	c167975_g1	GO:0008286	K02991	J
c167156_g1	GO:0005794	-	TU	c168011_g1	GO:0001782	K09263	K
c166208_g3	GO:0008152	K00309	QG	c169202_g4	GO:0006952	K01768	-
c122767_g1	GO:0009793	K08233	Q	c165942_g1	-	K06239	-
c160333_g1	GO:0051726	-	A	c168575_g1	GO:0005886	K09584	O
c169516_g1	GO:1900246	-	J	c161397_g2	GO:0071495	K04591	T
c169604_g2	GO:0005634	K01255	R	c166510_g1	GO:0002699	K06831	W
c165705_g2	GO:0005634	-	U	c169668_g1	GO:0008453	-	-
c169923_g2	GO:0004553	K01187	G	c157411_g1	GO:0051276	-	-
c167022_g1	GO:0005739	-	S	c27266_g2	GO:0048046	K00430	R
c156341_g1	GO:1900025	K06867	R	c157801_g2	GO:0005739	-	-
c169317_g1	GO:0055085	K01530	P	c169381_g3	GO:0005638	K07611	DY
c48978_g1	GO:0005634	-	R	c168697_g1	GO:2000670	K10093	W
c167943_g1	GO:0004775	K01899	C	c168912_g1	GO:0016020	K03251	J
c169829_g3	GO:0044707	-	R	c157621_g1	GO:0044444	-	-
c168672_g1	GO:0030246	K01206	G	c163625_g1	GO:0005515	K06255	O
c160508_g1	GO:0050830	-	-	c161121_g1	GO:0042373	K00079	Q
c169430_g2	GO:0005504	-	-	c26872_g1	GO:0090602	-	S
c168333_g1	GO:0040035	K03454	C	c167690_g2	GO:0010091	K09422	K
c170163_g1	GO:0005886	K07374	Z	c170126_g1	GO:0048523	K06816	U
c160631_g1	GO:0005783	K01802	O	c154400_g1	GO:0005524	K01876	J
c159673_g1	GO:0016020	K08059	O	c157335_g2	GO:0016757	-	-
c167936_g1	GO:0007040	K09105	K	c164169_g1	GO:0005634	K03454	C
c146172_g4	GO:0016023	K08252	T	c168999_g12	GO:0009992	K00924	T
c169268_g1	GO:0030448	-	BD	c165222_g2	GO:0004300	K00022	I
c167770_g1	GO:0030315	K05850	P	c168496_g1	GO:0030331	K01529	A
c164567_g2	GO:0005634	-	R	c164903_g1	GO:0005634	K02732	O
c42101_g1	GO:0098542	K00924	T	c169749_g1	GO:0009860	-	YU
c127683_g2	GO:0016132	K00559	IR	c165185_g3	GO:0044707	-	-
c164952_g1	GO:0005634	-	-	c168734_g1	GO:0005581	-	W
c195954_g1	GO:0000272	K01183	R	c168742_g3	GO:0072577	K06237	W
c165785_g1	GO:0001410	K01529	KL	c169573_g9	GO:0040035	K05692	Z
c168178_g2	GO:0044424	-	I	c169384_g1	GO:2000767	K09276	J
c115018_g2	GO:0005576	K01238	-	c165687_g1	GO:0030518	K05633	O
c167785_g1	GO:0030529	K09540	UO	c169352_g1	GO:0016020	K09500	O
c166647_g1	GO:0005789	K01897	I	c169677_g1	GO:0005200	K07374	Z
c168694_g2	GO:0052689	-	-	c161824_g1	GO:0042345	K03386	O
c173039_g1	GO:0080167	-	-	c155293_g2	GO:0003700	-	K
c163227_g1	GO:0048523	K01298	S	c169672_g1	GO:0016462	K01515	-
c169625_g1	GO:0007165	-	-	c167246_g1	GO:0044822	-	R
c169306_g1	GO:0070062	K02925	J	c175413_g1	GO:0005794	-	X
c163993_g1	GO:0005739	-	-	c169240_g1	GO:0003729	-	A
c169571_g1	GO:0034626	K10203	I	c157328_g2	GO:0005488	K08076	O
c169694_g1	GO:0007269	K00823	E	c161119_g2	GO:0003700	-	-
c181701_g1	-	-	X	c166898_g1	GO:0006283	K10599	A
c27690_g1	GO:0005886	K01757	R	c123108_g1	GO:0009507	K03321	P

c159706_g1	GO:0031225	K01184	R	c169139_g3	GO:0009887	K06777	-
c150320_g3	GO:0005222	K05391	PT	c163393_g1	GO:0031124	K03037	R
c169501_g9	GO:0008324	-	ET	c168052_g2	GO:0003725	-	K
c153077_g1	GO:0006950	-	-	c165515_g1	GO:0031012	-	-
c158557_g1	GO:0006289	-	-	c30023_g1	-	-	S
c166893_g1	GO:0006879	K02155	C	c114884_g1	GO:0042254	K02882	J
c165193_g1	GO:0006614	K02900	J	c166524_g3	GO:0006928	K06803	S
c165795_g2	GO:0016197	-	U	c161274_g2	GO:0016020	K06071	T
c166737_g3	GO:0032501	K10610	L	c160968_g1	GO:0008286	K02730	O
c162485_g2	GO:0044432	K06096	-	c165182_g2	GO:0060116	K01104	R
c166523_g1	GO:0006281	-	YU	c167820_g2	GO:0016020	K04437	Z
c167622_g1	GO:0034975	-	S	c159366_g1	GO:0001944	K01072	Z
c46749_g1	GO:0008177	-	-	c167715_g1	GO:0044421	K08129	R
c169500_g1	GO:0030198	-	Z	c163873_g1	GO:0005524	K01881	J
c150550_g2	GO:0005634	K08332	R	c169918_g1	GO:0044425	-	-
c166688_g1	GO:0005634	K02866	J	c115091_g1	GO:0031225	-	H
c165985_g1	GO:0015031	-	R	c196047_g1	-	-	X
c168032_g1	GO:0042347	K04457	T	c186728_g1	GO:0001662	-	-
c169465_g1	GO:0016020	K08193	G	c156113_g1	GO:0045893	K02977	J
c166446_g1	GO:0003700	-	K	c164381_g1	GO:0019843	K02893	J
c169851_g1	GO:0044421	K03514	D	c176399_g1	GO:0004650	K01213	R
c166775_g2	GO:0006644	K07752	R	c158341_g1	GO:0006734	K00164	G
c170280_g1	GO:0005516	K01537	P	c167632_g1	GO:0030016	-	-
c169263_g3	GO:0006334	-	-	c167188_g1	GO:0008340	K06630	O
c10902_g1	GO:0006950	K00286	E	c162068_g1	GO:0015825	-	S
c169112_g1	GO:0005886	-	T	c144904_g2	GO:0005634	-	K
c160686_g2	GO:0031408	K06900	I	c170111_g1	GO:0005634	-	-
c151753_g2	GO:0009611	K09422	K	c168605_g1	GO:0005488	K00924	T
c124410_g1	GO:0006888	-	U	c167004_g1	GO:0048205	-	-
c169170_g2	-	-	L	c159390_g2	GO:0006952	K05387	PET
c157439_g1	GO:0034641	K01080	I	c183610_g1	GO:0044464	-	-
c165358_g1	GO:0048468	-	-	c168956_g2	GO:0006546	K00382	C
c168509_g1	GO:0046320	K07200	C	c41753_g1	GO:0005515	K07023	R
c162155_g1	GO:0005634	K05757	Z	c151430_g1	GO:0005212	K09542	O
c146875_g4	GO:0003700	K09286	K	c166232_g1	GO:0044444	K02156	D
c158510_g2	GO:0003779	K05390	PT	c166468_g1	GO:0032501	-	-
c166997_g3	GO:0008286	-	-	c165474_g1	GO:0016020	-	V
c187273_g1	GO:0044249	-	RQ	c164637_g1	GO:0042347	-	-
c166777_g1	GO:0044249	K00128	E	c169826_g1	GO:0005739	K08585	OT
c38525_g1	GO:0043231	-	X	c147061_g1	GO:0009911	K03869	O
c152842_g1	GO:0007275	-	U	c162980_g1	GO:0046856	K01112	R
c42912_g1	GO:0016020	K08582	-	c169993_g1	GO:0030529	-	R
c68709_g1	GO:0034260	K04439	T	c165679_g3	GO:0044444	K09113	K
c167251_g1	GO:0005634	K00016	C	c166679_g1	GO:0044444	-	S
c168911_g1	GO:0005794	-	T	c169538_g17	GO:0003700	K09286	K
c167780_g1	GO:0005783	K00100	I	c167967_g1	GO:0006083	K01895	I
c145441_g1	GO:0032024	-	-	c133407_g1	GO:0051707	K08241	QV
c166420_g1	GO:0000398	-	A	c166431_g2	GO:0048568	K06255	W
c169986_g9	GO:0080142	K02183	RT	c126490_g1	-	-	X
c168273_g1	GO:0005634	-	R	c170495_g1	GO:0004022	K00001	Q
c152488_g1	GO:0001701	K03238	J	c165801_g9	-	-	I
c64947_g1	GO:0048046	-	X	c160902_g1	GO:0005886	-	M
c161070_g1	GO:0044707	-	R	c167841_g1	GO:0022008	K00160	S
c168742_g4	GO:0005488	-	-	c168729_g3	GO:0031055	K01529	L

c175580_g1	GO:0009901	K01184	R	c169037_g2	GO:0080187	-	-
c161247_g2	GO:0030151	K00360	C	c164953_g1	GO:0016020	K01509	A
c168264_g1	GO:0006122	K00411	C	c164214_g1	GO:0004540	K03469	L
c156295_g1	GO:0005634	K02877	J	c160284_g4	GO:0048316	K06626	D
c167476_g1	GO:0005739	K01802	O	c169818_g1	GO:0006011	K00963	G
c153802_g4	GO:0005886	-	-	c11806_g1	GO:0016020	K00517	Q
c163328_g8	GO:0097502	K00694	MG	c168019_g5	GO:0009267	K01915	E
c169891_g1	GO:0044237	K00754	-	c71527_g1	GO:0005634	K05865	-
c169104_g1	GO:0042981	-	-	c164872_g1	GO:0003729	K02872	J
c182311_g1	GO:0097367	-	-	c142673_g1	GO:0016482	K10380	-
c169024_g1	GO:0035522	-	B	c170028_g1	GO:0040002	K01509	O
c169232_g1	-	-	W	c156069_g6	-	K06910	-
c168772_g1	GO:0004872	K05719	TW	c162381_g1	GO:0050794	-	X
c158110_g1	GO:0040029	K00091	V	c21858_g1	GO:0022008	-	-
c26096_g1	GO:0003700	K09286	K	c166100_g1	GO:0010467	-	-
c160520_g1	GO:0006879	K02150	C	c169505_g1	GO:0044444	K01190	G
c12562_g1	GO:0009860	K08486	U	c168059_g1	GO:0005737	K01193	G
c91879_g1	GO:0005794	K01051	M	c166703_g1	GO:0008152	K09886	G
c66386_g1	GO:0003700	K09286	K	c169570_g1	GO:0006654	K00006	C
c170743_g1	GO:0060562	K08282	TR	c169939_g2	GO:0005681	K03283	O
c105606_g1	-	-	X	c165556_g1	GO:0048678	K04564	P
c160214_g2	GO:0003700	K09286	K	c164327_g2	GO:0009790	K00700	G
c169585_g2	GO:0002199	-	-	c168288_g1	GO:0010811	K05360	S
c169587_g2	GO:0007623	-	-	c158227_g2	GO:0021747	K06573	P
c163757_g3	GO:0048205	-	U	c166104_g1	GO:0034599	-	-
c53049_g1	GO:0031981	-	T	c165768_g1	GO:0044444	K03248	J
c164227_g1	GO:0008574	K10396	Z	c153080_g2	GO:0009612	K08235	M
c42358_g1	-	-	X	c168377_g1	GO:0005886	K00033	G
c147819_g1	GO:0005739	-	TU	c161539_g1	GO:0042278	K00943	BK
c166486_g1	GO:0005634	K03626	K	c28300_g1	GO:0003700	K01177	G
c164619_g1	GO:0031406	K01596	C	c166554_g1	GO:0006631	K00908	R
c159888_g5	GO:0016020	K03125	K	c167429_g1	GO:0031931	-	R
c170157_g1	-	-	T	c165430_g2	GO:0005886	K00238	C
c161587_g1	GO:0044421	K08753	I	c160005_g1	-	-	S
c49705_g1	GO:0016020	K00517	Q	c166808_g1	GO:0070062	-	-
c169970_g1	GO:0005739	K08660	E	c157476_g1	-	-	X
c6033_g1	-	-	S	c167197_g2	GO:0008080	-	-
c170069_g3	GO:0034622	-	YU	c167885_g1	GO:0045199	K04513	TU
c164258_g2	GO:0003779	K06768	N	c150185_g2	GO:0003700	K09286	K
c163759_g1	GO:0019784	K03035	OT	c162665_g1	GO:0007608	-	R
c168695_g1	GO:0061732	K00162	C	c169452_g1	GO:0070210	K06067	B
c149600_g1	GO:0000329	-	-	c165875_g5	GO:0016020	-	-
c163551_g1	GO:0042803	K05279	R	c168703_g1	GO:0016020	-	U
c165103_g2	GO:0006431	K01869	J	c169904_g6	GO:0005905	-	-
c167395_g3	GO:0007595	K05658	Q	c148117_g1	GO:0005773	K00279	C
c190719_g2	GO:0048046	-	X	c189679_g1	GO:0009792	-	S
c164270_g1	GO:0005634	K01835	G	c166530_g2	GO:0055120	K05759	Z
c161311_g2	GO:0031312	K08269	R	c49140_g1	GO:0015297	K03327	R
c58347_g1	GO:0007114	K04393	R	c169954_g9	GO:0005886	-	-
c122437_g1	GO:0006364	K01529	A	c169260_g1	GO:0034605	K09556	O
c161191_g1	GO:1901700	K09422	-	c165246_g1	GO:0008286	K02731	O
c155750_g3	-	-	X	c196180_g1	GO:0005634	K04488	C
c159397_g4	GO:0005886	K02183	T	c24923_g1	GO:0003779	K01623	G
c164171_g1	GO:0042030	-	-	c166274_g1	GO:0006106	K01679	C

c158947_g1	GO:0005992	-	X	c164306_g1	GO:0005759	K03934	C
c147302_g1	GO:0042254	-	-	c163994_g4	GO:0016020	-	S
c146875_g6	-	K09286	-	c159967_g2	GO:0000212	K07375	Z
c166362_g1	GO:0065007	K10088	R	c167176_g1	GO:0042803	K00357	E
c169121_g1	GO:0005739	K10351	Z	c97782_g2	GO:0016020	-	X
c171348_g1	GO:0005634	K10760	J	c169495_g1	GO:0046577	K00129	C
c181837_g1	GO:0005634	K10629	-	c126878_g1	GO:0009611	-	S
c168306_g1	GO:0019538	K00415	O	c125150_g1	GO:0005739	K02917	J
c152826_g2	GO:0050794	-	-	c167820_g4	GO:0016020	K04437	Z
c165491_g1	GO:0055088	K03522	C	c157065_g1	GO:0001701	K01802	DA
c157779_g1	GO:0044424	-	-	c135971_g1	GO:0009611	K09422	K
c40571_g1	GO:0009403	K00059	I	c169314_g1	GO:0006886	K05236	X
c165226_g2	GO:0016020	-	-	c167627_g1	GO:0031012	K01349	O
c167005_g1	GO:0031647	-	A	c158315_g2	GO:0005789	K00476	O
c168022_g3	GO:0006260	K01262	E	c167335_g1	GO:0006351	K03122	-
c136792_g1	GO:0005634	K09419	K	c170080_g3	GO:0040011	K07611	NZ
c169068_g1	GO:0005882	K07611	DY	c150433_g1	-	-	S
c170069_g1	GO:0009987	-	-	c168991_g1	-	-	W
c168058_g1	GO:0005739	K01078	I	c136898_g1	GO:0005886	-	-
c167947_g3	GO:0001725	-	-	c159683_g1	GO:0003700	K09422	K
c168100_g1	GO:0034260	K04352	R	c168327_g1	GO:0016020	-	AR
c169485_g1	GO:0015031	-	U	c45337_g1	GO:0004722	-	-
c169493_g1	GO:0006351	-	-	c169374_g1	GO:0009507	K08831	R
c180211_g1	GO:0048046	K01218	GM	c167999_g1	GO:0008177	K00234	C
c168957_g4	GO:0005488	K10593	O	c164297_g1	GO:1903040	K03257	A
c169661_g1	GO:0051491	K03262	J	c57729_g1	-	-	X
c168246_g1	GO:2001253	-	S	c168757_g2	GO:0005933	-	-
c163953_g5	GO:0005886	K01179	G	c157933_g1	GO:0070062	-	S
c167696_g29	-	-	R	c167571_g1	GO:0005634	K02137	C
c170035_g3	GO:0060341	K06115	Z	c165992_g1	GO:0009887	K03347	O
c169142_g1	GO:0044421	K01256	IOVE	c187029_g1	GO:0005886	-	R
c161205_g1	GO:0006626	-	U	c168317_g1	GO:0090129	-	AR
c163860_g1	GO:0005515	K09141	S	c169284_g1	GO:0007084	K03456	T
c169141_g1	GO:0045087	K00507	I	c164173_g1	GO:0005811	K02130	C
c168786_g1	GO:0003779	K01622	G	c168048_g1	GO:0005576	K09485	O
c167787_g1	GO:0000389	K02977	A	c170052_g1	GO:0016020	K01652	EH
c165004_g1	GO:0098805	K06528	-	c161440_g2	GO:0001938	K09272	KLB
c144990_g1	-	-	R	c165640_g1	GO:0044237	K01078	G
c166400_g1	GO:0006644	K06460	R	c165942_g2	GO:0040011	K05637	W
c166994_g1	-	K02138	C	c167185_g1	GO:0043197	K06269	T
c167487_g1	GO:0005634	-	D	c168645_g1	GO:0006281	K02941	J
c169306_g2	GO:0070062	K02925	J	c123443_g1	GO:0005886	K02891	J
c122545_g1	GO:0005886	-	X	c169794_g20	GO:0006559	K10775	Q
c143554_g2	-	-	S	c157091_g2	GO:0005737	-	-
c168519_g1	GO:0070991	K00249	R	c168151_g1	GO:0051704	-	T
c169454_g1	GO:0033206	K10355	Z	c169979_g2	GO:0006631	K00059	I
c167606_g1	GO:0005654	K06883	Z	c167274_g1	GO:0005737	K01193	G
c167079_g1	-	K01091	R	c168239_g1	GO:0009649	K04635	T
c170365_g1	GO:0030414	-	-	c165154_g1	GO:0070062	K03100	U
c155178_g1	GO:0005634	K00485	Q	c163431_g1	GO:0009833	-	MG
c169344_g1	GO:0005886	K01265	R	c161510_g2	GO:0016020	-	-
c164288_g2	GO:0050774	K03083	K	c169810_g2	GO:0005576	K09448	R
c185603_g1	-	-	R	c168740_g1	GO:0032926	K03102	K
c167460_g2	GO:0043933	-	-	c63439_g1	GO:0003824	-	T

c148610_g2	GO:0051707	K08241	QV	c124782_g1	GO:0042254	K02915	J
c163996_g1	GO:0044444	K01363	O	c157328_g1	GO:0005488	K08076	O
c150774_g2	GO:0003700	-	KT	c163624_g1	GO:0045732	K03097	K
c166525_g1	GO:0045087	K00507	I	c161040_g1	GO:0005768	K07976	U
c167173_g1	GO:0070062	K02936	J	c157116_g3	GO:0005886	K00754	G
c154431_g1	GO:0045860	-	-	c28300_g2	GO:0003700	K01177	G
c168415_g1	GO:0009735	K00026	C	c167984_g1	GO:0005794	-	-
c169971_g1	GO:0005739	K10590	O	c165215_g1	GO:0060341	-	-
c162195_g1	GO:0000814	-	K	c165796_g1	GO:1901564	-	-
c29329_g1	GO:0006351	-	TR	c166730_g1	GO:0008134	K04497	-
c195758_g1	GO:0005886	-	R	c158707_g1	GO:0016020	K09553	-
c147108_g1	-	K02129	-	c133321_g2	GO:0009961	-	KT
c41405_g1	GO:0009617	-	V	c28503_g1	GO:0005773	K01287	O
c161397_g3	GO:0071495	K04591	T	c168853_g1	GO:0005634	K09680	H
c167869_g1	GO:0030433	K10086	G	c167141_g1	GO:0034220	-	W
c70690_g1	-	-	GR	c168070_g1	GO:0009719	K06238	W
c170132_g1	GO:0031124	K08877	A	c158742_g1	GO:0016020	-	M
c123765_g1	GO:0015114	-	-	c167820_g3	GO:0016020	K04437	Z
c129225_g1	GO:0055114	K00511	I	c159541_g1	-	-	W
c161616_g1	GO:0005794	K03676	O	c169961_g1	GO:1901701	-	T
c169617_g1	GO:0007269	K03709	TU	c125415_g2	GO:0048316	K02220	D
c169904_g4	GO:0016482	K06233	O	c167621_g1	GO:0030331	-	O
c169889_g2	GO:0031012	-	-	c168252_g1	GO:0005681	-	AR
c181648_g1	GO:0097067	K01363	R	c161384_g1	GO:0016032	K02920	-
c67372_g1	GO:0008152	K01674	R	c163678_g1	GO:0005576	-	T
c169539_g1	GO:0044271	K06278	X	c166627_g1	GO:0005515	K02871	J
c169427_g1	GO:0044609	-	R	c169153_g2	GO:0006368	-	K
c139890_g1	GO:0005507	K01251	H	c163717_g1	GO:0022627	K02969	J
c165254_g1	GO:0015421	K05656	Q	c161480_g1	GO:0015908	K05681	Q
c169775_g1	GO:0030728	-	Z	c186197_g1	GO:0044464	-	V
c168361_g1	GO:0042826	K10752	R	c164851_g1	GO:0005504	K00001	Q
c168023_g1	GO:0061630	K02207	O	c113835_g1	GO:0016020	-	S
c169174_g2	GO:0005739	-	VT	c190886_g1	GO:0005634	K00924	T
c166586_g1	GO:0051028	-	-	c167452_g1	GO:0030033	K08486	U
c165649_g2	GO:0051959	K10413	Z	c168081_g1	GO:0022627	K02987	J
c169305_g2	GO:0097655	K01365	O	c160984_g3	GO:0006351	K02999	K
c117343_g1	GO:0009639	K05282	QR	c153334_g1	GO:0016020	-	V
c168790_g1	GO:0030315	K00942	S	c167707_g1	GO:0005634	-	R
c23797_g1	GO:0009742	K01288	OE	c170165_g1	GO:0016020	K03032	O
c163088_g3	GO:0009860	K03305	E	c158509_g1	GO:1901700	K08873	TBLD
c176464_g1	-	-	X	c165365_g1	GO:0050790	-	TU
c123374_g1	GO:0008021	K07976	R	c169775_g2	GO:0061061	-	Z
c168690_g1	GO:0070688	K09667	R	c168009_g2	GO:0005488	K07994	OW
c166889_g1	GO:0005819	-	-	c166769_g1	GO:0008286	K03038	O
c126722_g1	GO:0005886	K00924	T	c169840_g1	GO:0004618	K00927	G
c63509_g1	GO:0003700	-	K	c169731_g7	GO:0006644	-	T
c153473_g2	GO:0005634	-	R	c170859_g1	-	-	X
c167552_g1	GO:0016323	-	-	c166512_g1	-	-	X
c39757_g1	GO:0080082	K10746	L	c169271_g1	GO:1901741	-	TU
c157395_g1	GO:0005219	K09568	O	c31398_g1	GO:0001101	-	S
c142453_g1	GO:0009753	-	X	c186760_g1	GO:0016023	-	-
c161373_g1	GO:0002199	K09494	O	c167378_g1	GO:0033181	K02155	C
c165672_g1	-	-	W	c166140_g2	GO:0072669	K01509	A
c169604_g1	GO:0005634	K01255	R	c166254_g1	GO:0003677	K00088	F

c169365_g1	GO:0016020	-	-	c160773_g2	GO:0006952	-	-
c168884_g3	GO:0048046	K00430	R	c168214_g1	GO:0008430	K00432	O
c161823_g3	GO:0009785	K09422	K	c168982_g1	GO:0000910	-	-
c152953_g2	GO:2001020	-	S	c169423_g1	GO:0008286	K03065	O
c156562_g1	GO:0007165	K08707	T	c167986_g1	GO:0042327	K10482	R
c196860_g1	GO:0006360	K03020	K	c169263_g2	GO:0046982	-	B
c156856_g2	GO:0003700	K09338	K	c166430_g1	GO:0048046	K00030	E
c175241_g2	GO:0005576	-	-	c161794_g1	GO:0046755	-	S
c168955_g1	GO:0008299	K00869	I	c144461_g1	GO:0044424	-	-
c169463_g1	GO:0044444	K07189	OT	c170181_g1	GO:0004872	K06233	O
c167436_g1	GO:0042805	K06763	Z	c169386_g1	GO:0016310	-	-
c153990_g3	GO:0005886	K05280	Q	c168949_g3	GO:0003700	-	S
c169887_g4	GO:0005739	K02145	C	c169398_g1	GO:0004054	K00934	C
c169076_g1	GO:0098554	K09505	O	c168356_g1	GO:0004632	K01922	R
c122610_g1	GO:0033181	K02151	C	c169796_g4	GO:0005488	K00924	T
c169069_g4	GO:0001655	K02599	T	c149319_g1	GO:0003700	-	R
c166456_g1	GO:0005488	-	-	c180919_g1	GO:0005737	-	W
c170109_g1	GO:0031012	K01360	O	c163650_g4	GO:0010080	K09285	K
c42235_g1	GO:0055114	-	-	c161149_g1	GO:0048255	K01802	A
c158426_g1	-	K02268	-	c49768_g1	GO:0009961	-	KT
c159571_g4	GO:0080168	K03305	E	c122451_g2	-	-	X
c149402_g3	GO:0000166	K00058	C	c156692_g1	GO:0032266	-	-
c169892_g2	GO:0005768	K02154	C	c169290_g1	GO:0098542	K09418	K
c167743_g1	-	-	W	c169387_g1	GO:0035719	K09503	O
c153485_g1	GO:0030182	-	-	c168073_g1	GO:0044424	K00547	E
c68143_g1	GO:0044249	K04125	QR	c169072_g1	GO:0044237	K01426	J
c146621_g1	GO:0016266	K00710	O	c162075_g1	GO:0030433	K07151	O
c155258_g3	GO:0015578	-	-	c167313_g1	GO:0016020	K02734	O
c137843_g1	GO:0003700	-	KT	c168202_g2	-	-	A
c125700_g1	GO:0009699	K00475	QR	c196416_g1	GO:0009753	-	-
c167444_g1	GO:0035965	K07509	I	c169346_g1	GO:0098542	K00430	PQ
c167977_g1	GO:0002199	K09499	O	c168851_g1	GO:0009682	K00517	QI
c168162_g2	GO:0009507	-	-	c155258_g2	GO:0046323	K08070	R
c152792_g2	GO:0016020	K01509	K	c39931_g1	GO:0009723	K09286	K
c169904_g3	GO:0060341	-	-	c157042_g1	GO:0005634	K00826	E
c166828_g3	GO:0016020	K05695	T	c169119_g1	GO:0018279	K00730	O
c168538_g1	GO:0044767	-	X	c168600_g3	GO:0016020	-	-
c167868_g5	GO:0019899	K06530	O	c162660_g1	GO:0022627	K02975	J
c161497_g1	GO:0008286	K02737	O	c196504_g1	GO:0042349	-	S
c154944_g1	GO:0016324	K10364	Z	c196474_g1	GO:0005886	K02183	T
c165742_g3	GO:0048046	-	T	c166993_g1	GO:0000989	-	R
c164592_g1	GO:0030619	-	A	c168085_g1	GO:0005654	-	A
c168268_g1	GO:0006734	K00030	E	c196163_g1	GO:0052325	-	RQ
c169108_g1	GO:0061635	K01376	-	c169599_g1	GO:0043204	K03102	A
c170069_g2	GO:0009987	-	YU	c160861_g1	-	K06867	-
c163715_g1	-	-	S	c165565_g1	GO:0005886	-	T
c166520_g1	GO:0048585	K10093	W	c168199_g1	GO:0016592	-	K
c168814_g1	GO:0043292	K00799	O	c165085_g2	GO:0045167	K08825	-
c192683_g1	GO:0044424	-	I	c161502_g1	GO:0005634	-	-
c159992_g1	-	-	X	c164369_g1	GO:0018279	-	S
c165617_g1	GO:0005789	-	V	c165538_g1	GO:0042593	K10523	DR
c168617_g1	GO:0005634	K01501	FR	c164627_g1	GO:0005634	K03768	O
c43062_g1	-	-	R	c128727_g1	GO:0015807	-	E
c159677_g1	GO:0048561	-	K	c168409_g1	GO:0019843	K02940	J

c166859_g1	-	-	R	c170357_g1	GO:0003700	K09286	K
c64492_g1	GO:0016020	K00517	Q	c169320_g6	GO:0005764	K05126	-
c168057_g1	GO:0015908	K00624	I	c147690_g1	GO:0006614	K02889	J
c169281_g1	GO:0051437	-	R	c169337_g1	GO:0004177	K01423	OR
c165350_g1	GO:0044249	K00022	Q	c193102_g1	GO:0005739	K09510	-
c165285_g1	GO:0050794	-	-	c180612_g1	GO:0048046	-	T
c101685_g1	GO:0090406	K08070	R	c170960_g1	-	K01206	G
c162838_g1	GO:0071817	-	S	c158510_g4	GO:0003779	-	-
c156489_g1	GO:0006986	-	-	c125081_g1	GO:0045087	K00924	T
c166514_g1	GO:0019551	K00813	E	c166013_g1	GO:0005739	-	-
c106589_g1	GO:0006342	K10646	R	c169800_g1	-	-	IG
c167291_g1	GO:0032000	K01897	I	c166652_g1	GO:0005789	K06890	V
c124340_g1	GO:0010467	-	R	c185794_g1	-	K04618	S
c168328_g3	GO:0006260	-	O	c166457_g1	GO:0007021	K09496	O
c149565_g1	GO:0003700	-	KT	c165355_g1	GO:0008021	K02146	C
c168013_g3	-	K06036	X	c166600_g1	GO:0010171	K09290	Z
c165446_g1	GO:0021675	K06272	T	c169390_g1	GO:0036091	K04494	KL
c181204_g1	GO:0003700	K09286	K	c168588_g1	GO:1901564	K01078	I
c168700_g1	GO:0005737	-	A	c168333_g2	GO:0070062	-	C
c169341_g1	GO:0005886	K00026	C	c165198_g2	GO:0005886	K01090	T
c156986_g1	GO:0000398	-	A	c168069_g1	GO:0005886	K02930	A
c169848_g1	GO:0016020	K03864	U	c166781_g1	GO:0010948	K00777	Z
c169751_g1	GO:0006511	K03029	O	c132145_g1	GO:0030687	-	-
c166833_g1	GO:0005634	K08769	C	c167535_g1	GO:0007076	-	-
c164524_g2	GO:0003824	K06972	O	c157307_g1	GO:0022627	K02949	J
c167273_g1	GO:0008152	K01175	R	c166968_g1	GO:0030968	K03110	U
c160140_g1	-	-	R	c165647_g1	GO:0016020	-	A
c162302_g1	GO:0048443	K05658	Q	c166725_g1	GO:0086004	-	-
c139115_g1	GO:0043231	K02183	T	c133101_g1	GO:0006360	K03008	K
c157397_g2	GO:0005737	-	-	c168863_g1	GO:0048598	-	-
c166797_g1	GO:0006665	-	-	c160313_g1	GO:0086004	-	-
c43184_g1	GO:0044767	-	-	c167340_g1	GO:0003974	K01784	M
c170980_g1	-	-	X	c169821_g1	GO:0004029	K00140	C
c168006_g2	GO:0048514	K06237	W	c159364_g2	GO:0008152	K00307	QG
c165089_g1	GO:0048046	-	S	c129613_g1	GO:0005634	K10258	I
c151967_g1	GO:0006351	-	KT	c168477_g1	GO:0044421	K05022	P
c164623_g1	GO:0006289	K03036	O	c162736_g1	GO:0005524	K01610	C
c164767_g1	GO:0044822	K00914	R	c169920_g1	GO:0005739	K03178	O
c162951_g3	GO:0009506	-	S	c168865_g1	GO:0051052	-	K
c169978_g1	-	-	W	c170022_g6	GO:0048870	K10382	Z
c164565_g1	-	-	T	c17872_g1	GO:0009785	-	R
c144290_g1	GO:0005524	K00789	H	c146380_g1	GO:0003700	K09286	K
c163667_g1	GO:0051959	K10418	Z	c168999_g3	GO:0009992	K00924	T
c132993_g2	GO:0010091	K09422	K	c169035_g5	-	-	X
c196563_g1	GO:0009266	-	X	c160557_g1	GO:0070993	K03246	I
c166341_g2	GO:0022904	-	-	c153065_g5	GO:0005576	K04436	T
c153088_g1	GO:0040029	K05628	B	c135970_g1	GO:0097060	K05313	PET
c162558_g1	GO:0022627	K02964	J	c166999_g2	GO:0009117	K00059	IQ
c169588_g1	GO:0008080	K00657	-	c162463_g1	-	K03959	-
c169931_g1	GO:0051704	K06233	T	c49973_g1	GO:0005488	K01324	-
c169722_g1	GO:0016020	K06948	R	c152320_g2	GO:0003700	K09264	K
c166305_g1	GO:0005654	-	A	c142939_g2	GO:0060341	K09259	R
c196900_g1	GO:0005222	K05391	PT	c198496_g1	GO:0008137	K00329	CD
c169591_g1	GO:0044822	-	R	c118147_g2	GO:0009832	-	X

c163276_g1	GO:0009073	K01555	G	c169652_g1	GO:0070613	K01647	C
c166814_g1	GO:0007049	K10317	R	c169971_g2	GO:0060341	K10590	O
c169879_g1	GO:0005576	K01697	E	c152320_g3	GO:0048481	K09264	K
c168260_g3	GO:0008593	-	TU	c168604_g1	GO:0005085	K03232	K
c159299_g1	GO:0070062	K02894	J	c169100_g1	GO:0046949	K00252	I
c165236_g2	GO:0048193	K05236	U	c164532_g2	GO:0086004	-	-
c163305_g1	GO:0005488	K10593	O	c153006_g1	GO:0000398	-	A
c121880_g1	GO:0010103	K04416	R	c167594_g2	GO:0005783	K01228	G
c167054_g1	GO:0022904	K02136	C	c168824_g2	GO:0005524	K01870	J
c159160_g1	GO:0022627	K02953	J	c166770_g1	GO:0016020	K03039	O
c165395_g1	GO:0008152	K10758	D	c130909_g1	GO:0001054	K03016	K
c164390_g2	GO:0070735	K10695	O	c151796_g4	GO:0003700	-	-
c168775_g4	GO:0003729	-	-	c152651_g1	GO:0031490	-	S
c169754_g2	GO:0046903	K00493	Q	c169235_g1	GO:0016020	-	-
c166789_g1	GO:0070735	K10597	O	c138476_g1	GO:0032502	K09230	-
c169583_g1	GO:0030279	K00500	E	c102953_g1	GO:0006364	K02936	A
c137245_g1	GO:0005654	-	A	c168887_g1	GO:0005488	K05501	D
c167732_g1	GO:0004872	K06252	TW	c169013_g1	GO:0005778	-	-
c192247_g1	GO:0080030	K08233	Q	c165660_g1	GO:0006368	K03006	K
c168326_g4	GO:0010073	K00985	A	c168699_g1	GO:0005794	K01711	G
c134292_g1	GO:0019899	K00326	C	c170211_g3	GO:0050896	-	I
c168410_g1	GO:0080142	K02183	RT	c165879_g5	GO:0005634	-	-
c143741_g1	GO:0043425	-	KT	c168082_g1	GO:0019222	K10371	Z
c170367_g1	GO:0048046	K01569	V	c159065_g2	GO:0048046	-	T
c169225_g1	GO:0005789	K00636	I	c161709_g1	GO:0016020	K03036	BK
c167510_g1	GO:0018279	-	U	c166038_g1	GO:0006355	-	L
c168950_g1	GO:0004185	K01287	OE	c169443_g1	GO:0030331	K01529	K
c167668_g4	GO:0016274	K10297	R	c160327_g1	GO:0005789	K04571	U
c150389_g1	GO:0016998	K01183	R	c169991_g2	GO:0048731	-	-
c169177_g1	GO:0018279	K00730	O	c185786_g1	GO:0016020	K08900	O
c170004_g1	GO:0045087	K01363	R	c158990_g1	GO:0010286	-	P
c169201_g1	GO:0031124	K09564	A	c165257_g1	GO:0030315	K04348	T
c132727_g1	GO:0040035	K06689	O	c145087_g2	GO:0030151	-	-
c169381_g1	GO:0070887	K07611	DY	c159805_g1	GO:0044419	-	R
c170193_g3	GO:0005634	K01680	CE	c167794_g1	GO:0019843	K02938	J
c149663_g1	GO:0009961	-	KT	c166318_g3	GO:0006950	-	-
c153505_g1	GO:0060341	-	-	c165941_g1	GO:0005739	K02503	T
c175484_g1	GO:0009753	-	-	c168879_g1	GO:0022627	K02981	J
c46769_g1	GO:0043226	K09542	-	c162126_g1	GO:0030315	K05850	P
c170964_g1	GO:0005618	-	-	c81124_g2	GO:0044421	-	X
c48865_g1	GO:0048046	K00430	R	c167853_g1	GO:0043197	K05849	PT
c165192_g1	GO:0030308	K01758	E	c169170_g1	-	-	L
c166058_g1	-	-	X	c147979_g1	GO:1901137	K01840	G
c166524_g4	GO:0070097	K04601	S	c165398_g1	GO:0008152	K03964	C
c168065_g1	GO:0016020	-	A	c164761_g1	GO:0034315	K07937	U
c153414_g1	GO:0010105	K09286	K	c162190_g1	GO:0009873	-	PQ
c167598_g1	GO:0008340	K03030	OT	c170018_g7	GO:0035556	K00924	T
c169730_g3	GO:0006355	-	A	c144243_g2	GO:0005634	-	X
c166057_g3	GO:0007399	K00699	GC	c169675_g1	GO:0008340	K01509	O
c167958_g1	GO:0005886	K01552	Q	c168428_g1	GO:0005739	-	T
c169565_g1	GO:0032496	K05666	Q	c169678_g1	GO:0005886	K01692	I
c168141_g1	GO:0044237	K00025	C	c170107_g2	GO:0042803	K00615	G
c168468_g2	GO:0034199	K07376	R	c168823_g1	GO:0060341	K01044	T
c180622_g1	GO:0005737	K08237	GC	c161620_g1	GO:0019843	K02868	J

c170247_g1	GO:0032501	-	I	c190903_g1	GO:0009834	-	S
c167627_g2	GO:0005576	K08654	O	c165505_g1	GO:0005739	K02865	J
c168202_g3	GO:0022414	K00985	A	c169909_g1	GO:0031012	K00873	G
c168960_g1	GO:0006678	K00139	E	c169214_g1	GO:0048365	K07897	R
c161437_g1	GO:0005634	-	S	c169127_g1	GO:0000389	-	A
c168505_g3	GO:0005515	-	R	c169805_g1	GO:0048468	K05099	T
c163818_g1	GO:0044429	K02886	J	c169897_g2	GO:1902904	K03283	O
c167225_g1	GO:0006710	K00120	Q	c166345_g1	GO:0006979	K00658	C
c169847_g6	GO:0005634	-	K	c166990_g4	GO:0003779	K05691	W
c169932_g1	GO:0005576	K07960	T	c161917_g1	GO:0009617	K06071	L
c163970_g3	GO:0044237	K07566	R	c166206_g1	GO:0009507	K10580	O
c159154_g1	GO:0022904	K02267	C	c166351_g1	GO:0048046	K00430	R
c169939_g1	GO:0005681	-	-	c163815_g1	GO:0000147	-	F
c143678_g1	GO:0005783	-	P	c48500_g1	GO:0006342	K01509	KL
c165527_g1	GO:0005634	K03138	K	c168515_g1	GO:0070062	K09523	O
c168410_g2	GO:0031326	K02183	RT	c168778_g1	GO:0005739	-	-
c168798_g1	GO:0008286	K03061	O	c166658_g1	GO:0005634	-	K
c169078_g1	GO:0060255	K00599	K	c170119_g1	GO:0006826	K01301	OPR
c169849_g1	GO:0015630	K00134	G	c164669_g3	GO:0003700	K09286	K
c165952_g1	GO:0008286	K02726	O	c161750_g1	GO:0009793	K01887	J
c161441_g1	GO:0031124	-	A	c165999_g1	GO:0044430	-	-
c176834_g1	-	-	X	c169017_g1	GO:0009617	K04692	A
c169423_g2	GO:0008286	K03065	O	c164177_g1	GO:0016020	K03843	M
c168310_g1	GO:0044444	K04649	T	c9993_g1	GO:0005886	-	S
c153788_g1	GO:0003008	K06573	P	c191800_g1	GO:0005783	-	R
c165544_g1	GO:0044822	K03236	J	c116639_g1	GO:0031463	K03872	K
c146638_g1	GO:0009792	K00235	C	c169946_g2	GO:0008286	K03028	O
c167556_g1	GO:0005634	K02880	J	c117714_g2	GO:0003700	-	S
c145163_g1	GO:0008194	K08237	GC	c124171_g2	GO:0001701	-	-
c151696_g4	-	-	X	c167379_g1	-	-	X
c17716_g3	GO:0005886	K06900	I	c195769_g1	GO:0031408	K06900	I
c159065_g1	GO:0048046	-	T	c159320_g1	GO:0009785	K09422	K
c167541_g2	GO:0005886	K00924	T	c164682_g1	GO:0005886	-	U
c165024_g1	GO:0009860	K05929	IR	c166157_g1	GO:0006631	K01634	E
c153875_g1	GO:0009735	-	R	c168838_g1	GO:0035363	K06959	A
c169837_g1	GO:0005576	K01044	T	c164527_g1	-	-	X
c169323_g1	GO:0005739	K01911	I	c155590_g1	GO:0009860	-	-
c164019_g1	GO:0032024	K01551	P	c5829_g1	GO:0005634	K03936	C
c165227_g2	GO:0005634	K09114	-	c153943_g1	GO:0001919	-	U
c180741_g1	GO:0030905	-	V	c167873_g7	GO:0035556	-	-
c169927_g2	GO:0031931	-	OJ	c150633_g1	GO:0016161	-	-
c166530_g3	GO:0003779	K05759	Z	c170040_g1	GO:0046667	K04646	U
c167200_g6	GO:0009267	-	U	c137475_g1	GO:0031012	K01342	OR
c168341_g1	GO:0065007	K01287	OE	c165334_g1	GO:0030163	K01436	E
c169624_g1	GO:0004735	K00286	E	c158180_g1	GO:0000387	K04796	A
c168602_g1	GO:0005215	-	T	c146879_g1	-	-	T
c166908_g1	GO:0001851	-	O	c168590_g1	GO:0016020	-	JK
c131095_g1	GO:0016020	K00517	Q	c157964_g1	GO:0008466	K00750	G
c167517_g2	GO:0015115	K09874	G	c131390_g1	GO:0009694	K00517	QI
c164349_g5	GO:0003700	K09287	TK	c161241_g1	GO:0006351	-	-
c166730_g2	GO:0009887	K04497	R	c26182_g1	-	-	C
c163088_g2	GO:0009860	K03305	E	c166872_g1	GO:0008170	K00599	AR
c159612_g1	GO:0003700	-	KT	c63206_g2	GO:0003700	K09286	K
c162950_g2	GO:0005739	K01873	J	c169809_g1	GO:0036091	K01509	KL

c167370_g3	GO:0010769	K06756	T	c167301_g1	GO:0016579	-	TO
c167910_g3	GO:0005992	K00697	G	c168669_g1	GO:0005886	K00022	I
c161436_g1	GO:0003924	K06948	R	c167800_g2	GO:0032000	K01897	I
c143270_g1	GO:0005504	K00001	Q	c168307_g1	GO:0042769	-	-
c169109_g1	GO:0009507	K02985	J	c151144_g1	GO:0019843	K02870	J
c169892_g1	GO:0006879	K02154	C	c159185_g4	GO:0006508	K01289	OE
c169139_g2	GO:0044444	-	-	c161416_g2	GO:0005739	K00129	C
c167299_g1	GO:0006879	K02149	C	c168158_g2	-	-	X
c163346_g1	GO:0000398	K09528	O	c175321_g1	GO:0010033	K01528	UR
c168943_g1	GO:0022627	K02997	J	c168397_g2	GO:0030182	-	A
c86591_g1	GO:0048046	-	S	c118031_g1	GO:0044249	-	RQ
c164509_g1	GO:0007093	K02896	J	c137531_g1	GO:0051865	-	R
c169047_g1	-	-	R	c162742_g4	GO:0032501	K01175	V
c191647_g1	GO:0032543	-	J	c167518_g1	-	-	V
c167227_g1	GO:0010923	-	-	c167283_g1	GO:0097308	K01802	O
c170022_g1	GO:0045647	K10382	Z	c169585_g1	GO:0002199	K09495	O
c167727_g1	GO:0044421	K01298	S	c161383_g1	GO:0044281	K03952	C
c163074_g1	GO:0005783	-	-	c170059_g1	GO:0005739	K09239	R
c190891_g1	GO:0016023	-	-	c127554_g1	GO:0005515	K02954	J
c118485_g1	GO:0004055	K01940	E	c167612_g1	GO:0018444	K03266	J
c165272_g1	GO:0030509	-	-	c167351_g1	GO:0005783	-	U
c160033_g1	GO:0044822	-	R	c164437_g1	GO:0006979	-	-
c168715_g1	GO:0051591	K00456	E	c169792_g1	GO:0005737	K00001	CR
c169598_g3	GO:0009793	K03217	T	c165148_g1	GO:0003729	K01855	J
c149234_g2	GO:0008171	K05279	R	c24145_g1	-	-	S
c168827_g1	GO:0022627	K02984	J	c167701_g2	GO:0048441	K09589	QI
c165562_g2	GO:0016568	-	BK	c167581_g1	GO:0005634	K02727	O
c168307_g2	GO:0042769	K02927	DO	c163800_g3	GO:0046543	-	O
c164530_g1	GO:0015630	K10631	-	c31778_g1	GO:0048046	-	RI
c169506_g1	GO:0008286	K02729	O	c168075_g5	GO:0009833	-	GE
c168244_g1	GO:0008152	K09876	G	c160894_g1	GO:0000281	K05868	D
c164292_g1	GO:0008080	K00181	S	c133497_g1	GO:0015807	-	-
c151421_g1	GO:0009313	K03115	TDK	c165559_g1	GO:0042493	-	-
c164901_g3	GO:0030017	-	-	c146349_g1	GO:0005886	K00224	RQ
c135918_g1	GO:0003700	K09286	K	c148610_g1	GO:0051707	K08241	QV
c165424_g1	GO:0016310	K00852	G	c27848_g1	GO:0008514	-	Q
c165748_g1	GO:0016020	-	-	c172779_g1	GO:0022627	K02976	J
c181140_g1	GO:0005488	K03304	X	c124137_g1	-	-	S
c168836_g1	GO:0005768	K01072	-	c156441_g1	GO:0005886	K01193	G
c166681_g1	GO:0004775	K01903	C	c122022_g3	-	-	X
c162831_g1	GO:0045087	K05387	PET	c160895_g1	GO:0034626	K10203	I
c152961_g1	GO:0042574	K00128	-	c166493_g1	GO:0004568	-	-
c164630_g3	GO:0003700	K09422	K	c56891_g1	GO:0009699	K05278	QR
c169889_g1	GO:0071556	K08057	O	c142819_g3	GO:0048468	K10260	R
c167486_g1	GO:1902042	K06890	T	c167586_g1	GO:0071287	K09490	O
c177109_g1	GO:0005488	K09060	K	c163330_g1	GO:0005634	K02921	J
c168084_g1	GO:0008286	K03064	O	c155643_g2	GO:0005524	K00924	T
c145361_g1	GO:0005886	K00924	T	c42787_g1	GO:0050793	-	S
c150233_g4	-	-	X	c48518_g2	GO:0016020	K00517	QI
c164407_g2	GO:0005634	-	AR	c168479_g1	GO:0005782	K00827	R
c170141_g3	GO:0016020	K06560	-	c66164_g2	GO:0005634	-	-
c125655_g1	GO:0070551	-	J	c161026_g1	GO:0022008	K02903	-
c162535_g1	GO:1901566	-	-	c166849_g5	GO:0003700	K09286	K
c165853_g2	GO:0005789	-	U	c166955_g1	GO:0005886	-	-

c169130_g2	GO:0031012	K09487	O	c156643_g2	GO:0016020	-	-
c167047_g2	GO:0005783	K05280	QI	c168746_g1	GO:0022627	K02989	J
c167284_g2	GO:0016020	-	-	c170193_g2	GO:0043566	K01680	CE
c165434_g2	GO:0005739	K05971	K	c165214_g2	GO:0031369	K03252	J
c136361_g1	GO:0009793	K09522	K	c131923_g1	GO:0003824	-	-
c165946_g1	GO:0005739	K01868	J	c66521_g1	GO:0009617	-	V
c167634_g1	GO:0005634	K10582	O	c166929_g1	GO:0030030	K03130	K
c168948_g2	GO:0005524	K06185	EJ	c150251_g1	GO:0040002	K02087	T
c144256_g3	GO:0010280	K01710	G	c162191_g1	GO:0044464	K00924	T
c167319_g1	GO:0016328	-	S	c167146_g1	GO:0042254	K01529	A
c134858_g1	GO:0003700	K09422	K	c156243_g2	GO:0003700	K09286	-
c169477_g1	GO:0006351	-	K	c168254_g1	GO:0006950	K08136	W
c164684_g1	GO:0005739	K02263	C	c163778_g4	GO:0035826	-	-
c164954_g1	GO:0005938	K10351	T	c169934_g2	GO:0060562	-	-
c26597_g1	GO:0005886	K00754	G	c150647_g1	GO:0005634	K00522	P
c169563_g1	GO:0048227	K00680	TU	c136133_g1	GO:0009267	-	U
c170040_g2	GO:0046667	K04646	U	c158832_g1	GO:0098542	K07140	R
c169962_g1	GO:0048523	-	R	c166579_g1	-	-	S
c118646_g1	GO:0048046	-	-	c185998_g1	GO:0005515	K03686	-
c27266_g1	GO:0048046	-	-	c165375_g2	GO:0005739	K00525	F
c161748_g1	GO:0031012	K01342	OR	c169325_g1	GO:0001944	-	DR
c161300_g1	GO:0022904	K02134	C	c169102_g2	GO:0033290	K03242	J
c131236_g1	GO:0003700	K09286	K	c158775_g1	GO:0005634	-	R
c191102_g1	GO:0043226	K08282	Z	c168256_g1	GO:0098542	K01373	R
c167565_g1	GO:0042769	-	-	c168938_g2	GO:0005886	K00487	QI
c168481_g1	GO:0048731	K00908	S	c165238_g1	GO:0042345	K03386	O
c169320_g1	GO:0045893	K04362	T	c49768_g2	GO:0009961	-	-
c153446_g2	GO:0009611	K09422	K	c165791_g1	GO:0005537	-	-
c168019_g2	GO:0005739	K01915	E	c165207_g1	GO:0016020	K01509	A
c145972_g2	GO:0015297	K03327	R	c169253_g1	GO:0009612	K08235	M
c162137_g1	GO:0030239	K00907	Z	c162000_g2	GO:0003779	K09355	K
c166594_g1	GO:0022627	K02995	J	c167637_g2	GO:0045859	-	-
c166019_g1	GO:0005768	K00680	T	c159388_g1	GO:0005634	-	-
c40378_g1	GO:0048046	K00368	Q	c166248_g2	GO:0003700	-	D
c170202_g1	GO:0006325	-	-	c123139_g1	-	-	X
c170035_g4	GO:0060341	K06115	Z	c168899_g1	-	-	V
c163655_g1	GO:0050793	-	-	c163241_g1	GO:0031326	K02183	RT
c167170_g2	GO:1902589	K07196	-	c168002_g2	GO:0005789	K00021	I
c167562_g1	GO:0005737	-	-	c167027_g3	GO:0003682	-	A
c166543_g1	GO:0048856	K01540	P	c166918_g1	GO:0048255	K03102	R
c152653_g2	GO:0016032	-	-	c170079_g1	GO:0006879	K02147	C
c166692_g1	GO:0005886	K03234	J	c165948_g1	GO:0070062	K07508	I
c163339_g3	-	-	X	c161577_g1	GO:0016324	K07199	G
c154142_g1	GO:0070062	K02910	J	c168391_g1	GO:0003729	K03257	A
c113463_g1	GO:0005739	-	U	c157743_g1	GO:0031435	K06689	O
c68021_g1	GO:0016020	K01568	EH	c121826_g1	GO:0006950	-	-
c169495_g2	GO:0005739	K00129	C	c167312_g1	GO:0070345	K04683	K
c143890_g2	GO:0006760	K00059	R	c169074_g1	GO:0061732	K00627	C
c24640_g1	GO:0005515	-	-	c166927_g2	GO:0003700	K09286	K
c169715_g2	GO:0042063	K06243	W	c165727_g1	GO:0008340	K06630	O
c168370_g1	GO:0031012	K00430	PQ	c169809_g2	GO:0043167	K01509	R
c167879_g2	-	-	K	c166837_g3	GO:0071555	K00694	MG
c169927_g1	GO:0031931	-	OJ	c169637_g2	GO:0071495	K06115	Z
c170013_g3	GO:0046889	K08675	O	c166304_g5	GO:0005739	K01342	OR

c161870_g1	GO:0000398	-	K	c51126_g1	GO:0005783	-	R
c157269_g2	GO:0016165	K00454	I	c170107_g1	GO:0042803	K00615	G
c159948_g1	GO:0005654	-	-	c157947_g2	GO:0006011	K00012	GT
c164521_g1	GO:0022904	-	-	c168606_g2	GO:0035072	K01509	K
c169937_g1	GO:0010033	K01920	Q	c164119_g1	GO:0006364	K02898	J
c162721_g2	GO:0034214	-	-	c170146_g1	GO:0005524	K01959	C
c169163_g2	GO:0003810	-	-	c168234_g1	GO:0055114	K00365	Q
c158572_g4	GO:0005634	K00472	E	c168845_g1	GO:0005488	-	I
c169835_g2	GO:0071495	-	-	c162435_g1	GO:0000389	-	A
c166501_g1	GO:0019054	K08332	U	c123560_g1	GO:0006979	K00430	R
c168344_g2	GO:0044444	-	-	c150516_g1	GO:0009705	K03327	R
c166083_g1	GO:0035072	K01529	L	c168942_g2	GO:0005634	K03233	O
c27898_g1	GO:0048678	K04453	-	c152254_g1	GO:0003700	-	KT
c130794_g1	GO:0022627	K02973	J	c115636_g1	GO:0005739	-	S
c169958_g2	GO:0048870	-	-	c168696_g1	GO:0016568	-	-
c146795_g3	GO:0005739	K00888	T	c166483_g1	GO:0019784	K09602	S
c166886_g1	GO:0044707	K06084	Z	c169274_g1	GO:0009834	K02366	GMW
c181328_g1	GO:0034219	-	R	c170047_g2	GO:0043170	-	-
c168998_g1	GO:0044444	K03260	J	c186391_g1	GO:0071944	-	S
c156472_g1	GO:0014041	K07860	R	c185671_g1	GO:0005783	K01199	G
c170081_g1	GO:0016568	-	S	c165726_g1	GO:0047066	K00432	O
c168567_g1	GO:0050017	K01738	E	c169824_g3	GO:0005730	K02133	C
c163207_g1	GO:0005654	K03105	U	c164642_g7	GO:0045893	K10395	Z
c175576_g1	GO:0005794	-	S	c158225_g1	GO:0005739	K01527	K
c168631_g1	GO:0032024	K00261	E	c152753_g1	GO:0003700	K09338	K
c168753_g1	GO:0005158	-	U	c175319_g1	GO:0003700	-	KT
c22576_g1	GO:0098542	K00924	T	c157826_g1	GO:0005634	K07179	TR
c155825_g2	GO:0016020	-	S	c165875_g1	-	K08900	O
c145581_g1	GO:0010089	K03920	I	c161034_g1	GO:0055114	-	-
c167261_g1	GO:0042826	K00653	-	c167479_g2	GO:0051707	K10139	V
c168962_g1	GO:1901687	K01919	H	c190910_g1	GO:0032559	K00666	I
c165660_g4	GO:0006281	K03006	-	c169755_g1	GO:0042826	-	A
c167866_g1	GO:0005773	K00279	C	c162733_g1	-	-	L
c39816_g1	GO:0044444	-	R	c167289_g1	GO:0003729	K02918	J
c169874_g1	GO:0022904	K02132	C	c135893_g2	GO:0006979	K00754	G
c166128_g1	GO:1903955	-	S	c167139_g1	GO:0022627	-	-
c152678_g1	GO:0042254	-	-	c152269_g1	GO:0050681	-	-
c168559_g1	GO:0010888	-	-	c167769_g1	GO:0086004	-	-
c131771_g2	GO:0044444	K09753	V	c169828_g1	GO:0044444	K07195	U
c167541_g1	GO:0005886	K08286	T	c168683_g2	GO:0033523	-	-
c164233_g2	GO:0042826	-	NI	c169843_g1	GO:0004866	K01072	O
c162387_g1	GO:0034098	-	S	c169916_g1	GO:0005634	-	K
c158723_g1	GO:0045202	-	-	c175582_g1	GO:0048046	K00430	R
c157118_g1	GO:2000032	-	-	c167848_g1	GO:0005813	K02183	T
c163448_g3	GO:0019538	K08900	O	c157336_g3	GO:0006336	K01509	KL
c166995_g1	GO:0070888	K00029	C	c66412_g1	GO:0005634	-	-
c163979_g1	GO:0016020	K01309	O	c164111_g1	GO:0007275	K03687	O
c167993_g1	GO:0005634	-	DZ	c161722_g1	-	-	S
c170216_g1	GO:0007512	K10352	Z	c163362_g1	GO:0006816	K10645	-
c164561_g1	GO:0003779	-	-	c164024_g1	GO:0006123	K02264	C
c169422_g2	GO:0044421	-	T	c139890_g3	GO:0005507	K01251	H
c175299_g1	GO:0005576	K01569	V	c122156_g1	-	-	W
c157588_g1	GO:0005789	K07976	U	c166569_g1	GO:0005488	-	I
c89444_g1	GO:0022627	K02971	J	c160140_g2	GO:0048568	K09259	R

c158233_g1	GO:0016020	K02265	C	c170216_g2	GO:0014728	K10352	Z
c68981_g1	GO:0016787	K07025	R	c166539_g1	GO:0006366	-	A
c170155_g6	GO:0040029	K10706	L	c169090_g1	GO:0045087	K01369	O
c169085_g1	GO:0072697	K05762	Z	c168701_g1	GO:0055059	K04382	T
c157921_g2	GO:0030315	K04515	T	c113299_g1	GO:0009507	K08193	G
c133911_g1	GO:0009057	K01183	M	c167857_g1	GO:0005833	K00326	HC
c168750_g1	GO:0005886	K02934	J	c169071_g1	GO:0016740	K00791	J
c168729_g2	GO:0031055	-	-	c169471_g1	GO:0032940	K01251	H
c168165_g1	GO:0045153	K00413	C	c156553_g1	GO:0006364	-	-
c142651_g1	GO:0005886	-	-	c151209_g1	-	K00417	C
c169479_g1	GO:0016491	-	T	c160995_g1	GO:0048146	K03937	C
c123948_g1	GO:0008340	K03868	O	c118260_g1	GO:0003700	-	KT
c165886_g1	GO:0005488	-	R	c168350_g1	GO:0002164	-	-
c164603_g1	GO:0005634	K03953	C	c167212_g1	GO:0008024	K06063	AB
c166890_g1	GO:0000098	K00540	C	c167440_g1	-	-	S
c165273_g1	GO:0005856	-	S	c169230_g1	GO:0005634	K01568	EH
c150613_g1	GO:0006626	-	-	c169782_g1	GO:0060627	K01072	O
c164454_g2	GO:0005856	-	-	c153659_g1	GO:0046332	-	K
c168970_g1	GO:0019005	K01072	O	c170230_g3	GO:0060359	K00265	E
c166987_g1	-	-	K	c167142_g1	GO:0070062	K01529	B
c163328_g7	GO:0071555	K00694	MG	c164430_g1	GO:0030981	K10436	DZ
c69763_g1	GO:0003700	K09286	K	c169744_g2	GO:0006355	K05628	R

Supplementary Tables S3 Plant hormone-related genes

geneid	orthologs	hormones	descriptions
c167826_g1	AT2G26980	D E V	+ R U P R Q H V L J (
F	$ 7B J*	D E V	+ R U P R Q H V L J (
F	$ 7B J*	D E V	+ R U P R Q H V L J (
F	$ 7B J*	D E V	+ R U P R Q H V L J (
F	$ 7B J*	D E V	+ R U P R Q H V L J (
F	$ 7B J*	D E V	+ R U P R Q H V L J (
F	$ 7B J*	D E V	+ R U P R Q H V L J (
F	$ 7B J*	D E V	+ R U P R Q H V L J (
F	$ 7B J*	D E V	+ R U P R Q H V L J (
F	$ 7B J*	D E V	+ R U P R Q H V L J (
F	$ 7B J*	D E V	+ R U P R Q H V L J (
F	$ 7B J*	D E V	+ R U P R Q H V L J (
F	$ 7B J*	D E V	+ R U P R Q H V L J (
F	$ 7B J*	D E V	+ R U P R Q H V L J (
F	$ 7B J*	D E V	+ R U P R Q H V L J (
F	$ 7B J*	D E V	+ R U P R Q H V L J (
F	$ 7B J*	D E V	+ R U P R Q H V L J (
F	$ 7B J*	D E V	+ R U P R Q H V L J (
F	$ 7B J*	D E V	+ R U P R Q H V L J (
F	$ 7B J*	D E V	+ R U P R Q H V L J (
F	$ 7B J*	D E V	+ R U P R Q H V L J (
F	$ 7B J*	D E V	+ R U P R Q H V L J (
F	$ 7B J*	D E V	+ R U P R Q H V L J (
F	$ 7B J*	D E V	+ R U P R Q H V L J (
F	$ 7B J*	D E V	+ R U P R Q H V L J (
F	$ 7B J*	D E V	+ R U P R Q H V L J (
F	$ 7B J*	D E V	+ R U P R Q H V L J (
F	$ 7B J*	D X [L+Q	R U P R Q H V L J (
F	$ 7B J*	D X [L+Q	R U P R Q H V L J (
F	$ 7B J*	D X [L+Q	R U P R Q H W U D Q V S R U W L R Q
F	$ 7B J*	D X [L+Q	R U P R Q H V L J (
F	$ 7B J*	D X [L+Q	R U P R Q H V L J (
F	$ 7B J*	D X [L+Q	R U P R Q H W U D Q V S R U W L R Q
F	$ 7B J*	D X [L+Q	R U P R Q H V L J (
F	$ 7B J*	D X [L+Q	R U P R Q H V L J (
F	$ 7B J*	D X [L+Q	R U P R Q H V L J (
F	$ 7B J*	D X [L+Q	R U P R Q H V L J (
F	$ 7B J*	D X [L+Q	R U P R Q H V L J (
F	$ 7B J*	D X [L+Q	R U P R Q H V L J (
F	$ 7B J*	D X [L+Q	R U P R Q H V L J (
F	$ 7B J*	D X [L+Q	R U P R Q H P H W D E R O L V P
F	$B7J *	D X [L+Q	R U P R Q H V L J (
F	$ 7B J*	D X [L+Q	R U P R Q H V L J (
F	$ 7B J*	D X [L+Q	R U P R Q H W U D Q V S R U W L R Q
F	$ 7B J*	D X [L+Q	R U P R Q H V L J (
F	$ 7B J*	% U D	+ R U P R Q H V L J (
F	$B7J *	% U D	+ R U P R Q H V L J (
F	$ 7B J*	% U D	+ R U P R Q H V L J (
F	$ 7B J*	% U D	+ R U P R Q H V L J (
F	$ 7B J*	E U D	+ R U P R Q H V L J (
F	$ 7B J*	% U D	+ R U P R Q H V L J (
F	$ 7B J*	% U D	+ R U P R Q H V L J (
F	$ 7B J*	E U D	+ R U P R Q H V L J (

c137843_g1	AT4G36540	brassino:	Hormone signal transduction
c151753_g2	AT5G56110	Brassino:	Hormone signal transduction
c163271_g6	AT1G55610	brassino:	Hormone receptor
c131390_g1	AT2G26710	brassino:	Hormone metabolism
c170027_g12	AT1G71830	brassino:	Hormone signal transduction
c28503_g1	AT4G30610	brassino:	Hormone signal transduction
c185671_g1	AT3G23770	Brassino:	Hormone signal transduction
c168246_g1	AT1G32130	Brassino:	Hormone signal transduction
c144077_g2	AT1G07340	Brassino:	Hormone signal transduction
c156856_g2	AT2G18550	Brassino:	Hormone signal transduction
c43818_g1	AT2G36800	brassino:	Hormone metabolism
c157668_g1	AT3G16857	cytokinin	Hormone signal transduction
c147691_g2	AT3G57040	cytokinin	Hormone signal transduction
c167866_g1	AT2G41510	cytokinin	Hormone metabolism
c148117_g1	AT5G56970	cytokinin	Hormone metabolism
c113835_g1	AT1G76420	Cytokinin	Hormone signal transduction
c165434_g2	AT1G59940	cytokinin	Hormone signal transduction
c163953_g5	AT5G49720	Cytokinin	Hormone signal transduction
c125415_g2	AT3G50070	cytokinin	Hormone signal transduction
c167004_g1	AT3G53710	ethylene	Hormone response
c135893_g2	AT3G01040	ethylene	Hormone response
c170357_g1	AT5G61600	ethylene	Hormone signal transduction
c168853_g1	AT4G32180	ethylene	Hormone response
c162736_g1	AT4G37870	ethylene	Hormone response
c170119_g1	AT3G54720	ethylene	Hormone signal transduction
c168813_g1	AT4G12400	ethylene	Hormone response
c166486_g1	AT3G12390	ethylene	Hormone response
c186728_g1	AT3G45780	ethylene	Hormone response
c169849_g1	AT1G79530	ethylene	Hormone response
c165538_g1	AT5G21010	ethylene	Hormone signal transduction
c168391_g1	AT1G54270	ethylene	Hormone response
c168786_g1	AT3G52930	ethylene	Hormone response
c147061_g1	AT1G26830	ethylene	Hormone signal transduction
c175484_g1	AT1G75830	ethylene	Hormone signal transduction
c159683_g1	AT1G56160	ethylene	Hormone signal transduction
c170163_g1	AT5G19770	ethylene	Hormone response
c170495_g1	AT1G77120	ethylene	Hormone response
c169179_g2	AT5G58350	ethylene	Hormone response
c168032_g1	AT4G26080	ethylene	Hormone signal transduction
c113156_g1	AT5G58950	ethylene	Hormone response
c161058_g1	AT2G37340	ethylene	Hormone response
c169889_g1	AT5G61790	ethylene	Hormone response
c168740_g1	AT2G16940	ethylene	Hormone response
c147819_g1	AT4G32285	ethylene	Hormone response
c170137_g1	AT5G56010	ethylene	Hormone response
c166927_g2	AT1G78080	ethylene	Hormone signal transduction
c167745_g2	AT5G44790	ethylene	Hormone signal transduction
c166544_g1	AT1G24180	ethylene	Hormone response
c169284_g1	AT1G25490	ethylene	Hormone signal transduction
c165764_g1	AT3G13224	ethylene	Hormone response
c168690_g1	AT3G11540	gibberel	Hormone signal transduction
c162742_g4	AT5G27320	gibberel	Hormone receptor
c169796_g4	AT5G01550	Gibberel	Hormone signal transduction
c186187_g1	AT3G03450	gibberel	Hormone signal transduction

c165992_g1	$ 7 *	M D V	+ R U P R Q H	V L J (
F	$ 7B J*	- D V	+ R U P R Q H	V L J (
F	$ 7B J*	M D V	+ R U P R Q H	V L J (
F	$ 7B J*	- D V	+ R U P R Q H	V L J (
F	$ 7B J*	M D V	+ R U P R Q H	V L J (
F	$ 7B J*	M D V	+ R U P R Q H	V L J (
F	$ 7B J*	M D V	+ R U P R Q H	V L J (
F	$ 7B J*	- D V	+ R U P R Q H	V L J (
F	$ 7B J*	M D V	+ R U P R Q H	V L J (
F	$ 7B J*	- D V	+ R U P R Q H	V L J (
F	$ 7B J*	V D O	+ R U P R Q H	V L J (
F	$ 7B J*	V D O	+ R U P R Q H	V L J (
F	$ 7B J*	V D O	+ R U P R Q H	V L J (
F	$ 7B J*	V D O	+ R U P R Q H	V L J (
F	$ 7B J*	V D O	+ R U P R Q H	V L J (
F	$ 7B J*	V D O	+ R U P R Q H	V L J (
F	$ 7B J*	V D O	+ R U P R Q H	V L J (
F	$ 7B J*	V D O	+ R U P R Q H	V L J (
F	$ 7B J*	V D O	+ R U P R Q H	V L J (
F	$ 7B J*	V D O	+ R U P R Q H	V L J (
F	$B7J *	V D O	+ R U P R Q H	V L J (
F	$ 7B J*	V D O	+ R U P R Q H	V L J (
F	$ 7B J*	V D O	+ R U P R Q H	P H W D E R O L V P
F	$ 7B J*	V D O	+ R U P R Q H	V L J (
F	$ 7B J*	V D O	+ R U P R Q H	V L J (
F	$ 7B J*	V D O	+ R U P R Q H	V L J (
F	$ 7B J*	V D O	+ R U P R Q H	V L J (
F	$B7J *	V D O	+ R U P R Q H	V L J (
F	$B7J *	V D O	+ R U P R Q H	V L J (
F	$ 7B J*	V D O	+ R U P R Q H	V L J (
F	$ 7B J*	V D O	+ R U P R Q H	V L J (
F	$B7J *	V D O	+ R U P R Q H	V L J (

Supplementary Tables S4. Down-steam genes of the plant hormones

geneid	orthologs	genename	e-value	hormone
c170357_g1	AT2G44840	ERF13	3.00E-29	ET
c39931_g1	AT3G16770	ERF72	3.00E-35	ET
c166849_g5	AT2G35700	ERF38	1.00E-45	ET
c181204_g1	AT5G50080	ERF110	1.00E-27	ET
c169538_g17	AT3G23240	ERF1	4.00E-50	ET
c190677_g1	AT5G44210	ERF9	2.00E-31	ET
c113156_g1	AT5G03730	CTR1	6.00E-36	ET
c170419_g1	AT4G33950	SNRK2.6	1.00E-27	ABA
c167826_g1	AT2G23030	SNRK2.9	8.00E-60	ABA
c167848_g1	AT3G43810	CAM7		ABA
c159397_g4	AT5G21274	CAM6	8.00E-38	ABA
c186097_g1	AT1G45249	AREB1	4.00E-11	ABA
c29329_g1	AT1G04240	Auxin_Auxin3	1.00E-55	Auxin
c151796_g4	AT1G04250	Auxin_Auxin17	3.00E-15	Auxin
c164349_g5	AT2G33860	ARF3	5.00E-15	Auxin
c177109_g1	AT5G65210	TGA1	5.00E-11	SA
c149565_g1	AT1G80840	WRKY40	2.00E-84	SA
c63509_g1	AT5G15850	COL1	3.00E-42	JA
c152423_g1	AT1G17380	JAZ5	5.00E-25	JA
c166318_g3	AT1G30135	JAZ8	9.00E-31	JA
c147987_g2	AT5G13220	JAZ10	3.00E-31	JA
c157668_g1	AT4G16110	ARR2	2.00E-27	CK

Supplementary Tables S5. The transcription factor genes and their expression patterns

geneid	TF names	3dt_re_ex*	14dt_re_ex	28dt_re_ex	e-value
c48539_g1	ERF	0.72	0.985	1.975	3.00E-46
c169740_g1	MYB	0.165	0.275	0.95	5.00E-32
c162155_g1	GRAS	0.05	0.23	0.62	1.00E-45
c163088_g3	TALE	1.005	2.23	0.785	3.00E-32
c151432_g2	C2H2	1.385	9.905	5.925	1.00E-77
c161722_g1	SBP	1.075	0.545	0.27	9.00E-48
c168178_g1	NAC	0.76	8.855	8.305	2.00E-93
c169585_g1	ERF	0.205	0.395	1.225	7.00E-23
c168075_g5	GATA	5.55	13.62	17.11	########
c169598_g3	FAR1	0.46	0.195	0.35	1.00E-40
c192506_g1	C2H2	2.915	6.12	7.28	6.00E-87
c159706_g1	C2H2	1.01	6.84	5.61	3.00E-67
c163328_g8	bZIP	4.825	8.34	19.76	5.00E-75
c175580_g1	C2H2	9.79	22.86	40.765	########
c164682_g1	GRAS	0.345	0.81	2.12	4.00E-23
c166404_g1	MYB	0.1	0.325	0.98	2.00E-40
c39931_g1	ERF	18.38	25.43	126.745	########
c49768_g1	WRKY	1.65	3.24	6.655	2.00E-86
c118814_g1	bHLH	6.91	8.08	13.42	########
c126878_g1	NAC	4.105	23.335	54.405	########
c166898_g1	bHLH	0.07	0.385	0.47	2.00E-39
c168629_g1	B3	0.085	0.07	0.65	########
c144904_g2	GATA	38.04	85.8	254.505	########
c170155_g6	C3H	0.17	1.665	1.485	6.00E-40
c150516_g1	ERF	0.045	0.625	0.045	3.00E-36
c170104_g1	bZIP	0.11	1.145	2.4	0
c165640_g1	C3H	0.04	0.095	0.565	3.00E-26
c160451_g1	WRKY	37.44	67.93	51.565	7.00E-76
c117714_g2	NAC	1.295	2.99	3.08	0
c66386_g1	ERF	1.31	1.27	29.27	5.00E-65
c169423_g2	FAR1	0.065	0.38	1.12	6.00E-53
c168349_g2	ERF	0	0.275	0.68	3.00E-33
c169573_g18	Dof	4.185	6.8	9.84	1.00E-56
c170040_g2	bHLH	0.155	0.56	1.15	6.00E-30
c163241_g1	C3H	0.52	1.58	1.285	2.00E-49
c170568_g1	bZIP	41.465	2.59	5.735	2.00E-61
c136792_g1	HSF	0.83	2.81	2.77	########
c166446_g1	TCP	0.44	0.855	0.875	########
c175455_g1	GRAS	36.975	36.66	37.805	2.00E-28
c165148_g1	WRKY	0.1	0.385	0.56	########
c169082_g2	LBD	1.03	4.995	19.005	0
c172779_g1	NAC	1.285	6.145	10.905	1.00E-39
c69763_g1	ERF	0.745	0.25	2.265	1.00E-94
c165257_g1	FAR1	0.405	0.38	0.8	2.00E-58
c163330_g1	B3	0.775	3.34	4.5	6.00E-27
c169972_g2	GATA	0.07	0.28	0.735	########
c155293_g2	bHLH	2.575	8.835	5.32	########
c196900_g1	FAR1	1.635	1.375	7.58	6.00E-21
c166849_g5	ERF	3.54	4.11	3.585	########
c122210_g1	bHLH	3.515	1.255	0.575	0
c168084_g1	FAR1	0.12	0.37	0.6	2.00E-62
c149663_g1	WRKY	3.24	3.7	10.165	########
c157614_g1	WRKY	13.245	8.97	41.655	2.00E-36

c169081_g1	C2H2	0.155	0.44	1.14	1.00E-25
c165505_g1	bZIP	0.445	2.555	4.79	4.00E-59
c169722_g1	GRAS	0.18	0.4	0.865	2.00E-39
c160284_g4	LBD	4.915	38.82	7.415	2.00E-25
c169202_g7	LBD	9.285	11.07	75.435	########
c161750_g1	B3	0.07	0.215	0.34	########
c186097_g1	bZIP	2.405	5.83	1.435	1.00E-71
c170107_g2	MYB	0.09	1.42	2.16	6.00E-27
c133911_g1	C2H2	1.915	1.955	0.85	3.00E-38
c160061_g1	WRKY	1.04	2.085	0.86	1.00E-65
c165565_g1	bHLH	0.445	1.04	0.97	3.00E-29
c168695_g1	NAC	0.06	0.255	0.555	3.00E-78
c42787_g1	NAC	1.655	9.5	8.38	0
c163624_g1	WRKY	0.22	0.23	0.745	2.00E-32
c156986_g1	C3H	0.26	0.365	0.58	7.00E-65
c161823_g3	MYB	2.825	2.62	4.125	########
c170216_g2	WRKY	0.96	0.35	0.75	9.00E-68
c169282_g1	MYB	0.415	1.335	2.18	1.00E-22
c196474_g1	ARF	10.45	31.03	62.61	2.00E-69
c169041_g30	SBP	5.01	10.095	13.155	2.00E-33
c166837_g3	bZIP	6.4	15.715	31.64	2.00E-65
c150564_g1	NAC	5.195	10.475	10.325	########
c31398_g1	NAC	0.69	2.685	1.225	########
c150774_g2	WRKY	2.795	12.6	17.07	########
c169595_g1	B3	0.145	0.465	0.645	5.00E-39
c160846_g1	bZIP	4.13	21.6	14.165	2.00E-51
c132727_g1	MYB	0.775	1.43	2.905	5.00E-60
c162831_g1	NAC	1.705	7.95	4.095	8.00E-83
c125700_g1	B3	13.5	15.555	4.295	1.00E-38
c160033_g1	C3H	0.17	0.295	0.555	4.00E-55
c134858_g1	MYB	421.995	280.525	107.99	########
c160723_g2	ERF	6.01	5.305	157.87	########
c169579_g1	ERF	0.075	1.075	2.35	7.00E-32
c180504_g1	FAR1	9.43	15.495	54.11	5.00E-38
c26872_g1	NAC	1.225	1.51	2.725	0
c168410_g1	C3H	19.55	95.245	32.65	4.00E-53
c168798_g1	FAR1	0.17	0.7	1.435	2.00E-58
c186006_g1	MYB	27.435	4.11	1.74	2.00E-37
c168575_g1	MYB	0.285	1.18	1.795	6.00E-21
c28300_g1	BES1	8.47	0.855	0.845	4.00E-64
c146875_g6	ERF	0.065	0.1	8.175	1.00E-45
c151967_g1	bHLH	0.755	0.615	9.57	########
c156390_g1	GRAS	0.76	3.1	4.65	1.00E-38
c23797_g1	WRKY	1.215	6.45	13.96	3.00E-33
c66164_g1	WRKY	3.445	15.805	4.18	2.00E-76
c169024_g1	MYB	0.035	0.19	0.61	2.00E-21
c170980_g1	FAR1	7.265	13.995	60.125	9.00E-49
c166902_g1	MYB	0.24	1.56	3.35	2.00E-69
c165475_g1	GATA	1.75	7.185	12.13	2.00E-31
c114884_g1	NAC	1.215	5.26	7.695	4.00E-51
c169075_g1	B3	0.5	4.495	7.105	3.00E-26
c168949_g3	NAC	0.675	2.42	7.055	8.00E-38
c167166_g4	bHLH	0.48	0.64	1.59	########
c168999_g3	FAR1	27.08	1.67	6.72	1.00E-86

c180622_g1	HSF	185.545	4.07	0.865	4.00E-35
c175299_g1	bZIP	19.76	40.9	63.175	3.00E-46
c161119_g2	bHLH	8.105	9.605	46.535	2.00E-96
c162733_g1	SBP	30.195	28.375	271.83	0
c162954_g2	C2H2	0.845	0.34	0.295	6.00E-21
c149319_g1	C2H2	4.79	3.095	1.245	########
c167977_g1	ERF	0.115	0.635	1.15	0
c169405_g1	bZIP	0.41	0.98	1.865	2.00E-30
c66164_g2	WRKY	3.475	20.715	5.115	########
c123108_g1	MIKC	41.39	4.595	0.655	2.00E-48
c169756_g2	VOZ	0.315	0.245	0.42	6.00E-39
c161373_g1	B3	0.16	0.45	0.815	0
c131236_g1	ERF	3.59	10.46	2.92	########
c170576_g1	bZIP	18.965	55.915	75.91	2.00E-85
c167142_g1	C3H	0.255	0.57	1.075	5.00E-31
c165236_g2	B3	0.13	0.245	0.63	3.00E-42
c163431_g1	bZIP	13.69	22.595	39.545	########
c165044_g1	C2H2	0.05	0.36	0.73	3.00E-61
c166208_g3	GRAS	0.03	0.14	0.22	5.00E-24
c128577_g1	ERF	0.035	0.185	2.275	3.00E-88
c169730_g3	C2H2	0.455	1.005	1.745	1.00E-31
c159571_g4	TALE	12.69	19.33	24.835	########
c153446_g2	MYB	2.785	2.06	2.8	########
c150185_g2	ERF	0.245	0.3	4.905	########
c166232_g1	GATA	0.05	0.205	0.66	1.00E-83
c145163_g1	HSF	10.685	26.865	10.505	5.00E-31
c159031_g1	MYB	1.365	3.455	0.9	0
c161709_g1	bHLH	0.065	0.28	0.585	4.00E-42
c165941_g1	bZIP	0	0.29	0.87	4.00E-25
c46919_g1	Trihelix	1.525	6.91	19.645	0
c168410_g2	C3H	1.64	4.8	3.26	2.00E-50
c153006_g1	C3H	0.14	0.305	0.605	2.00E-70
c169263_g3	C3H	0.06	0.83	1.615	2.00E-31
c162988_g1	MYB	0.15	0.145	0.385	1.00E-96
c68021_g1	bHLH	7.775	46.535	214.925	3.00E-28
c163994_g4	NAC	0.97	9.355	8.36	2.00E-93
c28300_g2	BES1	10.215	1.245	1.21	3.00E-50
c152254_g1	bHLH	3.125	1.31	15.21	########
c170040_g1	bHLH	0.06	0.255	0.57	3.00E-43
c157743_g1	MYB	0.87	0.74	2.92	2.00E-62
c39259_g1	C3H	0.945	1.145	1.785	0
c48886_g1	GRAS	12.575	17.495	10.635	6.00E-38
c158341_g1	MYB	0.085	0.165	0.505	########
c155825_g2	NAC	6.68	10.465	10.925	########
c191395_g1	WRKY	1.35	4.905	15.495	2.00E-70
c170193_g3	bHLH	0.235	0.675	1.73	2.00E-25
c170216_g1	NAC	0.43	0.285	0.57	3.00E-72
c168970_g1	GATA	0.05	0.215	0.585	9.00E-35
c168701_g1	FAR1	0.35	0.69	1.625	1.00E-81
c180626_g1	HSF	17.66	5.305	2.51	2.00E-26
c195758_g1	SBP	18.565	23.085	7.68	1.00E-45
c163088_g2	TALE	1.12	3.94	1.44	5.00E-49
c118439_g1	NAC	0.91	2.29	7.14	2.00E-59
c160214_g2	ERF	0.44	2.835	1.945	########

c170025_g1	NAC	0.28	0.74	1.875	8.00E-37
c187273_g1	ARF	1.535	9.145	5.91	5.00E-23
c168617_g1	C3H	0.23	0.555	1.545	1.00E-22
c163328_g7	bZIP	3.535	7.39	18.08	4.00E-92
c175319_g1	bHLH	18.525	16.9	95.31	0
c168528_g1	GeBP	0.585	3.2	2.815	5.00E-95
c196163_g1	ARF	61.865	44.48	30.06	1.00E-33
c180211_g1	WRKY	25.21	58.95	119.855	0
c169352_g1	B3	0.035	0.455	0.84	5.00E-26
c169768_g6	NAC	2.61	1.76	0.775	8.00E-56
c196088_g1	C2H2	0.82	0.275	0.885	2.00E-81
c169675_g1	FAR1	0.305	0.765	1.625	5.00E-61
c153321_g1	bZIP	0.14	0.235	0.995	1.00E-68
c124137_g1	SBP	0.55	0.07	1.03	1.00E-23
c170028_g1	FAR1	0.55	1.475	2.42	0
c135971_g1	MYB	1.045	1.905	2.445	########
c166457_g1	ERF	0.37	1.38	2.43	8.00E-74
c24923_g1	ERF	36.935	79.375	282.845	1.00E-24
c30023_g1	Trihelix	1.795	13.345	68.305	0
c149402_g3	bHLH	2.955	1.16	6.54	1.00E-61
c166206_g1	MYB	0.29	0.585	1.465	3.00E-28
c166329_g1	C2H2	0.165	0.53	0.895	5.00E-38
c195994_g1	MYB	9.315	14.24	43.075	########

* mean the relative expression of treatment compareed to the control

Supplementary Tables S7. The differential expressed DEUs

geneid	gene name	kog group	cluster	3d_treamnt	14d_treat	28d_treamnt
c134084_g1	c1	KOG1700	TZ	-0.06031873	6.895919	7.753891
c151100_g1	c2	KOG1547	DTZ	-0.55537478	0.437255	6.594704
c153804_g1	c3	KOG3380	Z	0.863037621	0.116663	6.778495
c154944_g1	c4	KOG0836	Z	0.202744205	-0.14876	7.00927
c157921_g2	c5	KOG0613	Z	0.912170149	-0.89125	6.594704
c159366_g1	c6	KOG2026	Z	0.01492239	0.778844	6.292844
c159967_g2	c7	KOG1374	Z	0.853744652	9.324316	10.60899
c161770_g1	c8	KOG0073	UZ	-0.51660567	7.243548	7.51159
c162137_g1	c9	KOG0613	Z	-0.76707254	7.63674	8.331222
c162155_g1	c10	KOG1523	Z	-0.93332926	0.553351	6.778493
c162590_g2	c11	KOG0613	Z	-0.91629389	0.325775	6.715766
c163667_g1	c12	KOG3430	Z	-0.99693164	7.754506	8.754152
c164227_g1	c13	KOG0247	Z	0.611677535	0.812473	6.530366
c164258_g2	c14	KOG0613	Z	0.172305615	0.89464	6.219734
c164430_g1	c15	KOG3000	DZ	-0.04931394	-0.25685	6.772799
c164578_g1	c16	KOG1700	TZ	-0.95997636	-0.15081	7.183258
c164642_g7	c17	KOG0244	Z	-0.61253187	-0.61409	6.225964
c164761_g1	c18	KOG0073	UZ	0.869103596	7.281899	7.986063
c164954_g1	c19	KOG0031	Z	-0.03998501	-0.65776	7.352033
c165298_g1	c20	KOG1654	Z	0.190143342	6.715149	8.160585
c165312_g1	c21	KOG0046	Z	0.461207116	6.889698	8.277846
c165649_g2	c22	KOG3595	Z	-0.46397404	-0.00313	6.830099
c166530_g2	c23	KOG1755	Z	0.832999218	0.934648	7.074273
c166530_g3	c24	KOG1755	Z	0.340923757	0.289457	7.420673
c166600_g1	c25	KOG1003	Z	10.22670354	9.877979	10.09072
c166781_g1	c26	KOG0318	Z	0.197441247	-0.87656	6.384152
c166886_g1	c27	KOG2046	Z	0.956498895	0.58298	6.526726
c167436_g1	c28	KOG0613	Z	-0.11566251	-0.89817	7.028296
c167606_g1	c29	KOG0444	Z	-0.59279187	6.644079	7.544572
c167639_g1	c30	KOG2046	Z	9.18085431	8.369447	8.036279
c167820_g2	c31	KOG0518	Z	0.816160849	-0.93672	6.384154
c167820_g3	c32	KOG0518	Z	-0.74623632	6.67481	7.608302
c167820_g4	c33	KOG0518	Z	0.917249344	6.775352	8.054449
c167826_g1	c34	KOG0613	Z	-0.76628164	6.99735	8.271161
c167848_g1	c35	KOG0031	Z	-0.5304544	-0.15871	5.145364
c167993_g1	c36	KOG1727	DZ	0.098245982	7.784249	8.776206
c168082_g1	c37	KOG3977	Z	0.568087091	6.833243	6.866354
c168512_g1	c38	KOG1702	Z	-0.66558531	-0.16221	7.367739
c169085_g1	c39	TWOG0145	Z	0.713151172	8.360136	9.032583
c169121_g1	c40	KOG0031	Z	0.321634049	-0.13676	7.080495
c169385_g1	c41	KOG3699	TZ	0.51575031	8.07323	10.03776
c169454_g1	c42	KOG0680	Z	-0.88363053	-0.23943	6.378291
c169500_g1	c43	KOG1425	Z	-0.95691884	-0.08346	6.713212
c169568_g2	c44	KOG0613	Z	0.10338459	8.315037	7.468422
c169573_g9	c45	KOG0680	Z	9.4373089	7.813564	10.59921
c169616_g1	c46	LSE0592	Z	0.093031989	8.984292	10.35159
c169637_g2	c47	KOG0040	Z	0.980874591	7.875494	8.907882
c169638_g1	c48	KOG1700	TZ	-0.61005191	0.627359	7.907518
c169670_g1	c49	KOG1840	Z	0.797026815	7.375125	7.256422
c169677_g1	c50	KOG1374	Z	-0.51166525	10.59227	11.6245
c169775_g1	c51	KOG3634	Z	7.241195705	8.057926	8.36841
c169775_g2	c52	KOG3634	Z	-0.67676864	-0.84013	6.375579
c169948_g1	c53	KOG0161	Z	0.701823855	7.16076	8.187837

c169958_g5	c54	KOG0516	Z	0.225448902	0.158698	7.365853
c170022_g1	c55	KOG0516	Z	0.065512416	-0.54817	7.170058
c170022_g6	c56	KOG0516	Z	-0.37561864	6.550093	8.096845
c170035_g3	c57	KOG0040	Z	-0.58646054	0.929777	7.763832
c170035_g4	c58	KOG0040	Z	0.463643375	7.189304	8.898573
c170080_g3	c59	LSE0589	NZ	0.235791641	7.679149	8.514464
F	F B J	. 2 * =				
F	F B J	. 2 * =				
F	F B J	. 2 * =				
F	F B J	. 2 * =				
F	F B J	. 2 * =				

\ H O O R Z U H S U H V H Q W V 0 \ R V L Q J H Q H V D Q G

Supplementary Tables S8. The pathogen-related DEUs

geneid	gene	e-value	3d	14d	28d
c48539_g	PBS1	6.00E-37	0	0	3.154312
c149663_g	WRKY2	2.00E-29	0	0	2.322398
c169740_g	MPK4	0	0	7.321713	8.970135
c190886_g	PBS1	3.00E-61	0	2.346388	0
c158848_g	FLS2	2.00E-20	0	0	2.117866
c170195_g	WRKY22	1.00E-40	0	0	-2.14599
c155643_g	BAK1	1.00E-55	0	0	2.168102
c151753_g	MYB	1.00E-93	0	0	-2.39997
c170018_g	BAK1	6.00E-59	0	2.597475	2.290406
c170743_g	MKK6	0	0	0	6.594707
c145361_g	PBS1	########	0	0	3.195368
c167826_g	CDPK9	0	0	6.99735	8.271161
c159683_g	MYB	3.00E-70	0	0	2.07808
c168999_g	PBS1	9.00E-49	0	0	3.916032
c169598_g	FRK1	4.00E-12	0	0	-2.10727
c164954_g	CAM7	2.00E-88	0	0	7.352033
c170137_g	Hsp81.4	0	0	10.52143	9.113101
c160553_g	putative	2.00E-24	0	0	6.591545
c196251_g	MYB	1.00E-53	0	0	3.088777
c169756_g	CPK14	4.00E-99	0	0	6.452761
c163624_g	MPK3	0	0	0	7.034665
c161823_g	MYB	8.00E-68	0	0	2.413364
c139115_g	CML41	2.00E-12	0	3.674934	0
c169130_g	SHD	########	0	0	6.447409
c196474_g	AGD11	5.00E-28	0	0	2.063878
c42101_g	EFR	########	0	2.860893	0
c169130_g	Hsp89.1	########	0	7.519939	7.987817
c150320_g	DND1	0	0	0	3.564644
c191092_g	RD19	0	0	0	2.004668
c132993_g	MYB	3.00E-73	0	2.057775	0
c167541_g	SERK4	2.00E-40	0	7.280575	4.826916
c126086_g	FLS2	9.00E-20	0	0	3.481331
c150774_g	WRKY2	5.00E-28	0	2.864878	2.859664
c157921_g	CDPK2	0	0	0	6.594704
c162191_g	SERK4	1.00E-38	0	9.305124	3.026726
c159397_g	CAM6	1.00E-35	0	2.069602	0
c160451_g	WRKY2	7.00E-29	0	2.177943	0
c134858_g	MYB	1.00E-60	0	0	-2.0809
c168605_g	FLS2	5.00E-36	0	5.08366	5.29383
c168468_g	CPK19	0	0	7.089478	8.207304
c162190_g	RBOH_F	5.00E-58	0	0	2.417715
c169796_g	PBS1	2.00E-52	0	2.917551	0
c149565_g	ZAP1	7.00E-25	0	0	2.736457
c165983_g	MPK6	########	0	6.81905	7.622297
c113156_g	CERK1	########	0	0	6.305477
c164630_g	MYB	8.00E-79	0	0	3.375092
c169273_g	RD19	########	0	0	7.57315
c170027_g	BAK1	5.00E-65	0	2.03484	0
c133321_g	WRKY33	3.00E-29	0	3.373361	3.262762
c161191_g	MYB	1.00E-53	0	5.177657	0
c125081_g	CERK1	2.00E-33	0	0	2.037598
c163271_g	FLS2	0	0	0	2.039382
c167606_g	FLS2	4.00E-45	0	6.644079	7.544572

c135971_{MYB (
F 0 3 .
F 0 < % (
F & $ 0 (
F 0 3 .
F & (5 . (
F & $ 0 (
F 3 %B 6J (
F 0 (. . (
F 5 ’
F 0 < % (
F 0 < % (
F 0 < % (
F 3 % 6 (
F) / 6 (
F 5 ’ (
F ’ 1 ’
F & 3 .

supplementary Table 10

>c181328_g1_f
TTGGATTTTTGCTTTTGGTC
>c181328_g1_r
CGATGTAAACGGTCTCAATG
>c167541_g2_f
TACTCCGACCGCTCTCTGTT
>c167541_g2_r
ATTGAAGCAAGCAGGCAGAT
>c167606_g1_f
AACGAACTCTGTGCTTTTCCA
>c167606_g1_r
TCCAAAGTTTTGTTAATGCCG
>c145581_g1_f
TCCACGAAATACACACCATCAT
>c145581_g1_r
TTGAACACATCAATCTTTTCCG
>c150774_g2_f
AACAACTTGATCAGGACTCATC
>c150774_g2_r
AATTCCGACTTTGTTATAAATGC
>c150251_g1_f
AGGTGTTACACAATTGCCAGACT
>c150251_g1_r
AACACCATTTAATTTCCGTCATCT
>c157668_g1_f
CAAGAGTGGTCCTTTAGCTACTGAG
>c157668_g1_r
TCATAAGCTCAAGTATTTTGGAAGG
>c170107_g1_f
AGGAATTCAAGTTGCTGTTATTGAC
>c170107_g1_r
ATAAGCTCATCAGGTGTACCAGAAC
>c166902_g1_f
TGCTTTGCTTAAAGTTCCAAATATC
>c166902_g1_r
CAGCAGTAACAGATCCTCCATAAAT
>c195758_g1_f
AATGGAAATTTGGAATGGAGG
>c195758_g1_r
CCAACTTTAAGGCCATGAAC
>c159397_g4_f
CCGCCGTTTCTCTCTCTCT
>c159397_g4_r
CCAAGCTCCTTGGTTGTGAT
>c169071_g1_f
GATAGAGGAAGAGGAGAAACAATCC
>c169071_g1_r
CCCCGTAGTTCGATAAATTTCTAAT
>c134858_g1_f
CACATGAGAAAAGTAGCACAAGAG
>c134858_g1_r
CCATCGTATAACAATAACCCTTTTG
>c39931_g1_f
GCTCCCATTCCTCAATTTC

>c39931_g1_r
CCTCAGAAAGCTTTTGCACC
>c166034_g2_f
TTGCTCAATTATCTTTAAGTCCAGG
>c166034_g2_r
CAATTCATTACACAACGATCACAAT
>c165983_g1_f
CGTTGGATGAGTTTGACGATT
>c165983_g1_r
TTTTCATCCTCAACATTTCGC
>c149565_g1_f
TAAAGATGGATATCAATGGAGG
>c149565_g1_r
CATATGTGGCTACAAGAACTG
>c170018_g7_f
GGCGGAATATTTGTATTGGTGT
>c170018_g7_r
TGTGGGTATTTCCTTCTTCTTC
>c164561_g1_f
TTTCCGATTCGTCAGAAACC
>c164561_g1_r
CGGGTTTCAAGATTGTCGTT
>c133321_g2_f
CCCATTCTCTGTTCCGTACAA
>c133321_g2_r
CATCAAATTGCGACGAAAAAT
>c117343_g1_f
GGCCAACTTTTCTTCATTTTTCT
>c117343_g1_r
CATGGGGGTGATTAATTTACAG
>c167826_g1_f
CTGCTTATTTATTCCCGCCA
>c167826_g1_r
TTGATGGAGACGAGCAGTTG
>c164349_g5_f
TGTATTTGAGGACAGAATTAGGAGG
>c164349_g5_r
TGATGATGATGATGATTTTCCATAC
>c166318_g3_f
TTCAGGCAAGAGCAATTCTAAAG
>c166318_g3_r
TGAACCCTATGCTTTCTCTTCTG
